# Elucidation of the stereocontrol mechanisms of the chemical and biosynthetic intramolecular Diels–Alder cycloaddition for the formation of bioactive decalins†

**DOI:** 10.1039/d3ra04406h

**Published:** 2023-09-19

**Authors:** Takumi Kariya, Hayato Hasegawa, Taro Udagawa, Yusaku Inada, Kyoko Nishiyama, Mieko Tsuji, Tasuku Hirayama, Tatsuo Suzutani, Naoki Kato, Shingo Nagano, Hideko Nagasawa

**Affiliations:** a Laboratory of Pharmaceutical and Medicinal Chemistry, Gifu Pharmaceutical University 1-25-4 Daigaku-nishi Gifu 501-1196 Japan hnagasawa@gifu-pu.ac.jp; b Department of Engineering, Graduate School of Sustainability Science, Tottori University 4-101 Koyama-cho Minami Tottori 680-8552 Japan; c Department of Chemistry and Biomolecular Science, Faculty of Engineering, Gifu University 1-1 Yanagido Gifu 501-1193 Japan; d Department of Microbiology, Fukushima Medical University 1 Hikarigaoka Fukushima 960-1295 Japan; e Faculty of Agriculture, Setsunan University 45-1 Nagaotoge-cho, Hirakata Osaka 573-0101 Japan; f Department of Chemistry and Biotechnology, Graduate School of Engineering, Tottori University 4-101 Koyama-cho Minami Tottori 680-8552 Japan; g Center for Research on Green Sustainable Chemistry, Tottori University 4-101 Koyama-cho Minami Tottori 680-8552 Japan

## Abstract

The intramolecular Diels–Alder reaction (IMDA) is a powerful method for regioselective and stereoselective construction of functionalised decalin skeletons, and the recent discovery of enzymes that catalyse IMDA cycloaddition in biosynthesis has generated considerable interest. This study focused on the role of the absolute configuration of the C-6 carbon of the substrate polyene in the stereocontrol of the IMDA reaction catalysed by Fsa2 and Phm7, which construct different enantiomeric decalin skeletons. Their enantiomeric precursor polyenes were synthesised and subjected to enzymatic or thermal IMDA reactions to isolate various diastereomeric decalines and determine their absolute configuration. Furthermore, density functional theory calculations were performed to elucidate the stereocontrol mechanism underlying the formation of decalin. The results showed that Fsa2 exhibits the same equisetin-type stereoselectivity for enantiomeric substrates regardless of the 6-methyl group configuration of the substrate, while Phm7 shows two types of stereoselectivity depending on the configuration of the 6-methyl group. We also found a unique stereochemistry–activity relationship in antibacterial activity for decalin diastereomers, including new derivatives. This study provides new insights into the stereoselectivity of DAase, which is important in the synthesis of natural product skeletons.

## Abstract 2

This study elucidates the role of the absolute configuration at the C-6 carbon of the substrate polyene in the stereocontrol of the IMDA reaction catalyzed by Fsa2 and Phm7, which construct different enantiomeric decalin skeletons.

## Graphical abstract

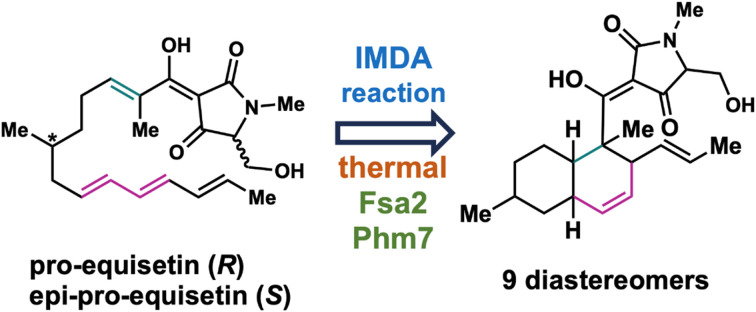

## 1. Introduction

The Diels–Alder (DA) reaction, a [4 + 2] cycloaddition reaction, is a powerful carbon–carbon bond-forming reaction in synthetic chemistry, allowing regio- and stereoselective construction of functionalised cyclohexene frameworks.^1^ This cycloaddition reaction is also important in the biosynthesis of complex natural products, and the existence of Diels–Alderase (DAase), which catalyses the DA reaction to form the characteristic polycyclic structure, has long been discussed.^2–4^ In recent years, many studies have discovered and reported a series of DAases that play essential roles in secondary metabolic biosynthetic pathways that produce complex bioactive natural product.^5–7^

**Fig. 1:**
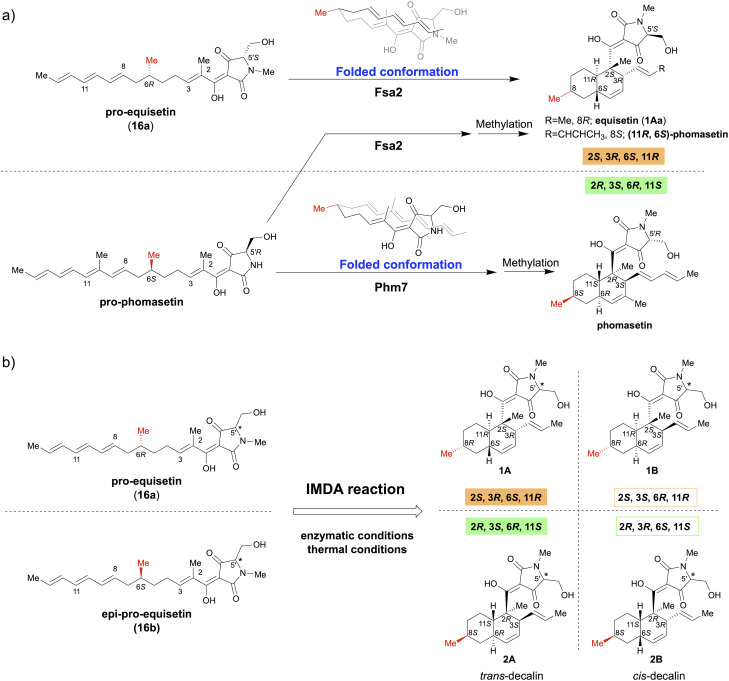
Stereoselective IMDA reaction to form tetramic acid-bearing decalin diastereomers. (a) Enzymatic IMDA cycloadditions by Fsa2 and Phm7 to form enantiomeric decalin scaffolds.^9^ (b) Stereoselective synthesis of various diastereomeric decalins by enzymatic or thermal IMDA reaction.

Fsa2 and Phm7 are unique DAases that catalyse the intramolecular Diels–Alder (IMDA) reaction during the biosynthesis of equisetin (1Aa) and its enantiomeric homolog, phomasetin, respectively.^8,9^ They have a pyrrolidine-2,4-dione (tetramic acid)-bearing decalin scaffold and exhibit various biological activities, including potent anti-HIV activity.^10–12^ Kato *et al.* identified Fsa2 as a DAase involved in the stereoselective formation of the decalin ring of 1Aa from *Fusarium* sp. FN080326 (ref. 8) and Phm7 as a DAase involved in the corresponding cycloaddition reaction of phomasetin from *Pyrenochaetopsis* sp. RK10-F058.^9^ The most striking feature of these pericyclases is that they catalyse reversely stereoselective [4 + 2] cycloadditions using enantiomeric polyene to generate four pairs of inverse chiral centres. Furthermore, the gene replacement of *phm7* with *fsa2* in the phomasetin-producing fungus resulted in equisetin (1Aa)-type stereoselectivity (2*S*, 3*R*, 6*S*, 11*R*),^13^ indicating that Fsa2 and Phm7 fold the polyenes inversely to construct the corresponding enantiomeric decalin ring, irrespective of the absolute configuration of the methyl group at C-6 of the linear polyene substrate (Fig. 1a).^9^

This study focused on the role of the absolute configuration at the C-6 carbon of the substrate polyene in the stereocontrol of Fsa2 and Phm7, which catalyse IMDA cycloadditions to construct different enantiomeric decalin scaffolds. Accordingly, a pair of C-6 epimer polyene precursors (16a and 16b) were synthesised as substrates for the IMDA reaction and subjected to thermal or enzymatic IMDA reactions with Fsa2 and Phm7. All resulting stereoisomers were isolated, and their absolute configurations were determined to elucidate their stereoselectivity under each condition (Fig. 1b). Furthermore, density functional theory (DFT) calculations were performed to elucidate the stereoselectivity mechanism of the IMDA reaction in detail. The antibacterial activity of these diastereomeric decalins was also evaluated and the correlation between stereochemistry and activity was investigated.

## 2. Results and discussion

### 2.1. Synthesis of precursors **16a** and **16b**

**Scheme 1:**
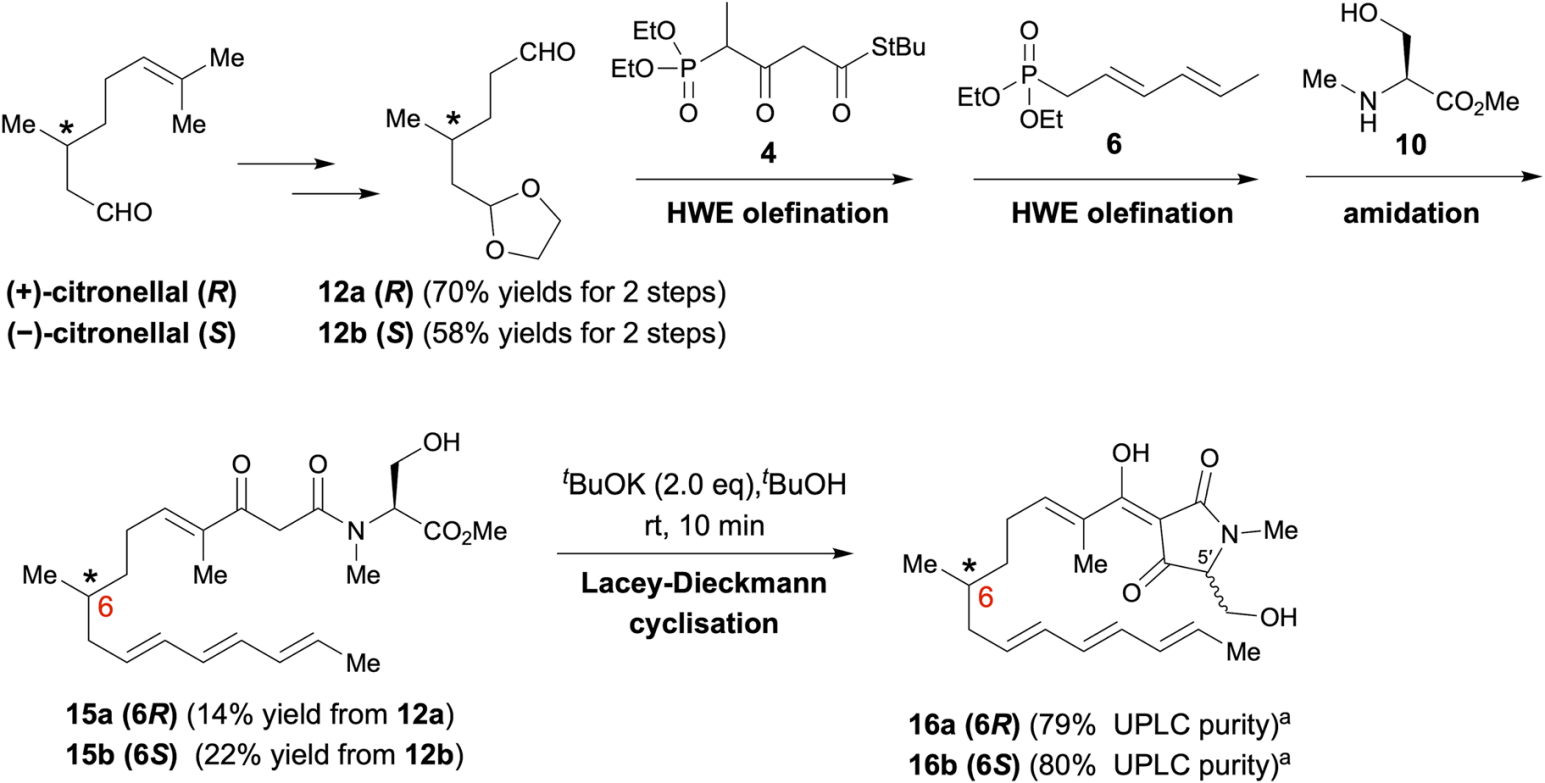
Synthesis of pro-equisetin (16a) and *epi*-pro-equisetin (16b). ^*a*^Purities were analysed by UPLC (Fig. S4†). It was used in the next reaction without purification.

**Fig. 2:**
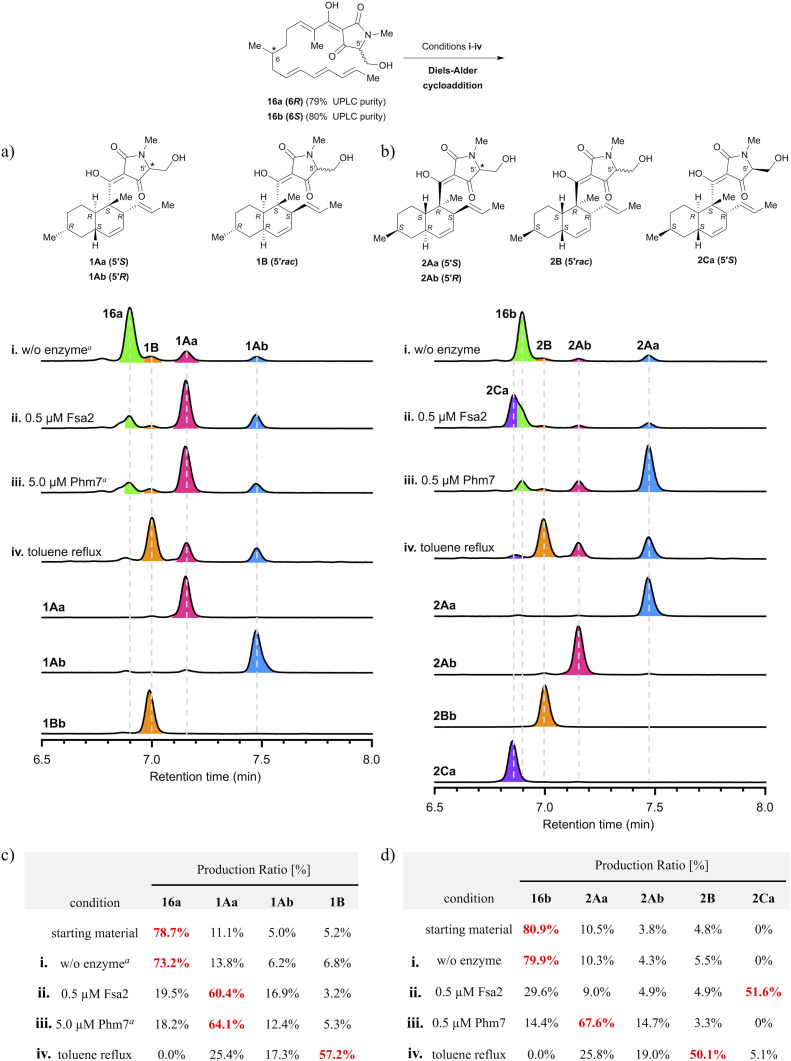
Analysis of stereoselectivity of enzymatic or thermal IMDA reaction of polyene precursor 16a or 16b. (a, b) UPLC analysis of Fsa2/Phm7-catalysed or thermal reaction products. UV detection was carried out at 290 nm. The substrate solution was incubated under various conditions: (i) without enzyme at 25 °C for 60 min; (ii) with 0.5 μM Fsa2 at 25 °C for 60 min; (iii) with 0.5 or 5.0 μM Phm7 at 25 °C for 60 min; (iv) reflux in toluene for 60 min. ^*a*^The incubation time was extended to 24 h. (c, d) Ratio of diastereomers under each condition. Diastereomeric ratios were calculated from the integrated peak areas of (a) or (b). See Fig. S6 and S7 in the ESI† for the time course up to 24 h for the production ratios of decalin diastereomers for each reaction condition.

As depicted in Scheme 1, polyenoyltetramic acids 16a and 16b, the precursors of the IMDA reaction, were synthesised according to the method of Gao *et al.*^14,15^ (+)- or (−)-Citronellal was converted to aldehyde 12, and then, successive deacetalization and Horner–Wadsworth–Emmons (HWE) olefination was performed using separately synthesised phosphonate 4 (ref. 16) and phosphonate 6 (ref. 16) to afford 14a and 14b (Schemes S1 and S2†). Subsequent amidation of 14a and 14b with the l-serine derivative 10 (ref. 17) yielded polyenoylamino esters 15a and 15b (Schemes S3 and S4†). Finally, Lacey–Dieckmann cyclisation of 15a and 15b with a base yielded the desired 16a and 16b with some epimerisation at the 5′ position. The epimerisation of tetramic acid at C-5′ (generally 10–30%) under strongly basic conditions during the Lacey–Dieckmann cyclisation reaction depends on the reaction time, substrate and base used in the reaction. Therefore, with reference to the review by Lambert *et al.*,^18^ we investigated conditions with low epimerisation and high conversion rates and chose the conditions shown in Scheme 1. The products of the Lacey–Dieckmann reaction (16a, 16b) were subjected to ultra-performance liquid chromatography (UPLC) analysis without further purification, and both contained a small amount of DA cyclised product with purity of approximately 80% (Scheme 1 and Fig. 2); thus, they were used directly in the next DA reaction.

### 2.2. Synthesis of decalin diastereomers

To analyse the stereoselectivity of the IMDA reaction, the reaction was monitored using UPLC (Fig. 2). Authentic samples of all diastereomeric decalins for peak assignment using UPLC were synthesised by thermal or enzymatic scale-up IMDA reactions and isolated by high-performance liquid chromatography (HPLC) for stereochemistry determination. The *trans*-decalins 1Aa, 1Ab and 2Aa, 2Ab were isolated from the products obtained by Lacey–Dieckman cyclisation and subsequent thermal IMDA reaction from the corresponding serine amides 15a and 15b. A diastereomer 2Ca was obtained in a two-step isolation yield of 11% by basic treatment of 15b to form tetramic acid 16b, immediately followed by incubation with 1 μM Fsa2 at 25 °C. Comparing the electronic circular dichroism (ECD) spectrum of 2Ca with each of 1Aa and 1Ab, we found that the 5′ position of the tetramic acid side chain is in the *S* configuration because the peak shape of 2Ca is very similar to that of 1Aa (Fig. S3†). Furthermore, as the C-5′ epimers of *cis*-decalins 1B and 2B were difficult to separate, they were converted to diastereomeric acetals 17a and 17b and 18a and 18b using the chiral resolving agent (*S*)-ALBO, which were successfully separated by HPLC, respectively. These were deprotected with acid to yield two pairs of C-5′ epimers 1Ba and 1Bb and 2Ba and 2Bb (Scheme S5†). Thus, nine decalin diastereomers were isolated and purified, which were analysed by UPLC and found to be 91–100% pure (Fig. S7†). Their stereochemistry was determined by Two-dimensional nuclear magnetic resonance (2D NMR) spectroscopy (HH-COSY, HMBC, NOESY) with reference to the spectra of the known compounds, equisetin(1Aa)^8^ and *cis*-decaline 1Ba (ref. 8) (Tables S1–S8†). The absolute configuration of all chiral centres, including the C-5′ of the tetramic acid moiety, was estimated through ECD spectroscopy^11,19^ (Fig. S1 and S2†). Interestingly, there were significant differences in the peak shape around 230 nm depending on the absolute configuration of C-5′ of the tetramic acid. These spectral datasets will be useful in determining the stereochemistry of natural tetramic acid-bearing decalins, which has been difficult in the past.

### 2.3. Analysis of stereoselectivity of enzymatic or thermal IMDA cycloadditions

The precursors (16a and 16b) of the IMDA reaction were subjected to enzymatic or thermal IMDA conditions and the products were analysed by UPLC over time (Fig. 2a and b). Polyene 16a was incubated in enzyme reaction buffer (20 mM Tris–HCl, 10 mM NaCl, 10 mM EDTA, pH 7.5) at 25 °C for 24 h to investigate its stability. The results showed that the ratio of the cyclised products remained almost the same as at the start of the reaction, suggesting that IMDA cyclisation does not proceed under the ambient condition in the absence of the enzymes (Fig. 2c and d). Separately, 1Aa, one of the decalin diastereomers produced, was confirmed to be stable without isomerization under either enzyme buffer or thermal reaction conditions (Fig. S10†). Treatment of precursor 16a with 0.5 μM Fsa2 at 25 °C for 60 min produced a ratio of 60.4% of equisetin (1Aa) and 16.9% of C-5′ epimer 1Ab, exhibiting that *trans-*selective cycloaddition occurs as in biosynthesis (Fig. 2a and c). Interestingly, the formation of 1Aa was almost completed within 10 min, whereas the C-5′ epimer, 1Ab, was gradually formed until 60 min later (Fig. S8ii†). When 16a was treated with 0.5 μM Phm7, the reaction barely proceeded; thus, a 10-fold higher dose of 5.0 μM was used, resulting in a 64.1% ratio of 1Aa after 24 h. Phm7 exhibits phomasetin-type stereoselectivity (2*R*, 3*S*, 6*R*, 11*S*) in biosynthesis; however, when 16a (pro-equisetin), the reverse configuration of its natural substrate, is used as a substrate, it is found to exhibits equisetin-type stereoselectivity (2*S,* 3*R*, 6*S*, 11*R*), although the reaction is slow (Fig. 2a, c and S8iii†). On the other hand, the reaction of 16b (*epi*-pro-equisetin) with 0.5 μM Fsa2 for 60 min selectively yielded a new diastereomer, 8-*epi*-equisetin (2Ca) (Fig. 2b and d). Treatment of 16b with 0.5 μM Phm7 yielded 2Aa as the main product with a 67.6% formation ratio. The reaction yielding 2Aa was completed within 20 min, whereas the formation of 2Ab (*ent*-equisetin)^20^ was slower with a formation ratio of 14.7% at 60 min. Note that the unnatural 5′*S*-epimer, rather than a precursor with the same 5′*R* configuration as the biosynthetic substrate of Phm7, reacted predominantly to produce 2Aa (Fig. S9iii†).

To summarise the stereoselectivity of the enzymatic IMDA reaction, both Fsa2 and Phm7 exhibited the same equisetin-type stereoselectivity for 16a (6*R*, pro-equisetin). In contrast, when 16b (6*S*, *epi*-pro-equisetin) was used as substrate, cyclisation proceeded with Fsa2- and Phm7-specific stereoselectivity as in biosynthesis to produce 2Ca (8-*epi*-equisetin) and 2Aa, respectively. These data are consistent with the reaction selectivity of Fsa2 with pro-phomasetin in the phomasetin-producing fungus.^9^ These results show that Fsa2 can takes both enantiomeric polyene precursors as substrates regardless of the stereochemistry of 6-methyl group (but is less reactive with the 6*S* precursor), yielding an equisetin-type [2*S*, 3*R*, 6*S*, 11*R*]-diastereomer with stereoselectivity *via* “Fsa2 type” folding. On the other hand, Phm7 recognises the 6-methyl configuration of the substrate and produced a phomasetin-type [2*R*, 3*S*, 6*R*, 11*S*]-diastereomer from the 6*S* precursor (16b) and an equisetin-type [2*S*, 3*R*, 6*S*, 11*R*]-diastereomer from the 6*R* precursor (16a).

The steady-state kinetic parameters of Fsa2 and Phm7 were determined using 16a and 16b synthesised and purified as described above. Since the maximum absorption wavelength of the precursor polyenes is 267 nm, corresponding to the absorption minimum in the spectra of the product decalins, the initial velocities of all IMDA reactions were determined from the change in absorbance at 267 nm (Fig. S11–S13†). The results showed that Fsa2 had the highest *k*_cat_ value for biosynthetic substrate 16a and significantly higher catalytic efficiency than that of Phm7 using 16b (*k*_cat_/*K*_m_: 2.80 × 10^2^ and 3.11 μM^−1^ min^−1^, respectively) (Fig. S14 and Table S11†). To be precise, 16b differs from pro-phomasetin, the natural substrate of Phm7, by having one short conjugated group and an *N*-methylated tetrameric acid, but the extent to which this difference affects the efficiency of Phm7 catalysis is currently unknown. In combination with unnatural substrates, Fsa2 reacted efficiently with 16b (*k*_cat_/*K*_m_ = 2.41 μM^−1^ min^−1^), whereas the catalytic efficiency of the reaction of 16a with Phm7 was very low and showed unexpected stereoselectivity (*k*_cat_/*K*_m_ = 2.36 × 10^−2^ μM^−1^ min^−1^). See also the docking study below.

When 16a or 16b was subjected to the thermal IMDA reaction by refluxing in toluene for 60 min, *cis*-decalin 1B and 2B were produced as the main products, respectively, and some *trans*-decalin (1Aa and 1Ab or 2Aa and 2Ab) was also formed (*cis*-selectivity of 77% for 1B and 73% for 2B).

### 2.4. Elucidation of stereocontrol mechanisms of thermal or enzymatic IMDA reactions by DFT calculations

To explore the reaction mechanism and stereocontrol of the IMDA [4 + 2] cycloaddition of a pair of tetramic acid-bearing polyene enantiomers 16a and 16b, DFT calculations were performed at the M06-2X/6-311++G** (scrf = CPCM, water) level. The initial structures were constructed using the calculated data^9^ from the IMDA reaction of the methyl thioester model substrate. The conformational free energies were calculated at the M06-2X/6-311++G** level for the three conformations with different hydrogen bonds in the tetrameric acid moiety, and the results showed that III was more stable than I and II by 14 and 18 kJ mol^−1^, respectively; thus, the tetramic acid moiety in the initial structure was determined to be conformer III (Scheme S6†).^21^

**Fig. 3:**
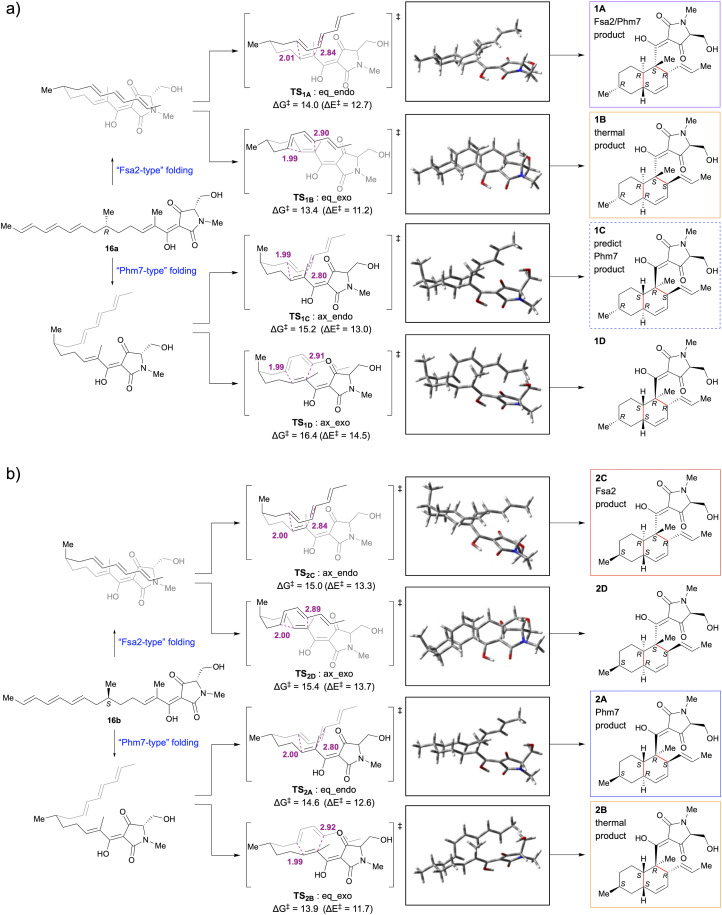
DFT calculations for competing IMDA reaction pathway to produce four decalin stereoisomers. IMDA reaction of (a) 6*R* precursor (16a) (b) 6*S* precursor (16b) in water. Structural and geometrical changes during IMDA reaction and activation free energy (Δ*G*^‡^ and Δ*E*^‡^) were calculated using M06-2X/6-311++G** (scrf = CPCM, water). All free energies are given in kcal mol^−1^, and distances are shown in Å.

DFT calculations yielded four transition state structures and associated activation barriers (Δ*G*^‡^ and Δ*E*^‡^) for the formation of the decalin ring from each of the 6-*epi*-polyene precursors 16a and 16b in water (Fig. 3). They suggest that the two factors that determine the activation free energy of the transition state in the IMDA reaction are the *endo-* or *exo-*addition and the configuration of the 6-methyl group of the substrate polyene. Transition states with the 6-methyl group in the equatorial position have lower activation free energies than those in the axial position. The transition states requiring the lowest and next lowest activation barriers for the uncatalysed IMDA reactions of 16a and 16b are TS_1B_:eq_exo (Δ*G*^‡^ = 13.4 kcal mol^−1^) < TS_1A_:eq_endo (Δ*G*^‡^ = 14.0 kcal mol^−1^) and TS_2B_:eq_exo (Δ*G*^‡^ = 13.9 kcal mol^−1^) < TS_2A_:eq_endo (Δ*G*^‡^ = 14.6 kcal mol^−1^), respectively, and the order of decalin stereoisomers expected to be formed is 1B > 1A from 16a and 2B > 2A from 16b. This was consistent with the experimental results showing *cis***-**decalin selectivity in the thermal IMDA reaction (Fig. 2).

Fujiyama *et al.* performed MD simulations of the reaction of pro-phomasetin with Phm7 and DFT calculations of the uncatalysed IMDA reaction of pro-phomasetin in water and showed that the geometry between the dienophile and diene moieties in the major folded structure in the pocket of Phm7 is close to the conformation of transition state that yields phomasetin.^22^ In the reported models of Fsa2 and Phm7 binding with their respective substrates, the diene and dienophile moieties were arranged to form *endo*-transition states in both enzymes. Their folded shapes are close to the conformation of the lower energy transition states, TS_1A_:eq_endo and TS_2A_:eq_endo, which generate 1A and 2A, respectively and are mirror images of each other.^22^ This is supported by experimental data showing that 1A and 2A are selectively obtained from the reactions of 16a with Fsa2 and 16b with Phm7, respectively. Thus, it appears that the absolute configuration of the 6-methyl group is recognised in the chiral reaction field in the enzyme pocket and the folding orientation is regulated to favour *endo-*addition.

Then, what happens when each enzyme reacts with a substrate that has an absolute configuration opposite to that of the biosynthetic substrate? Fsa2 cyclised the 6*S* precursor (16b) with the same stereoselectivity (2*S*, 3*R*, 6*S*, 11*R*) as the biosynthetic substrate 16a to yield 2C (8-*epi*-equisetin). In the transition state, the 6-methyl group would assume an axial configuration (TS_2C_:ax_endo), which was possible because of the relatively wide binding pocket of Fsa2.^22^ When the 6*R* precursor (16a) was reacted with Phm7, contrary to expectations, cycloaddition occurred with Fsa2-type stereoselectivity, yielding 1A. Docking simulations of Phm7 (PDB ID: 7E5V)-16a to investigate this mechanism showed that 16a can fit into the binding pocket of Phm7 in a “Fsa2-type” folded pose suggested by previous molecular dynamics (MD) simulation^22,23^ (Fig. S15†). Comparing the pose of 16b in the binding pocket of Phm7 with the best docked pose of 16a (Fig. S15a and b†), both are similar in the *endo*-type-folded structure with the 6-methyl group in the equatorial position, only the tetramic acid plane is inverted. This means that 16b and 16a are in opposite turn conformations in the same binding pocket. The reason for this unusual stereoselectivity may be that the relatively narrow binding pocket of Phm7, as shown in the crystal structure analysis,^22,23^ does not allow the 6-methyl group to be placed in the axial configuration. Further MD simulations and crystallographic analysis studies are required to prove this interesting result.

### 2.5. Antibacterial evaluation

Finally, the various diastereomers of tetramic acid-bearing decalins isolated in this study were evaluated for antibacterial activity against methicillin-sensitive and -resistant *Staphylococcus aureus* (MSSA and MRSA, respectively) (Table 1). Equisetin (1Aa) exhibited significant antibacterial activity against MRSA and MSSA, as previously reported.^24,25^ Interestingly, 2Ab (*ent*-equisetin) exhibited better activity against MRSA than 1Aa. Phomasetin, which has the same stereochemistry as 2Ab, exhibited antibacterial activity^26^ but its activity against MRSA is unknown. The loss of activity in the C-5′ epimers of both compounds, 1Ab and 2Aa, suggests that the configuration at the 5′ position of tetramic acid is critical for antibacterial activity, as previously reported.^27^ The activity of 8-*epi*-equisetin (2Ca) was also reduced, suggesting that the stereochemistry of the 8-methyl group on the decalin skeleton is also important for its antibacterial activity. In contrast to *trans*-decalins, neither *cis*-decalins 1B nor 2B exhibited any antibacterial activity. Although various natural products having a tetramic acid-bearing decalin scaffold have been evaluated for antimicrobial and other biological activities,^6^ there is no precedent for comparing the antibacterial activity of diastereomers of decalins with the same substituents. Therefore, we believe that this structure–activity relationship study focusing on stereochemistry has provided useful information for the molecular design of antimicrobial agents.

**Table 1:** Antibacterial activity of equisetin diastereomers against MSSA or MRSA

	MIC^a^ [μg mL^−1^]			
Compound	*S*. *aureus* (NBRC12732)	*S. aureus* (clinical strain)	MRSA (clinical strain)	MRSA (clinical strain)
1Aa	32	16	16	16
1Ab	>64	64	64	>64
1Bb	>64	64	64	>64
2Aa	64	>64	>64	>64
2Ab	16	16	8	16
2Ba	64	64	64	64
2Bb	>64	>64	>64	>64
2Ca	64	64	32	64
Oxacillin^b^	1	<0.5	>16	>16

a MIC, minimal inhibitory concentration. Determined by the broth microdilution method.

b Culture medium contained 2% NaCl.

## 3. Conclusions

In this study, we focused on the role of the configuration of 6-methyl group of the substrate polyene in the stereocontrol of the IMDA cycloaddition catalysed by Fsa2 and Phm7. Pro-equisetin (16a), the substrate of Fsa2, and its C-6 epimer (16b) were synthesised and subjected to thermal and enzymatic IMDA reactions, and all the nine decalin diastereomers produced were successfully isolated and purified. Then, their absolute configuration was determined by various 2D NMR, ECD, and other spectral analyses. We also performed DFT calculations for the IMDA reactions of 16a and 16b to investigate the mechanism of enzymatic and thermal IMDA stereoselectivity.

Fsa2 and Phm7 are a unique pair of DAases that catalyse the IMDA reaction with opposite stereoselectivity in the biosynthesis of equisetin and phomasetin, tetramic acid-bearing decalin natural products. They use polyene analogues as substrates, each of which has a different absolute configuration of the 6-methyl group. We investigated the mechanism of their stereocontrol in the IMDA cycloaddition by reacting these enzymes with biosynthetic substrate and its enantiomer. As a result, Fsa2 cyclised both enantiomeric polyenes with the same equisetin-type stereoselectivity regardless of the configuration of the 6-methyl group. On the other hand, Phm7 folded the substrate in opposite orientations depending on the configuration of the 6-methyl group to construct equisetin- and phomasetin-type decalins. Although the mechanisms for these results are unclear, the difference in stereoselectivity of Fsa2 and Phm7 toward their antipodal substrates is a noteworthy finding because it highlights the differences in the internal structure of the binding pocket.

Finally, we evaluated the antimicrobial activity of the diastereomers isolated in this study and investigated the structure–activity relationship focusing on stereochemistry. The results revealed that the *trans* conformation and stereochemistry of the 5′ position of tetramic acid and the 8 position of decalin are critical for antimicrobial activity.

As described above, this study provided unique insights into the stereoselectivity of enzymes catalysing the IMDA reactions, which plays a crucial role in the synthesis of natural product skeletons. It will also serve as a useful guide for future enzyme engineering studies of pericyclases.

## 4. Experimental

### 4.1. General procedures

All commercially available reagents and solvents were used without further purification. Normal-phase thin layer chromatography (TLC) was carried out on TLC Silica gel 60 F_254_ (Merck, 1.05715.0001) using reagent grade solvents. TLC was detected by the absorption of UV light (254 nm) or using visualization reagents (*p*-anisaldehyde). *R*_f_ values were obtained under the development conditions used for the separation and analysis of diastereomers by TLC. Preparative TLC was performed by PLC Silica gel 60 F_254_ (Merck, 1.05744.0001) with mixed solvents as described. ^1^H-NMR spectra were obtained at ambient temperature on JEOL ECA-500 spectrometer at 500 MHz, and JEOL ECA-600 spectrometer or Bruker Biospin Avance III 600 spectrometer at 600 MHz in CDCl_3_ with tetramethylsilane (TMS) as an internal standard. ^13^C-NMR spectra were obtained on JEOL ECA-500 spectrometer at 125 MHz and Bruker Biospin Avance III 600 spectrometer at 150 MHz in CDCl_3_. Chemical shifts of ^13^C-NMR are referenced to CDCl_3_ (77.16 ppm). Splitting patterns are designated as follows: s, singlet; d, doublet; dd, doublet of doublets; m, multiplet; br, broad. Electrospray ionization (ESI) mass spectra were carried out on Acquity RDa (Waters) mass spectrometer. Optical rotations were measured using the P-1020 (JASCO) apparatus. Absorbance measurements were performed on Agilent 8453 UV-VIS spectrophotometer (Agilent). Reversed phase ultra performance liquid chromatography (UPLC) analyses were performed by Acquity UPLC H-Class (Waters). Reversed phase high performance liquid chromatography (HPLC) purifications were performed by PU-4086-Binary Pump (JASCO) and UV-970 (JASCO) with TSKgel ODS-80Ts column (Tosoh Bioscience, 5 μm, 20.0 × 250 mm, flow rate 8.0 mL min^−1^). Ultra-pure water (solvent A) and MeCN (solvent B) containing 0.05% (v/v) formic acid were used as a solvent system, and the eluting products were detected by UV at 254 nm, 290 nm or 360 nm.

### 4.2. Synthesis of decalin diastereomers

#### 4.2.1 General procedure for thermal IMDA reactions

All to a solution of compound 15 (24.7 mM) in ^*t*^BuOH was added ^*t*^BuOK (2.0 equiv.) and the mixture was stirred at room temperature for 10 min. The reaction mixture was quenched with 1 M HCl aq. and extracted with AcOEt to afford 16 as a yellow oil. It was dissolved in toluene (final concentration = 10 mM) and the solution was refluxed for 1.0 h. The solution was concentrated *in vacuo* and the resulting residue was purified by preparative TLC.

For time-course experiment, the toluene solution of 16 was refluxed for 10 min, 20 min or 60 min, respectively. The reactions were terminated by concentration *in vacuo*. The residues were dissolved with EtOH (24.7 mM), diluted 100-fold with Tris–HCl buffer, poured into a two-fold volume of cold EtOH, and stored at −78 °C until UPLC analysis. Thawed samples were analysed by UPLC using UPLC-MS. The diastereomer production ratio was determined as the ratio of the peak area of each diastereomer to the sum of the peak areas of all diastereomers in the chromatogram obtained.

Conditions: column, Acquity UPLC BEH C18 (Waters, 1.7 μm, 2.1 × 100 mm); flow rate, 0.25 μL min^−1^; mobile phase, solvent A/B = 100/0 for 0.5 min, a linear gradient from 100/0 to 40/60 over 2.0 min and 40/60 to 0/100 over 6.0 min, and 0/100 for 3.0 min.

#### 4.2.2 Synthesis of diastereomeric decalins by thermal IMDA reaction

A toluene solution of 16a was prepared from 15a (380 mg, 0.94 mmol) according to the general procedure described above and refluxed for 60 min. The solution was concentrated, and the resulting residue was applied to preparative TLC and eluted three times repeatedly with petroleum ether/AcOEt (1 : 4, 0.1% acetic acid), followed by preparative HPLC with A/B (gradient 2 : 3 to 1 : 19, over 40 min) to afford 1Aa, 1Ab and 1B (1Aa; 5.0 mg, 1% over 2 steps, 1Ab; 4.0 mg, 1% over 2 steps, 1B; 121 mg, crude).

Similarly, the procedure described above was performed with compound 15b (200 mg, 0.49 mmol) to yield decalins 2Aa, 2Ab, and 2B (2Aa; 1.0 mg, 1% over 2 steps, 2Ab; 1.0 mg, 1% over 2 steps, 2B; 30 mg, crude).

Crude 1B and 2B were converted to diastereomeric acetals using the chiral resolving agent (*S*)-ALBO, followed by separation by preparative HPLC and subsequent deprotection to afford 1Ba and 1Bb and 2Ba and 2Bb, respectively (see Scheme S5 and Experimental procedures in ESI†).

##### 4.2.2.1 (3*Z*,5*S*)-3-{[(1*S*,2*R*,4a*S*,6*R*,8a*R*)-1,6-Dimethyl-2-[(*E*)-prop-1-enyl]-4a,5,6,7,8,8a-hexahydro-2*H*-naphthalen-1-yl]-hydroxymethylidene}-5-(hydroxymethyl)-1-methylpyrrolidine-2,4-dione (**1Aa**; equisetin)^14,15^ [CAS 1417403-52-1]

*R*_f_ = 0.28 (petroleum ether : AcOEt = 2 : 8, 3 times, 0.1% acetic acid); red oil; ^1^H-NMR (500 MHz, CDCl_3_) *δ* = 5.43–5.39 (m, 2H), 5.31–5.24 (m, 1H), 5.22–5.18 (m, 1H), 4.03 (brd, *J* = 8.0 Hz, 1H), 3.88 (brd, *J* = 8.0 Hz, 1H), 3.64–3.63 (m, 1H), 3.35 (brs, 1H), 3.06 (s, 3H), 1.97 (m, 1H), 1.87–1.80 (m, 2H), 1.77–1.74 (m, 1H), 1.68–1.66 (m, 1H), 1.56–1.46 (m, 1H), 1.55 (brd, *J* = 4.0 Hz, 3H), 1.46 (brs, 3H), 1.15–1.01 (m, 2H), 0.92 (d, *J* = 6.5 Hz, 3H), 0.90–0.82 (m, 1H); ^13^C-NMR (125 MHz, CDCl_3_) *δ* = 199.2, 190.7, 177.3, 131.0, 130.2, 127.2, 126.7, 100.0, 66.8, 60.6, 48.9, 45.1, 42.5, 40.0, 38.7, 35.8, 33.6, 28.4, 27.5, 22.6, 18.1, 14.1; [*α*]D24 −231.1° (*c* 0.13, CHCl_3_); UV *λ*_max_(MeOH)/nm 245 (log *ε* 3.73), 292 (3.79); HRMS (ESI^+^) *m*/*z* calcd for C_22_H_31_NO_4_Na [M + Na]^+^ 396.2145, found 396.2155.

##### 4.2.2.2 (3*Z*,5*R*)-3-{[(1*S*,2*R*,4a*S*,6*R*,8a*R*)-1,6-Dimethyl-2-[(*E*)-prop-1-enyl]-4a,5,6,7,8,8a-hexahydro-2*H*-naphthalen-1-yl]-hydroxymethylidene}-5-(hydroxymethyl)-1-methylpyrrolidine-2,4-dione (**1Ab**; 5′-*epi*-equisetin)^14^ [CAS 123406-60-0]

*R*_f_ = 0.75 (petroleum ether : AcOEt = 1 : 4, 3 times, 0.1% acetic acid); red oil; ^1^H-NMR (500 MHz, CDCl_3_) *δ* = 5.40–5.38 (m, 2H), 5.29–5.22 (m, 1H), 5.18–5.15 (m, 1H), 4.06 (brd, *J* = 11.5 Hz, 1H), 3.84 (dd, *J* = 5.0, 11.5 Hz, 1H), 3.67 (brs, 1H), 3.36 (brs, 1H), 3.05 (s, 3H), 2.01–1.96 (m, 1H), 1.87–1.75 (m, 3H), 1.72–1.65 (m, 1H), 1.53 (d, *J* = 5.7 Hz, 3H), 1.49–1.47 (m, 1H), 1.47 (brs, 1H), 1.11 (brdd, *J* = 12.0, 2.3 Hz, 1H), 1.09–1.02 (m, 1H), 0.92 (d, *J* = 6.3 Hz, 3H), 0.89–0.87 (m, 1H); ^13^C-NMR (125 MHz, CDCl_3_) *δ* = 199.2, 191.0, 177.2, 131.0, 130.1, 127.2, 126.8, 100.4, 66.3, 60.4, 48.9, 45.1, 42.4, 40.1, 38.7, 35.8, 33.7, 28.5, 27.3, 22.6, 18.1, 14.2; [*α*]D24 −106.3° (*c* 0.11, CHCl_3_); UV *λ*_max_(MeOH)/nm 244 (log *ε* 3.72), 293 (3.74); HRMS (ESI^+^) *m*/*z* calcd for C_22_H_31_NO_4_Na [M + Na]^+^ 396.2145, found 396.2144.

##### 4.2.2.3 (3*Z*,5*S*)-3-{[(1*S*,2*S*,4a*R*,6*R*,8a*R*)-1,6-Dimethyl-2-[(*E*)-prop-1-enyl]-4a,5,6,7,8,8a-hexahydro-2*H*-naphthalen-1-yl]-hydroxymethylidene}-5-(hydroxymethyl)-1-methylpyrrolidine-2,4-dione (**1Ba**)^22^ [CAS 2095311-86-5]

*R*_f_ = 0.64 (petroleum ether : AcOEt = 1 : 4, 0.1% acetic acid, 3 times); red oil; ^1^H-NMR (600 MHz, CDCl_3_) *δ* = 5.63–5.61 (m, 1H), 5.50–5.43 (m, 2H), 5.42–5.38 (m, 1H), 4.07 (brd, *J* = 7.5 Hz, 1H), 3.89 (brd, *J* = 7.5 Hz, 1H), 3.69 (brs, 1H), 3.35 (brs, 1H), 3.07 (s, 3H), 2.52 (brd, *J* = 9.8 Hz, 1H), 2.18–2.15 (m, 1H), 1.70–1.65 (m, 2H), 1.69 (d, *J* = 7.2 Hz, 3H), 1.61–1.59 (m, 1H), 1.44 (dd, *J* = 23.0, 13.9 Hz, 1H), 1.35–1.32 (m, 1H), 1.33 (brs, 3H), 1.08–1.04 (m, 1H), 0.93–0.87 (m, 1H), 0.82 (d, *J* = 6.4 Hz, 3H); ^13^C-NMR (150 MHz, CDCl_3_) *δ* = 201.7, 190.5, 177.8, 131.5, 130.4, 129.9, 127.8, 97.8, 66.6, 60.3, 49.5, 42.5, 40.7, 37.9, 35.3, 34.7, 28.4, 27.5, 22.9, 22.7, 18.6, 18.2; [*α*]D24 +85.6° (*c* 0.11, CHCl_3_); UV *λ*_max_(MeOH)/nm 250 (log *ε* 3.84), 291 (3.94); HRMS (ESI^+^) *m*/*z* calcd for C_22_H_32_NO_4_ [M + H]^+^ 374.2326, found 374.2327.

##### 4.2.2.4 (3*Z*,5*R*)-3-{[(1*S*,2*S*,4a*R*,6*R*,8a*R*)-1,6-Dimethyl-2-[(*E*)-prop-1-enyl]-4a,5,6,7,8,8a-hexahydro-2*H*-naphthalen-1-yl]-hydroxymethylidene}-5-(hydroxymethyl)-1-methylpyrrolidine-2,4-dione (**1Bb**)

*R*_f_ = 0.64 (petroleum ether : AcOEt = 1 : 4, 0.1% acetic acid, 3 times); red oil; ^1^H-NMR (600 MHz, CDCl_3_) *δ* = 5.62 (brd, *J* = 9.6 Hz, 1H), 5.45–5.43 (m, 2H), 5.39 (brd, *J* = 9.6 Hz, 1H), 4.05 (brd, *J* = 8.4 Hz, 1H), 3.88 (brd, *J* = 8.4 Hz, 1H), 3.70 (brs, 1H), 3.35 (brs, 1H), 3.07 (s, 3H), 2.57 (brd, *J* = 4.9 Hz, 1H), 2.16 (brs, 1H), 1.68–1.66 (m, 2H), 1.68 (d, J = 3.6 Hz, 3H), 1.60 (brd, *J* = 12.4 Hz, 1H), 1.44 (dd, *J* = 23.7, 12.0 Hz, 1H), 1.34–1.31 (m, 1H), 1.31 (brs, 3H), 1.12–1.09 (m, 1H), 0.90 (dd, *J* = 23.5, 12.0 Hz, 1H), 0.82 (d, *J* = 6.0 Hz, 3H); ^13^C-NMR (150 MHz, CDCl_3_) *δ* = 202.0, 190.4, 177.8, 131.5, 130.5, 129.8, 127.8, 97.6, 66.7, 60.3, 49.6, 42.4, 40.7, 37.9, 35.4, 34.8, 28.4, 27.6, 22.9, 22.7, 18.5, 18.2; [*α*]D24 +64.6° (*c* 0.07, CHCl_3_); UV *λ*_max_(MeOH)/nm 250 (log *ε* 3.76), 290 (3.87); HRMS (ESI^+^) *m*/*z* calcd for C_22_H_31_NO_4_Na [M + Na]^+^ 396.2145, found 396.2158.

##### 4.2.2.5 (3*Z*,5*S*)-3-{[(1*R*,2*S*,4a*R*,6*S*,8a*S*)-1,6-Dimethyl-2-[(*E*)-prop-1-enyl]-4a,5,6,7,8,8a-hexahydro-2*H*-naphthalen-1-yl]-hydroxymethylidene}-5-(hydroxymethyl)-1-methylpyrrolidine-2,4-dione (**2Aa**) [CAS: 1212527-32-6]

*R*_f_ = 0.66 (petroleum ether : AcOEt = 1 : 4, 3 times, 0.1% acetic acid); red oil; ^1^H-NMR (500 MHz, CDCl_3_) *δ* = 5.42–5.38 (m, 2H), 5.27–5.22 (m, 1H), 5.19–5.14 (m, 1H), 4.06 (brd, *J* = 11.5 Hz, 1H), 3.84 (dd, *J* = 11.5, 4.6 Hz, 1H), 3.67 (brs, 1H), 3.35 (brs, 1H), 3.05 (s, 3H), 1.99–1.97 (m, 1H), 1.87–1.80 (m, 2H), 1.78–1.75 (m, 1H), 1.70–1.66 (m, 1H), 1.53–1.50 (m, 1H) 1.53 (d, *J* = 6.0 Hz, 3H), 1.47 (brs, 3H), 1.16–1.11 (m, 1H), 1.09–1.02 (m, 1H), 0.92 (d, *J* = 6.0 Hz, 3H), 0.89–0.81 (m, 1H); ^13^C-NMR (125 MHz, CDCl_3_) *δ* = 199.1, 190.9, 177.2, 131.0, 130.1, 127.2, 126.8, 100.4, 66.3, 60.4, 48.9, 45.1, 42.4, 40.1, 38.7, 35.8, 33.7, 28.5, 27.3, 22.6, 18.0, 14.2; [*α*]D24 +230.2° (*c* 0.12, CHCl_3_); UV *λ*_max_(MeOH)/nm 250 (log *ε* 3.72), 292 (3.84); HRMS (ESI^+^) *m*/*z* calcd for C_22_H_31_NO_4_Na [M + Na]^+^ 396.2145, found 396.2146.

##### 4.2.2.6 (3*Z*,5*R*)-3-{[(1*R*,2*S*,4a*R*,6*S*,8a*S*)-1,6-Dimethyl-2-[(*E*)-prop-1-enyl]-4a,5,6,7,8,8a-hexahydro-2*H*-naphthalen-1-yl]-hydroxymethylidene}-5-(hydroxymethyl)-1-methylpyrrolidine-2,4-dione (**2Ab**) [CAS 1417403-55-4]

*R*_f_ = 0.20 (petroleum ether : AcOEt = 1 : 4, 3 times, 0.1% acetic acid); red oil; ^1^H-NMR (600 MHz, CDCl_3_) *δ* = 5.43–5.39 (m, 2H), 5.28–5.24 (m, 1H), 5.19–5.10 (m, 1H), 4.03 (dd, *J* = 11.4, 2.4 Hz, 1H), 3.89 (brd, *J* = 11.4 Hz, 1H), 3.64 (brs, 1H), 3.34 (brs, 1H), 3.06 (s, 3H), 1.98–1.96 (m, 1H), 1.87–1.80 (m, 2H), 1.77–1.76 (m, 1H), 1.68–1.66 (m, 1H), 1.55–1.46 (m, 1H), 1.55 (brs, 3H), 1.46 (brs, 3H) 1.15–1.09 (dd, J = 24.0, 12.0 Hz, 1H), 1.08–1.02 (dd, J = 24.0, 12.0 Hz, 1H), 0.92 (d, *J* = 6.0 Hz, 3H), 0.89–0.82 (m, 1H); ^13^C-NMR (125 MHz, CDCl_3_) *δ* = 199.2, 190.7, 177.3, 131.0, 130.1, 127.2, 126.7, 100.1, 66.8, 60.6, 48.8, 45.1, 42.4, 40.0, 38.7, 35.8, 33.6, 27.5, 22.6, 18.1, 14.1; [*α*]D24 +241.5° (*c* 0.08, CHCl_3_); UV *λ*_max_(MeOH)/nm 249 (log *ε* 3.57), 293 (3.71); HRMS (ESI^+^) *m*/*z* calcd for C_22_H_31_NO_4_Na [M + Na]^+^ 396.2145, found 396.2153.

##### 4.2.2.7 (3*Z*,5*S*)-3-{[(1*R*,2*R*,4a*S*,6*S*,8a*S*)-1,6-Dimethyl-2-[(*E*)-prop-1-enyl]-4a,5,6,7,8,8a-hexahydro-2*H*-naphthalen-1-yl]-hydroxymethylidene}-5-(hydroxymethyl)-1-methylpyrrolidine-2,4-dione (**2Ba**)

*R*_f_ = 0.63 (petroleum ether : AcOEt = 1 : 4, 0.1% acetic acid, 3 times); red oil; ^1^H-NMR (500 MHz, CDCl_3_) *δ* = 5.62 (m, 1H), 5.50–5.42 (m, 2H), 5.39 (brd, *J* = 9.5 Hz, 1H), 4.04 (dd, *J* = 11.0, 3.0 Hz, 1H), 3.88 (dd, *J* = 11.0, 4.5 Hz, 1H), 3.70 (brs, 1H), 3.36 (brs, 1H), 3.07 (s, 3H), 2.58 (brd, *J* = 9.5 Hz, 1H), 2.16 (brs, 1H), 1.68–1.65 (m, 2H), 1.68 (d, *J* = 5.5 Hz, 3H), 1.60 (brd, *J* = 12.5 Hz, 1H), 1.44 (ddd, *J* = 25.3, 13.0, 3.3 Hz, 1H), 1.37–1.31 (m, 1H), 1.31 (brs, 3H), 1.11 (ddd, *J* = 12.5, 12.5, 4.5 Hz, 1H), 0.93–0.86 (m, 1H), 0.82 (d, *J* = 6.0 Hz, 3H); ^13^C-NMR (125 MHz, CDCl_3_) *δ* = 202.0, 190.4, 178.0, 131.6, 130.6, 129.7, 127.8, 97.7, 66.7, 60.5, 49.6, 42.4, 40.7, 38.0, 35.4, 34.9, 28.4, 27.6, 23.0, 22.7, 18.5, 18.2; [*α*]D24 −176.1° (*c* 0.13, CHCl_3_); UV *λ*_max_(MeOH)/nm 249 (log *ε* 3.80), 290 (3.88); HRMS (ESI^+^) *m*/*z* calcd for C_22_H_31_NO_4_Na [M + Na]^+^ 396.2145, found 396.2154.

##### 4.2.2.8 (3*Z*,5*R*)-3-{[(1*R*,2*R*,4a*S*,6*S*,8a*S*)-1,6-Dimethyl-2-[(*E*)-prop-1-enyl]-4a,5,6,7,8,8a-hexahydro-2*H*-naphthalen-1-yl]-hydroxymethylidene}-5-(hydroxymethyl)-1-methylpyrrolidine-2,4-dione (**2Bb**)

*R*_f_ = 0.63 (petroleum ether : AcOEt = 1 : 4, 0.1% acetic acid, 3 times); red oil; ^1^H-NMR (600 MHz, CDCl_3_) *δ* = 5.62–5.61 (m, 1H), 5.50–5.42 (m, 2H), 5.39 (brd, *J* = 9.6 Hz, 1H), 4.06 (brd, *J* = 10.2 Hz, 1H), 3.89 (brd, *J* = 10.2 Hz, 1H), 3.68 (brs, 1H), 3.36 (brs, 1H), 3.06 (s, 3H), 2.52 (brd, *J* = 11.4 Hz, 1H), 2.18 (brs, 1H), 1.68–1.64 (m, 2H), 1.68 (d, *J* = 4.2 Hz, 3H), 1.60 (brd, *J* = 13.2 Hz, 1H), 1.47–1.41 (m, 1H), 1.36–1.33 (m, 1H), 1.33 (brs, 3H), 1.11–1.03 (m, 1H), 0.93–0.87 (m, 1H), 0.82 (d, *J* = 7.2 Hz, 3H); ^13^C-NMR (125 MHz, CDCl_3_) *δ* = 201.7, 190.4, 177.9, 131.6, 130.5, 129.8, 127.7, 97.8, 66.7, 60.4, 49.6, 42.5, 40.7, 38.0, 35.4, 34.8, 28.4, 27.5, 22.9, 22.6, 18.6, 18.2; [*α*]D24 −115.4° (*c* 0.15, CHCl_3_); UV *λ*_max_(MeOH)/nm 248 (log *ε* 3.77), 290 (3.83); HRMS (ESI^+^) *m*/*z* calcd for C_22_H_31_NO_4_Na [M + Na]^+^ 396.2145, found 396.2158.

#### 4.2.3 General procedure for enzymatic IMDA reactions

Fsa2 and Phm7 were prepared as previously reported.^22^ To a ^*t*^BuOH solution of compound 15 (24.7 mM) was added ^*t*^BuOK (2.0 equiv.), and the mixture was stirred at room temperature for 10 min to prepare 16. 1 μL of this solution (final concentration = 247 μM) was added to 99 μL of Fsa2 or Phm7 in 20 mM Tris–HCl buffer (10 mM NaCl, 10 mM EDTA, pH 7.5) and incubated at 25 °C. For the time-course experiment for the reaction of 16a, 0.05 nmol of Fsa2 (final concentration = 0.5 μM) or 0.5 nmol of Phm7 (final concentration = 5.0 μM) was used and incubated for 10, 20 or 60 min or 1.0, 2.0, 4.0, 8.0 or 24 h, respectively. For reaction analysis of 16b, 0.05 nmol of Fsa2 or Phm7 (final concentration = 0.5 μM) was used and incubated for 10, 20 or 60 min. The enzyme reactions were terminated by pouring into a 2-fold volume of cold EtOH and immediately stored at −78 °C until UPLC analysis. UPLC analysis was performed according to the method described above.

#### 4.2.4 Synthesis of 2Ca by IMDA reaction using Fsa2

##### 4.2.4.1 (3*Z*,5*S*)-3-{[(1*S*,2*R*,4a*S*,6*S*,8a*R*)-1,6-Dimethyl-2-[(*E*)-prop-1-enyl]-4a,5,6,7,8,8a-hexahydro-2*H*-naphthalen-1-yl]-hydroxymethylidene}-5-(hydroxymethyl)-1-methylpyrrolidine-2,4-dione (**2Ca**)

To a solution of compound 15b (30.0 mg, 74.0 μmol) in ^*t*^BuOH (3 mL; 24.7 mM) was added ^*t*^BuOK (17 mg, 148 μmol, 2.0 equiv.). The reaction mixture was stirred at room temperature for 10 minutes and then poured into the solution of Fsa2 (6.3 mg, 0.15 μmol) in buffer (147 mL; 20 mM Tris–HCl, 10 mM NaCl, 10 mM EDTA, pH = 7.5). The solution was incubated at 25 °C for 2.5 h and extracted with CHCl_3_ (200 mL × 3). The combined organic layers were washed with brine (300 mL), dried over Na_2_SO_4_, filtered, and evaporated. The residue was purified by preparative TLC with AcOEt (3 times, 1.0% acetic acid) followed by preparative HPLC with A/B (gradient 1 : 3 to 3 : 17 over 40 min) to afford compounds 2Ca as a red oil (2Ca; 3.0 mg, 11% over 2 steps). *R*_f_ = 0.48 (AcOEt, 3 times, 1.0% acetic acid); ^1^H-NMR (600 MHz, CDCl_3_) *δ* = 5.43–5.41 (m, 1H), 5.36–5.34 (m, 1H), 5.28–5.25 (m, 1H), 5.20–5.20 (m, 1H), 4.04 (brd, *J* = 10.2 Hz, 1H), 3.88 (brd, *J* = 10.2 Hz, 1H), 3.64 (brs, 1H), 3.34 (brs, 1H), 3.06 (s, 3H), 2.07–2.03 (m, 2H), 1.76–1.69 (m, 3H), 1.62–1.41 (m, 2H), 1.59 (brd, *J* = 12.6 Hz, 1H), 1.55 (brs, 3H), 1.48 (brs, 3H), 1.23 (dd, *J* = 23.0, 11.3 Hz, 1H), 1.02 (d, *J* = 6.4 Hz, 3H); ^13^C-NMR (150 MHz, CDCl_3_) *δ* = 199.3, 190.7, 177.3, 131.1, 130.4, 127.2, 127.0, 100.1, 66.8, 60.6, 48.9, 45.0, 41.1, 39.6, 32.6, 27.9, 27.5, 23.0, 19.0, 18.1, 14.2; [*α*]D24 −289.4° (*c* 0.07, CHCl_3_); UV *λ*_max_(MeOH)/nm 245 (log *ε* 3.66), 294 (3.77); HRMS (ESI^+^) *m*/*z* calcd for C_22_H_31_NO_4_Na [M + Na]^+^ 396.2145, found 396.2153.

### 4.3. Bacterial strains

The *Staphylococcus aureus* (*S. aureus*, NBRC12732) strain as a wild type standard strain was kindly supplied from The National Institute of Technology and Evolution (Tokyo, Japan). The strain of methicillin-sensitive *S. aureus* (MSSA) and two methicillin-resistant *S. aureus* (MRSA) strains were clinical isolates.

### 4.4. Minimum inhibition concentration (MIC) assays

*S. aureus* strains stocked in a Microbank™ tube (PRO-LAB Diagnostics Ltd.) were cultivated on a nutrient agar plate at 35 °C for 24 h. One colony on the plate was inoculated and cultivated again on a nutrient agar plate at 35 °C for 24 h. Colonies on the second culture plate were suspended in Mueller Hinton broth (Eiken chemical Co., Ltd.) at a concentration of approximately 1 × 10^7^ cfu mL^−1^. The test compounds or oxacillin were dissolved in Mueller Hinton broth supplemented with or without 2% NaCl at 64 μg mL^−1^, and then diluted in 2-fold steps to 0.5 μg mL^−1^. 100 μL of each dilution was placed in a 96-well plate, and 5 μL of the bacterial suspension was added to each well. After incubation of the plate at 35 °C for 24 h, the growth of the bacteria was judged by visual observation of the turbidity of the medium and the MIC of these compounds was determined.

### 4.5. Computational details

All calculations were carried out with the Gaussian 16 (revision C.02) program package.^28^ The molecular structures and harmonic vibrational frequencies were obtained using the hybrid density functional method based on the M06-2X functional.^29,30^ The 6–311++G** basis set was used.^31^ The self-consistent reaction field (SCRF) method, based on the conductor-like polarizable continuum model (CPCM),^32–35^ was employed to evaluate the solvent reaction field (water = 78.39). Initial structures were constructed with reference to the previous report.^9^ All stationary points were optimised without any symmetry assumptions and characterized by normal coordinate analysis at the same level of theory (number of imaginary frequencies, NIMAG: 0 for minima and 1 for TSs). Gibbs free energies were calculated at 298.15 K. The intrinsic reaction coordinate (IRC) method was used to track minimum energy paths from transition structures to the corresponding local minima.^36–39^

## Author contributions

Conceptualization, H. N., S. N. and N. K.; investigation, T. K., T. U., Y. I., H. H., K. N. and T. S.; data curation, M. T., T. H. and H. N.; resources, H. H., S. N., T. S. and N. K.; formal Analysis, T. K. and T. U.; writing – original draft, T. K. and H. N.; writing – review & editing, M. T., T. H., T. U., T. S., S. N. and N. K.; funding acquisition, H. N. All authors have read and agreed to the published version of the manuscript.

## Conflicts of interest

There are no conflicts to declare.

## Supplementary Material

RA-013-D3RA04406H-s001
