# Supplementary material for: Elucidation of the stereocontrol mechanisms of the chemical and biosynthetic intramolecular Diels–Alder cycloaddition for the formation of bioactive decalins
Source: RSC Adv. 2023 Sep 19;13(40):27828–38. doi: 10.1039/d3ra04406h (PMC10508222; doi:10.1039/d3ra04406h)
Supplement: RA-013-D3RA04406H-s001 [file RA-013-D3RA04406H-s001.pdf]

Electronic Supplementary Material (ESI) for RSC Advances.
This journal is © The Royal Society of Chemistry 2023

Supporting Information

Elucidation of the stereocontrol mechanisms of the chemical and biosynthetic intramolecular Diels–Alder cycloaddition for the formation of bioactive decalins

Takumi Kariya[a], Hayato Hasegawa[b], Taro Udagawa[c], Yusaku Inada[a], Kyoko Nishiyama[d], Mieko Tsuji[a], Tasuku Hirayama[a], Tatsuo Suzutani[d], Naoki Kato[e], Shingo Nagano[f, g] and Hideko Nagasawa*[a]

[a] Laboratory of Pharmaceutical and Medicinal Chemistry, Gifu Pharmaceutical University, 1-25-4 Daigaku-nishi, Gifu, 501-1196, Japan.

**e-mail: hnagasawa@gifu-pu.ac.jp*

[b] Department of Engineering, Graduate School of Sustainability Science, Tottori University, 4-101 Koyama-cho Minami, Tottori 680-8552, Japan

[c] Department of Chemistry and Biomolecular Science, Faculty of Engineering, Gifu University, 1-1 Yanagido, Gifu 501-1193, Japan

[d] Department of Microbiology, Fukushima Medical University, 1 Hikarigaoka, Fukushima 960-1295, Japan

[e] Faculty of Agriculture, Setsunan University, 45-1 Nagaotoge-cho, Hirakata, Osaka 573-0101, Japan

[f] Department of Chemistry and Biotechnology, Graduate School of Engineering, Tottori University, 4-101 Koyama-cho Minami, Tottori 680-8552, Japan

[g] Center for Research on Green Sustainable Chemistry, Tottori University, 4-101 Koyama-cho Minami, Tottori 680-8552, Japan

List of contents

1. Experimental procedures ······ S3

1.1. General procedures ······ S3

1.2. Synthesis and characterization of compounds ······ S4

1.3. Kinetic analysis ······ S15

1.4. Molecular Docking ······ S15

2. Spectroscopic data ······ S16

Table S1. NMR data of compound **1Ab** ······ S16

Table S2. NMR data of compound **1Ba** ······ S17

Table S3. NMR data of compound **1Bb** ······ S18

Table S4. NMR data of compound **2Aa** ······ S19

Table S5. NMR data of compound **2Ab** ······ S20

Table S6. MNR data of compound **2Ba** ······ S21

Table S7. NMR data of compound **2Bb** ······ S22

Table S8. NMR data of compound **2Ca** ······ S23

Table S9. NMR data of compound **17a** and **17b** ······ S24

Table S10. NMR data of compound **18a** and **18b** ······ S25

Figure S1. Structures and ECD spectra of *trans*–decalins **1Aa**, **1Ab**, **2Aa** and **2Ab** ······ S26

Figure S2. Structures and ECD spectra of *cis*-decalins **1Ba**, **1Bb**, **2Ba** and **2Bb** ······ S27

Figure S3. Overlaid ECD spectra of **1Aa**, **1Ab** and a new diastereomer **2Ca** ······ S28

Figure S4. Structures and absorption spectra of trans–decalins **1Aa**, **1Ab**, **2Aa**, **2Ab** and **2Ca** ······ S29

Figure S5. Structures and absorption spectra of cis-decalins **1Ba**, **1Bb**, **2Ba** and **2Bb** ······ S30

3. UPLC analyses of compounds and reactions ······ S31

Figure S6. UPLC analysis of tetramic acid-bearing polyenes **16a** and **16b** ······ S31

Figure S7. UPLC analysis of the 8*S* decalins **1Aa**, **1Ab**, **1Ba**, **1Bb**, and 8*R* decalins **2Aa**, **2Ab**, **2Ba**, **2Bb** and **2Ca** ······ S32

Figure S8. Time-course of IMDA reactions of precursor **16a** ······ S34

Figure S9. Time-course of IMDA reactions of precursor **16b** ······ S35

Figure S10. UPLC analysis of thermodynamic isomerization of decalin **1Aa** ······ S36

4. Kinetic analyses of IMDA reaction catalyzed by Fsa2 and Phm7 ······ S37

Figure S11. Structures and absorption spectra of precursors **16a** and **16b** ······ S37

Figure S12. Absorption spectral change of **16a** ······ S38

Figure S13. Absorption spectral change of **16b** ······ S39

Figure S14. Kinetic parameters of Fsa2 and Phm7-catalyzed IMDA reactions ······ S40

Table S11. Enzyme kinetics of Fsa2 and Phm7 ··· S41

5. Computational study ··· S42

Scheme S6. The calculated conformational free energies of three tautomers of tetramic acid moiety ··· S42

Figure S15. Binding mode of Phm7 (PDB ID: 7E5V) and precursor **16a** ··· S43

References ··· S44

S16–S85. ^{1}H NMR, ^{13}C NMR, HH-COSY, HSQC, HMQC, HMBC, NOESY spectra ··· S45

Cartesian coordinates and energies obtained by DFT calculation ··· S115

1. Experimental procedures

1.1. General procedures

All commercially available reagents and solvents were used without further purification. Normal-phase thin layer chromatography (TLC) was carried out on TLC Silica gel 60 F_{254} (Merck, 1.05715.0001) using reagent grade solvents. TLC was detected by the absorption of UV light (254 nm) or using visualization reagents (molybdophosphoric acid or *p*-anisaldehyde). Preparative TLC was performed by PLC Silica gel 60 F_{254} (Merck, 1.05744.0001) with mixed solvents as described. Column chromatography was performed by hand using silica-gel (Taiko-shoji, AP-300S) or on a Biotage Accelerated Chromatographic Isolation System with silica-gel-packed column (Fuji silysia, Chromatorex or FL60D) with mixed solvents as described. ^{1}H-NMR spectra were obtained at ambient temperature on JEOL ECZ-400 spectrometer at 400 MHz, JEOL ECA-500 spectrometer at 500 MHz, and JEOL ECA-600 spectrometer or Bruker Biospin Avance III 600 spectrometer at 600 MHz in $CDCl_3$ or CD_3OD with tetramethylsilane (TMS) as an internal standard. ^{13}C-NMR spectra were obtained on JEOL ECA-500 spectrometer at 125 MHz, JEOL ECZ-400 spectrometer at 100 MHz, and Bruker Biospin Avance III 600 spectrometer at 150 MHz in $CDCl_3$. Chemical shifts of ^{13}C-NMR are referenced to $CDCl_3$ (77.16 ppm). Splitting patterns are designated as follows: s, singlet; d, doublet; t, triplet; dd, doublet of doublets; dt, doublet of triplets; td, triplet of doublets; tt, triplet of triplets; q, quartet; dq, doublet of quartets; tq, triplet of quartets; ddd, doublet of doublet of doublets; m, multiplet; br, broad. Electrospray ionization (ESI) mass spectra were carried out on JMS-SX102A (JEOL), LCMS-IT-TOF (Shimadzu), and Acquity RDa (Waters) mass spectrometer. Optical rotations were measured using the P-1020 (JASCO) apparatus. Absorbance measurements were performed on Agilent 8453 UV-VIS spectrophotometer (Agilent) and Duetta fluorescence and absorbance spectrometer (HORIBA). Electronic Circular dichroism (ECD) spectra were recorded at ambient temperature on J-820P (JASCO) equipped with a 1 mm path length quartz cell over a wavelength range of 200–400 nm. Data pitch was set to 0.1 nm. The scanning speed was set to 100 nm/min, and the spectra were averaged from two scans. Reversed phase ultra-performance liquid chromatography (UPLC) analyses were performed by Acquity UPLC H-Class (Waters) with Acquity UPLC BEH C18 (Waters, 1.7 μm, 2.1 × 50 mm, flow rate 0.25 μL/min). Reversed phase high performance liquid chromatography (HPLC) purifications were performed by PU-4086-Binary Pump (JASCO) and UV-970 (JASCO) with TSKgel ODS-80Ts column (Tosoh Bioscience, 5 μm, 20.0 × 250 mm, flow rate 8.0 mL/min). Ultra-pure water (solvent A) and MeCN (solvent B) containing 0.05% (v/v) formic acid were used as a solvent system, and the eluting products were detected by UV at 254 nm, 290 nm or 360 nm.

1.2. Synthesis and Characterization of Compounds

1.2.1 Synthesis and Characterization of HWE reagent 4

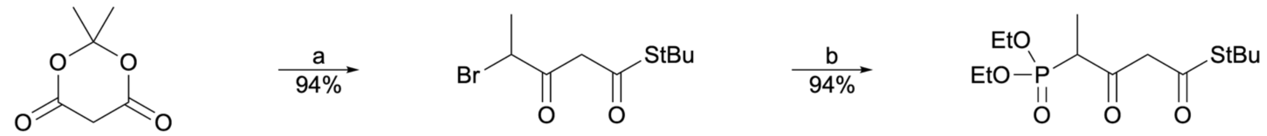

Meldrum's acid **3** **4**

***Scheme S1. Synthesis of HWE reagent 4*:** (a) (i) 2-bromopropionyl bromide, pyridine, CH_2Cl_2, 0 °C, 2.0 h; (ii) ^{t}BuSH, benzene, reflux, 2.0 h; (b) NaH, THF, −30 °C to −20 °C, 2.0 h, then $(EtO)_2PONa$, THF, −20 °C to rt, overnight.

***tert*-Butyl 4-bromo-3-oxopentanethioate (3)**[1] [CAS: 845729-78-4 (keto) / 1450742-27-4 (enol)]

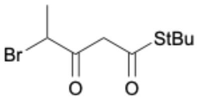

To a solution of Meldrum's acid (10.0 g, 69.4 mmol) and pyridine (11.2 mL, 139 mmol, 2.0 equiv.) in CH_2Cl_2 (104 mL) was added 2-bromopropionyl bromide (8.0 mL, 76.3 mmol, 1.1 equiv.) dropwise *via* syringe at 0 °C. The reaction mixture was stirred for 2.0 h at 0 °C and quenched with 2 M HBr aq. (350 mL). The aqueous layer was extracted with CH_2Cl_2 (200 mL × 2). The combined organic layers were washed with brine (200 mL), dried over $MgSO_4$, filtered, and concentrated to afford a brown oil.

To a solution of the residue in benzene (104 mL) was added *tert*-butylthiol (23.5 mL, 208 mmol, 3.0 equiv.), and the mixture was refluxed for 2.0 h. The solvent was evaporated, and the resulting residue was purified by column chromatography eluted with *n*-hexane / AcOEt (gradient 10 : 0 to 40 : 1) and *n*-hexane / Et_2O (40 : 1) to afford **3** as an orange oil (17.5 g, 94 % over 2 steps). Compound **3** exists as a (6.0 : 4.0) *keto* : *enol* mixture in $CDCl_3$ at room temperature. *R*f = 0.50 (*n*-hexane : Et_2O = 10 : 1); ^{1}H-NMR (500 MHz, $CDCl_3$) δ = 5.54 (s, 0.4H, *enol*), 4.60 (q, *J* = 7.0 Hz, 0.6H, *keto*), 4.37 (q, *J* = 7.0 Hz, 0.4H, *enol*), 4.02 (d, *J* = 14.9 Hz, 0.6H, *keto*), 3.75 (d, *J* = 14.9 Hz, 0.6H, *keto*), 1.83 (d, *J* = 7.0 Hz, 1.2H, *enol*), 1.76 (d, *J* = 7.0 Hz, 1.8H, *keto*), 1.52 (s, 3.6H, *enol*), 1.48 (s, 5.4H, *keto*).

***tert*-Butyl 4-diethylphosphono-3-oxopentanethioate (4)**[1] [CAS: 313072-28-5(keto) / 1450742-28-5(enol)]

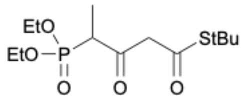

Sodium metal (884 mg, 38.4 mmol, 1.3 equiv.) was placed in a flask and washed with petroleum ether. Then, diethyl phosphite (7.3 g, 53.2 mmol, 1.8 equiv.) in THF (45 mL) was added dropwise *via* syringe. The reaction mixture was refluxed for 2.5 h to prepare the phosphite anion.

Sodium hydride (60% dispersion in mineral oil, 1.3 g, 32.6 mmol, 1.1 equiv.) was placed in a flask and washed with petroleum ether. Then, THF (50 mL) was added to the flask, and the resulting slurry was cooled to −30 °C. To the slurry was added a solution of **3** (7.9 g, 29.6 mmol) in THF (50 mL) dropwise *via* syringe. The resulting solution was stirred for 1.0 h and warmed to −20 °C before the addition of the solution of sodium diethyl phosphite *via* cannula. The reaction mixture was stirred overnight and slowly warmed to room temperature. The reaction was quenched with sat. NH_4Cl aq. (150 mL). The aqueous layer was extracted with Et_2O (200 mL × 3). The combined organic layers were washed with water (150 mL × 2) and brine (150 mL), dried over $MgSO_4$, filtered, and concentrated. The residue was purified by column chromatography eluted with *n*-hexane

/ AcOEt (gradient 10 : 0 to 1 : 1) to afford **4** as a red oil (9.0 g, 94%). Compound **4** exists as a (8.0 : 1.5) *keto* : *enol* mixture in $CDCl_3$ at room temperature. *R*f = 0.47 (*n*-hexane : AcOEt = 1 : 1); ^{1}H-NMR (400 MHz, $CDCl_3$) δ = 5.48 (d, *J* = 2.7 Hz, 0.15H, *enol*), 4.19–4.09 (m, 4H, *keto* / *enol*), 4.06 (d, *J* = 15.1 Hz, 0.85H, *keto*), 3.75 (d, *J* = 15.1 Hz, 0.85H, *keto*), 3.49 (dq, *J* = 26.4, 7.2 Hz, 0.85H, *keto*), 2.70 (dq, *J* = 23.2, 7.2 Hz, 0.15H, *enol*), 1.51 (s, 1.35H, *enol*), 1.47 (s, 7.2H, *keto*), 1.41–1.31 (m, 9H, *keto* / *enol*).

1.2.2 Synthesis and Characterization of HWE reagent 6

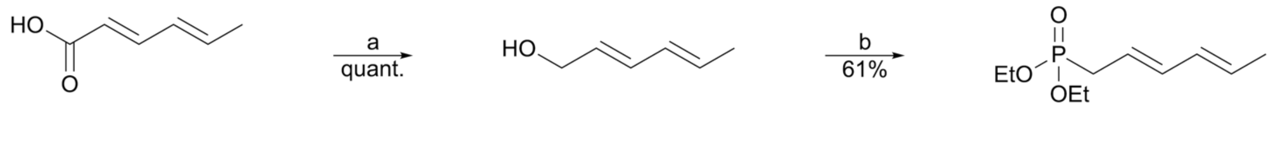

sorbic acid **5** **6**

***Scheme S2. Synthesis of HWE reagent 6*:** (a) (i) EDC•HCl, HOBt•H_2O, CH_2Cl_2, rt, 2.0 h; (ii) $NaBH_4$, H_2O, THF, 0 °C, 1.0 h; (b) (i) PBr_3, CH_2Cl_2, 0 °C, 1.0 h; (ii) $P(OEt)_3$, toluene, reflux, 14 h.

(2*E*, 4*E*)-Hexa-2,4-dien-1-ol (5)[2] [CAS: 17102-64-6]

To a solution of sorbic acid (10 g, 89.2 mmol) in CH_2Cl_2 (500 mL) were added EDC•HCl (18.6 g, 97.2 mmol, 1.1 equiv.) and HOBt•H_2O (14.9 g, 97.2 mmol, 1.1 equiv.). The reaction mixture was stirred at room temperature for 2.0 h. The mixture was washed with H_2O (200 mL × 2), and the aqueous layer was extracted with CH_2Cl_2 (200 mL). The combined organic portions were dried over $MgSO_4$, filtered, and concentrated to afford white solid.

The residue was dissolved in THF (500 mL) at 0 °C, and $NaBH_4$ (8.4 g, 223 mmol, 2.5 equiv.) was added. To the reaction mixture was added water (60 mL) dropwise, and the mixture was stirred at 0 °C for 1.0 h. The solution was diluted with MeOH (100 mL) and 10% citric acid aq. (50 mL). The organic solvent was removed, and the residue was extracted with $CHCl_3$ (150 mL × 3). The combined organic layers were washed with 10% citric acid aq. (100 mL × 2) and brine (200 mL), dried over $MgSO_4$, filtered, and concentrated. The residue was purified by column chromatography eluted with *n*-hexane / AcOEt (gradient 1 : 19 to 4 : 1) to afford **5** as a yellow oil (8.8 g, quant.). *R*f = 0.45 (*n*-hexane : AcOEt = 1 : 1); ^{1}H-NMR (400 MHz, $CDCl_3$) δ = 6.19 (dd, *J* = 14.4, 10.7 Hz, 1H), 6.05 (dd, *J* = 14.9, 10.7 Hz, 1H), 5.74–5.65 (m, 2H), 4.11 (d, *J* = 5.9 Hz, 2H), 2.72 (s, 1H), 1.75 (d, *J* = 6.9 Hz, 3H).

(2*E*, 4*E*)-Hexa-2,4-diene-1-diethylphosphonate (6)[1] [CAS: 41222-22-4]

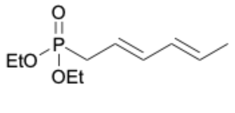

To a solution of **5** (6.7 g, 68.5 mmol) in CH_2Cl_2 (64 mL) was added PBr_3 (2.6 mL, 27.4 mmol, 0.4 equiv.) in CH_2Cl_2 (64 mL) over 0.5 h at 0 °C. The reaction mixture was stirred at 0 °C for 1.0 h and poured into sat. $NaHCO_3$ aq. (100 mL). The aqueous layer was extracted with CH_2Cl_2 (100 mL × 3). The combined organic layers were washed with brine (200 mL), dried over $MgSO_4$, filtered, and concentrated to afford yellow oil.

The residue was dissolved in toluene (137 mL) and $P(OEt)_3$ (135 mL, 788 mmol, 11.5 equiv.) was added. The mixture was refluxed for 14 h and concentrated by high vacuum at 60 °C. The residue was purified by column chromatography eluted with *n*-hexane / AcOEt (gradient 4 : 1 to 1 : 1) to afford **6** as a yellow oil (9.1 g, 61 % over 2 steps). *R*f = 0.35 (AcOEt only); ^{1}H-NMR (400 MHz, $CDCl_3$) δ = 6.17–6.10 (m, 1H), 6.08–6.01 (m, 1H), 5.70–5.61 (m, 1H), 5.49 (dq, *J* = 14.8, 7.6 Hz, 1H), 4.14–4.06 (m, 4H), 2.61 (dd, *J* = 22.4, 8.0 Hz, 2H), 1.75

(d, J = 7.6 Hz, 3H), 1.31 (t, J = 7.1 Hz, 6H).

1.2.3 Synthesis and Characterization of Serine derivative 10

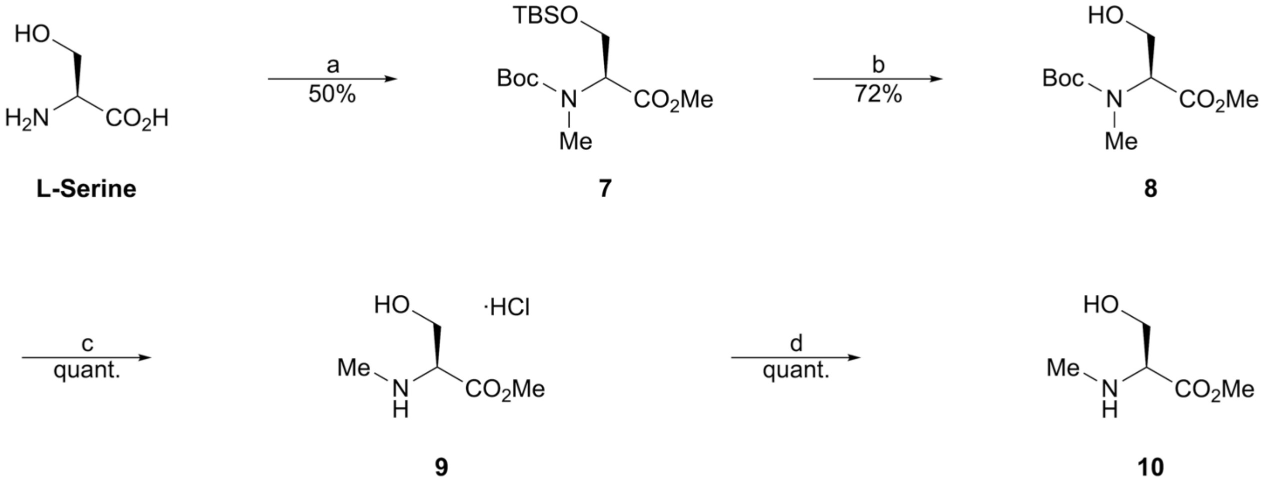

Scheme S3. Synthesis of N-methylserine ethyl ester 10**:** (a) (i) Boc_2O, 1,4-dioxane / NaOH aq., rt, overnight, (ii) TBDMSCl, imidazole, THF, rt, overnight, (iii) MeI, NaH, THF, rt, 4.5 h, (iv) EDC•HCl, DMAP, MeOH, CH_2Cl_2, rt, 10 h; (b) TBAF, THF, rt, 5.0 h; (c) 2 M HCl / EtOAc, rt, 1.5 h; (d) Et_3N, MeCN, rt, 1.0 h.

***N*-*tert*-Butoxycarbonyl-*N*-methyl-*O*-*tert*-butyldimethylsilyl-L-serine methyl ester (7)** [CAS 122902-77-6] To a solution of L-Ser-OH (10 g, 95.2 mmol) in 1,4-dioxane (100 mL) and 2 M NaOH aq. (200 mL) was added a solution of Boc_2O (33.2 g, 152 mmol, 1.6 equiv.) in 1,4-dioxane (100 mL) dropwise at 0 °C. The reaction mixture was stirred at room temperature overnight and then quenched with 1 M $KHSO_4$ aq. (250 mL), and AcOEt (100 mL) was added. The aqueous layer was extracted with AcOEt (150 mL × 2). The combined organic layers were washed with brine (200 mL), dried over $MgSO_4$, filtered, and concentrated to afford *N*-Boc derivatives as a white solid.

The residue was dissolved in THF (260 mL), and imidazole (19.4 g, 286 mmol, 3.0 equiv.) was added. To the mixture was added a solution of TBDMSCl (17.2 g, 114 mmol, 1.2 equiv.) in THF (140 mL) at 0 °C. The reaction mixture was stirred at room temperature overnight and filtered. The filtrate was mixed with 1 M $KHSO_4$ aq. (250 mL). The aqueous layer was extracted with AcOEt (150 mL × 2), and the combined organic layers were washed with brine (200 mL). The mixture was dried over $MgSO_4$, filtered, and concentrated to afford *O*-TBDMS derivatives as a white solid.

To a solution of the resulting crude in THF (400 mL), sodium hydride (60% dispersion in mineral oil, 15.2 g, 380 mmol, 4.0 equiv.) was added at 0 °C, and the mixture was stirred at 0 °C for 0.5 h. Then, methyl iodide (35.5 mL, 571 mmol, 6.0 equiv.) was added dropwise *via* syringe, and the mixture was stirred at room temperature for 4.5 h. The reaction mixture was diluted with 1 M $KHSO_4$ aq. (400 mL). The aqueous layer was extracted with AcOEt (300 mL × 2). The combined organic layers were washed with brine (300 mL), dried over $MgSO_4$, filtered, and evaporated to afford N-methyl derivatives as a yellow oil.

The residue was dissolved in CH_2Cl_2 (700 mL), EDC•HCl (20.1 g, 105 mmol, 1.1 equiv.), DMAP (16.3 g, 133 mmol, 1.4 equiv.), and MeOH (21.9 mL, 628 mmol, 6.6 equiv.) were added. The reaction mixture was stirred at room temperature for 10 h and 0.1 M HCl aq. (300 mL) was added. The organic layer was washed with $NaHCO_3$ aq. (300 mL) and brine (200 mL), dried over $MgSO_4$, filtered, and evaporated. The resulting residue

was purified by column chromatography eluted with *n*-hexane / AcOEt (gradient 9 : 1 to 1 : 1) to afford **7** as a yellow oil (16.6 g, 50% over 4 steps). *R*f = 0.74 (*n*-hexane : AcOEt = 7 : 3); ^{1}H-NMR (mixture of rotamers, 500 MHz, $CDCl_3$) δ = 4.71 and 4.32 (2 q, *J* = 4.0Hz and 4.0 Hz, 1H, rotamer), 4.04–3.93 (m, 2H), 3.70 and 3.69 (2 s, 3H, rotamer), 2.95 and 2.92 (2 s, 3H, rotamer), 1.44 and 1.40 (2 s, 9H, rotamer), 0.86 and 0.85 (2 s, 9H, rotamer), 0.04 and 0.03 (2 s, 6H, rotamer); ^{13}C-NMR (mixture of rotamers, 100 MHz, $CDCl_3$) δ = 170.6 and 170.6 (Ser-CO), 156.2 and 155.3 (Boc-CO), 80.4 and 80.0 (Boc-C), 62.3 and 61.8 (α-CH), 61.8 and 60.6 (β-CH_2), 52.0 (OMe), 33.7 and 33.0 (*N*-Me), 28.5 (Boc-CH_3), 25.8 (Si-C-CH_3), 18.2 and 18.1 (Si-C), −5.5 and −5.6 (Si-CH_3); HRMS (ESI+) *m*/*z* calcd for $C_{16}H_{33}NO_5SiNa$ $[M+Na]^+$ 370.2020, found 370.2031.

***N*-*tert*-Butoxycarbonyl-*N*-methyl-L-serine methyl ester (8)**[3] [CAS: 122902-81-2]

To a solution of **7** (12.8 g, 36.8 mmol) in THF (179 mL) was added TBAF (1 M solution in THF, 66.2 mL, 1.8 equiv.) dropwise *via* syringe at 0 °C. The mixture was stirred at 0 °C for 5.0 h, and the solvent was removed. The residue was dissolved in AcOEt (200 mL), and the mixture was washed with H_2O (100 mL) and brine (100 mL). The organic layer was dried over $MgSO_4$, filtered, and evaporated. The residue was purified by column chromatography eluted with *n*-hexane / AcOEt (gradient 9 : 1 to 1 : 1) to afford **8** as a yellow oil (6.1 g, 72%). *R*f = 0.26 (*n*-hexane : AcOEt = 1 : 1);^{1}H-NMR (mixture of rotamers, 500 MHz, $CDCl_3$) δ = 4.48 and 4.15 (2 t, *J* = 6.5 Hz and 6.5 Hz, 1H, rotamer), 4.05–3.99 (m, 1H), 3.82 and 3.74 (2 dd, *J* = 11.0, 6.5 Hz and 11.0, 6.5 Hz, 1H, rotamer), 3.68 (s, 3H), 3.28 and 3.03 (2 brs, 1H, rotamer), 2.89 and 2.84 (2 s, 3H, rotamer), 1.40 and 1.35 (2 s, 9H, rotamer); ^{13}C-NMR (mixture of rotamers, 125 MHz, $CDCl_3$) δ = 171.3 and 170.9 (Ser-CO), 156.5 and 155.2 (Boc-CO), 80.9 and 80.6 (Boc-C), 62.4 and 61.3 (α-CH), 61.0 and 60.8 (β-CH_2), 52.2 (OMe), 34.2 and 33.4 (*N*-Me), 28.3 (Boc-CH_3); HRMS (ESI+) *m*/*z* calcd for $C_{10}H_{19}NO_5Na$ $[M+Na]^+$ 256.1155, found 256.1164.

***N*-Methyl-L-serine methyl ester hydrochloride (9)**[3] [CAS: 207570-19-2]

To a solution of **8** (600 mg, 2.6 mmol) in AcOEt (2.4 mL) was added 4 M HCl in AcOEt (2.4 mL) dropwise at 0 °C. The reaction mixture was stirred at room temperature for 1.5 h and then evaporated to afford **9** as a white solid (436 mg, quant.). ^{1}H-NMR (400 MHz, CD_3OD) δ = 4.10 (t, *J* = 3.7 Hz, 1H), 4.03 (t, *J* = 3.7 Hz, 2H), 3.87 (s, 3H), 2.76 (s, 3H).

***N*-Methyl-L-serine methyl ester (10)**[4] [CAS: 111934-24-8]

To a solution of **9** (1.0 g, 5.9 mmol) in MeCN (5.9 mL) was added Et_3N (895 mg, 8.8 mmol, 1.5 equiv.). The mixture was stirred at room temperature for 1.0 h, and the resulting ammonium salts were filtered off and rinsed with AcOEt (5.9 mL). The filtrate was stirred at 0 °C for 10 min, and the precipitate was filtered. The filtrate was concentrated to ~30%(v/v) *in vacuo*. The resulting suspension was stirred at 0 °C for 10 min, filtered, and then evaporated to afford **10** as a yellow oil (790 mg, quant.). ^{1}H-NMR (400 MHz, CD_3OD) δ = 3.76 (dd, *J* = 1.8, 5.0 Hz, 1H), 3.74 (m, 1H), 3.74 (s, 3H), 3.31 (t, *J* = 5.0 Hz, 1H), 2.38 (s, 3H).

1.2.4 Synthesis and Characterization of β-Keto amide 15

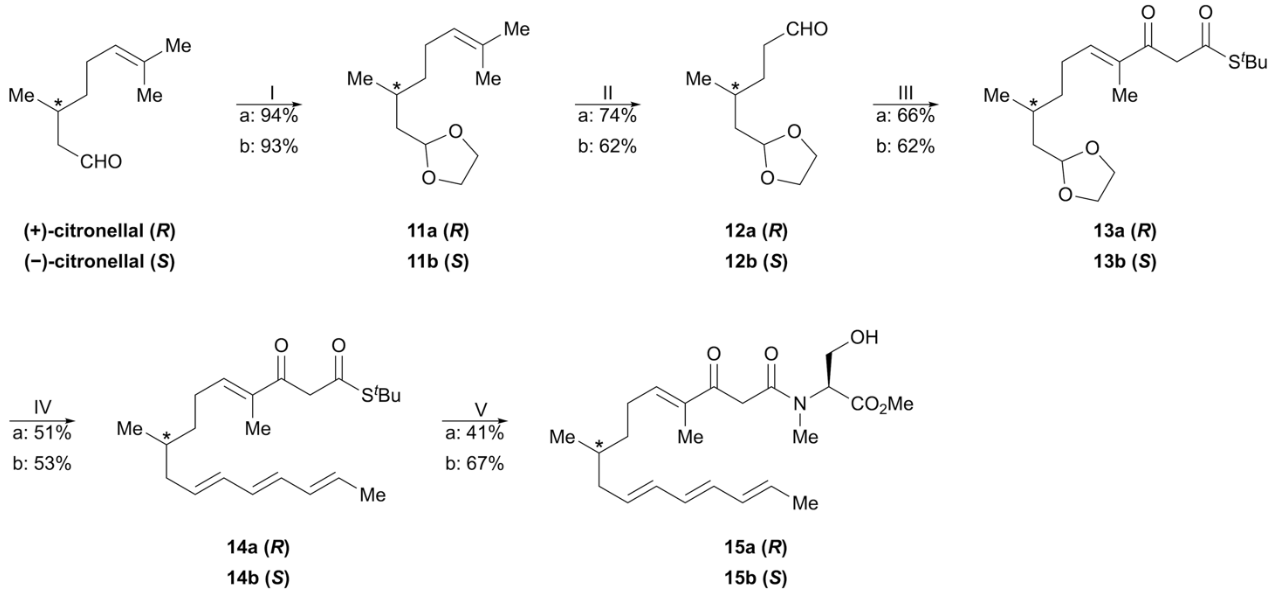

***Scheme S4. Synthesis of β-keto-amide 15*:** (I) ethylene glycol, (+)-CSA, $CH(OEt)_3$, CH_2Cl_2, rt, 4.0 h; (II) $RuCl_3$•*n*H_2O, $NaIO_4$, CH_2Cl_2, H_2O, rt; (III) **4**, LiHMDS, THF, −78 °C to rt; (IV) (i) 1 M HCl aq., THF, 60 °C, (ii) **6**, LiHMDS, THF, −78 °C to rt; (V) **10**, CF_3CO_2Ag, Et_3N, THF, −10 °C.

2-((*R*)-2,6-Dimethylhept-5-enyl)-1,3-dioxolane (11a)[5] [CAS: 1205-81-8]

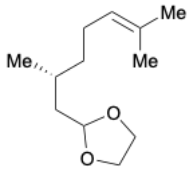

Ethylene glycol (10.8 mL, 195 mmol, 15 equiv.), (+)-CSA (151 mg, 0.65 mmol, 0.05 equiv.), and triethyl orthoformate (6.5 mL, 38.9 mmol, 3.0 equiv.) were dissolved in CH_2Cl_2 (94 mL). To the mixture, (+)-citronellal (2.0 g, 13.0 mmol) was added dropwise over 10 min. The reaction mixture was stirred at room temperature for 4.0 h, and sat. $NaHCO_3$ aq. (100 mL) was added. The aqueous layer was extracted with CH_2Cl_2 (100 mL × 3). The combined organic layers were washed with brine (150 mL), dried over $MgSO_4$, filtered, and concentrated. The residue was purified by column chromatography eluted with *n*-hexane / AcOEt (gradient 10 : 0 to 9 : 1) to afford **11a** as a colorless oil (2.4 g, 94%). *R*f = 0.37 (*n*-hexane : CH_2Cl_2 = 7 : 3); ^{1}H-NMR (500 MHz, $CDCl_3$) δ = 5.10 (tt, *J* = 7.2, 1.4 Hz, 1H), 4.90 (t, *J* = 4.9 Hz, 1H), 4.01–3.93 (m, 2H), 3.88–3.82 (m, 2H), 2.05–1.92 (m, 2H), 1.70–1.65 (m, 2H), 1.68 (s, 3H), 1.60 (s, 3H), 1.53–1.47 (m, 1H), 1.42–1.35 (m, 1H), 1.24–1.16 (m, 1H), 0.96 (d, *J* = 6.3 Hz, 3H).

2-((*S*)-2,6-Dimethylhept-5-enyl)-1,3-dioxolane (11b)[6] [CAS: 134876-96-3]

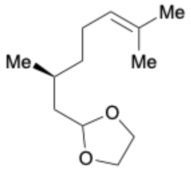

The target compound **11b** (5.9 g, 93%) as a colorless oil was synthesized according to the procedure for the synthesis of **11a** by using (−)-citronellal (5.0 g, 32.4 mmol). *R*f = 0.37 (*n*-hexane : CH_2Cl_2 = 7 : 3); ^{1}H-NMR (400 MHz, $CDCl_3$) δ = 5.10 (tt, *J* = 7.1, 1.4 Hz, 1H), 4.90 (t, *J* = 5.0 Hz, 1H), 4.01–3.92 (m, 2H), 3.88–3.79 (m, 2H), 2.06–1.91 (m, 2H), 1.70–1.64 (m, 2H), 1.68 (s, 3H), 1.60 (s, 3H), 1.53–1.46 (m, 1H), 1.43–1.34 (m, 1H), 1.24–1.15 (m, 1H), 0.96 (d, *J* = 6.4 Hz, 3H).

(*R*)-5-(1,3-Dioxolan-2-yl)-4-methylpentanal (12a)[7] [CAS: 1446-54-4]

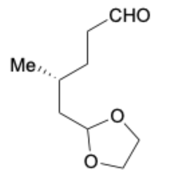

To a solution of **11a** (3.9 g, 19.7 mmol) and $RuCl_3$•$n$$H_2O$ (85% content, 110 mg, 0.45 mmol, 0.02 equiv.) in CH_2Cl_2 (92 mL) and H_2O (92 mL) was added $NaIO_4$ (8.4 g, 39.4 mmol, 2.0 equiv.) in portions over 5 min. The reaction mixture was stirred vigorously at room temperature for 4.5 h, and sat. $NaHCO_3$ aq. (150 mL) was added. The aqueous layer was extracted with AcOEt (100 mL × 3). The combined organic layers were washed with brine (150 mL), dried over $MgSO_4$, filtered, and concentrated. The residue was purified by column chromatography eluted with *n*-hexane / AcOEt (gradient 10 : 0 to 1 : 1) to afford **12a** as a volatility yellow oil (2.5 g, 74%). *R*f = 0.39 (*n*-hexane : AcOEt = 7 : 3); ^{1}H-NMR (400 MHz, $CDCl_3$) *δ* = 9.78 (t, *J* = 1.8 Hz, 1H), 4.90 (dd, *J* = 5.5, 4.6 Hz, 1H), 4.01–3.92 (m, 2H), 3.89–3.80 (m, 2H), 2.54–2.34 (m, 2H), 1.79–1.63 (m, 2H), 1.79–1.63 (m, 1H), 1.58–1.46 (m, 2H), 0.97 (d, *J* = 6.4 Hz, 3H).

(*S*)-5-(1,3-Dioxolan-2-yl)-4-methylpentanal (12b)[8] [CAS: 1627157-39-4]

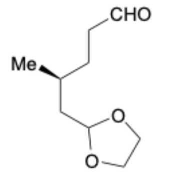

The target compound **12b** (2.6 g, 64%) was obtained as a volatile yellow oil according to the procedure for the synthesis of **12a** by using **11b** (4.7 g, 24.0 mmol). *R*f = 0.39 (*n*-hexane : AcOEt = 7 : 3); ^{1}H-NMR (400 MHz, $CDCl_3$) δ = 9.78 (t, *J* = 1.8 Hz, 1H), 4.90 (dd, *J* = 5.0, 5.0 Hz, 1H), 4.01–3.92 (m, 2H), 3.89–3.80 (m, 2H), 2.54–2.39 (m, 2H), 1.79–1.63 (m, 2H), 1.79–1.63 (m, 1H), 1.58–1.46 (m, 2H), 0.97 (d, *J* = 6.4 Hz, 3H).

(4*E*)-(*R*)-*tert*-Butyl 8-(1,3-dioxolan-2-yl)-4-methyl-3-oxonon-4-enethioate (13a)[9] [CAS: 1544617-90-4]

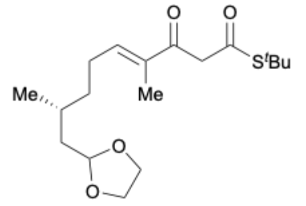

To a solution of **4** (5.8 g, 17.9 mmol, 1.3 equiv.) in THF (97 mL) was added LiHMDS (1.3 M solution in THF, 23.3 mL, 30.3 mmol, 2.2 equiv.) dropwise *via* syringe at −78 °C. After stirring at −78 °C for 0.5 h, a solution of **12a** (2.3 g, 13.8 mmol) in THF (29 mL) was added dropwise *via* cannula. The reaction mixture was stirred at −78 °C for 0.5 h, slowly warmed to room temperature, and stirred overnight. The reaction was quenched with sat. NH_4Cl aq. (100 mL). The aqueous layer was extracted with AcOEt (100 mL × 3), and the combined organic layers were washed with brine (150 mL), dried over $MgSO_4$, filtered, and evaporated. The resulting residue was purified by column chromatography eluted with *n*-hexane / AcOEt (gradient 10 : 0 to 9 : 1) to afford **13a** as a pale yellow oil (3.1 g, 66%). Compound **13a** exists as a (6.0 : 4.0) *keto* : *enol* mixture in $CDCl_3$ at room temperature. *R*f = 0.64 (*n*-hexane : AcOEt = 7 : 3); ^{1}H-NMR (400 MHz, $CDCl_3$) *δ* = 6.65 (m, 0.6H, *keto*), 6.57 (m, 0.4H, *enol*), 5.50 (s, 0.4H, *enol*), 4.91–4.88 (m, 1H, *keto* / *enol*), 4.00–3.92 (m, 2H, *keto* / *enol*), 3.89–3.81 (m, 2H, *keto* / *enol*), 3.84 (s, 1.2H, *keto*), 2.35–2.13 (m, 2H, *keto* / *enol*), 1.79 (d, *J* = 1.2 Hz, 1.8H, *keto*), 1.74 (d, *J* = 1.2 Hz, 1.2H, *enol*), 1.74–1.62 (m, 2H, *keto* / *enol*), 1.58–1.52 (m, 2H, *keto* / *enol*), 1.52 (s, 3.6H, *enol*), 1.47 (s, 5.4H, *keto*), 1.40–1.22 (m, 1H, *keto* / *enol*), 0.99 (d, *J* = 6.9 Hz, 1.8H, *keto*), 0.96 (d, *J* = 6.9 Hz, 1.2H, *enol*).

(4*E*)-(*S*)-*tert*-Butyl 8-(1,3-dioxolan-2-yl)-4-methyl-3-oxonon-4-enethioate (13b)[10] [CAS: 1627157-40-7]

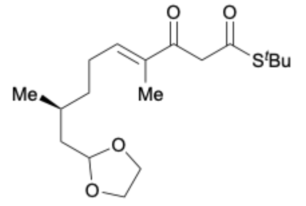

The target compound **13b** (2.9 g, 62%) was synthesized according to the procedure for the synthesis of **13a** by using **12b** (2.3 g, 13.5 mmol). Compound **13b** exists as a (6.2 : 3.8) *keto* : *enol* mixture in $CDCl_3$ at room temperature. *R*f = 0.63 (*n*-hexane : AcOEt = 7 : 3); ^{1}H-NMR (500 MHz, $CDCl_3$) *δ* = 6.65 (m, 0.62H, *keto*), 6.57 (m, 0.38H, *enol*), 5.50 (s, 0.38H, *enol*), 4.91–4.88 (m, 1H, *keto* / *enol*), 4.01–3.93 (m, 2H, *keto* / *enol*), 3.88–3.80 (m, 2H, *keto* / *enol*), 3.84 (s, 1.24H, *keto*), 2.35–2.14 (m, 2H, *keto* / *enol*), 1.79 (d, *J* = 1.1 Hz, 1.86H, *keto*), 1.74 (d, *J* = 1.1 Hz, 1.14H, *enol*), 1.76–1.65 (m, 2H, *keto* / *enol*), 1.59–1.50 (m, 2H,

keto / *enol*), 1.52 (s, 3.42H, *enol*), 1.47 (s, 5.58H, *keto*), 1.39–1.24 (m, 1H, *keto* / *enol*), 0.99 (d, J = 6.9 Hz, 1.86H, *keto*), 0.98 (d, J = 6.3 Hz, 1.14H, *enol*); ^{13}C-NMR (100 MHz, $CDCl_3$) δ = 196.3 (*enol*), 193.5, 193.2, 169.7 (*enol*), 145.8, 137.5 (*enol*), 136.9, 128.2 (*enol*), 103.4 (*enol*), 103.4, 97.2 (*enol*), 64.6, 64.6, 53.7, 48.7, 48.1 (*enol*), 40.6 (*enol*), 40.5, 36.2 (*enol*), 35.7, 30.1 (*enol*), 29.5, 29.1, 29.0 (*enol*), 26.7, 26.0 (*enol*), 19.7, 12.0 (*enol*), 11.2; HRMS (ESI+) *m*/*z* calcd for $C_{18}H_{31}O_4S$ $[M+H]^+$ 343.1938, found 343.1941.

(4*E*,10*E*,12*E*,14*E*)-(*R*)-*tert*-Butyl 4,8-dimethyl-hexadeca-4,10,12,14-butene-*β*-ketothioate (14a)[9] [CAS: 313072-23-0]

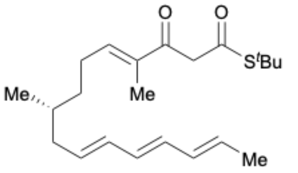

To a solution of **13a** (2.9 g, 8.5 mmol) in THF (43 mL) was added 1 M HCl aq. (42.5 mL, 42.5 mmol, 5.0 equiv.). The reaction mixture was stirred at 60 °C for 2.5 h and neutralized (to pH 7) with sat. $NaHCO_3$ aq.. The aqueous layer was extracted with AcOEt (50 mL × 2). The combined organic layers were washed with sat. $NaHCO_3$ aq. (100 mL), brine (100 mL), dried over $MgSO_4$, filtered, and evaporated to afford the aldehyde derivative as a yellow oil.

To a solution of **6** (contained $POEt_3$; 86% purity, 6.3 g, 25.5 mmol, 3.0 equiv.) in THF (111 mL) was added LiHMDS (1.3 M solution in THF, 22.8 mL, 29.6 mmol, 3.1 equiv.) dropwise *via* syringe at −78 °C. After stirring at −78 °C for 30 min, a solution of the above yellow oil in THF (24 mL) was added dropwise *via* cannula. The reaction mixture was stirred at −78 °C for 30 min, and then warmed to room temperature, and stirred at room temperature overnight. The reaction mixture was quenched with sat. NH_4Cl aq. (100 mL). The aqueous layer was extracted with AcOEt (100 mL × 2), and the combined organic layers were washed with H_2O (50 mL × 2) and brine (150 mL), dried over $MgSO_4$, filtered, and concentrated. The residue was purified by column chromatography eluted with *n*-hexane / AcOEt (gradient 100 : 0 to 97 : 3) to afford **14a** as a yellow oil (1.6 g, 51% over 2 steps). Compound **14a** exists as a (6.1 : 3.9) *keto* : *enol* mixture in $CDCl_3$ at room temperature. *R*f = 0.70 (*n*-hexane : AcOEt = 19 : 1, 2 times); ^{1}H-NMR (400 MHz, $CDCl_3$) δ = 6.63 (m, 0.61H, *keto*), 6.56 (m, 0.39H, *enol*), 6.21–5.99 (m, 4H, *keto* / *enol*), 5.74–5.56 (m, 2H, *keto* / *enol*), 5.50 (s, 0.39H, *enol*), 3.83 (s, 1.22H, *keto*), 2.35–2.17 (m, 2H, *keto* / *enol*), 2.15–2.07 (m, 1H, *keto* / *enol*), 2.01–1.88 (m, 1H, *keto* / *enol*), 1.79 (s, 1.83H, *keto*), 1.76 (d, J = 6.9 Hz, 3H, *keto* / *enol*), 1.73 (s, 1.17H, *enol*), 1.60–1.43 (m, 2H, *keto* / *enol*), 1.52 (s, 3.51H, *enol*), 1.46 (s, 5.49H, *keto*), 1.33–1.21 (m, 1H, *keto* / *enol*), 0.91 (d, J = 6.4 Hz, 1.83H, *keto*), 0.89 (d, J = 6.4 Hz, 1.17H, *enol*).

(4*E*,10*E*,12*E*,14*E*)-(*S*)-*tert*-Butyl 4,8-dimethyl-hexadeca-4,10,12,14-butene-*β*-ketothioate (14b)

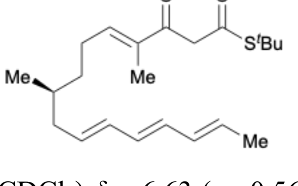

The target compound **14b** (1.4 g, 53% over 2 steps) as a yellow oil was synthesized according to the procedure for the synthesis of **14a** by using **13b** (2.4 g, 7.5 mmol). Compound **14b** exists as a (1.4 : 1.1) *keto* : *enol* mixture in $CDCl_3$ at room temperature. *R*f = 0.72 (*n*-hexane : AcOEt = 19 : 1, 2 times); ^{1}H-NMR (400 MHz, $CDCl_3$) δ = 6.63 (m, 0.56H, *keto*), 6.56 (m, 0.44H, *enol*), 6.21–5.99 (m, 4H, *keto* / *enol*), 5.74–5.58 (m, 2H, *keto* / *enol*), 5.50 (s, 0.44H, *enol*), 3.83 (s, 1.12H, *keto*), 2.33–2.17 (m, 2H, *keto* / *enol*), 2.15–2.06 (m, 1H, *keto* / *enol*), 2.01–1.91 (m, 1H, *keto* / *enol*), 1.79 (s, 1.68H, *keto*), 1.77 (d, J = 6.9 Hz, 3H, *keto* / *enol*), 1.73 (s, 1.32H, *enol*), 1.62–1.41 (m, 2H, *keto* / *enol*), 1.52 (s, 3.96H, *enol*), 1.46 (s, 5.04H, *keto*), 1.31–1.20 (m, 1H, *keto* / *enol*), 0.91 (d, J = 6.4 Hz, 1.68H, *keto*), 0.89 (d, J = 6.4 Hz, 1.32H, *enol*); ^{13}C-NMR (125 MHz, $CDCl_3$) δ = 196.6 (*enol*), 193.8, 193.5, 170.0 (*enol*), 146.2, 137.9 (*enol*), 137.1, 132.5 (*enol*), 132.2, 132.2, 132.1 (*enol*), 131.9 (*enol*), 131.8, 131.2, 131.1 (*enol*), 130.6 (*enol*), 130.5, 129.3, 129.1 (*enol*), 97.5 (*enol*), 53.9 (*keto* / *enol*),

49.0, 48.4 (*enol*), 40.3 (*enol*), 40.3, 35.6 (*enol*), 35.2, 33.2, 33.1 (*enol*), 30.3 (*enol*), 29.8, 27.1, 26.5 (*enol*), 19.5 (*keto* / *enol*), 18.4 (*keto* / *enol*), 12.2 (*enol*), 11.4 (*keto*); HRMS (ESI+) *m*/*z* calcd for $C_{22}H_{34}O_2SNa$ $[M+Na]^+$ 385.2172, found 385.2183.

(4*E*,10*E*,12*E*,14*E*)-(*R*)-*tert*-Butyl 4,8-dimethyl-hexadeca-4,10,12,14-butene-*β*-keto-*N*-methylserinemethyl-ester (15a)[9] [CAS: 1544618-01-0]

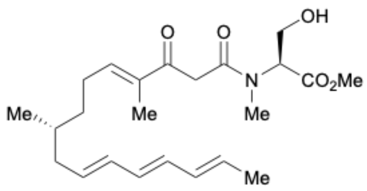

Compound **14a** (300 mg, 0.83 mmol), **10** (143 mg, 1.1 mmol, 1.3 equiv.), and Et_3N (460 µL, 3.3 mmol, 4.0 equiv.) were dissolved in THF (16.5 mL). The mixture was cooled to −10 °C and CF_3CO_2Ag (183 mg, 0.83 mmol, 1.0 equiv.) was added. The reaction mixture was stirred at −10 °C for 1.5 h, filtered by celite, and then the celite was washed with CH_2Cl_2 (50 mL). The collected filtrate was evaporated, and the residue was purified by column chromatography eluted with petroleum ether / AcOEt (gradient 7 : 3 to 0 : 10) to afford **15a** as a mixture (170 mg, 42%, calculated by ^{1}H-NMR, contained DA-proceeded products[9] 19 %). Compound **15a** exists as a (7.7 : 2.3) *keto* : *enol* mixture in $CDCl_3$ at room temperature. *R*f = 0.25 (petroleum ether : AcOEt = 1 : 1); ^{1}H-NMR (400 MHz, $CDCl_3$) δ = 6.68 (t, *J* = 7.1 Hz, 0.77H, *keto*), 6.52 (m, 0.23H, *enol*), 6.21–6.01 (m, 4H, *keto* / *enol*), 5.73–5.58 (m, 2H, *keto* / *enol*), 4.92–4.84 (m, 1H, *keto*), 4.68–4.65 (m, 1H, *enol*), 4.07–3.97 (m, 2H, *keto* / *enol*), 3.90 (s, 1.54H, *keto*), 3.79–3.75 (m, 3H, *keto* / *enol*), 3.09–2.90 (m, 3H, *keto* / *enol*), 2.33–2.20 (m, 2H, *keto* / *enol*), 2.14–2.07 (m, 1H, *keto* / *enol*), 2.04–2.06 (1H, *keto* / *enol*), 2.02–1.95 (m, 1H, *keto* / *enol*), 1.80 (s, 3H, *keto* / *enol*), 1.77 (d, *J* = 7.1 Hz, 3H, *keto* / *enol*), 1.60–1.46 (m, 2H, *keto* / *enol*), 1.31–1.27 (m, 2H, *keto* / *enol*), 0.92 (d, *J* = 6.4 Hz, 3H, *keto* / *enol*).

(4*E*,10*E*,12*E*,14*E*)-(*S*)-*tert*-Butyl-4,8-dimethyl-hexadeca-4,10,12,14-butene-*β*-keto-*N*-methylserinemethyl-ester (15b)

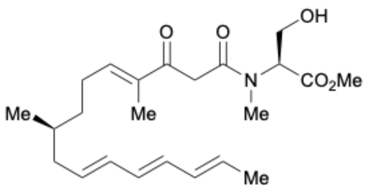

The target compound **15b** (150 mg, 67%) was obtained as a yellow oil according to the procedure for the synthesis of **15a** by using **14b** (200 mg, 0.55 mmol). Compound **15b** exists as a (8.5 : 1.5) *keto* : *enol* mixture in $CDCl_3$ at room temperature. *R*f = 0.25 (petroleum ether : AcOEt = 1 : 1); ^{1}H-NMR (400 MHz, $CDCl_3$) δ = 6.83 (t, *J* = 6.6 Hz, 0.15H, *enol*), 6.68 (t, *J* = 7.3 Hz, 0.85H, *keto*), 6.21–6.03 (m, 4H, *keto* / *enol*), 5.74–5.58 (m, 2H, *keto* / *enol*), 5.32 (s, 0.15H, *enol*), 4.89–4.83 (m, 0.85H, *keto*), 4.70–4.66 (m, 0.15H, *enol*) 4.11–3.99 (m, 2H, *keto* / *enol*), 3.90 (s, 1.7H, *keto*), 3.77 (s, 0.45H, *enol*), 3.75 (s, 2.55H, *keto*), 3.02 (s, 3H, *keto* / *enol*), 2.33–2.23 (m, 2H, *keto* / *enol*), 2.14–2.07 (m, 1H, *keto* / *enol*), 2.02–1.95 (m, 1H, *keto* / *enol*), 1.80 (s, 3H, *keto* / *enol*), 1.77 (d, *J* = 6.9 Hz, 3H, *keto* / *enol*), 1.58–1.47 (m, 2H, *keto* / *enol*), 1.35–1.29 (m, 1H, *keto* / *enol*), 0.90 (d, *J* = 6.4 Hz, 3H, *keto* / *enol*); ^{13}C-NMR (100 MHz, $CDCl_3$) δ = 195.4, 170.0, 169.2, 146.2, 136.9, 132.3, 132.1, 131.8, 131.3, 130.4, 129.4, 61.1, 60.2, 52.5, 44.7, 40.2, 35.7, 35.1, 33.3, 27.1, 19.5, 18.4, 11.4; HRMS (ESI+) *m*/*z* calcd for $C_{23}H_{36}NO_5$ $[M+H]^+$ 406.2588, found 406.2599.

1.2.5 Separation and Characterization of cis-decalin 1B and 2B

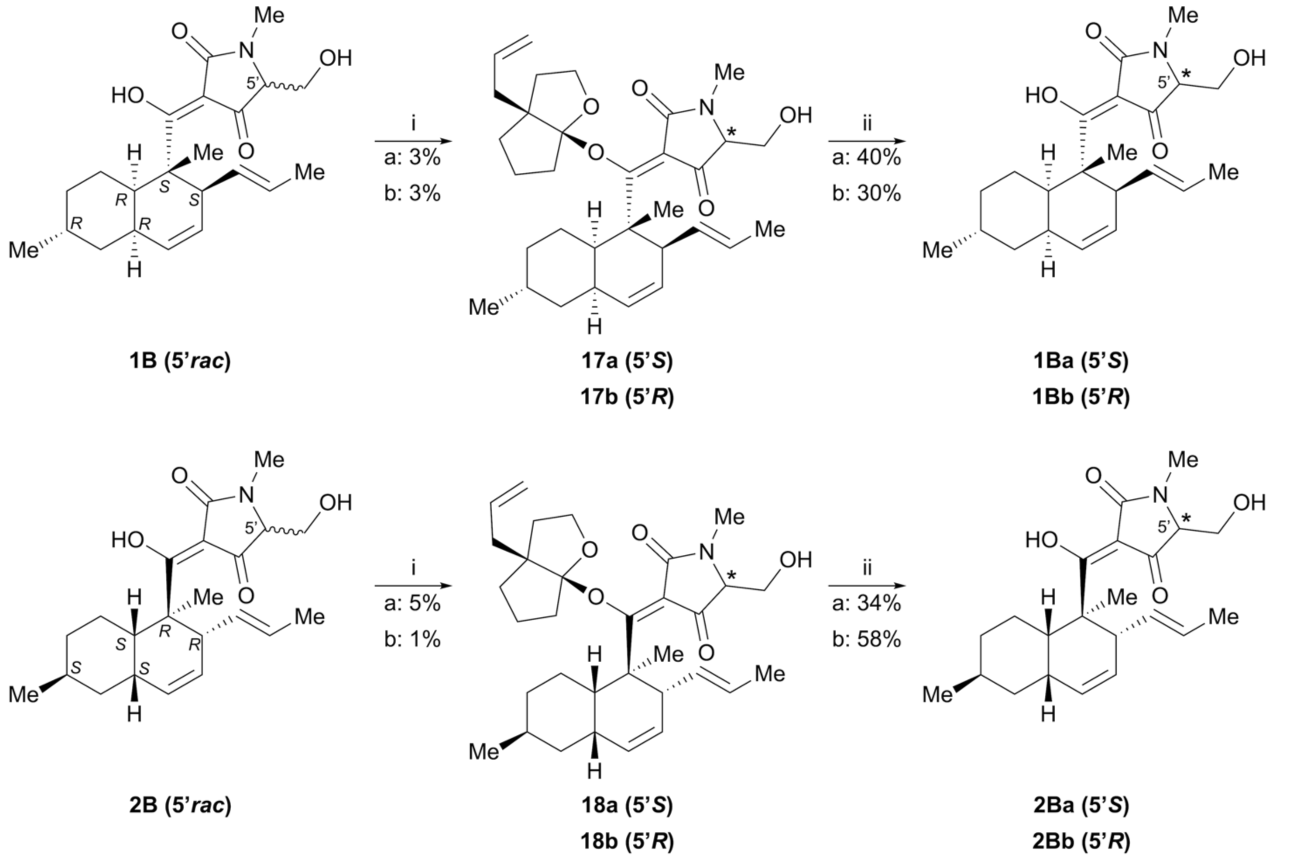

***Scheme S5. Separation of cis-decalin 1B and 2B*:** (i) (*S*)-5-allyl-2-oxabicyclo[3.3.0]oct-8-ene, PPTS, CH_2Cl_2, rt, 17 h; (ii) PPTS, MeOH, reflux, 3.0 h.

(3*Z*,5*S*)-3-({[(3aS,6aS)-3a-allylhexahydro-6aH-cyclopenta[b]furan-6a-yl]oxy}[(1*S*,2*S*,4a*R*,6*R*,8a*R*)-1,6-dimethyl-2-[(*E*)-prop-1-enyl]-4a,5,6,7,8,8a-hexahydro-2*H*-naphthalen-1-yl]-methylidene)-5-(hydroxymethyl)-1-methylpyrrolidine-2,4-dione (17a)

(3*Z*,5*R*)-3-({[(3aS,6aS)-3a-allylhexahydro-6aH-cyclopenta[b]furan-6a-yl]oxy}[(1*S*,2*S*,4a*R*,6*R*,8a*R*)-1,6-dimethyl-2-[(*E*)-prop-1-enyl]-4a,5,6,7,8,8a-hexahydro-2*H*-naphthalen-1-yl]-methylidene)-5-(hydroxymethyl)-1-methylpyrrolidine-2,4-dione (17b)

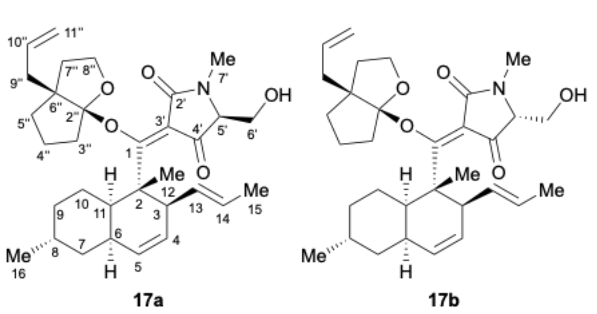

To a solution of the crude compound **1B** (121 mg) in CH_2Cl_2 (1 mL) were added (*S*)-5-allyl-2-oxabicyclo[3.3.0]oct-8-ene (58 mg, 38.9 μmol) and PPTS (1 mg, 4.0 μmol, 0.1 equiv.). The reaction mixture was stirred at room temperature for 17 h and poured into sat. $NaHCO_3$ aq. (20 mL). The aqueous layer was extracted with AcOEt (20 mL × 3), and the combined organic layers were washed with brine (30 mL), dried over Na_2SO_4 ,filtered, and evaporated. The resulting residue was purified by preparative HPLC eluted with A / B (1 : 19 over 50 min) to afford compounds **17a** and **17b** as red oils(**17a**; 14 mg, 3% over 3 steps, **17b**; 13 mg, 3% over 3 steps).

Compound 17a : *R*f = 0.71 (petroleum ether : AcOEt = 1 : 4); ^{1}H-NMR (600 MHz, $CDCl_3$) δ = 5.74–5.68 (m,

1H, C10”), 5.66–5.63 (m, 1H, C4), 5.50–5.43 (m, 2H, C13, C14), 5.41–5.39 (m, 1H, C5), 5.01–4.96 (m, 2H, C11”), 4.02 (dd, *J* = 10.2, 4.1 Hz, 1H, C6’a), 3.86–3.83 (m, 1H, C8”a), 3.81–3.77 (m, 1H, C6’b), 3.73 (brs, 1H, C5’), 3.67–3.63 (m, 1H, C8”b), 3.34 (brs, 1H, C3), 3.06 (s, 3H, C7’), 2.53 (brd, *J* = 12.0 Hz, 1H, C11), 2.18 (brs, 1H, C6), 2.12 (dd, *J* = 11.9, 4.7 Hz, 1H, C7”a), 2.02 (dd, *J* = 13.1, 7.2 Hz, 1H, C9”a), 1.97 (dd, *J* = 13.1, 7.2 Hz, 1H, C9”b), 1.88–1.85 (m, 1H, C3”a), 1.68–1.59 (m, 7H, C7*eq*, C9*eq*, C10*eq*, C3”b, C4”, C5”a), 1.68 (brs, 3H, C15), 1.58–1.42 (m, 3H, C10*ax*, C5”b, C”7b), 1.37–1.33 (m, 1H, C8), 1.33 (brs, 3H, C12), 1.04–0.99 (m, 1H, C7*ax*), 0.89–0.82 (m, 1H, C9*ax*), 0.80 (d, *J* = 6.4 Hz, 3H, C16); ^{13}C-NMR (100 MHz, $CDCl_3$) δ = 200.7 (C1), 188.9 (C4’), 177.5 (C2’), 136.2 (C10”), 131.8 (C14), 130.6 (C4), 129.8 (C5), 127.6 (C13), 117.4 (C2”), 117.3 (C11”), 98.0 (C3’), 66.4 (C5’), 66.3 (C8”), 60.9 (C6’), 54.3 (C6”), 49.3 (C2), 42.5 (C3), 40.9 (C7), 40.1 (C9”), 38.7 (C3”), 37.9 (C11), 36.9 (C5”), 35.5 (C9), 34.6 (C6), 34.5 (C7”), 28.3 (C8), 27.7 (C7’), 23.0 (C10), 22.7 (C16), 21.6 (C4”), 18.7 (C12), 18.2 (C15); HRMS (ESI+) *m* / *z* calcd for $C_{32}H_{45}NO_5Na$ $[M+Na]^+$ 546.3190, found 546.3182.

Compound 17b : *R*f = 0.71 (petroleum ether : AcOEt = 2 : 8); ^{1}H-NMR (600 MHz, $CDCl_3$) δ = 5.69–5.62 (m, 2H, C10”, C4), 5.44–5.40 (m, 3H, C5, C13, C14), 4.97–4.93 (m, 2H, C11”), 4.03 (brd, *J* = 9.0 Hz, 1H, C6’a), 3.83–3.81 (m, 2H, C6’b, C8”a), 3.69 (brs, 1H, C5’), 3.61–3.57 (m, 1H, C8”b), 3.35 (brs, 1H, C3), 3.06 (s, 3H, C7’), 2.66 (brd, *J* = 10.5 Hz, 1H, C11), 2.19 (brs, 1H, C6), 2.02–1.95 (m, 2H, C7”a, C9”a), 1.87–1.85 (m, 2H, C3”a, C9”b), 1.68–1.58 (m, 5H, C7*eq*, C9*eq*, C10*eq*, C3”b, C4”a), 1.68 (brs, 3H, C15), 1.50 (brs, 4H, C4”b, C5”, C7”b, overlap), 1.44–1.42 (m, 1H, C10*ax*), 1.34–1.32 (m, 1H, C8), 1.27 (s, 3H, C12), 1.13–1.08 (m, 1H, C7*ax*), 0.93–0.86 (m, 1H, C9*ax*), 0.81 (brs, 3H, C16); ^{13}C-NMR (100 MHz, $CDCl_3$) δ = 200.4 (C1), 189.8 (C4’), 177.6 (C2’), 136.4 (C10”), 131.7 (C14), 130.6 (C4), 129.8 (C5), 127.6 (C13), 117.8 (C2”), 117.1 (C11”), 98.4 (C3’), 66.5 (C5’), 66.3 (C8”), 60.0 (C6’), 54.3 (C6”), 49.3 (C2), 42.3 (C3), 40.7 (C7), 40.2 (C9”), 38.4 (C3”), 37.9 (C11), 37.4 (C5”), 35.4 (C9), 34.8 (C6), 34.6 (C7”), 28.5 (C8), 27.0 (C7’), 23.0 (C10), 22.7 (C16), 21.6 (C4”), 18.4 (C12), 18.2 (C15); HRMS (ESI+) *m/z* calcd for $C_{32}H_{45}NO_5Na$ $[M+Na]^+$ 546.3190, found 546.3193.

(3*Z*,5*S*)-3-({[(3aS,6aS)-3a-allylhexahydro-6aH-cyclopenta[b]furan-6a-yl]oxy}[(1*R*,2*R*,4a*S*,6*S*,8a*S*)-1,6-dimethyl-2-[(*E*)-prop-1-enyl]-4a,5,6,7,8,8a-hexahydro-2*H*-naphthalen-1-yl]-methylidene)-5-(hydroxymethyl)-1-methylpyrrolidine-2,4-dione (18a)

(3*Z*,5*R*)-3-({[(3aS,6aS)-3a-allylhexahydro-6aH-cyclopenta[b]furan-6a-yl]oxy}[(1*R*,2*R*,4a*S*,6*S*,8a*S*)-1,6-dimethyl-2-[(*E*)-prop-1-enyl]-4a,5,6,7,8,8a-hexahydro-2*H*-naphthalen-1-yl]-methylidene)-5-(hydroxymethyl)-1-methylpyrrolidine-2,4-dione (18b)

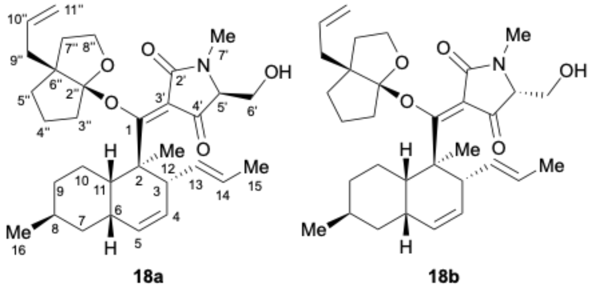

18a **18b**

The target compound **18a**, **18b** (**18a**; 12 mg, 5% over 3 steps, **18b**; 3.0 mg, 1% over 3 steps) was synthesized according to the procedure for the synthesis of compound **17a** by using compound **2B** (30 mg).

Compound 18a : *R*f = 0.71 (petroleum ether : AcOEt = 1 : 4); ^{1}H-NMR (600 MHz, $CDCl_3$) δ = 5.75–5.68 (m, 1H, C10”), 5.63–5.61 (m, 1H, C4), 5.50–5.43 (m, 2H, C13, C14), 5.41–5.38 (m, 1H, C5), 5.01–4.96 (m, 2H, C11”), 4.00 (dd, *J* = 10.1, 4.0 Hz, 1H, C6’a), 3.81 (td, *J* = 8.2, 3.7 Hz, 1H, C8”a), 3.75 (dd, *J* = 10.1, 2.6 Hz, 1H, C6’b), 3.73 (dd, *J* = 4.0, 2.6 Hz, 1H, C5’), 3.65–3.59 (m, 1H, C8”b), 3.37 (brs, 1H, C3), 3.05 (s, 3H, C7’), 2.64 (dt, *J* = 12.4, 3.5 Hz, 1H, C11), 2.19 (brs, 1H, C6),

2.10–2.07 (m, 1H, C7”a), 2.03 (dd, J = 13.8, 7.2 Hz, 1H, C9”a), 1.96 (dd, J = 13.8, 7.2 Hz, 1H, C9”b), 1.81 (ddd, J = 12.2, 6.8, 3.7 Hz, 1H, C3”a), 1.68–1.62 (m, 3H, C9*eq*, C10*eq*, C3”b), 1.67 (brs, 3H, C15), 1.61–1.55 (m, 5H, C7*eq*, C4”, C5”), 1.52–1.40 (m, 2H, C10*ax*, C7”b), 1.37–1.32 (m, 1H, C8), 1.29 (brs, 3H, C12), 1.14–1.07 (m, 1H, C7*ax*), 0.93–0.86 (m, 1H, C9*ax*), 0.81 (d, J = 6.5 Hz, 3H, C16); ^{13}C-NMR (100 MHz, $CDCl_3$) δ = 200.5 (C1), 189.4 (C4’), 177.6 (C2’), 136.4 (C10”), 131.8 (C14), 130.6 (C4), 129.8 (C5), 127.6 (C13), 117.3 (C2”), 117.2 (C11”), 98.1 (C3’), 66.6 (C5’), 66.4 (C8”), 60.7 (C6’), 54.4 (C6”), 49.3 (C2), 42.4 (C3), 40.7 (C7), 40.1 (C9”), 38.5 (C3”), 37.9 (C11), 36.9 (C5”), 35.4 (C9), 34.8 (C6), 34.3 (C7”), 28.5 (C8), 27.5 (C7’), 23.0 (C10), 22.7 (C16), 21.6 (C4”), 18.4 (C12), 18.2 (C15); HRMS (ESI+) *m*/*z* calcd for $C_{32}H_{45}NO_5Na$ $[M+Na]^+$ 546.3190, found 546.3197.

Compound 18b : *R*f = 0.71 (petroleum ether : AcOEt = 1 : 4); ^{1}H-NMR (400 MHz, $CDCl_3$) δ = 5.73–5.60 (m, 2H, C4, C10”), 5.48–5.43 (m, 2H, C13, C14), 5.39–5.37 (m, 1H, C5), 4.98–4.92 (m, 2H, C11”), 4.03 (dd, J = 10.3, 3.0 Hz, 1H, C6’a), 3.86–3.80 (m, 2H, C6’b, C8”a), 3.66–3.60 (m, 2H, C5’, C8”b), 3.37 (brs, 1H, C3), 3.04 (s, 3H, C7’), 2.57 (brd, J = 12.8 Hz, 1H, C11), 2.20 (brs, 1H, C6), 2.08–2.05 (m, 1H, C7”a), 1.99 (dd, J = 14.0, 7.1 Hz, 1H, C9”a), 1.91–1.82 (m, 2H, C3”a, C9”b), 1.68–1.65 (m, 1H, C10*eq*), 1.67 (brs, 3H, C15), 1.65–1.60 (m, 3H, C9*eq*, C3”b, C4”a), 1.57 (brs, 1H, C7*eq*), 1.55–1.50 (m, 5H, C4”b, C5”, C7”b), 1.48–1.41 (m, 1H, C10*ax*), 1.37–1.33 (m, 1H, C8), 1.31 (brs, 3H, C12), 1.07–1.00 (m, 1H, C7*ax*), 0.95–0.85 (m, 1H, C9*ax*), 0.80 (d, J = 6.4 Hz, 3H, C16); ^{13}C-NMR (100 MHz, $CDCl_3$) δ = 200.5 (C1), 189.4 (C4’), 177.6 (C2’), 136.1 (C10”), 131.8 (C14), 130.5 (C4), 129.9 (C5), 127.6 (C13), 117.8 (C2”), 117.4 (C11”), 98.3 (C3’), 66.3 (C5’, C8”), 59.9 (C6’), 54.3 (C6”), 49.3 (C2), 42.4 (C3), 41.0 (C7), 40.0 (C9”), 38.2 (C3”), 37.7 (C11), 37.4 (C5”), 35.4 (C9), 34.7 (C6), 34.6 (C7”), 28.4 (C8), 27.0 (C7’), 23.0 (C10), 22.7 (C16), 21.6 (C4”), 18.9 (C12), 18.2 (C15); HRMS (ESI+) *m*/*z* calcd for $C_{32}H_{45}NO_5Na$ $[M+Na]^+$ 546.3190, found 546.3193.

(3*Z*,5*S*)-3-{[(1*S*,2*S*,4a*R*,6*R*,8a*R*)-1,6-dimethyl-2-[(*E*)-prop-1-enyl]-4a,5,6,7,8,8a-hexahydro-2*H*-naphthalen-1-yl]-hydroxymethylidene}-5-(hydroxymethyl)-1-methylpyrrolidine-2,4-dione (1Ba)[11] [CAS 2095311-86-5]

To a solution of compound **17a** (14 mg, 26.7 μmol) in MeOH (1 mL) was added PPTS (1 mg, 4.0 μmol, 0.1 equiv.). The reaction mixture was refluxed for 3.0 h and poured into sat. $NaHCO_3$ aq. (20 mL). The aqueous layer was extracted with AcOEt (30 mL × 3), and the combined organic layers were washed with brine (30 mL), dried over Na_2SO_4, filtered, and evaporated. The residue was purified by preparative HPLC eluted with A / B (gradient 2 : 3 to 1 : 19, over 40 min) to afford compounds **1Ba** (4.0 mg, 40%). Spectral data for **1Ba**, **1Bb**, **2Ba** and **2Bb** are shown in Experimental in the text.

(3*Z*,5*R*)-3-{[(1*S*,2*S*,4a*R*,6*R*,8a*R*)-1,6-dimethyl-2-[(*E*)-prop-1-enyl]-4a,5,6,7,8,8a-hexahydro-2*H*-naphthalen-1-yl]-hydroxymethylidene}-5-(hydroxymethyl)-1-methylpyrrolidine-2,4-dione (1Bb)

Following the procedure described above with compound **17b** (19 mg, 36.3 μmol), **1Bb** was obtained (4 mg, 30%).

(3*Z*,5*S*)-3-{[(1*R*,2*R*,4a*S*,6*S*,8a*S*)-1,6-dimethyl-2-[(*E*)-prop-1-enyl]-4a,5,6,7,8,8a-hexahydro-2*H*-naphthalen-1-yl]-hydroxymethylidene}-5-(hydroxymethyl)-1-methylpyrrolidine-2,4-dione (2Ba)

Following the procedure described above with compound **18a** (29 mg, 55.4 μmol), **2Ba** was obtained (7.0 mg, 34%).

(3*Z*,5*R*)-3-{[(1*R*,2*R*,4a*S*,6*S*,8a*S*)-1,6-dimethyl-2-[(*E*)-prop-1-enyl]-4a,5,6,7,8,8a-hexahydro-2*H*-

naphthalen-1-yl]-hydroxymethylidene}-5-(hydroxymethyl)-1-methylpyrrolidine-2,4-dione (2Bb)
Following the procedure described above with compound **18b** (17 mg, 32.5 μmol), **2Bb** was obtained (7.0 mg, 58%).

1.3. Kinetic analysis

To a ^{t}BuOH solution of compound **15** (30 mM) was added ^{t}BuOK (2.0 equiv.), and the mixture was stirred at room temperature for 10 min to prepare **16**. The solution (final concentration = 300 μM) was diluted 100-fold by Tris - HCl buffer (20 mM, 10 mM NaCl, 10 mM EDTA, pH 7.5). To determine the kinetics of Fsa2, the assays were performed at 2.5 mL scale with 0.5 or 50 nM Fsa2 and 10–100 μM **16a** or **16b** at room temperature for 5.0 min. To determine the kinetics of Phm7, the assays were performed with 50 or 500 nM Phm7 and 10–100 μM **16a** or **16b** at room temperature for 5.0 min. The UV absorption spectra were acquired every 60 seconds. All assays were performed in triplicate. The resulting initial velocities were then fitted to the Michaelis-Menten equation using Kaleida Graph (Synergy Software) extract the parameters Km and Vmax.

1.4. Molecular Docking

Docking simulation was performed using crystal structure of inhibitor-bound form of Phm7 (PDB ID: 7E5V[12]) and **16a** [with conformation of the transition state TS_{1A} (Figure 3 in the main text) as the initial conformation] using BIOVIA Discovery Studio (DS) 2019. Before molecular docking, water molecules and co-crystallized inhibitor were deleted and then the protein was prepared using the Prepare Protein protocol. CDOCKER module of DS was used for this study. CDOCKER is a grid-based molecular docking method based on CHARMm[13]. The enzyme protein is kept rigid while the ligand is allowed to flex during the docking process.

2. Spectroscopic data

Table S1. Spectroscopic data of compound 1Ab: ^{1}H NMR spectrum (500 MHz) and ^{13}C NMR spectrum (125 MHz) in $CDCl_3$

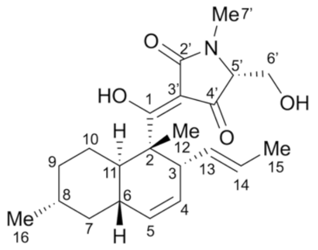

1Ab

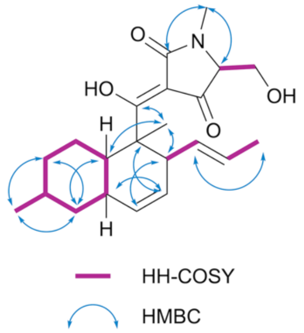

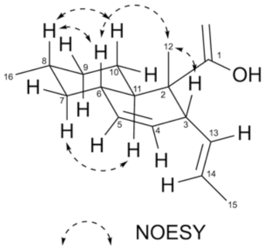

Position		δ_C	δ_H (J in Hz)	COSY
1		199.2	–	–
2		48.9	–	–
3		45.1	3.36 brs	H13
4		130.1	5.39 m	–
5		126.8	5.39 m	–
6		38.7	1.86 m	H7ax, H11
7	ax	42.4	0.88 m	H6
	eq		1.82 m	H8
8		33.7	1.49 m	H7eq, H9ax, H16
9	ax	35.8	1.11 brdd (12.0, 2.3)	H8, H10eq
	eq		1.76 m	H10ax
10	ax	28.5	1.04 m	H9eq, H11
	eq		1.99 m	H9ax
11		40.1	1.67 m	H6, H10ax
12		14.2	1.47 brs [3H]	–
13		131.0	5.16 m	H3
14		127.2	5.25 m	H15
15		18.1	1.53 d (5.7) [3H]	H14
16		22.6	0.92 d (6.3) [3H]	H8
2’		177.2	–	–
3’		100.4	–	–
4’		191.0	–	–
5’		66.3	3.67 brs	H6’a, H6’b
6’	a	60.4	4.06 brd (11.5)	H5’
	b		3.84 dd (5.0, 11.5)	H5’
7’		27.3	3.05 s [3H]	–

Table S2. Spectroscopic data of compound 1Ba: ^{1}H NMR spectrum (600 MHz) and ^{13}C NMR spectrum (150 MHz) in $CDCl_3$

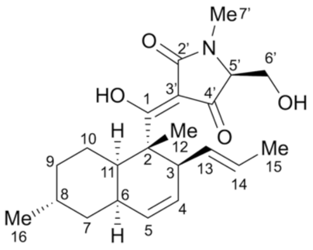

1Ba

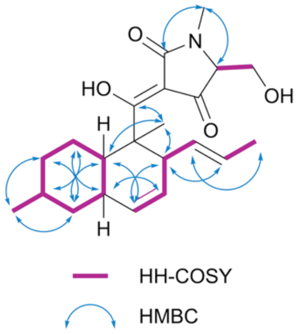

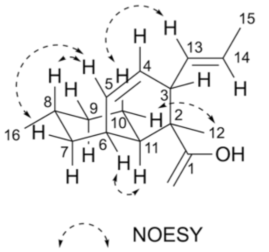

Position		δC	δH (J in Hz)	COSY
1		201.7	–	–
2		49.5	–	–
3		42.5	3.35 brs	H4, H13
4		130.4	5.62 m	H3, H5
5		129.9	5.40 m	H4
6		34.7	2.16 m	H7ax, H7eq, H11
7	ax	40.7	1.06 m	H6, H8
	eq		1.60 m	H6
8		28.4	1.35 m	H7ax, H9ax, H9eq, H16
9	ax	35.3	0.90 m	H8, H10ax, H10eq
	eq		1.65 m	H10ax
10	ax	22.9	1.44 dd (23.0, 13.9)	H9ax, H9eq, H11
	eq		1.65 m	H9ax, H11
11		37.9	2.52 brd (9.8)	H6, H10ax, H10eq
12		18.6	1.33 brs [3H]	–
13		127.8	5.46 m	H3
14		131.5	5.46 m	H15
15		18.2	1.69 d (7.2) [3H]	H14
16		22.7	0.82 d (6.4) [3H]	H8
2’		177.8	–	–
3’		97.8	–	–
4’		190.5	–	–
5’		66.6	3.69 brs	H6’a, H6’b
6’	a	60.3	4.07 brd (7.5)	H5’
	b		3.89 brd (7.5)	H5’
7’		27.5	3.07 s [3H]	–

Table S3. Spectroscopic data of compound 1Bb: ^{1}H NMR spectrum (600 MHz) and ^{13}C NMR spectrum (150 MHz) in $CDCl_3$

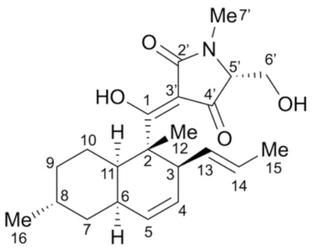

1Bb

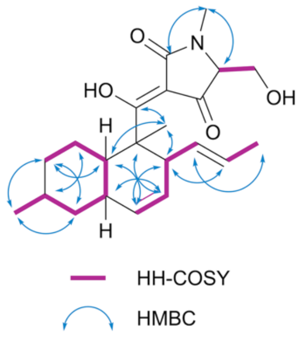

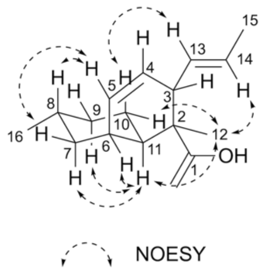

Position		δ_C	δ_H (J in Hz)	COSY
1		202.0	–	–
2		49.6	–	–
3		42.4	3.35 brs	H4, H13
4		130.5	5.62 brd (9.6)	H3, H5
5		129.8	5.39 brd (9.6)	H4
6		34.8	2.16 brs	H7ax, H11
7	ax	40.7	1.11 m	H6, H8
	eq		1.60 brd (12.4)	–
8		28.4	1.34 m	H7ax, H16
9	ax	35.4	0.90 dd (23.5, 12.0)	H10ax, H10eq
	eq		1.66 m	H10ax
10	ax	22.9	1.44 dd (23.7, 12.0)	H9ax, H9eq, H11
	eq		1.66 m	H9ax
11		37.9	2.57 brd (4.9)	H6, H10ax
12		18.5	1.31 brs [3H]	–
13		127.8	5.44 m	H3
14		131.5	5.44 m	H15
15		18.2	1.68 d (3.6) [3H]	H14
16		22.7	0.82 d (6.0) [3H]	H8
2’		177.8	–	–
3’		97.6	–	–
4’		190.4	–	–
5’		66.7	3.70 brs	H6’a, H6’b
6’	a	60.3	4.05 brd (8.4)	H5’
	b		3.88 brd (8.4)	H5’
7’		27.6	3.07 s [3H]	–

Table S4. Spectroscopic data of compound 2Aa**:** ^{1}H NMR spectrum (500 MHz) and ^{13}C NMR spectrum (125 MHz) in $CDCl_3$

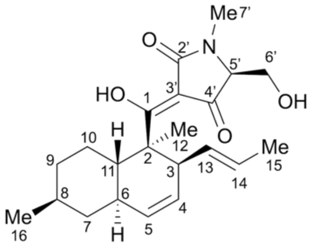

2Aa

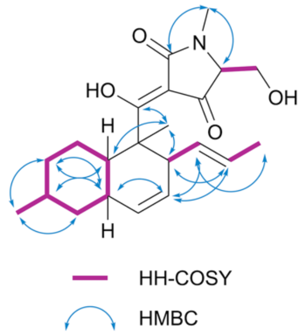

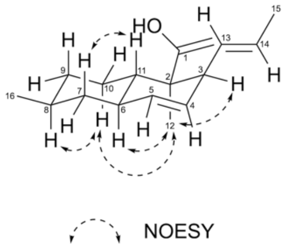

Position		δ_C	δ_H (J in Hz)	COSY
1		199.1	–	–
2		48.9	–	–
3		45.1	3.35 brs	H13
4		130.1	5.40 m	–
5		126.8	5.40 m	–
6		38.7	1.86 m	H7ax, H11
7	ax	42.4	0.85 m	H6
	eq		1.81 m	H8
8		33.7	1.50 m	H7eq, H9ax, H16
9	ax	35.8	1.13 m	H8, H10eq
	eq		1.76 m	H10ax
10	ax	28.5	1.05 m	H9eq, H11
	eq		1.98 m	H9ax
11		40.1	1.68 m	H6, H10ax
12		14.2	1.47 brs [3H]	–
13		131.0	5.16 m	H3
14		127.2	5.24 m	H15
15		18.0	1.53 d (6.0) [3H]	H14
16		22.6	0.92 d (6.0) [3H]	H8
2’		177.2	–	–
3’		100.4	–	–
4’		190.9	–	–
5’		66.3	3.67 brs	H6’a, H6’b
6’	a	60.4	4.06 brd (11.5)	H5’
	b		3.84 dd (11.5, 4.6)	H5’
7’		27.3	3.05 s [3H]	–

Table S5. Spectroscopic data of compound 2Ab: ^{1}H NMR spectrum (600 MHz) and ^{13}C NMR spectrum (125 MHz) in $CDCl_3$

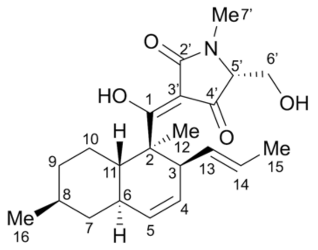

2Ab

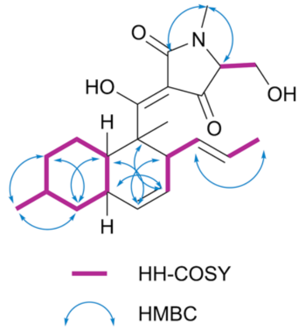

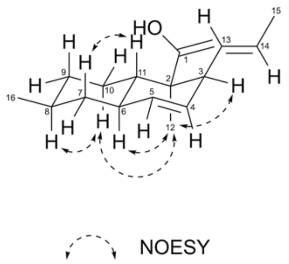

Position		δ_C	δ_H (J in Hz)	COSY
1		199.2	–	–
2		48.8	–	–
3		45.1	3.34 brs	H4, H13
4		130.1	5.41 m	H3
5		126.7	5.41 m	–
6		38.7	1.86 m	H7ax, H11
7	ax	42.4	0.85 m	H6, H8
	eq		1.82 m	–
8		33.6	1.51 m	H7ax, H9ax, H16
9	ax	35.8	1.12 dd (24.0, 12.0)	H8, H10ax
	eq		1.76 m	–
10	ax	28.4	1.05 dd (24.0, 12.0)	H9ax, H11
	eq		1.97 m	–
11		40.0	1.67 m	H6, H10ax
12		14.1	1.46 brs [3H]	–
13		131.0	5.15 m	H3
14		127.2	5.26 m	H15
15		18.1	1.55 brs [3H]	H14
16		22.6	0.92 d (6.0) [3H]	H8
2’		177.3	–	–
3’		100.1	–	–
4’		190.7	–	–
5’		66.8	3.64 brs	H6’a, H6’b
6’	a	60.6	4.03 dd (11.4, 2.4)	H5’
	b		3.89 brd (11.4)	H5’
7’		27.5	3.06 s [3H]	–

***Table S6. Spectroscopic data of compound 2Ba*:** ^{1}H NMR spectrum (500 MHz) and ^{13}C NMR spectrum (125 MHz) in $CDCl_3$

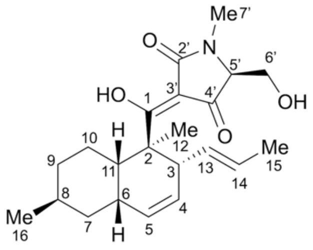

2Ba

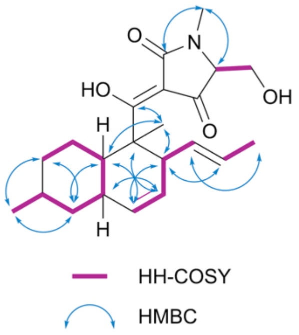

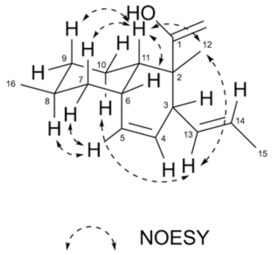

Position		δC	δH (J in Hz)	COSY
1		202.0	–	–
2		49.6	–	–
3		42.4	3.36 brs	H4, H13
4		130.6	5.62 m	H3, H5
5		129.7	5.39 brd (9.5)	H4
6		34.9	2.16 brs	H7ax, H7eq, H11
7	ax	40.7	1.11 ddd (12.5, 12.5, 4.5)	H6, H8
	eq		1.60 brd (12.5)	H6
8		28.4	1.36 m	H7ax, H16
9	ax	35.4	0.89 m	H10ax, H10eq
	eq		1.65 m	H10ax
10	ax	23.0	1.44 ddd (25.3, 13.0, 3.3)	H9ax, H9eq, H11
	eq		1.66 m	H9ax, H11
11		38.0	2.58 brd (9.5)	H6, H10ax, H10eq
12		18.5	1.31 brs [3H]	–
13		127.8	5.46 m	H3
14		131.6	5.46 m	H15
15		18.2	1.68 d (5.5) [3H]	H14
16		22.7	0.82 d (6.0) [3H]	H8
2’		178.0	–	–
3’		97.7	–	–
4’		190.4	–	–
5’		66.7	3.70 brs	H6’a, H6’b
6’	a	60.5	4.04 dd (11, 3.0)	H5’
	b		3.88 dd (11, 4.5)	H5’
7’		27.6	3.07 s [3H]	–

Table S7. Spectroscopic data of compound 2Bb: ^{1}H NMR spectrum (600 MHz) and ^{13}C NMR spectrum (125 MHz) in $CDCl_3$

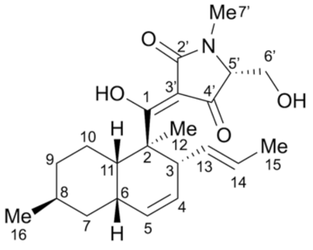

2Bb

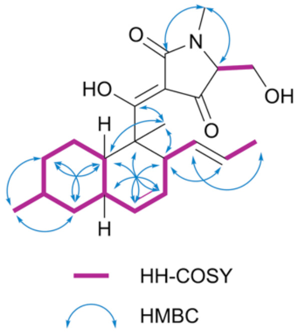

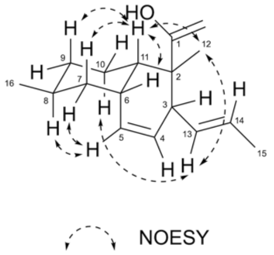

Position		δC	δH (J in Hz)	COSY
1		201.7	–	–
2		49.6	–	–
3		42.5	3.36 brs	H4, H13
4		130.5	5.62 m	H3, H5
5		129.8	5.39 brd (9.6)	H4, H6
6		34.8	2.18 brs	H5, H7ax, H7eq, H11
7	ax	40.7	1.07 m	H6, H8
	eq		1.60 brd (13.2)	H6
8		28.4	1.35 m	H7ax, H9ax, H16
9	ax	35.4	0.90 m	H8, H10ax, H10eq
	eq		1.68 m	H10ax
10	ax	22.9	1.44 m	H9ax, H9eq, H11
	eq		1.65 m	H9ax, H11
11		38.0	2.52 brd (11.4)	H6, H10ax, H10eq
12		18.6	1.33 brs [3H]	–
13		127.7	5.46 m	H3
14		131.6	5.46 m	H15
15		18.2	1.68 d (4.2) [3H]	H14
16		22.6	0.82 d (7.2) [3H]	H8
2’		177.9	–	–
3’		97.8	–	–
4’		190.4	–	–
5’		66.7	3.68 brs	H6’a, H6’b
6’	a	60.4	4.06 brd (10.2)	H5’
	b		3.89 brd (10.2)	H5’
7’		27.5	3.06 s [3H]	–

***Table S8. Spectroscopic data of compound 2Ca*:** ^{1}H NMR spectrum (600 MHz) and ^{13}C NMR spectrum (150 MHz) in $CDCl_3$

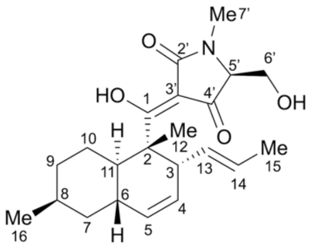

2Ca

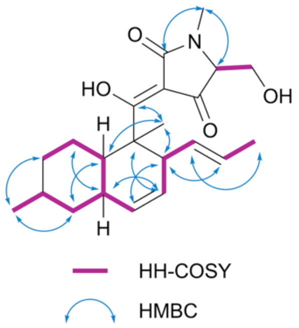

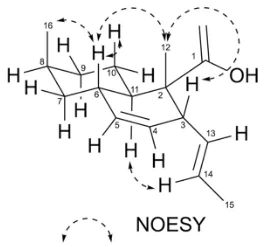

Position		δ$_C$	δ$_H$ (*J* in Hz)	COSY
1		199.3	–	–
2		48.9	–	–
3		45.0	3.34 brs	H4, H13
4		127.0	5.42 m	H3
5		130.4	5.35 m	H6
6		32.6	2.04 m	H5, H7ax, H7eq, H11
7	ax	39.6	1.44 m	H6, H8
	eq		1.59 brd (12.6)	H6
8		27.9	2.07 m	H7ax, H16
9	ax	32.6	1.51 m	H10ax, H10eq
	eq		1.72 m	H10eq
10	ax	23.0	1.23 dd (23.0, 11.3)	H9ax, H11
	eq		1.75 m	H9eq
11		41.1	1.68 m	H6, H10ax
12		14.2	1.48 brs [3H]	–
13		131.1	5.20 m	H3
14		127.2	5.26 m	H15
15		18.1	1.55 brs [3H]	H14
16		19.0	1.02 d (6.4) [3H]	H8
2'		177.3	–	–
3'		100.1	–	–
4'		190.7	–	–
5'		66.8	3.64 brs	H6'a, H6'b
6'	a	60.6	4.04 brd (10.2)	H5'
	b		3.88 brd (10.2)	H5'
7'		27.5	3.06 s [3H]	–

Table S9. Spectroscopic data of compound 17a and 17b: ^{1}H NMR spectrum (600 MHz) and ^{13}C NMR spectrum (100 MHz) in $CDCl_3$

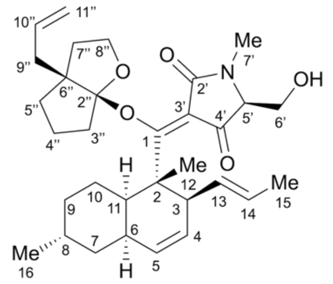

17a

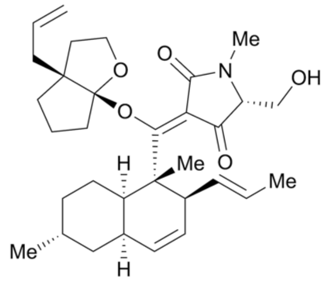

17b

Position		**17a**		**17b**	
		δ_C	δ_H (J in Hz)	δ_C	δ_H (J in Hz)
1		200.7	–	200.4	–
2		49.3	–	49.3	–
3		42.5	3.34 brs	42.3	3.35 brs
4		130.6	5.65 m	130.6	5.63 m
5		129.8	5.40 m	129.8	5.41 m
6		34.6	2.18 brs	34.8	2.19 brs
7	ax	40.9	1.02 m	40.7	1.11 m
	eq		1.59 m		1.61 m
8		28.3	1.35 m	28.5	1.33 m
9	ax	35.5	0.86 m	35.4	0.90 m
	eq		1.66 m		1.66 m
10	ax	23.0	1.42 m	23.0	1.43 m
	eq		1.67 m		1.68 m
11		37.9	2.53 brd (12.0)	37.9	2.66 brd (10.5)
12		18.7	1.33 brs [3H]	18.4	1.27 brs [3H]
13		127.6	5.47 m	127.6	5.45 m
14		131.8	5.47 m	131.7	5.44 m
15		18.2	1.68 brs [3H]	18.2	1.68 brs [3H]
16		22.7	0.80 d (6.4) [3H]	22.7	0.81 brs [3H]
2’		177.5	–	177.6	–
3’		98.0	–	98.4	–
4’		188.9	–	189.8	–
5’		66.4	3.73 brs	66.5	3.69 brs
6’	a	60.9	4.02 dd (10.2, 4.1)	60.0	4.03 brd (9.0)
	b		3.79 m		3.82 m
7’		27.7	3.06 s [3H]	27.0	3.06 s [3H]
2”		117.4	–	117.8	–
3”	a	38.7	1.87 m	38.4	1.86 m
	b		1.68 m		1.66 m
4”	a	21.6	1.60 m	21.6	1.61 m
	b		1.60 m		1.50 brs overlap
5”	a	36.9	1.59 m	37.4	1.50 brs overlap
	b		1.51 m		1.50 brs overlap
6”		54.3	–	54.3	–
7”	a	34.5	2.12 dd (11.9, 4.7)	34.6	2.02 m
	b		1.46 m		1.50 brs overlap
8”	a	66.3	3.85 m	66.3	3.82 m
	b		3.65 m		3.59 m
9”	a	40.1	2.02 dd (13.1, 7.2)	40.2	1.98 m
	b		1.97 dd (13.1, 7.2)		1.86 m
10”		136.2	5.71 m	136.4	5.68 m
11”		117.3	4.99 m [2H]	117.1	4.95 m [2H]

Table S10. Spectroscopic data of compound 18a and 18b: ^{1}H NMR spectrum (600 MHz or 400 MHz) and ^{13}C NMR spectrum (100 MHz) in $CDCl_3$

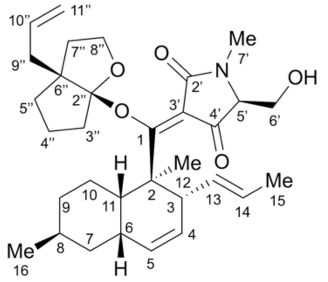

18a

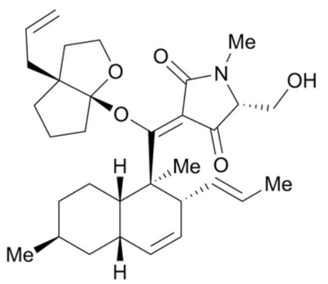

18b

Position		18a		18b	
		δ_C	δ_H (*J* in Hz)	δ_C	δ_H (*J* in Hz)
1		200.5	–	200.5	–
2		49.3	–	49.3	–
3		42.4	3.37 brs	42.4	3.37 brs
4		130.6	5.62 m	130.5	5.64 m
5		129.8	5.40 m	129.9	5.38 m
6		34.8	2.19 brs	34.7	2.20 brs
7	ax	40.7	1.11 m	41.0	1.04 m
	eq		1.58 m		1.57 brs
8		28.5	1.33 m	28.4	1.35 m
9	ax	35.4	0.90 m	35.4	0.90 m
	eq		1.67 m		1.64 m
10	ax	23.0	1.45 m	23.0	1.45 m
	eq		1.67 m		1.67 m
11		37.9	2.64 dt (12.4, 3.5)	37.7	2.57 brd (12.8)
12		18.4	1.29 brs [3H]	18.9	1.31 brs [3H]
13		127.6	5.45 m	127.6	5.47 m
14		131.8	5.45 m	131.8	5.47 m
15		18.2	1.67 brs [3H]	18.2	1.67 brs [3H]
16		22.7	0.81 d (6.5) [3H]	22.7	0.80 d (6.4) [3H]
2'		177.6	–	177.6	–
3'		98.1	–	98.3	–
4'		189.4	–	189.4	–
5'		66.6	3.73 dd (4.0, 2.6)	66.3	3.65 m
6'	a	60.7	4.00 dd (10.1, 4.0)	59.9	4.03 dd (10.3, 3.0)
	b		3.75 dd (10.1, 2.6)		3.85 m
7'		27.5	3.05 s [3H]	27.0	3.04 s [3H]
2"		117.3	–	117.8	–
3"	a	38.5	1.81 ddd (12.2, 6.8, 3.7)	38.2	1.88 m
	b		1.66 m		1.65 m
4"	a	21.6	1.60 m	21.6	1.63 m
	b		1.60 m		1.54 m
5"	a	36.9	1.57 m	37.4	1.52 m
	b		1.57 m		1.52 m
6"		54.4	–	54.3	–
7"	a	34.3	2.09 m	34.6	2.07 m
	b		1.45 m		1.52 m
8"	a	66.4	3.81 td (8.2, 3.7)	66.3	3.82 m
	b		3.62 m		3.62 m
9"	a	40.1	2.03 dd (13.8, 7.2)	40.0	1.99 dd (14.0, 7.1)
	b		1.96 dd (13.8, 7.2)		1.88 m
10"		136.4	5.72 m	136.1	5.67 m
11"		117.2	4.99 m [2H]	117.4	4.95 m [2H]

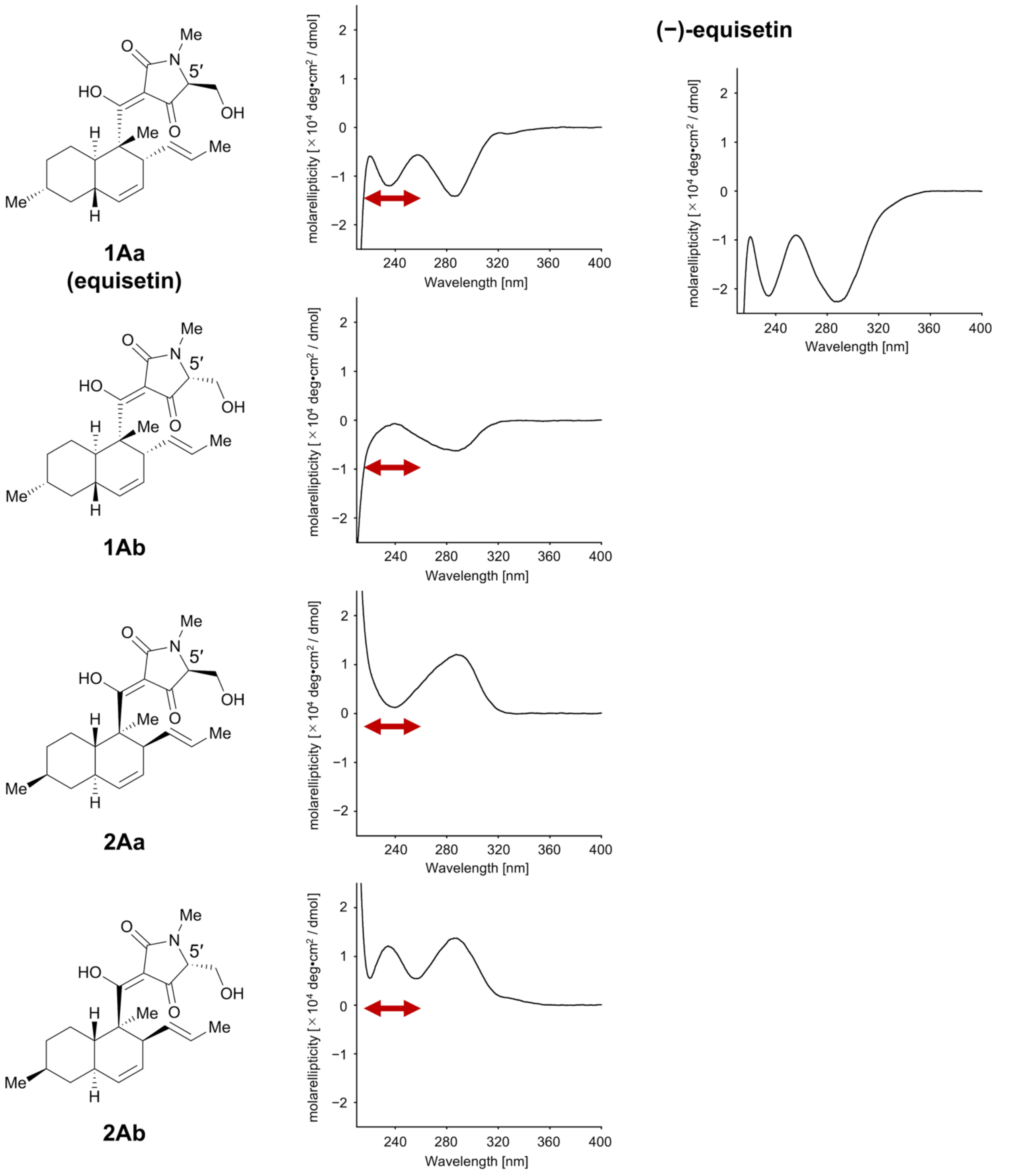

***Figure S1. Structures and ECD spectra of trans−decalins 1Aa, 1Ab, 2Aa and 2Ab*:** All spectra were obtained using 1.94 mM sample solution in MeOH. Equisetin (Cayman Chemical) was used as the authentic sample to identify the resulting decalins. The peak around 230 nm (range indicated by red arrow) was significantly different between **1Aa** and **1Ab**, or **2Aa** and **2Ab**, which have opposite absolute configurations at the 5′ position.

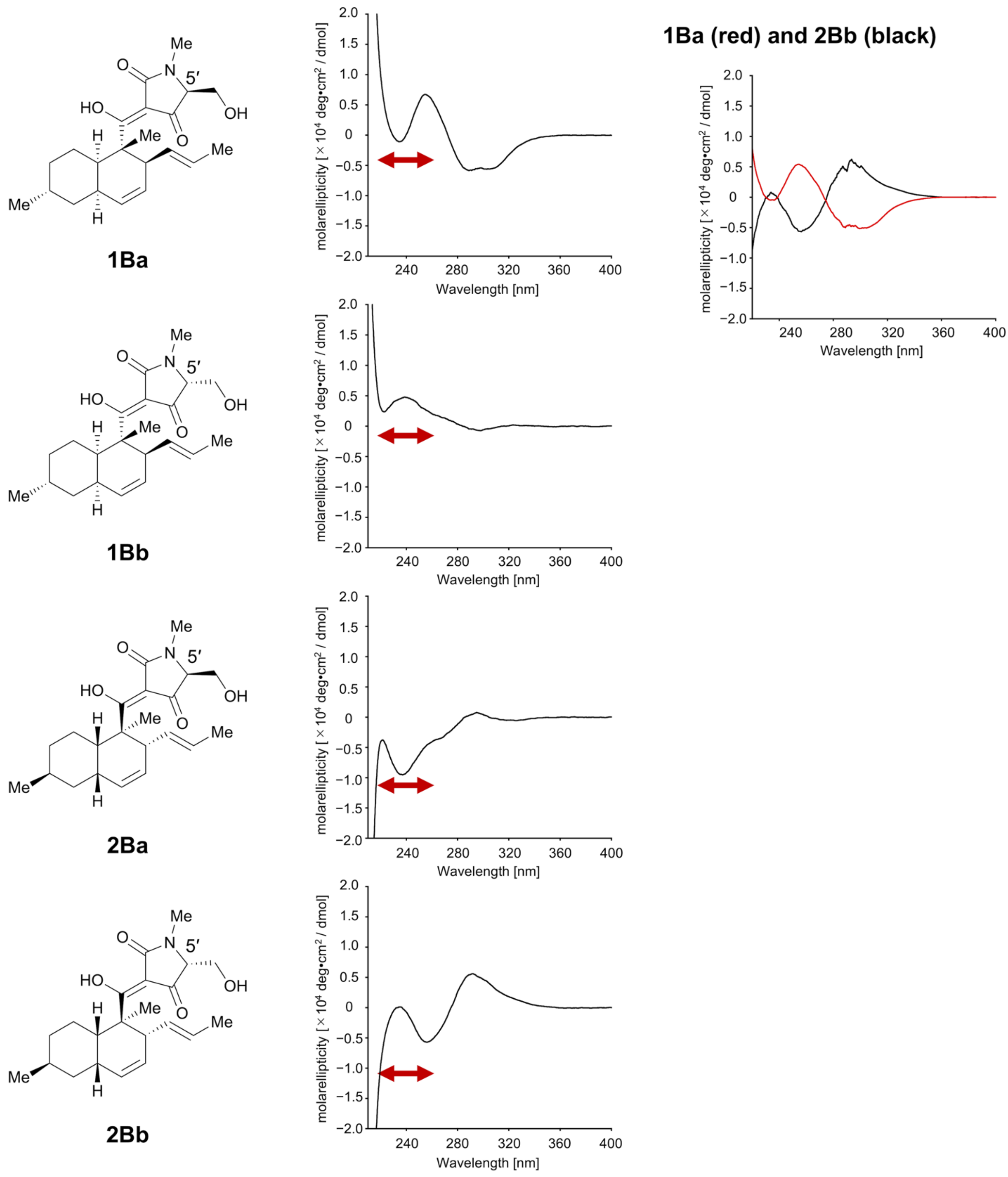

***Figure S2. Structures and ECD spectra of cis-decalins 1Ba, 1Bb, 2Ba and 2Bb*:** All spectra were obtained using 1.94 mM sample solution in MeOH. The peak around 230 nm (range indicated by red arrow) was significantly different between **1Ba** and **1Bb**, or **2Ba** and **2Bb**, which have opposite absolute configurations at the 5′ position. ECD spectra of 4.85 mM MeOH solutions of **1Ba** (red) or **2Bb** (black) are overlaid.

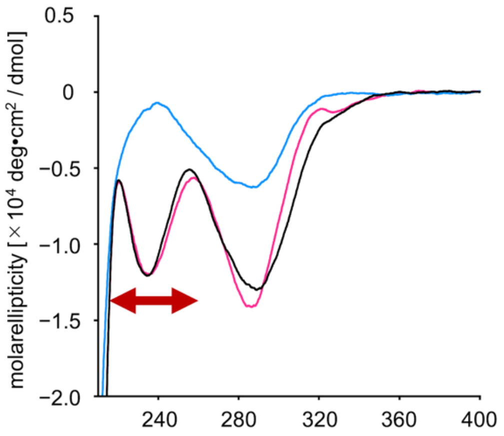

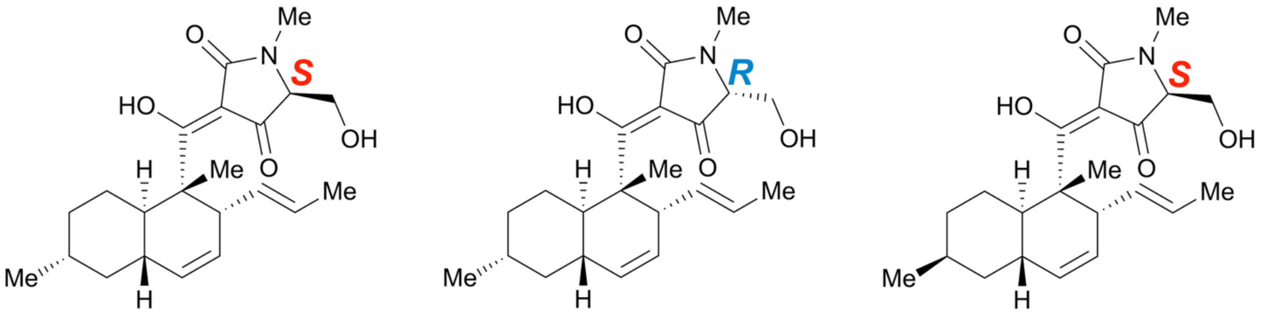

***Figure S3. Overlaid ECD spectra of 1Aa, 1Ab and the new diastereomer 2C*a:** All spectra were obtained using 1.94 mM sample solution in MeOH. The absolute configuration of the 5′ position of **2Ca** was determined as *S* based on the spectral coincidence around 230 nm.

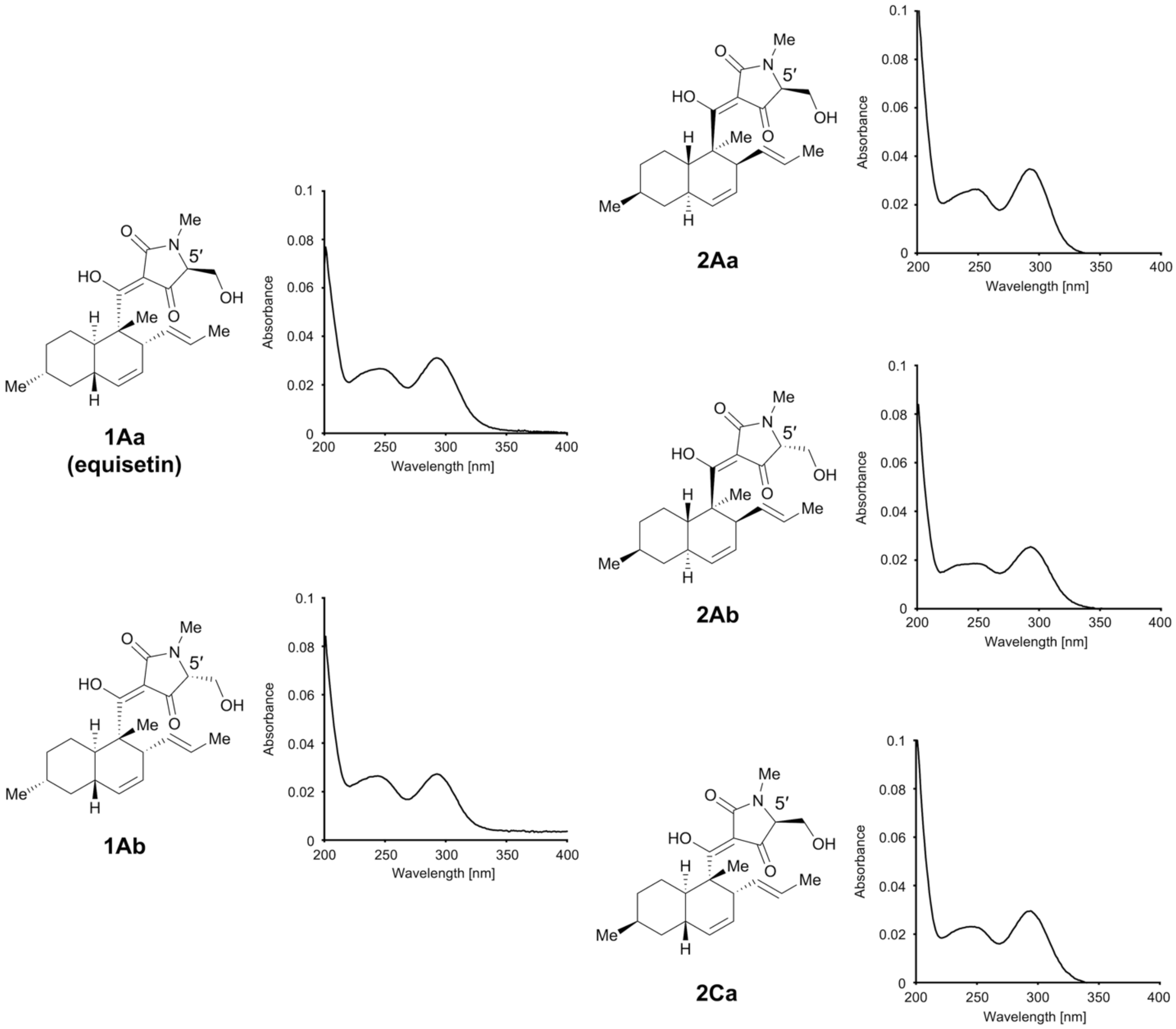

Figure S4. Structures and absorption spectra of trans−decalins 1Aa, 1Ab, 2Aa, 2Ab and 2Ca: All spectra were obtained using 5.0 μM sample solution in MeOH. The path length was 1.0 cm with a cell volume of 3.0 mL.

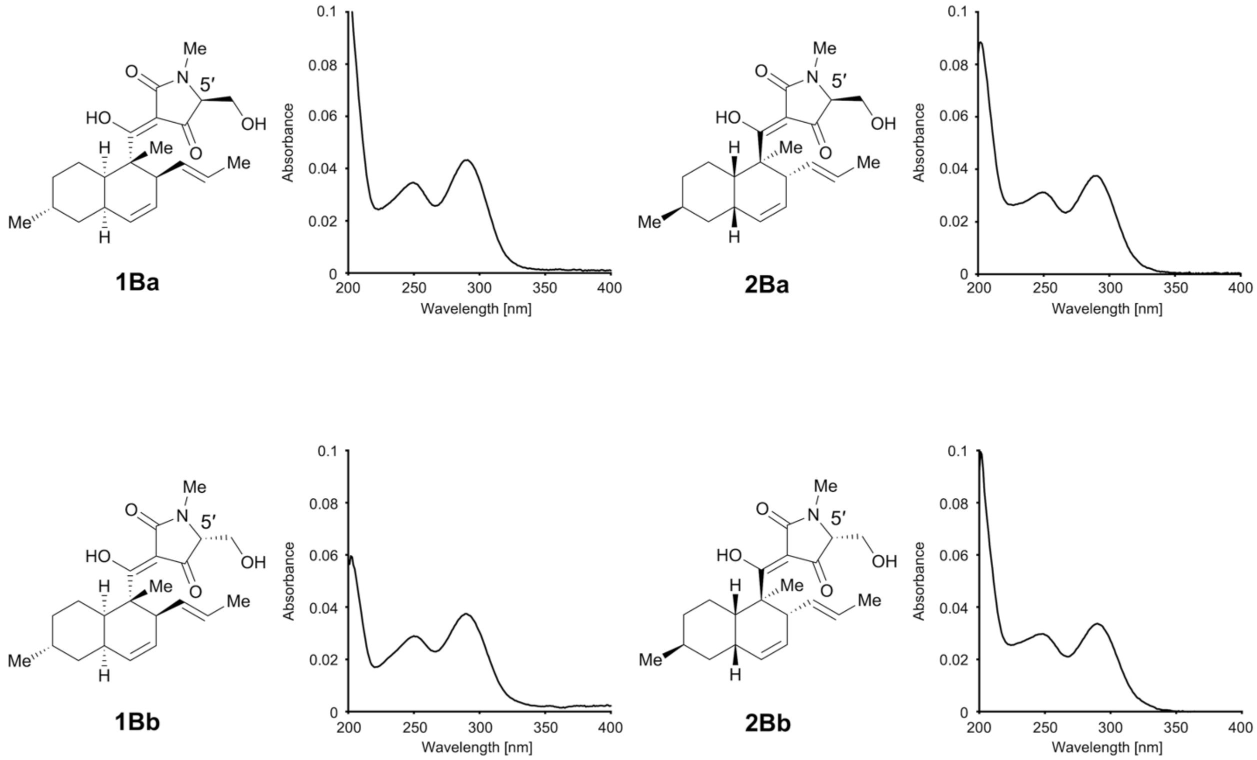

***Figure S5 Structures and absorption spectra of cis-decalins 1Ba, 1Bb, 2Ba and 2Bb*:** All spectra were obtained using 5.0 µM sample solution in MeOH. The path length was 1.0 cm with a cell volume of 3.0 mL.

3. UPLC analyses of compounds and reactions

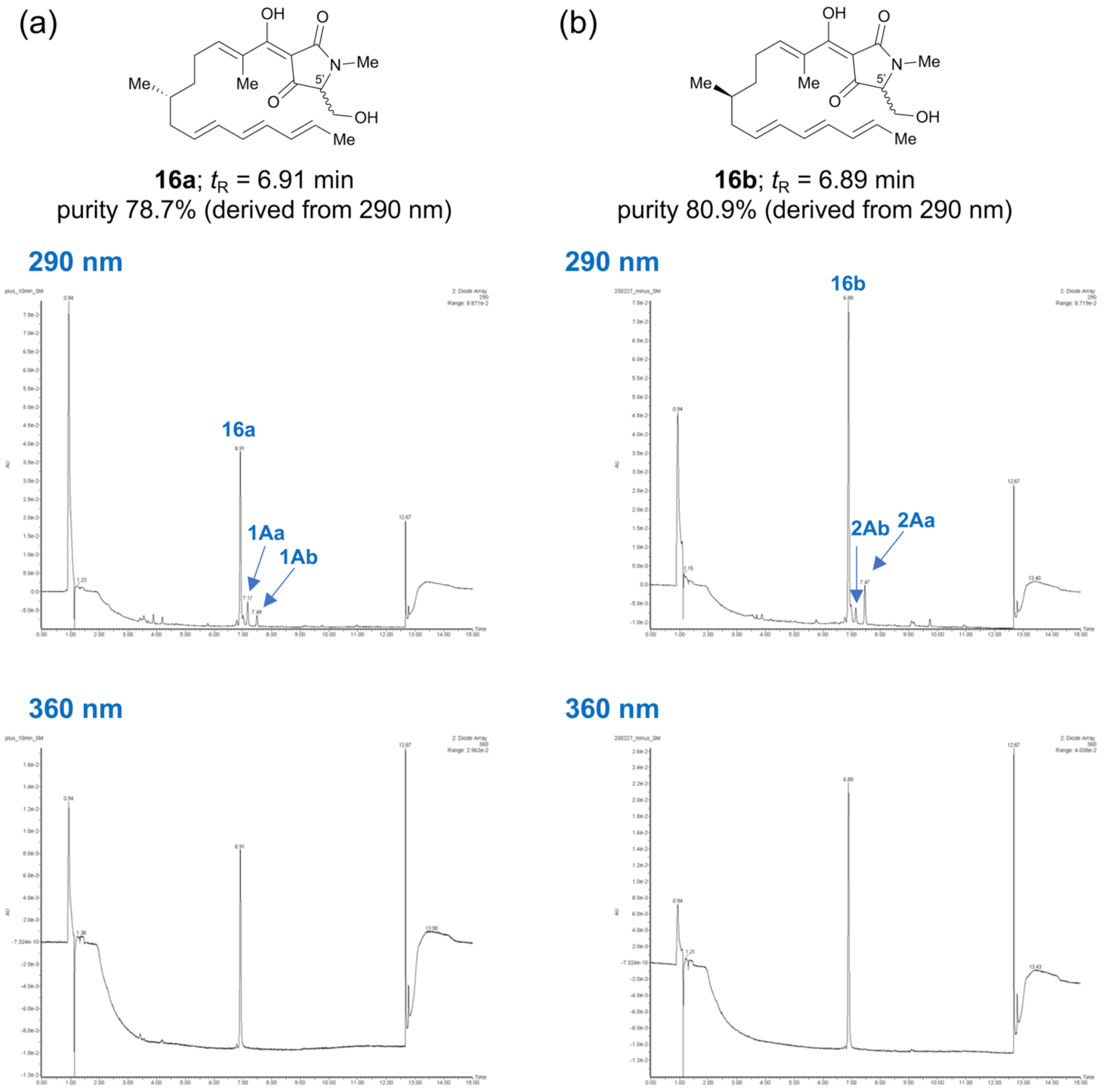

Figure S6. UPLC analysis of tetramic acid-bearing polyenes 16a (a) and 16b (b): As described in the Experimental section, **15a** and **15b** were treated with base to prepare **16a** and **16b**, which were subjected to UPLC and detected at 290 and 360 nm. The characteristic absorption maxima for tetramic acids are 290 nm and for linear polyenes are 360 nm, respectively. Purity was determined from the peak area ratio of the chromatogram detected at 290 nm.

a)

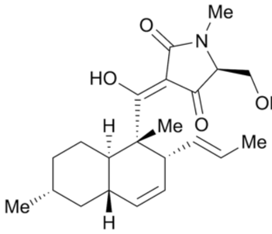

1Aa; t_R = 7.16 min
purity 97%

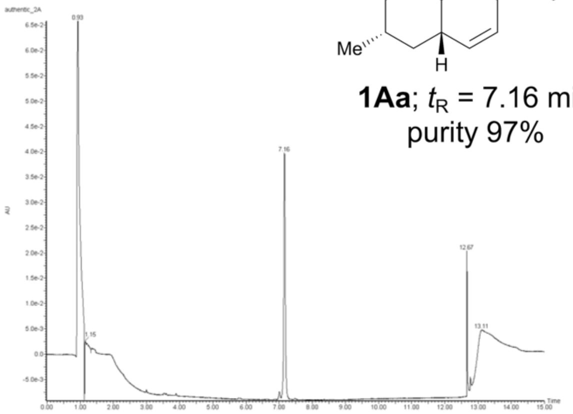

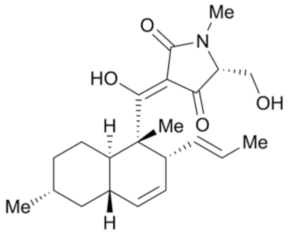

1Ab; t_R = 7.48 min
purity 91%

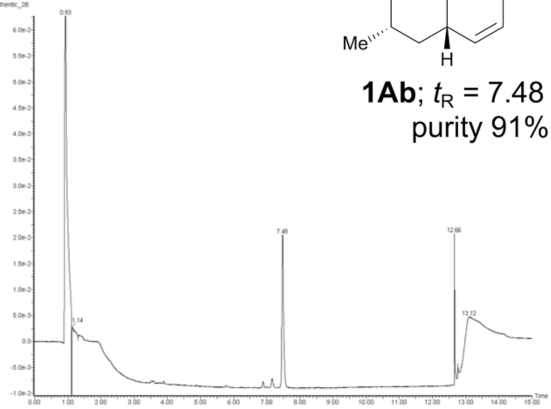

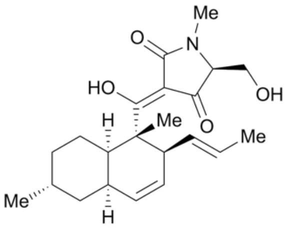

1Ba; t_R = 7.00 min
purity 98%

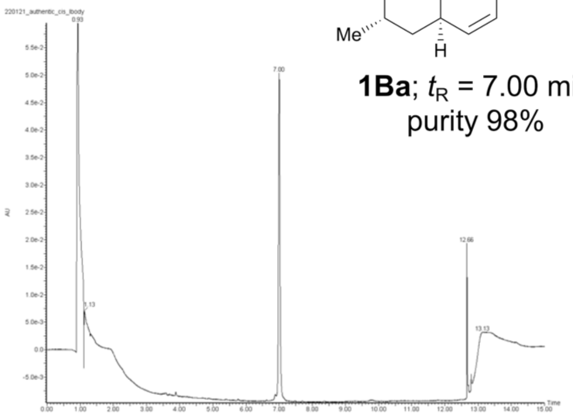

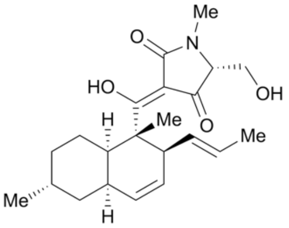

1Bb; t_R = 6.99 min
purity 97%

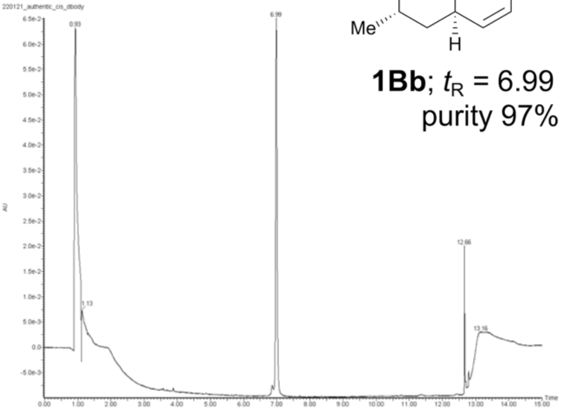

b)

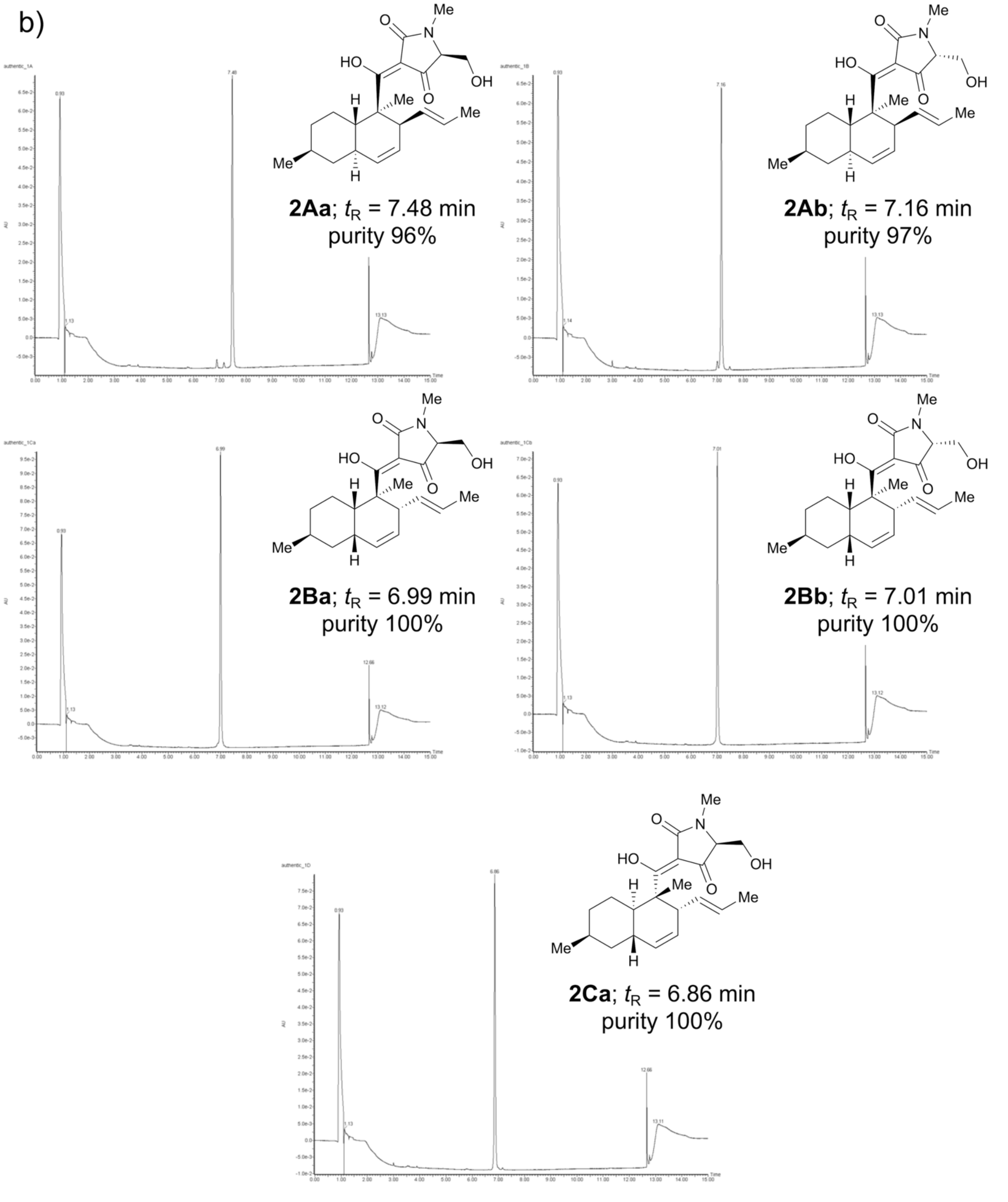

Figure S7. UPLC analysis of the 8S decalins (a) 1Aa, 1Ab, 1Ba, 1Bb and 8R decalins (b) 2Aa, 2Ab, 2Ba, 2Bb and 2Ca: Purity was determined from the peak area ratio of the chromatogram detected at 290 nm.

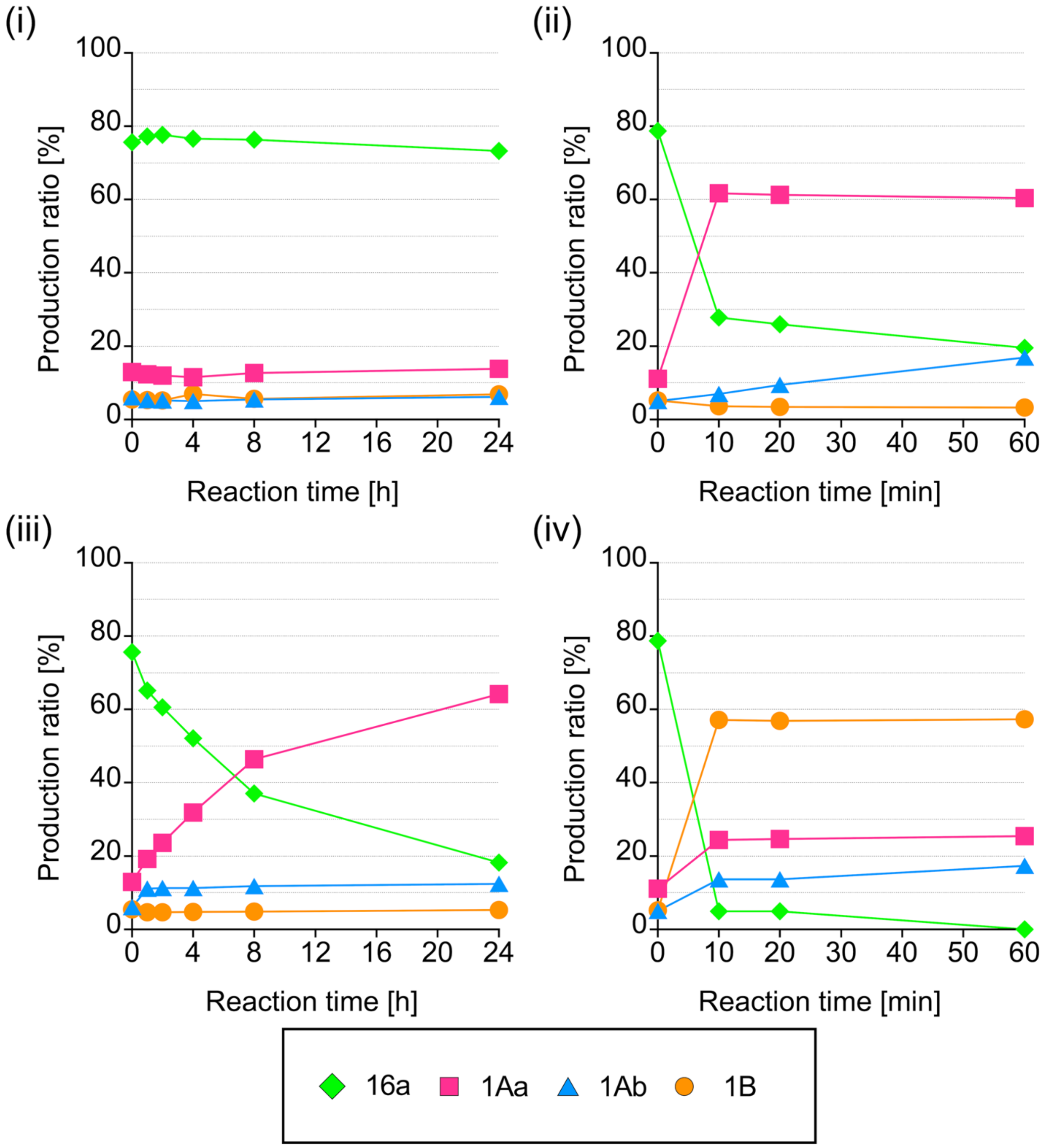

Figure S8. Time-course of IMDA reactions of precursor 16a**:** Precursor **16a** was incubated under various conditions: (i) in Tris-HCl buffer (pH 7,5) without enzyme at 25 °C; (ii) with 0.5 μM Fsa2 at 25 °C; (iii) with 5.0 μM Phm7 at 25 °C; (iv) reflux in toluene. The production ratio was calculated from peak areas of **16a** (green diamond), **1Aa** (red square), **1Ab** (blue triangle) and **1B** (orange circle) by UPLC analysis.

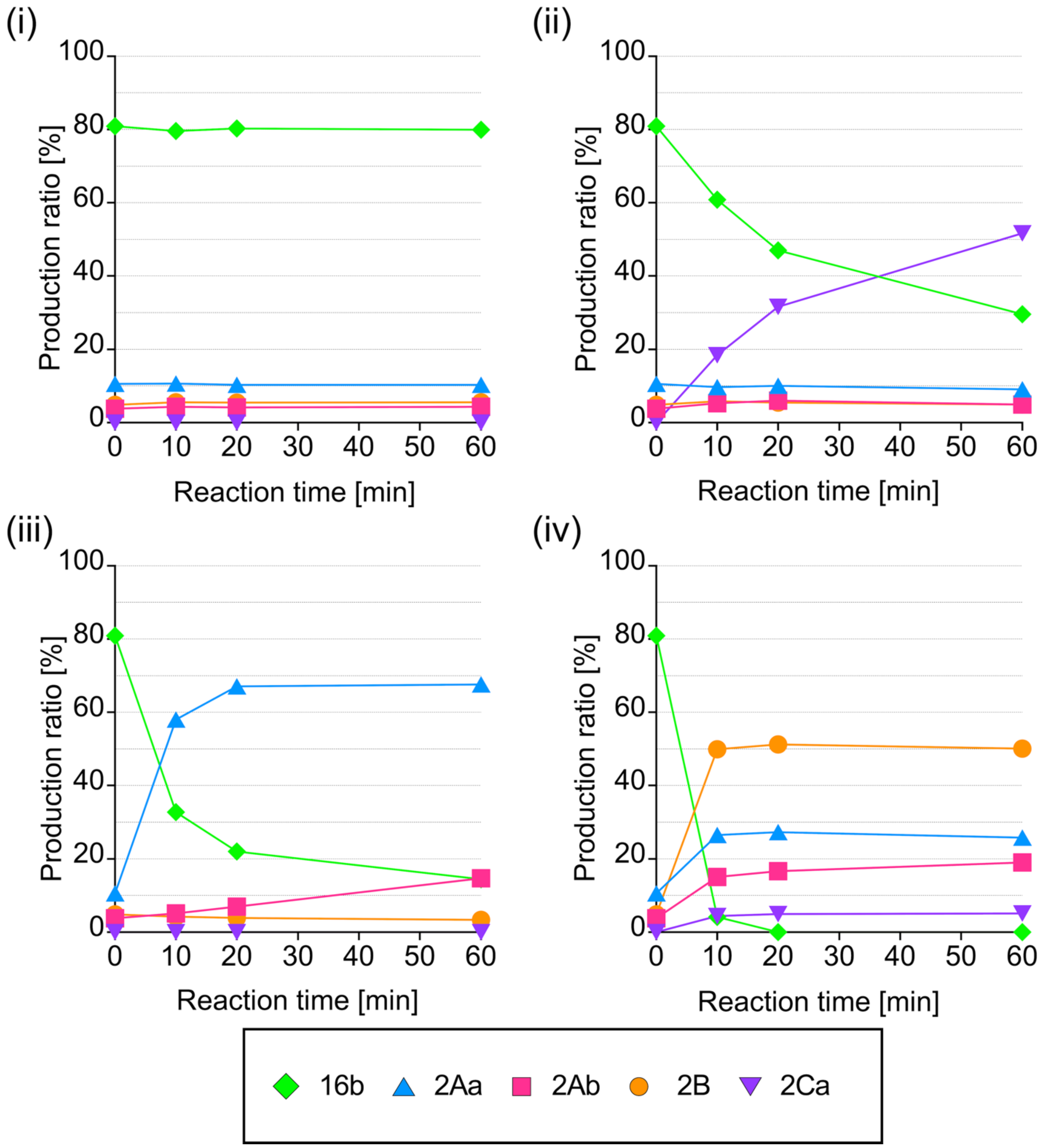

***Figure S9. Time-course of IMDA reactions of precursor 16b*:** Precursor **16b** was incubated under various conditions: (i) in Tris-HCl buffer (pH 7,5) without enzyme at 25 °C; (ii) with 0.5 µM Fsa2 at 25 °C; (iii) with 5.0 µM Phm7 at 25 °C; (iv) reflux in toluene. The production ratio was calculated from peak areas of **16b** (green diamond), **2Aa** (blue triangle), **2Ab** (red square), **2B** (orange circle) and **2Ca** (purple inverted triangle). by UPLC analysis.

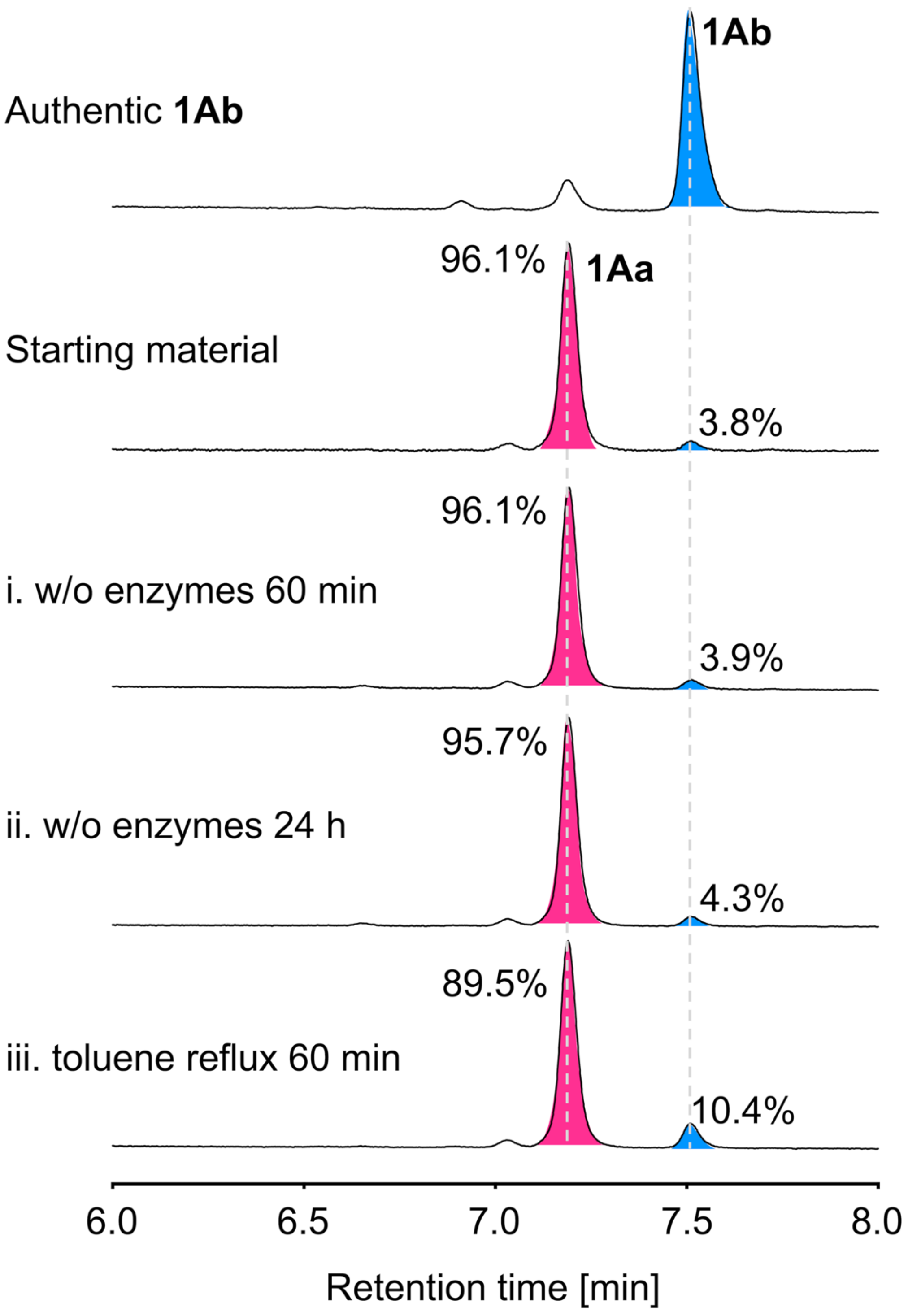

Figure S10. UPLC analysis of thermodynamic isomerization of decalin 1Aa: The substrate solution was incubated under various conditions: **i**) in Tris-HCl buffer without enzyme at 25 °C for 60 min; **ii**) in Tris-HCl buffer without enzyme at 25 °C for 24 h; **iii**) reflux in toluene for 60 min. Diastereomeric ratios were calculated from the integrated peak areas of each chart.

4. Kinetic analyses of IMDA reaction catalyzed by Fsa2 and Phm7

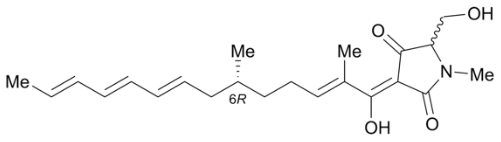

16a
(pro-equisetin)

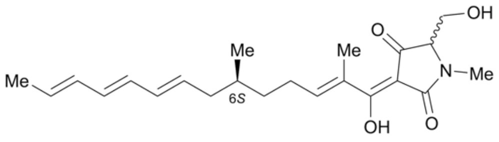

16b
(epi-pro-equisetin)

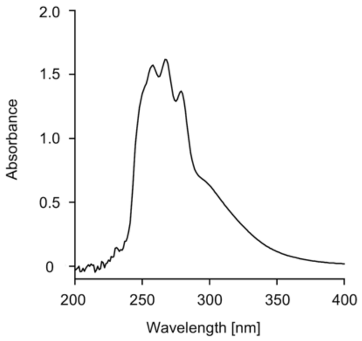

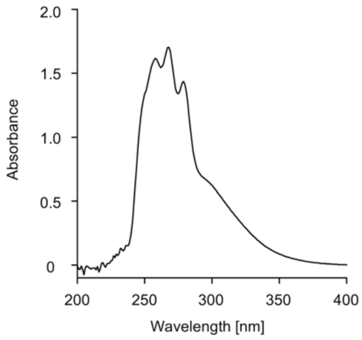

***Figure S11. Structures and absorption spectra of precursors 16a and 16b*:** All spectra were obtained using 100 µM sample solution in 1% ^{t}BuOH/Tris - HCl buffer (pH 7.5). The path length was 1.0 cm with a cell volume of 3.0 mL.

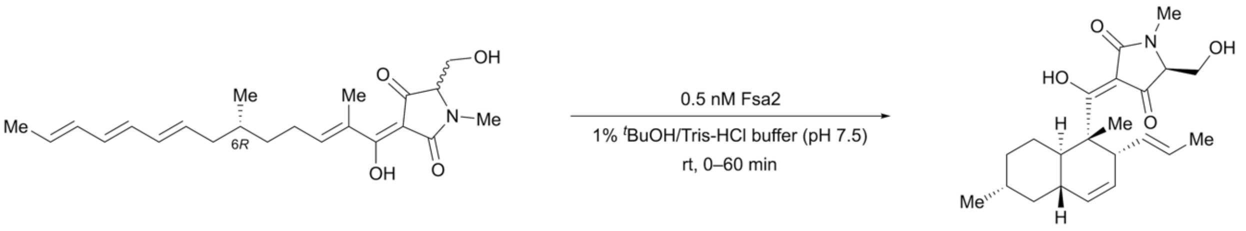

100 μM **16a**

1Aa

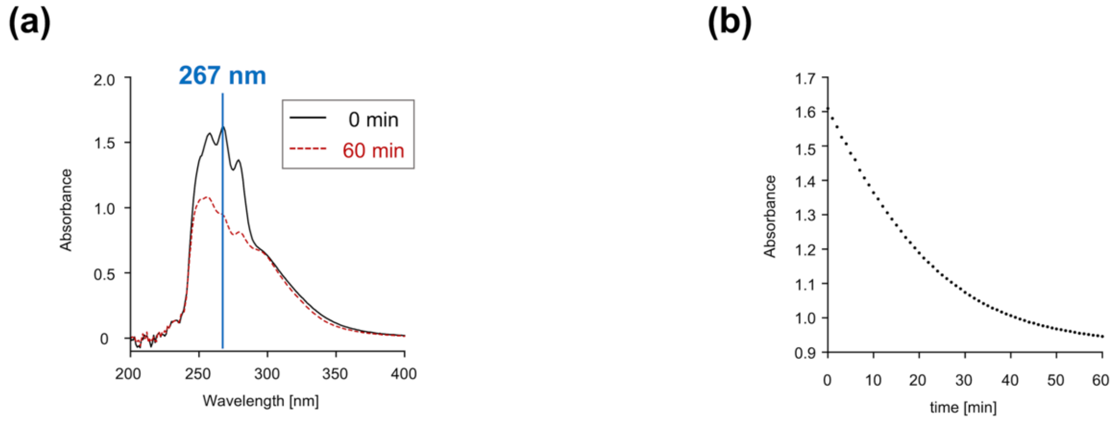

***Figure S12. Absorption spectral change in the IMDA reaction of 16a with Fsa2*:** All spectra were obtained using 100 μM **16a** solution with 0.5 nM Fsa2 in 1% ᵗBuOH/Tris - HCl buffer (pH 7.5). (a) The spectra were obtained after 0 min (black solid) and 60 min (red dash). (b) Plot of absorption measured at 267 nm against time. The data were acquired every 60 seconds.

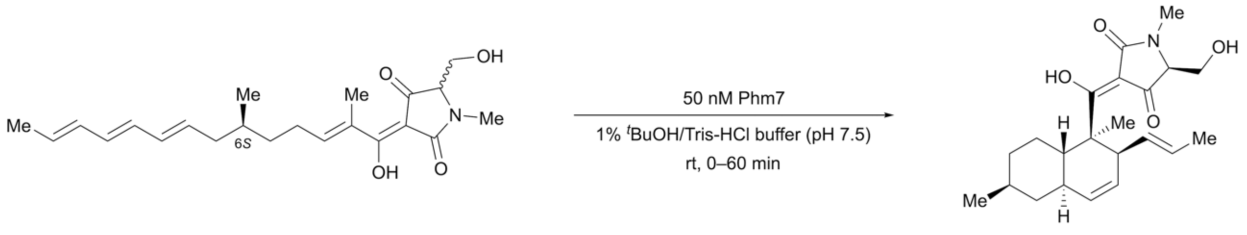

100 µM **16b**

2Aa

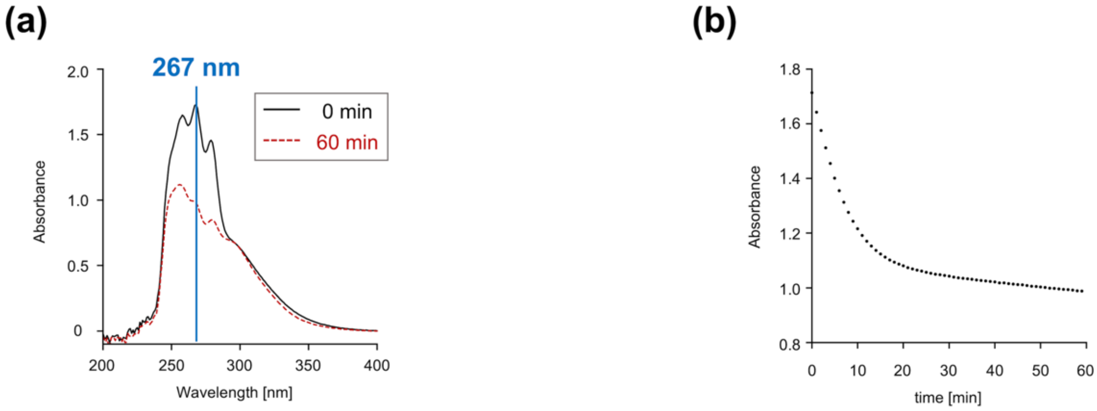

***Figure S13. Absorption spectral change in the IMDA reaction of 16b with Phm7*: All spectra were obtained using 100 µM **16b** solution with 50 nM Phm7 in 1% ^{t}BuOH/Tris - HCl buffer (pH 7.5). (a) The spectra were obtained after 0 min (black solid) and 60 min (red dash). (b) Plot of absorption measured at 267 nm against time. The data were acquired every 60 seconds.

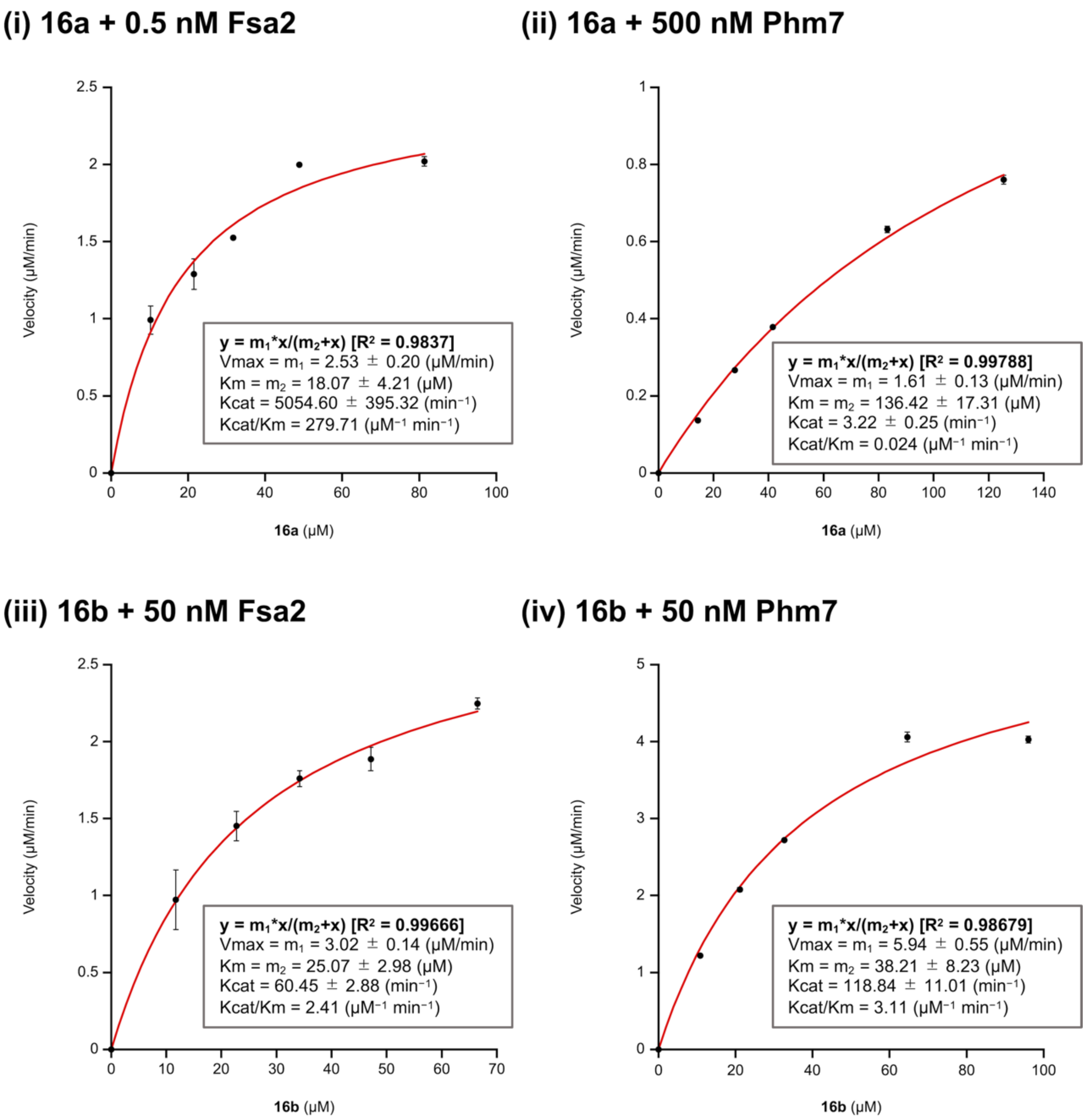

***Figure S14. Kinetic parameters of Fsa2 and Phm7-catalyzed IMDA reactions*:** The assays were performed under various conditions: (i) with 10–100 µM **16a** and 0.5 nM Fsa2; (ii) with 10–125 µM **16a** and 500 nM Phm7; (iii) with 10–70 µM **16b** and 50 nM Fsa2; (iv) with 10–100 µM **16b** and 50 nM Phm7. All assays were performed in 1% ^{t}BuOH/Tris‑HCl buffer (pH 7.5) at room temperature for 1 min. Velocity was calculated from UV absorption at 267 nm. Each data point was obtained in triplicate.

***Table S11. Enzyme kinetics of Fsa2 and Phm7*:** Corresponding Km, Kcat and Kcat/Km values with standard deviations for 3 technical replicates are shown for each tested substrate.

		Km (μM)	Kcat (min^{-1})	Kcat/Km ($μM^{-1}$ min^{-1})
Fsa2	**16a***	18.07 ± 4.21	5.05×10^3 ± 3.95×10^2	2.80×10^2
	**16b***	25.07 ± 2.98	60.45 ± 2.88	2.41
Phm7	**16a***	1.36×10^2 ± 17.31	3.22 ± 0.25	2.36×10^{-2}
	**16b***	38.21 ± 8.23	1.19×10^2 ± 11.01	3.11

*Mixture of C-5′ epimers.

5. Computational study

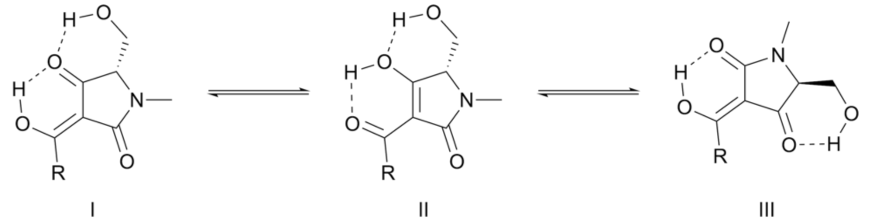

	I	II	III
Electronic Energy (EE) [A.U.] :	−1212.447955	−1212.443406	−1212.453295
EE + Zero-point Energy [A.U.] :	−1211.949839	−1211.945988	−1211.955203
Gibbs Free energy [A.U.] :	−1212.009076	−1212.007525	−1212.014489

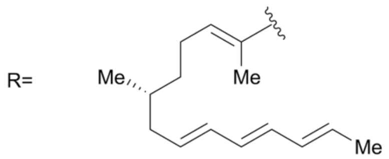

Scheme S6. The calculated conformational free energies of three tautomers of tetramic acid moiety: Structural and geometrical changes during IMDA reaction and activation free energy ($\Delta G^‡$ and $\Delta E^‡$) were calculated using M06-2X/6-311++G** (scrf =CPCM, water).

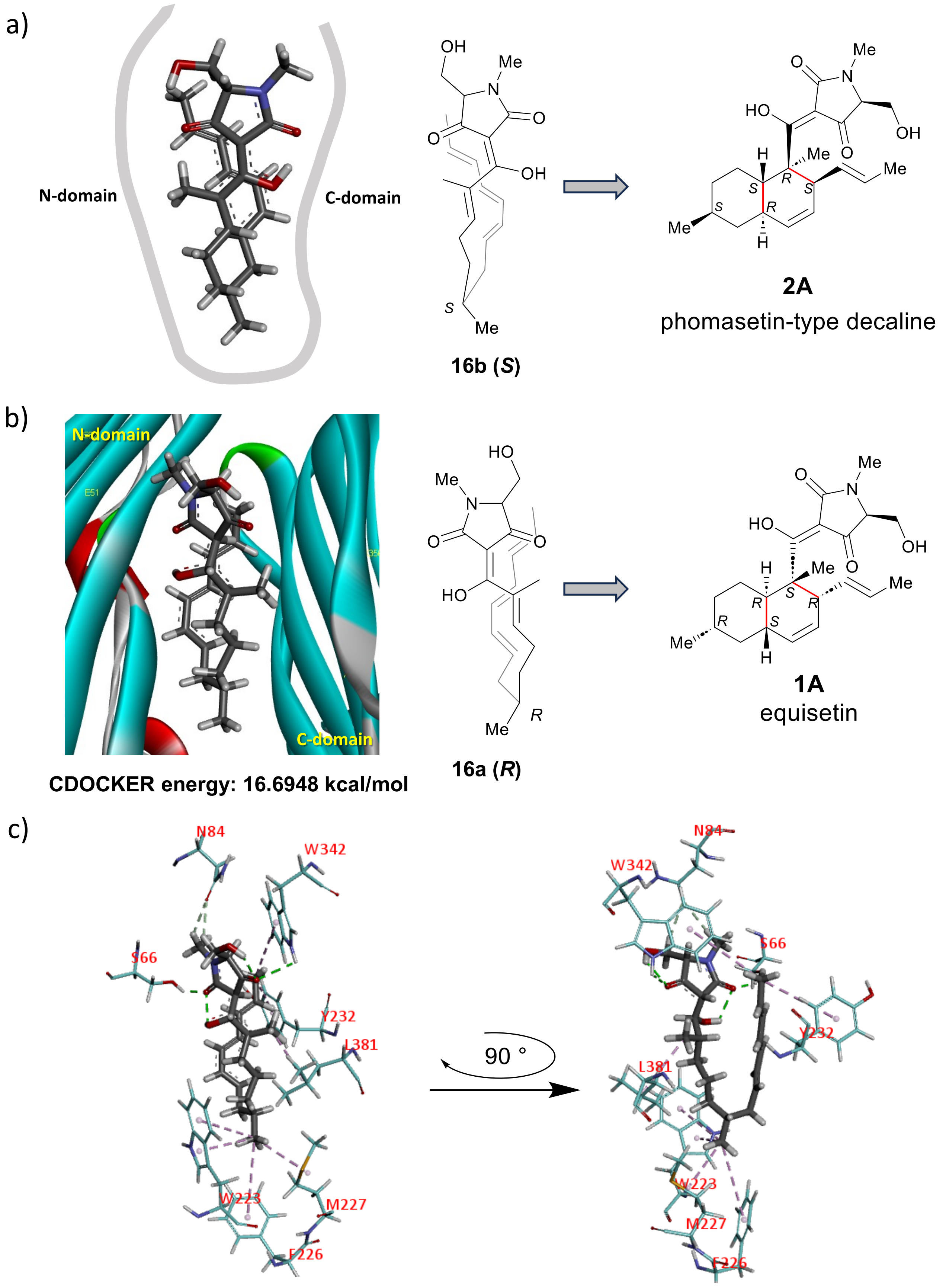

***Figure S15. Binding Mode of Phm7 (PDB ID: 7E5V) and precursor 16a*:** a) The representative binding pose of **16b** (6*S*) predicted based on the binding poses of pro-phomasetin (6*S*) in Phm7 obtained by MD simulation.[12] b) The best Docking pose of **16a** (6*R*) with Phm7. c) Interaction of **16a** with the key residues at the binding pocket.

References

1 L. T. Burke, D. J. Dixon, S. V. Ley and F. Rodríguez, *Org. Biomol. Chem.*, 2005, **3**, 274–280.

2 J. Cárdenas, J. Morales-Serna, E. García-Ríos, J. Bernal, E. Paleo and R. Gaviño, *Synthesis*, 2011, **9**, 1375–1382.

3 V. V. Sokolov, S. I. Kozhushkov, S. Nikolskaya, V. N. Belov, M. Es-Sayed and A. de Meijere, *European J. Org. Chem.*, 1998, **5**, 777–783.

4 K. N. White and J. P. Konopelski, *Org. Lett.*, 2005, **7**, 4111–4112.

5 J. Kuhnke and F. Bohlmann, *Justus Liebigs Ann. Chem.*, 1988, **8**, 743–748.

6 M. Zaghouani, C. Kunz, L. Guédon, F. Blanchard and B. Nay, *Chemistry*, 2016, **22**, 15257–15260.

7 Y. Nishikawa, M. Kitajima, N. Kogure and H. Takayama, *Tetrahedron*, 2009, **65**, 1608–1617.

8 M. Heinrich, J. J. Murphy, M. K. Ilg, A. Letort, J. Flasz, P. Philipps and A. Fürstner, *Angew. Chem. Int. Ed Engl.*, 2018, **57**, 13575–13581.

9 J. Yin, L. Kong, C. Wang, Y. Shi, S. Cai and S. Gao, *Chemistry*, 2013, **19**, 13040–13046.

10 L. Kong, M. Rao, J. Ou, J. Yin, W. Lu, M. Liu, X. Pang and S. Gao, *Org. Biomol. Chem.*, 2014, **12**, 7591–7597.

11 N. Kato, T. Nogawa, H. Hirota, J.-H. Jang, S. Takahashi, J. S. Ahn and H. Osada, *Biochem. Biophys. Res. Commun.*, 2015, **460**, 210–215.

12 K. Fujiyama, N. Kato, S. Re, K. Kinugasa, K. Watanabe, R. Takita, T. Nogawa, T. Hino, H. Osada, Y. Sugita, S. Takahashi and S. Nagano, *Angew. Chem. Int. Ed Engl.*, 2021, **60**, 22401–22410.

13 J. K. Gagnon, S. M. Law and C. L. Brooks 3rd, *J. Comput. Chem.*, 2016, **37**, 753–762.

Figure S16. ^{1}H NMR spectrum of Compound 7 in CDCl₃ (500 MHz)

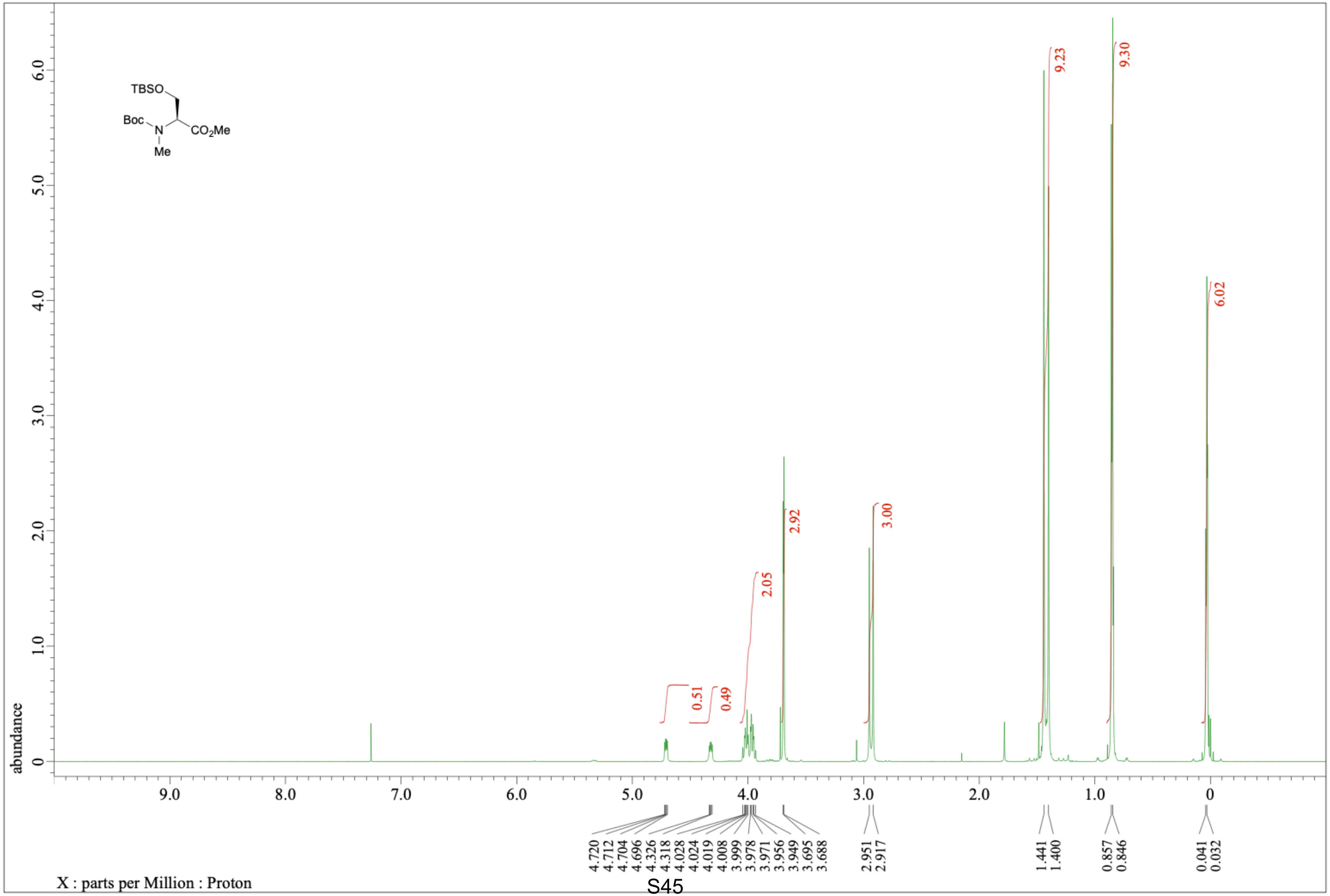

Figure S17. ^{13}C NMR spectrum of Compound 7 in $CDCl_3$ (100 MHz)

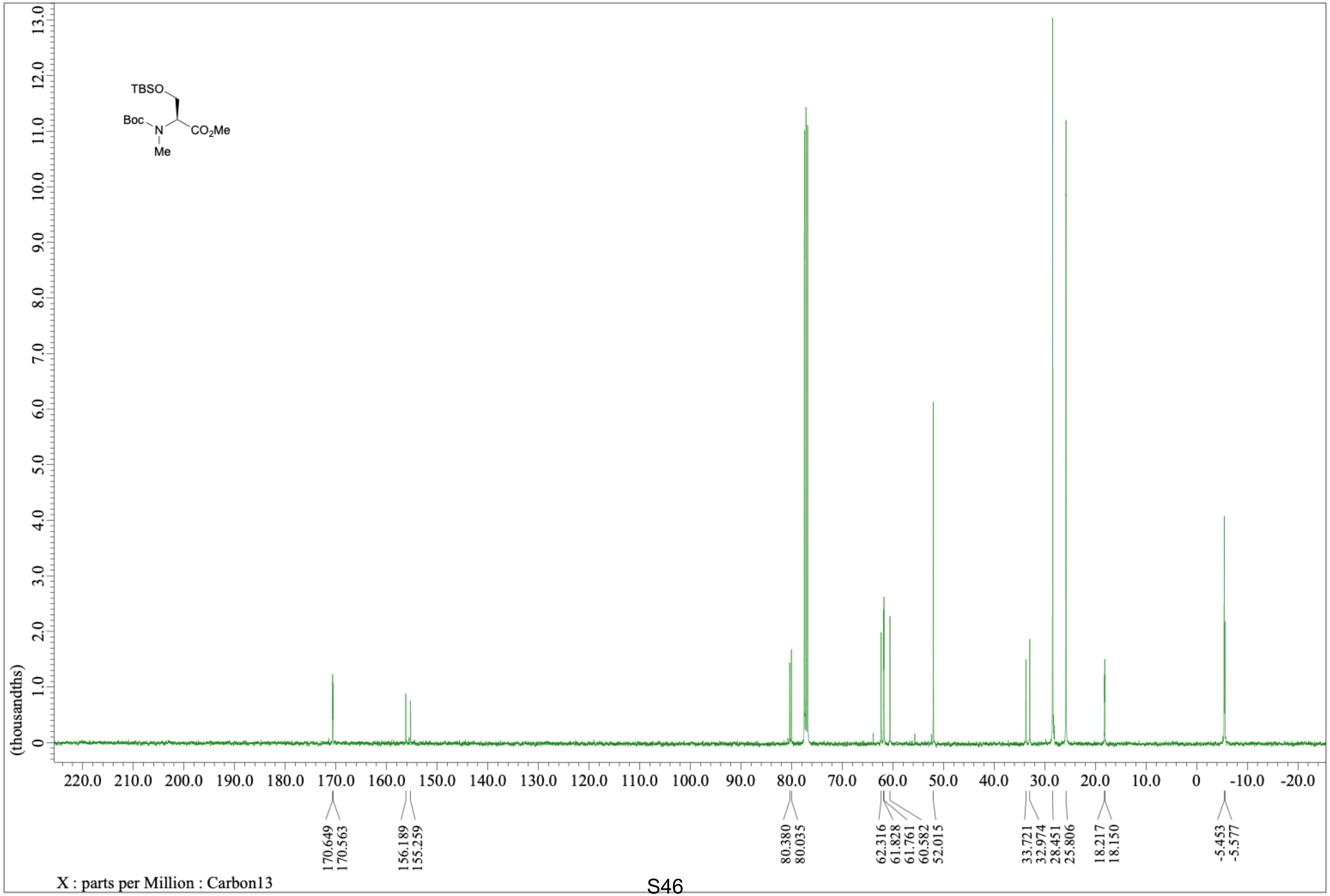

Figure S18. ***^{1}H NMR spectrum of Compound 13b in CDCl₃ (500 MHz)***

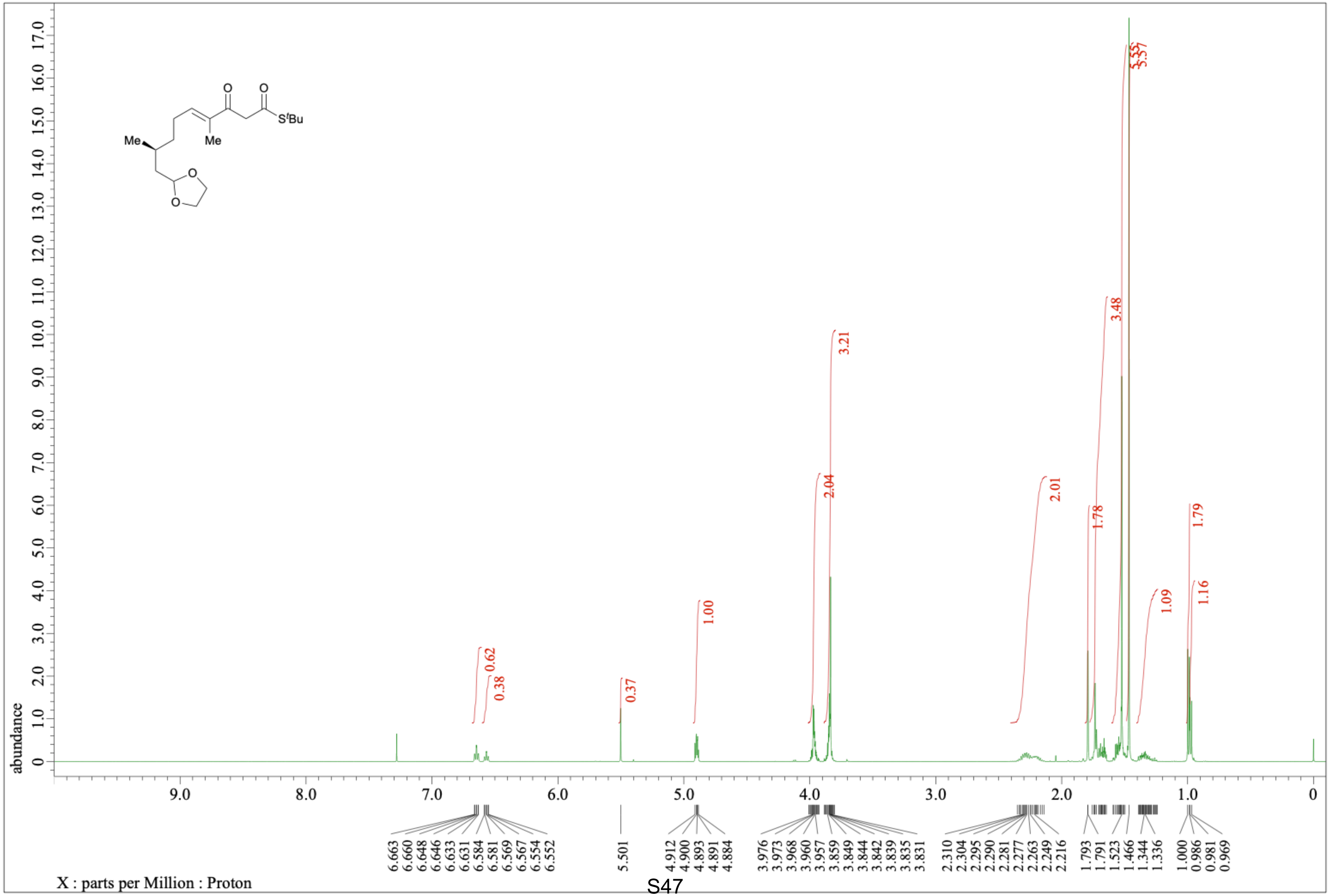

Figure S19. ***^{13}C NMR spectrum of Compound 13b in $CDCl_3$ (100 MHz)***

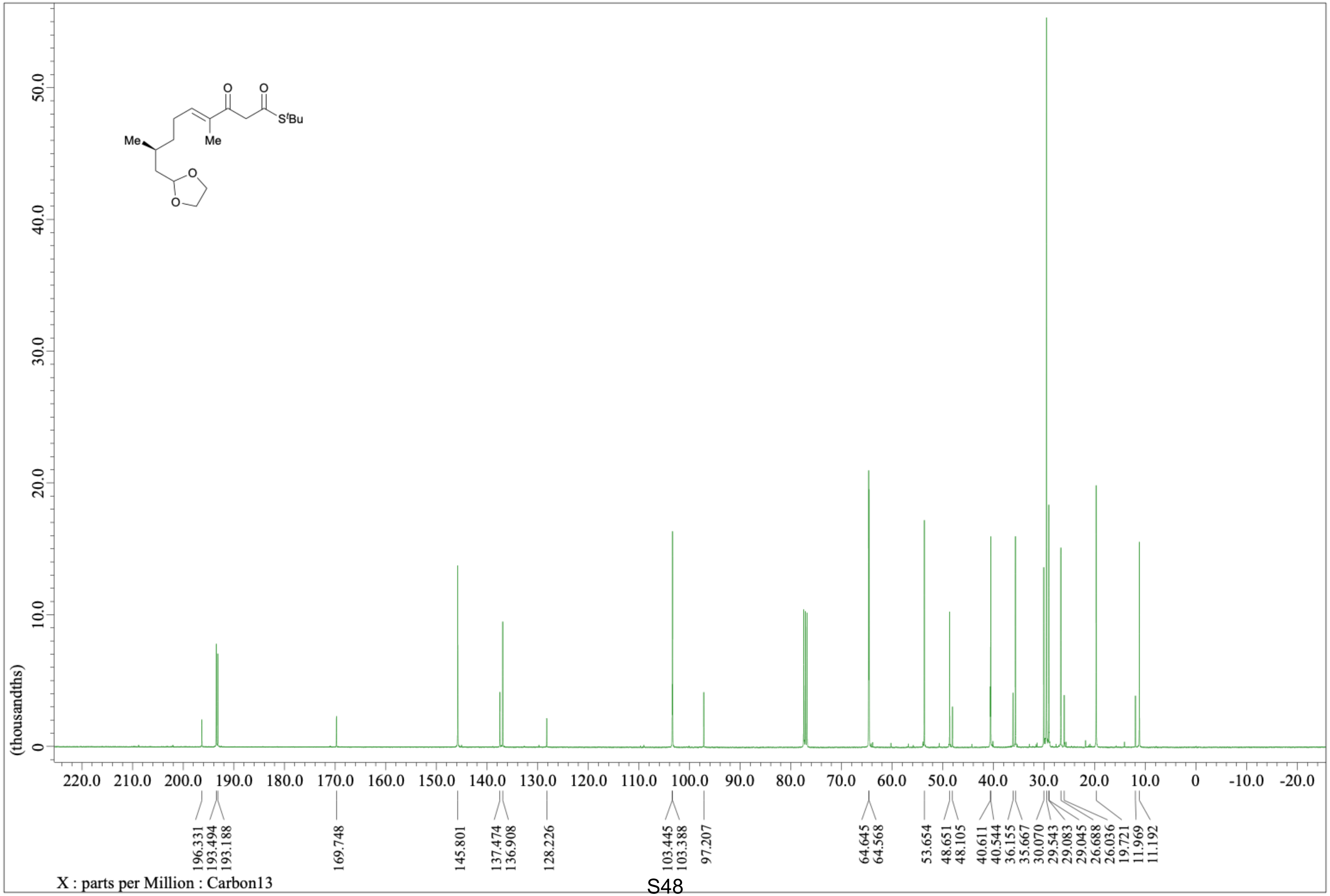

Figure S20. ^{1}H NMR spectrum of Compound 14b in CDCl₃ (400 MHz)

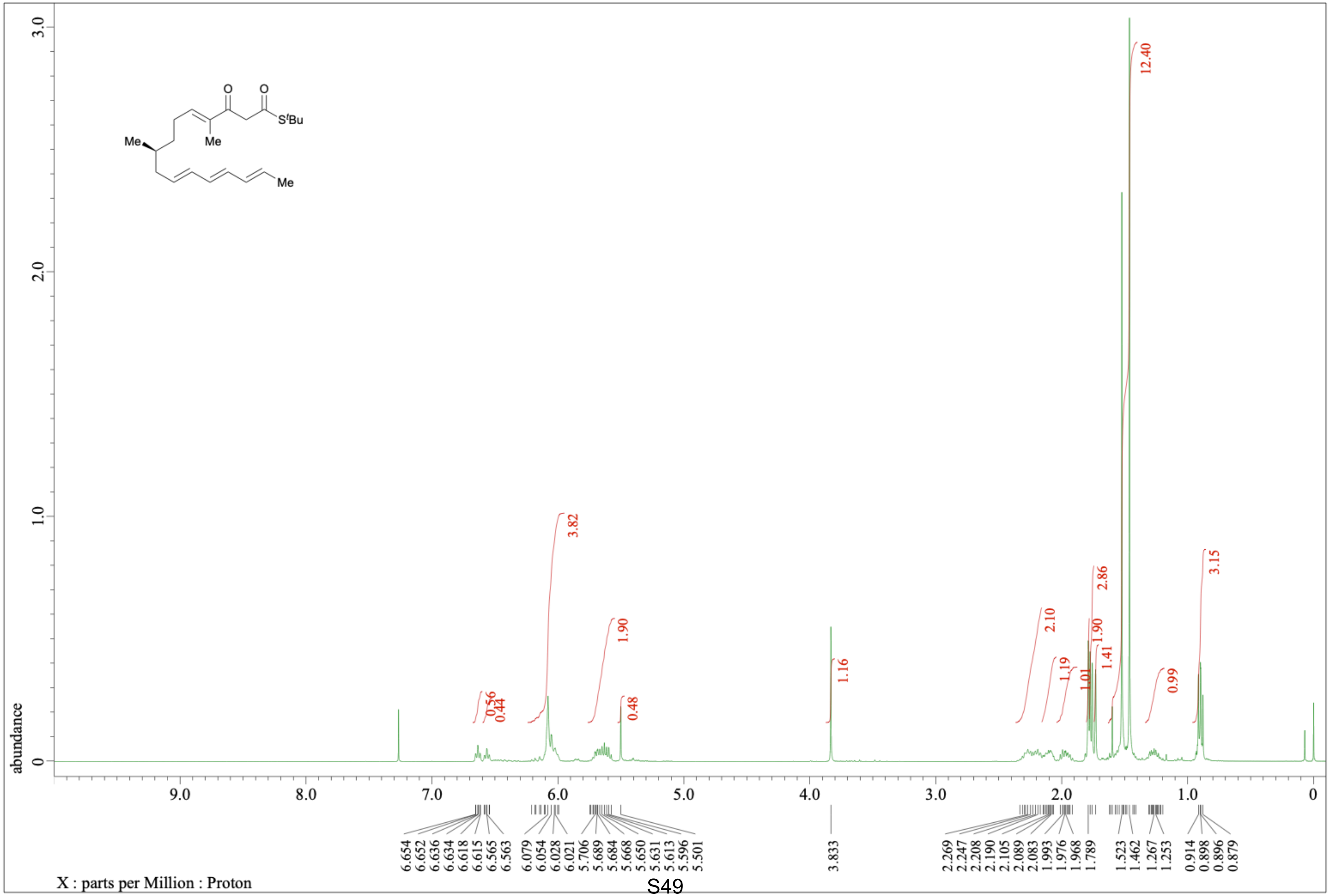

Figure S21. ^{13}C NMR spectrum of Compound 14b in CDCl$_3$ (125 MHz)

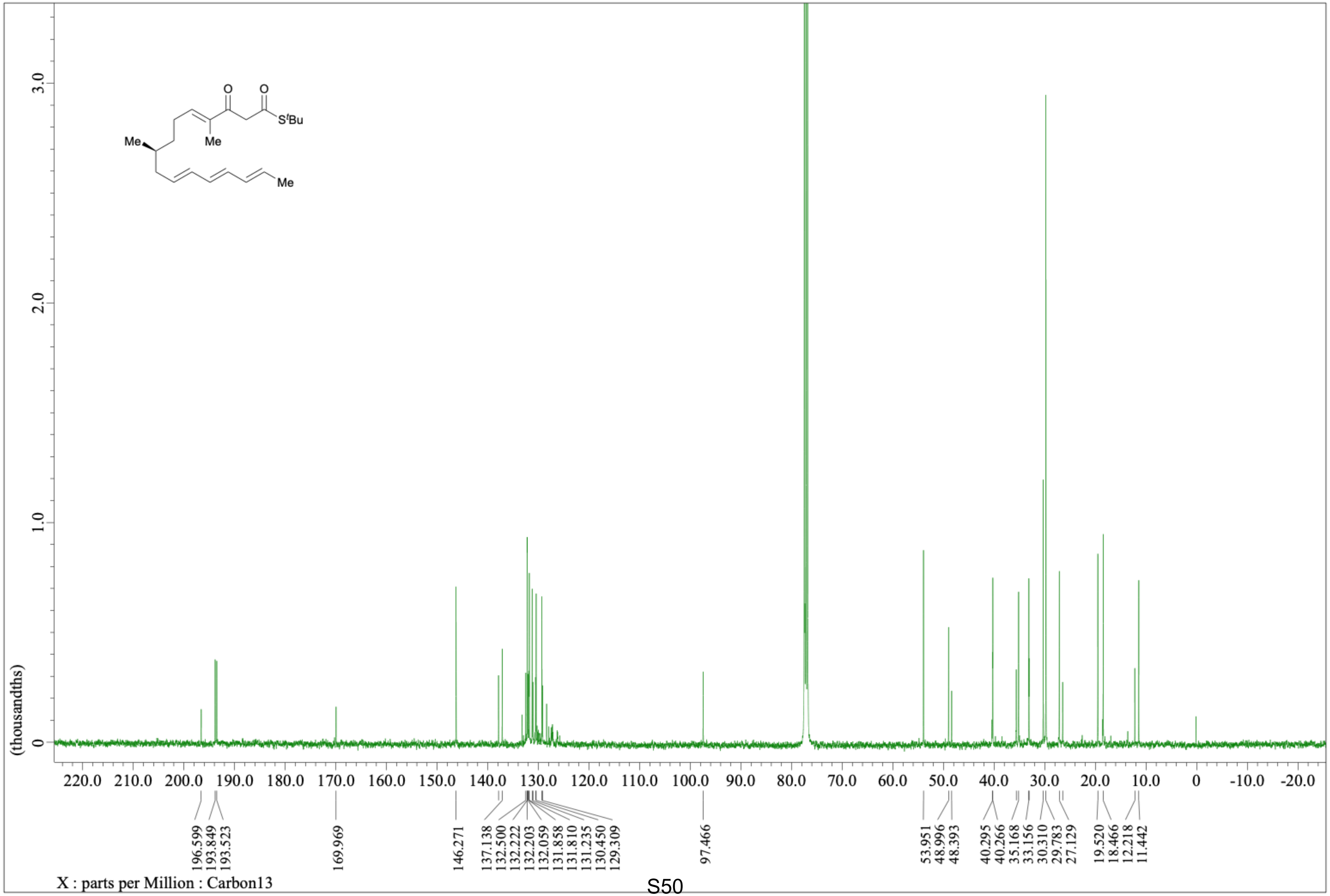

Figure S22. ^{1}H NMR spectrum of Compound 15b in CDCl₃ (400 MHz)

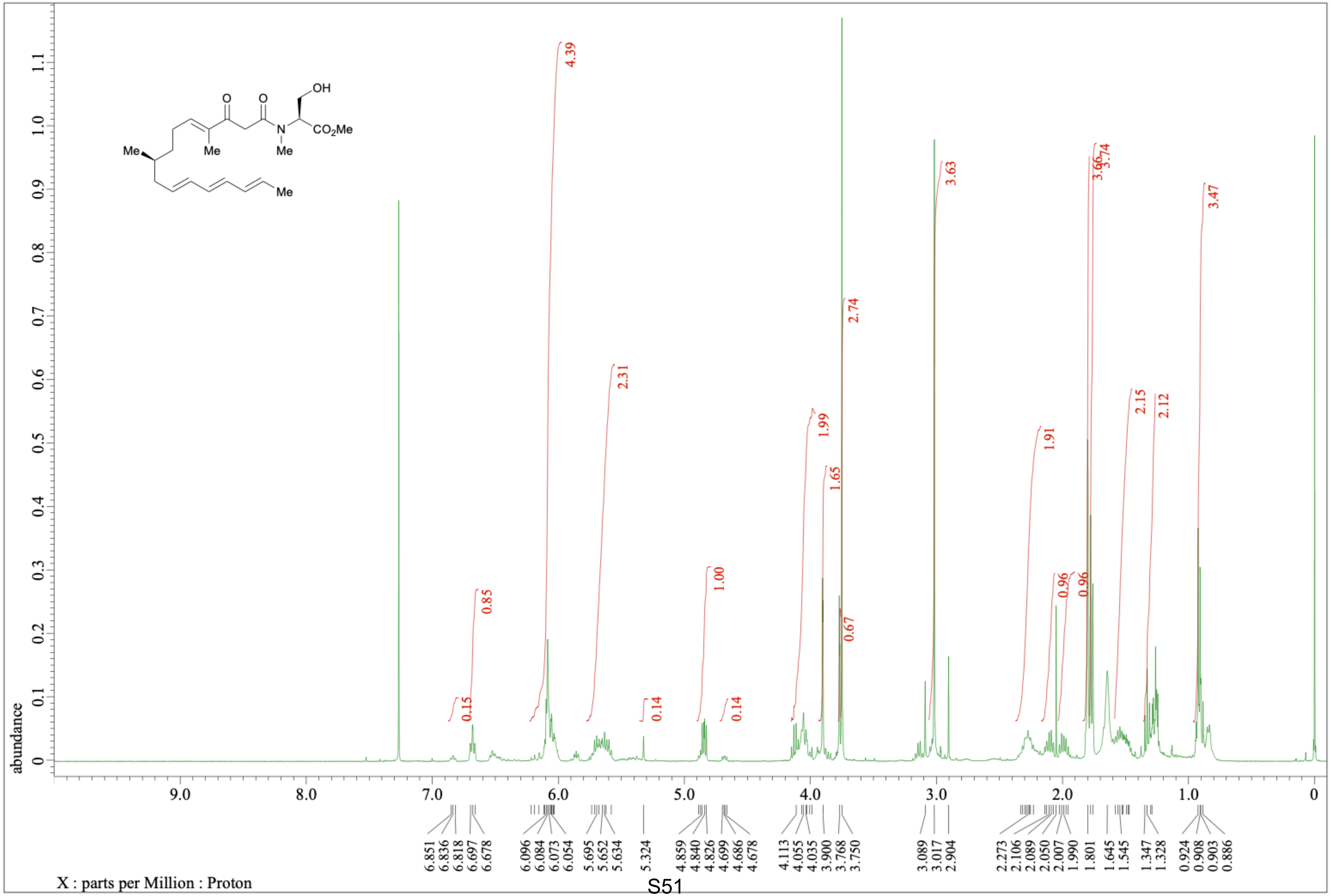

Figure S23. 13C NMR spectrum of Compound 15b in CDCl3 (100 MHz)

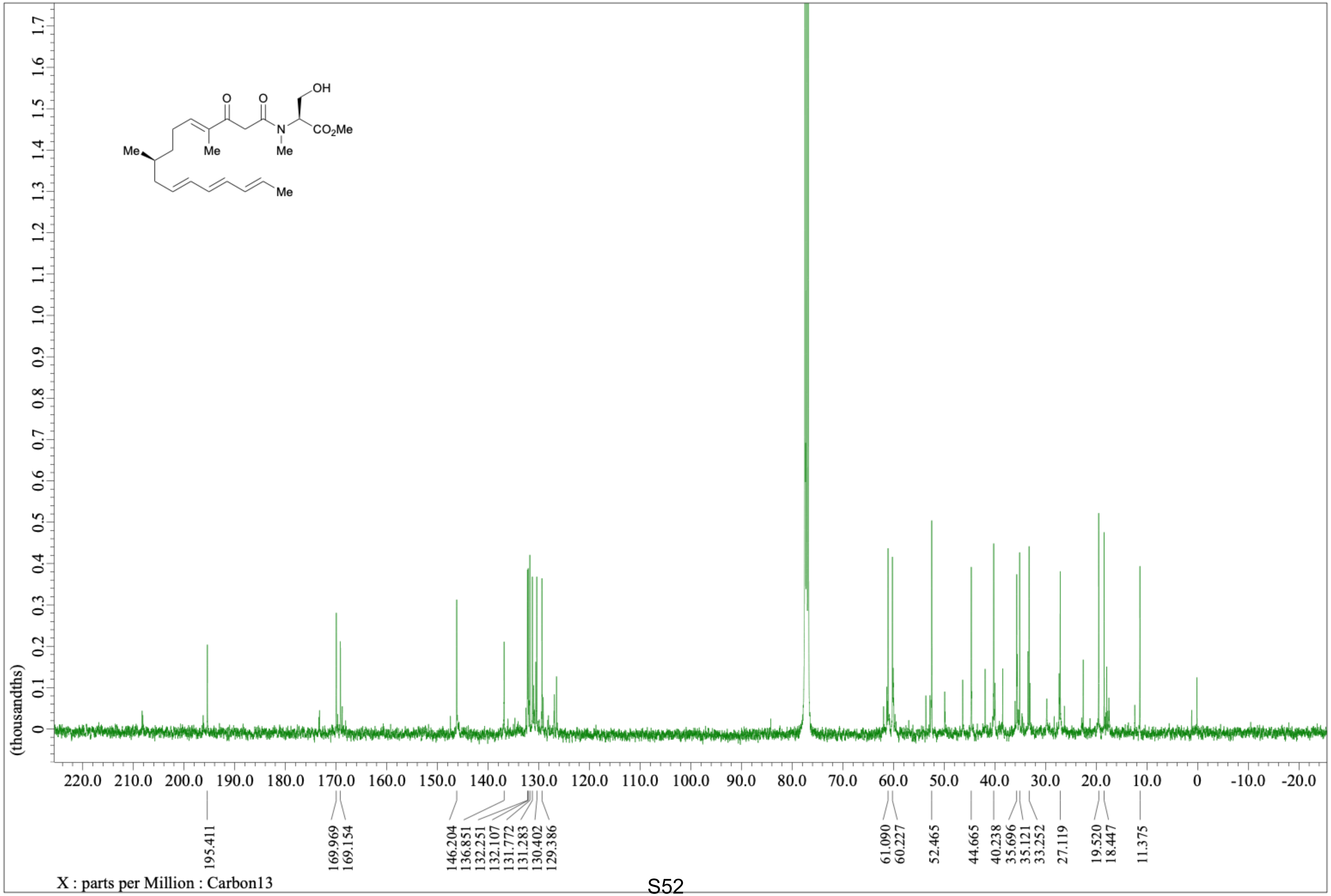

Figure S24. ^{1}H NMR spectrum of Compound 1Aa in $CDCl_3$ (500 MHz)

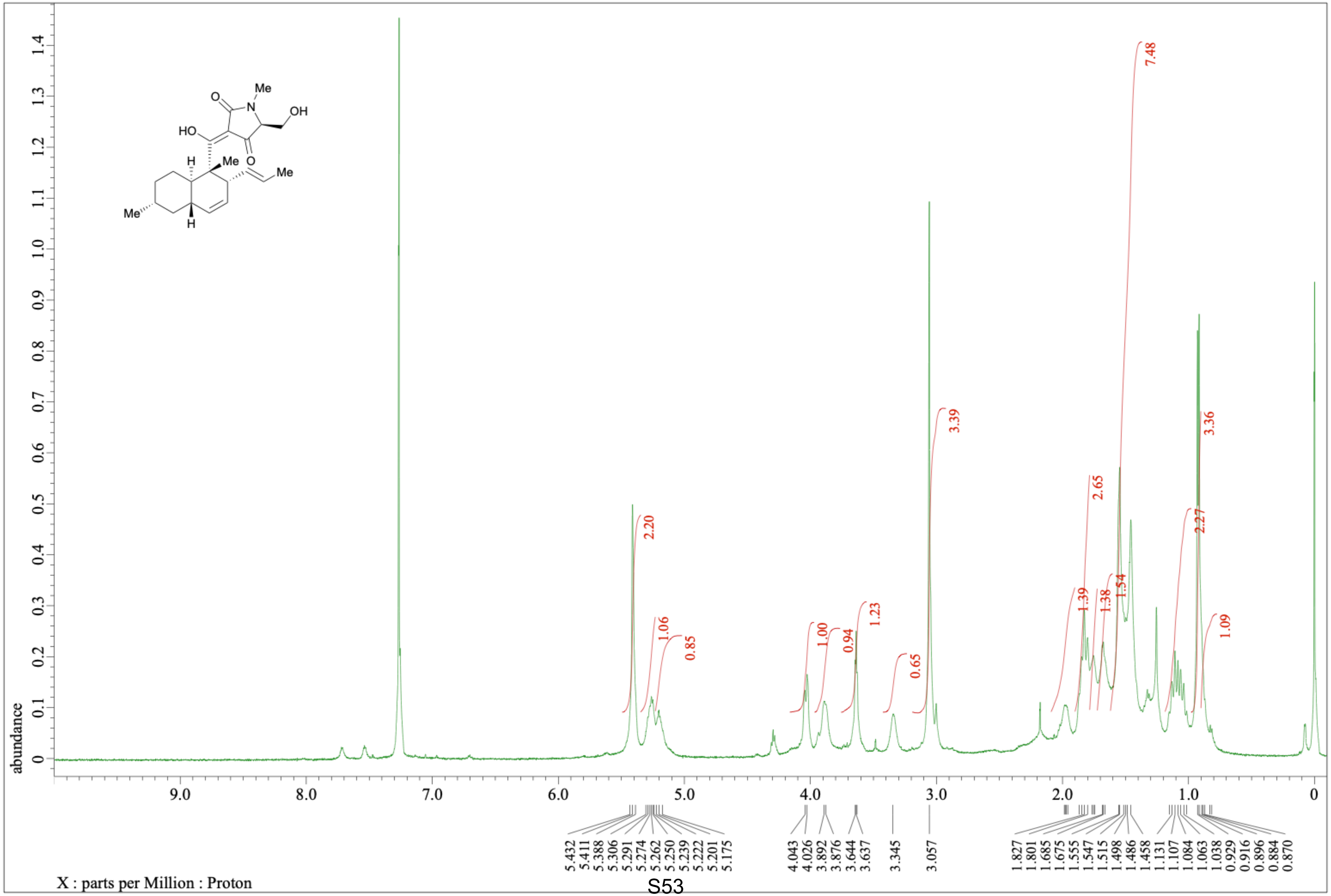

Figure S25. ^{13}C NMR spectrum of Compound 1Aa in $CDCl_3$ (125 MHz)

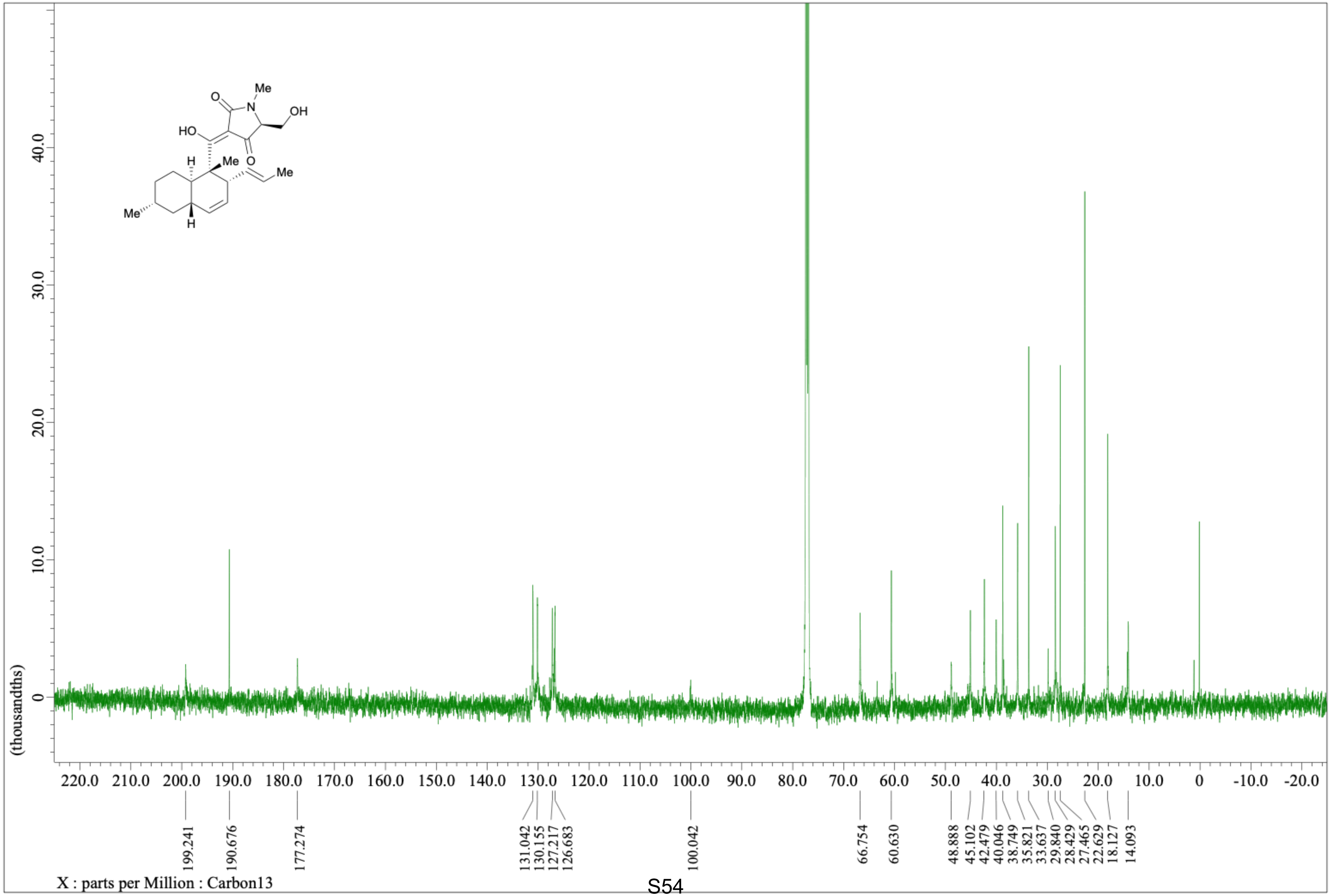

***Figure S26.** *[1]*H NMR spectrum of Compound 1Ab in* $CDCl_3$ *(500 MHz)*

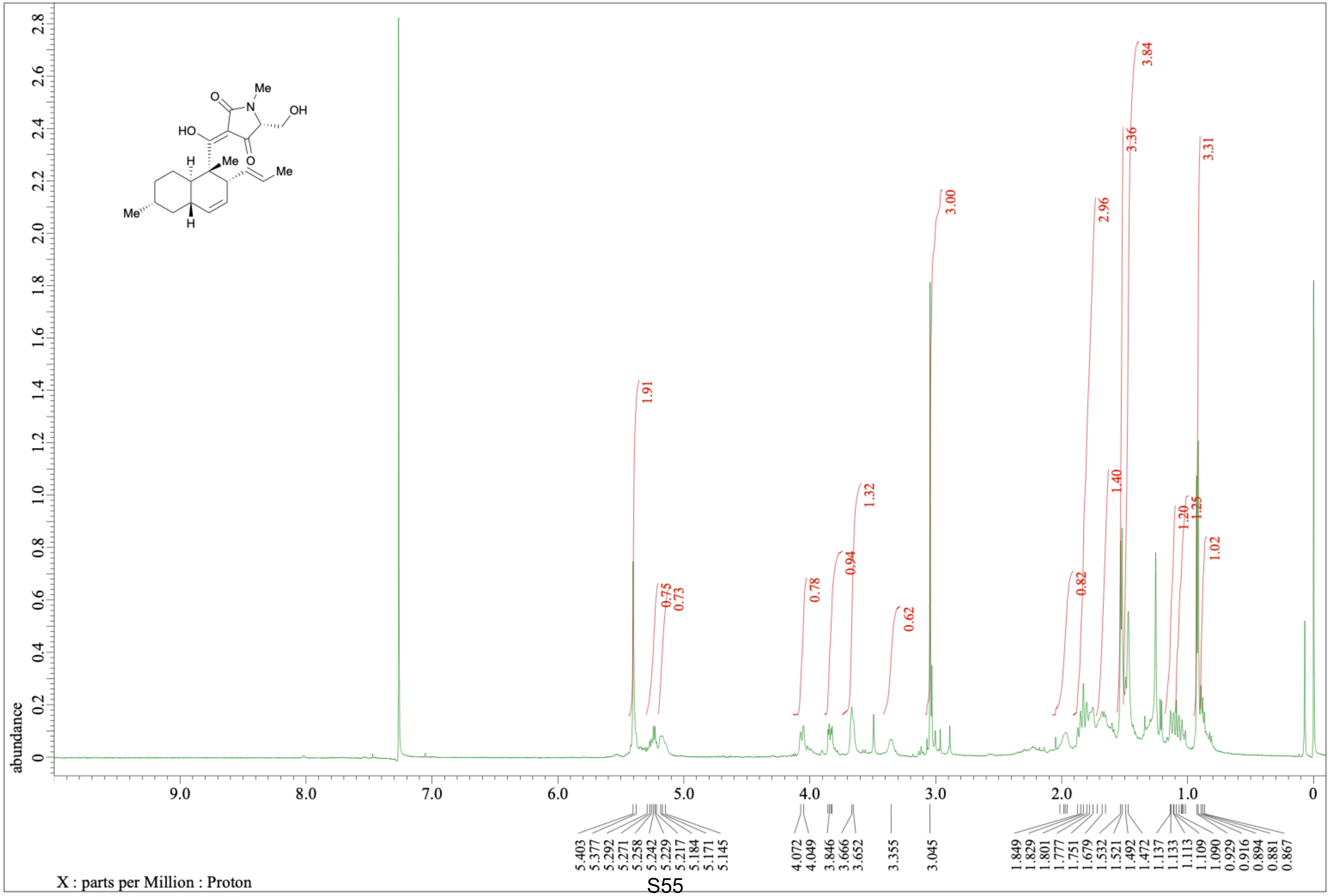

Figure S27. ^{13}C NMR spectrum of Compound 1Ab in $CDCl_3$ (125 MHz)

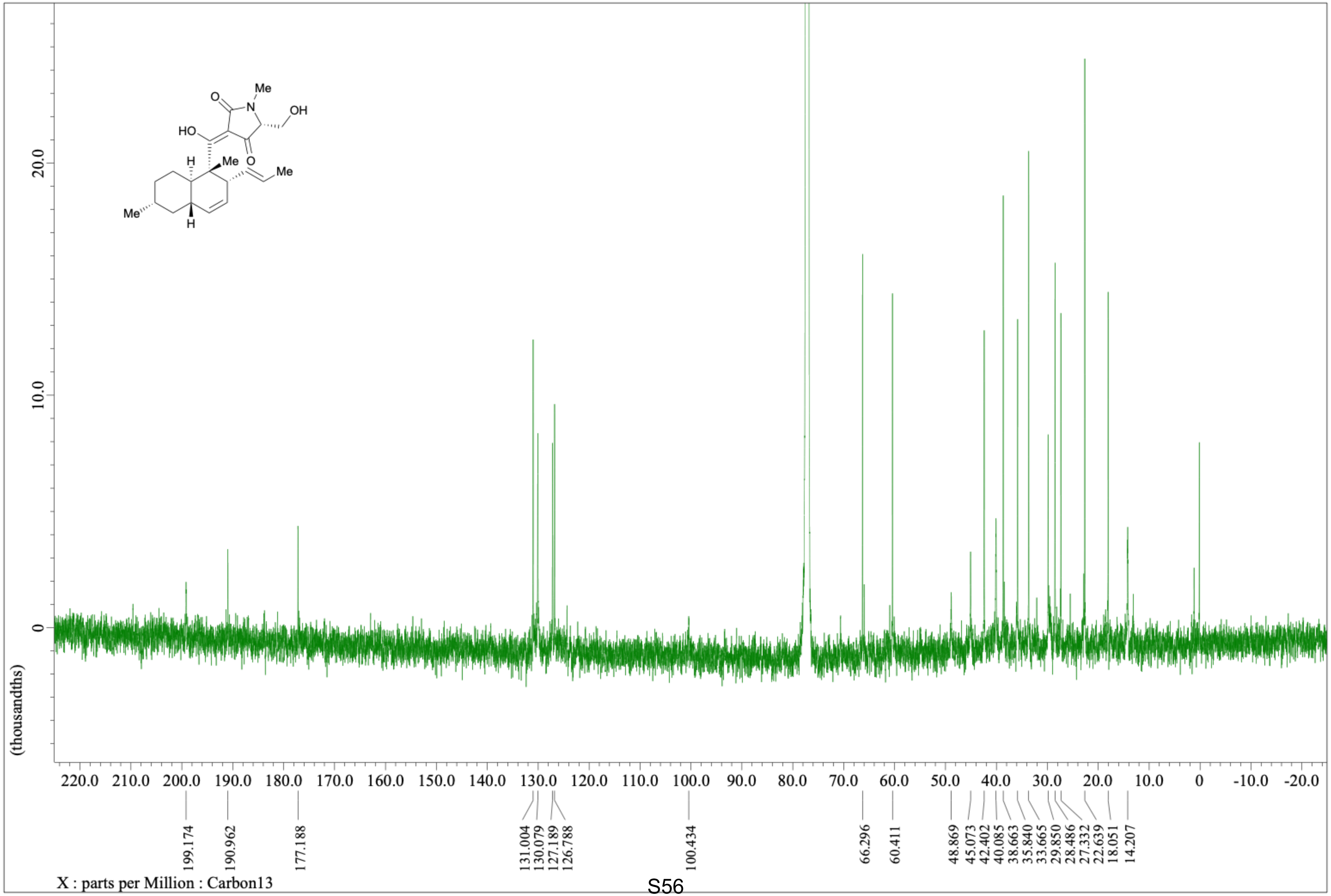

Figure S28. HH-COSY spectrum of Compound 1Ab in CDCl$_3$

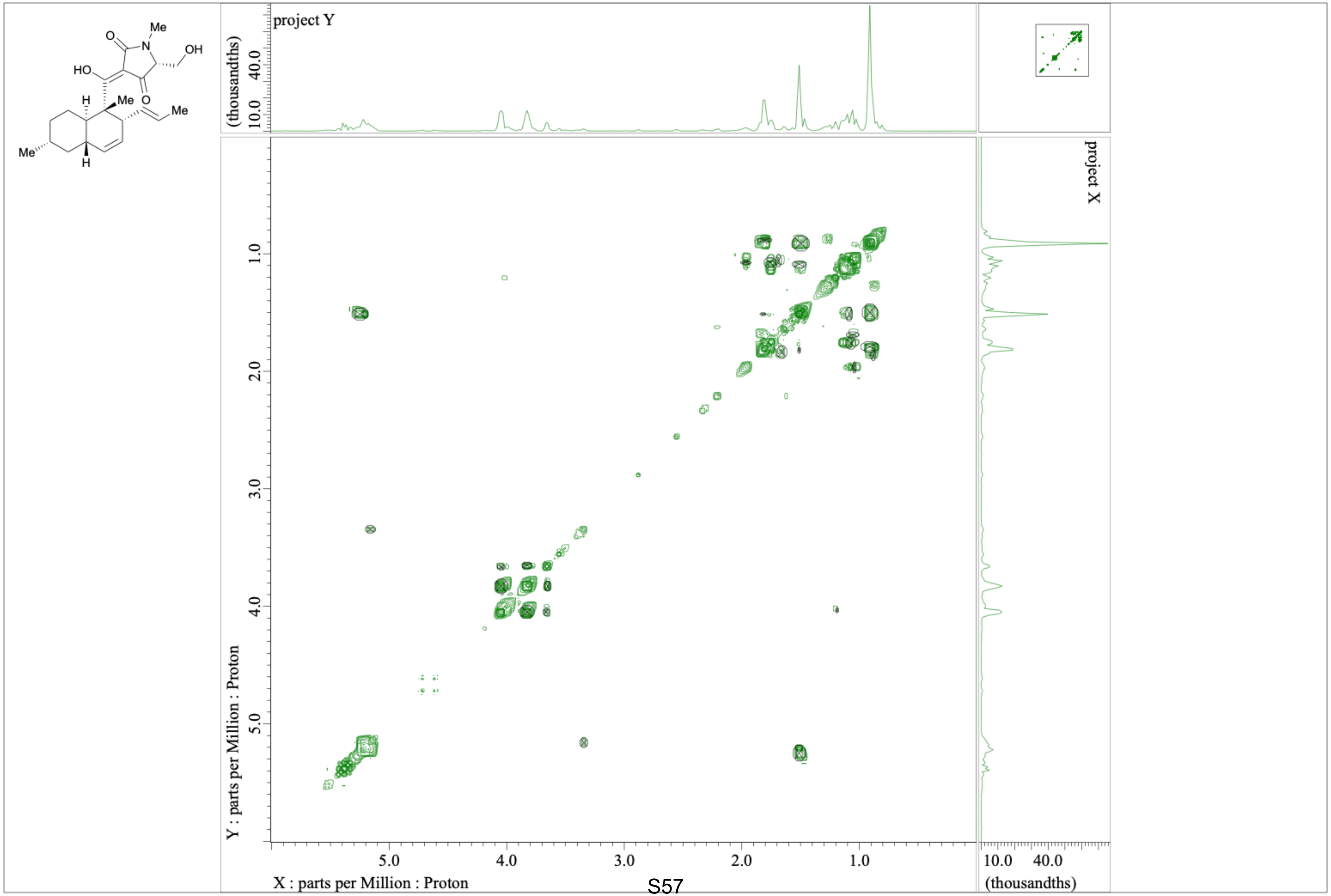

Figure S29. HSQC spectrum of Compound 1Ab in $CDCl_3$

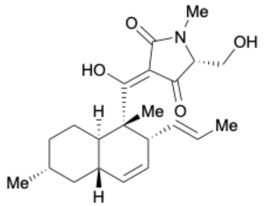

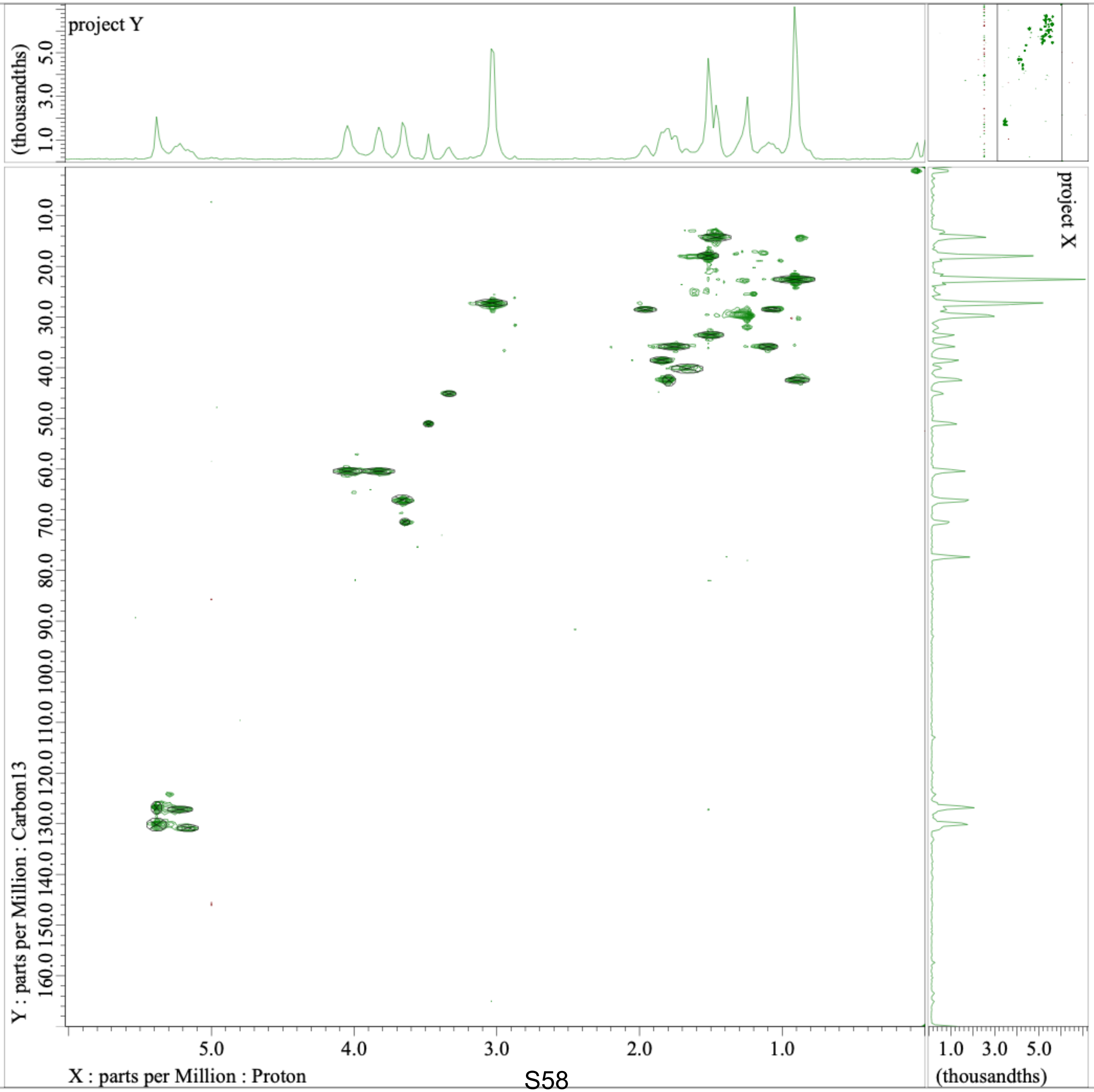

Figure S30. HMBC spectrum of Compound 1Ab in CDCl$_3$

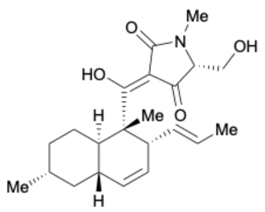

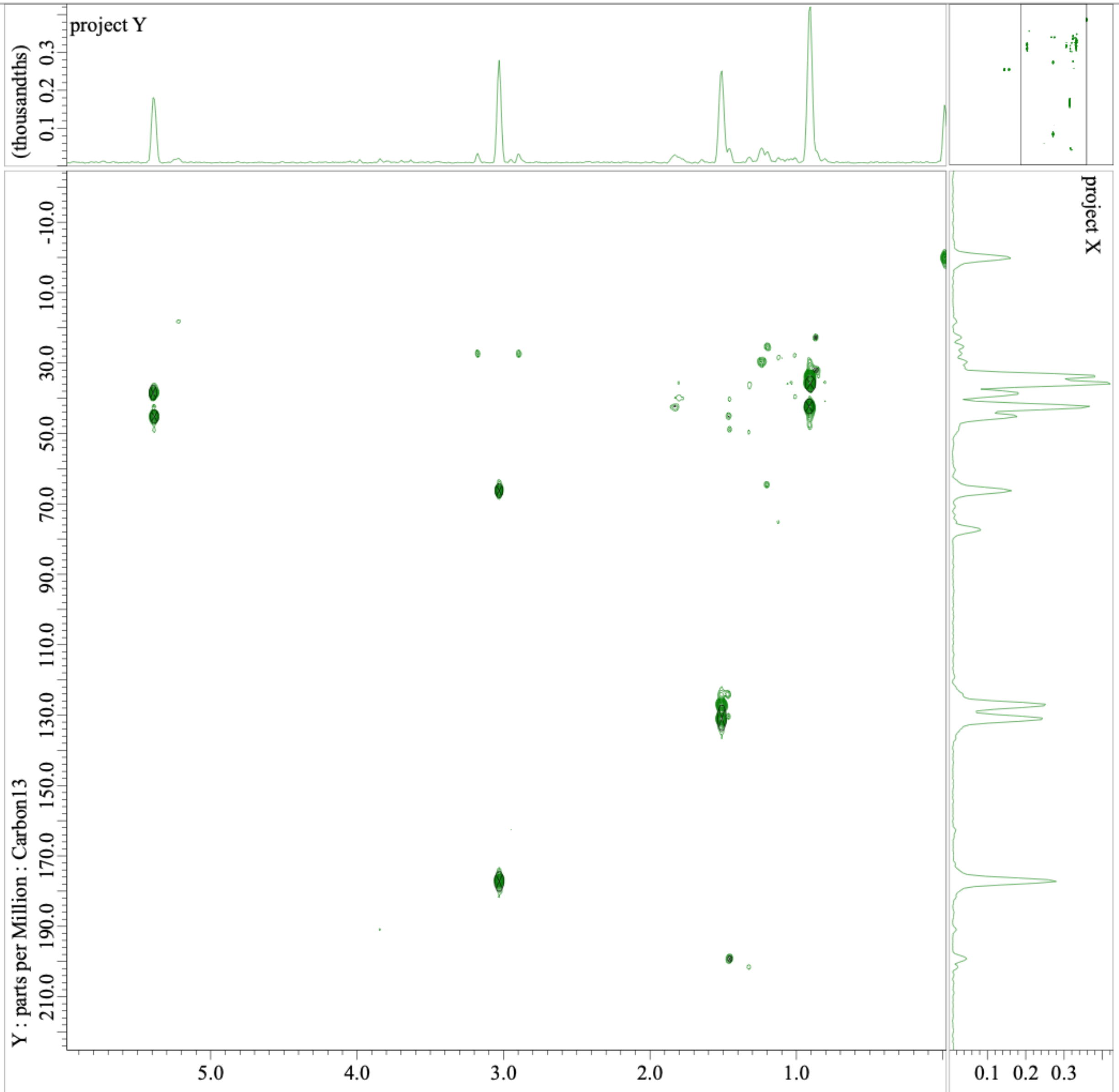

Figure S31. NOESY spectrum of Compound 1Ab in $CDCl_3$

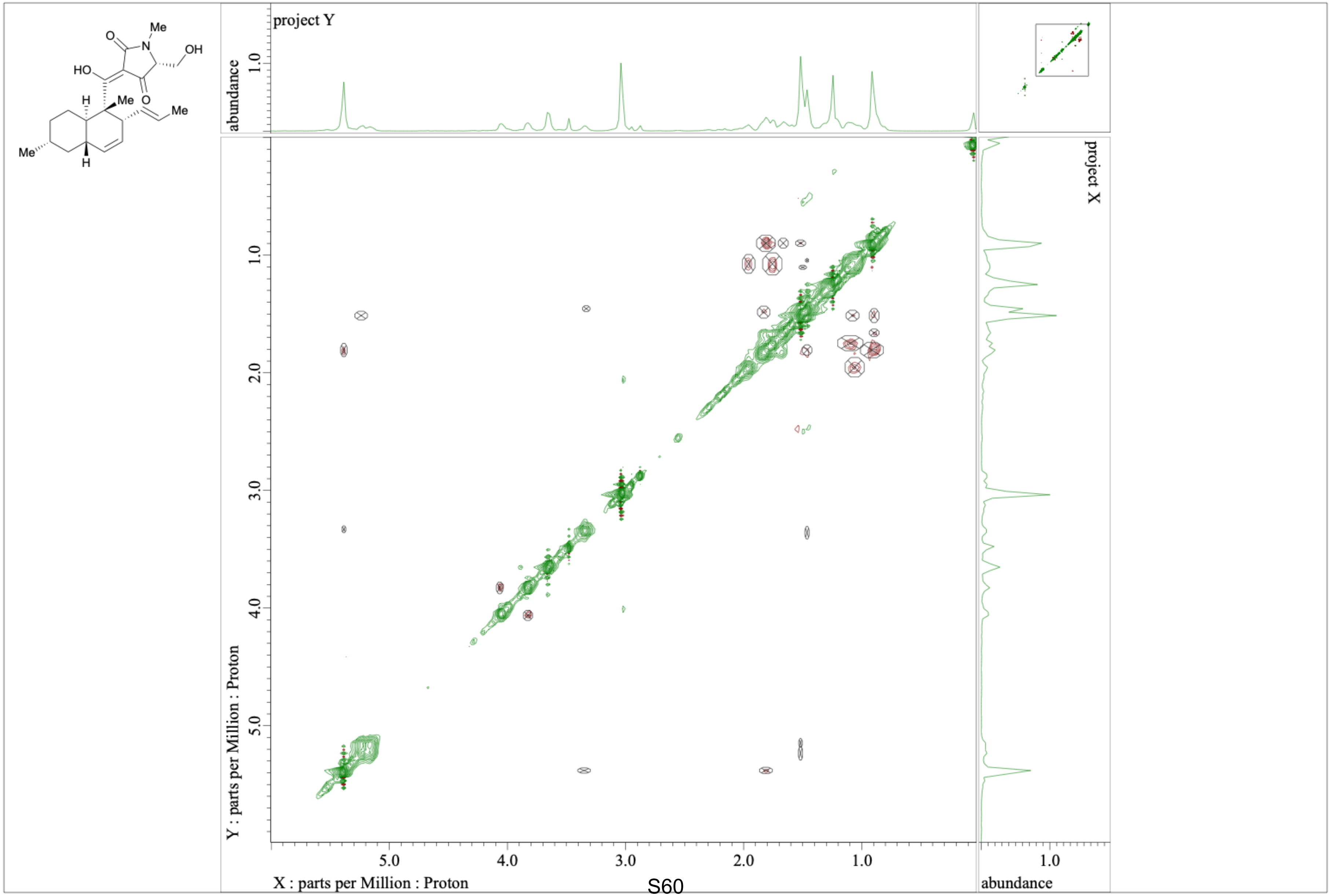

Figure S32. ***^{1}H NMR spectrum of Compound 2Aa in $CDCl_3$ (500 MHz)***

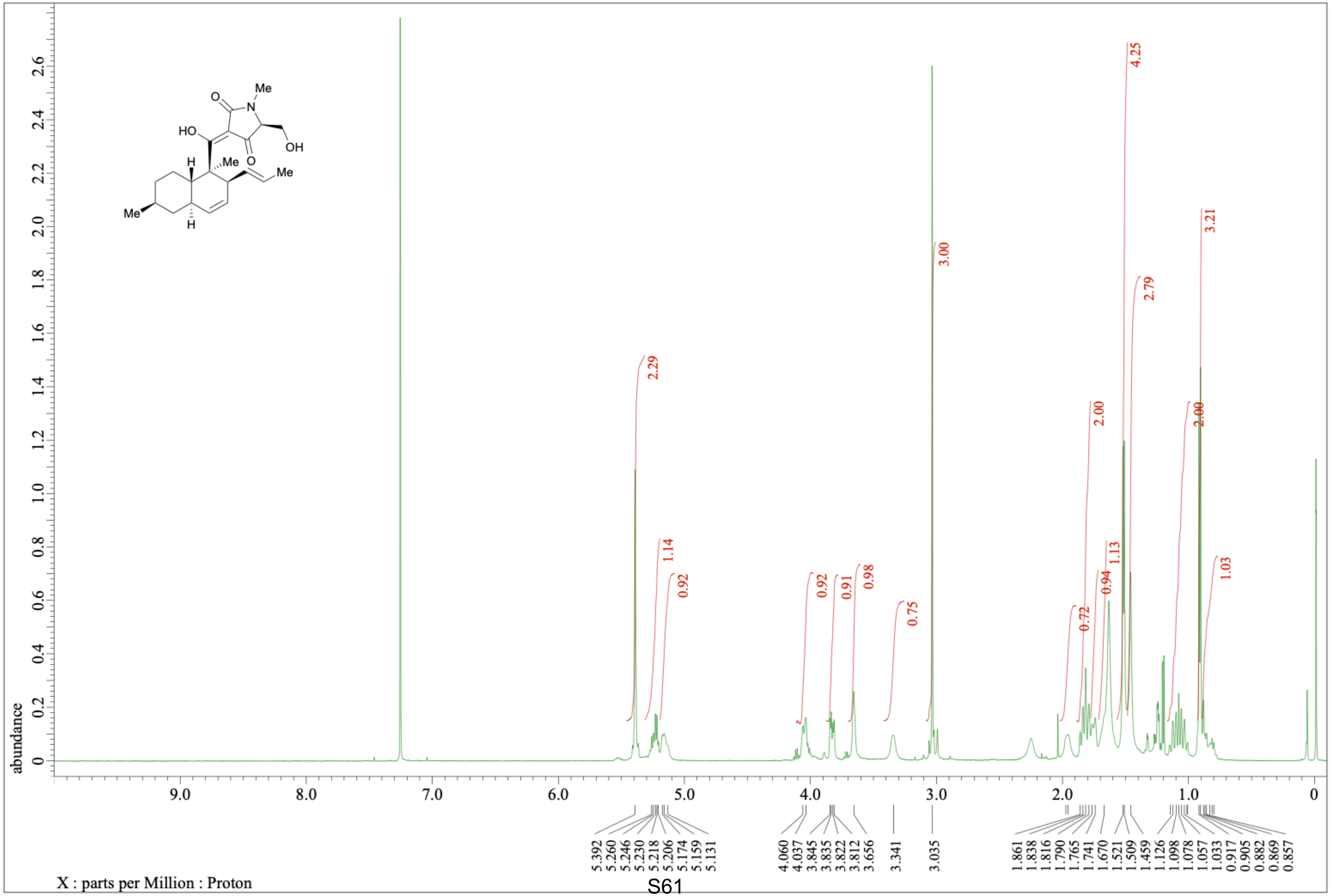

Figure S33. ***^{13}C NMR spectrum of Compound 2Aa in $CDCl_3$ (125 MHz)***

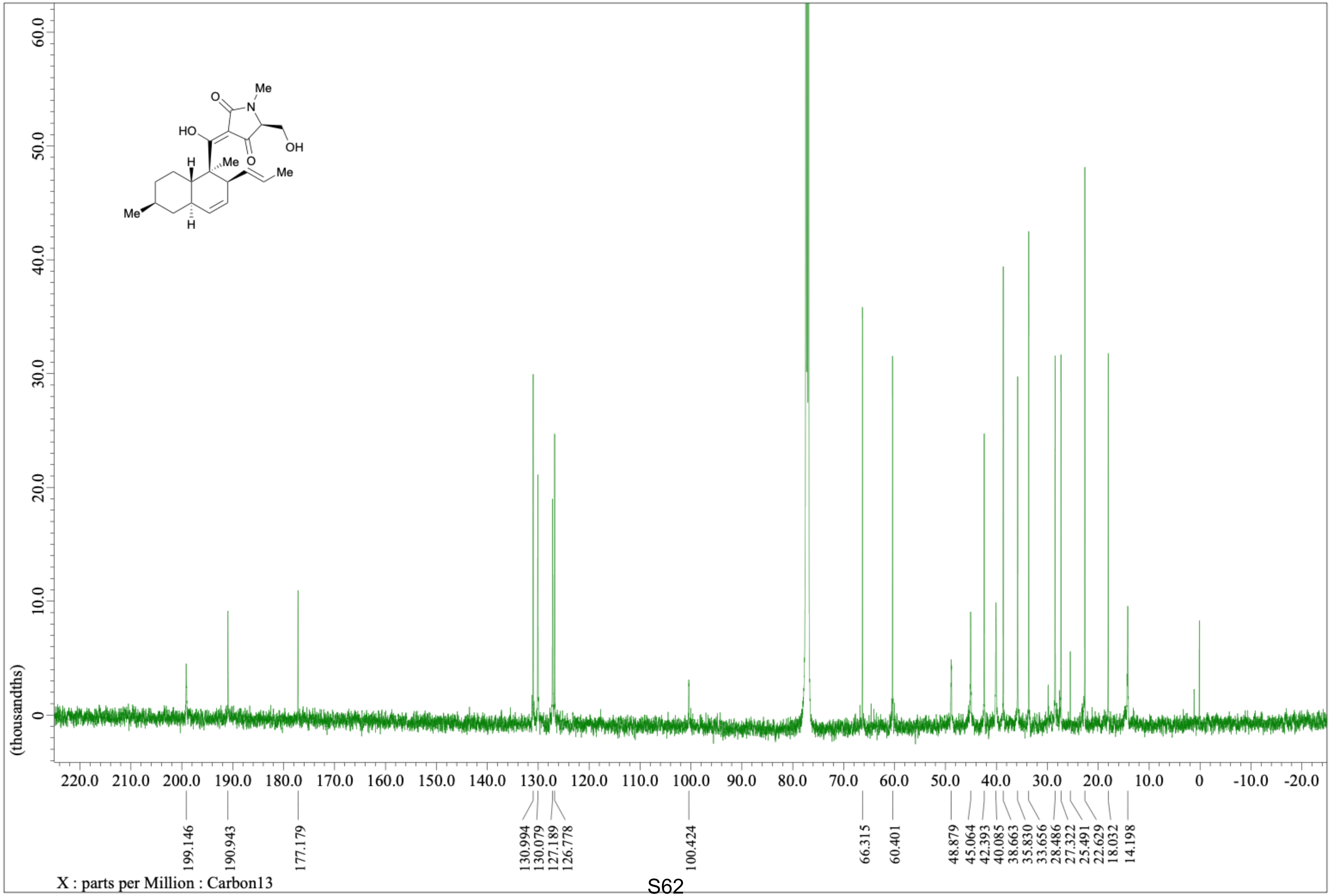

Figure S34. HH-COSY spectrum of Compound 2Aa in $CDCl_3$

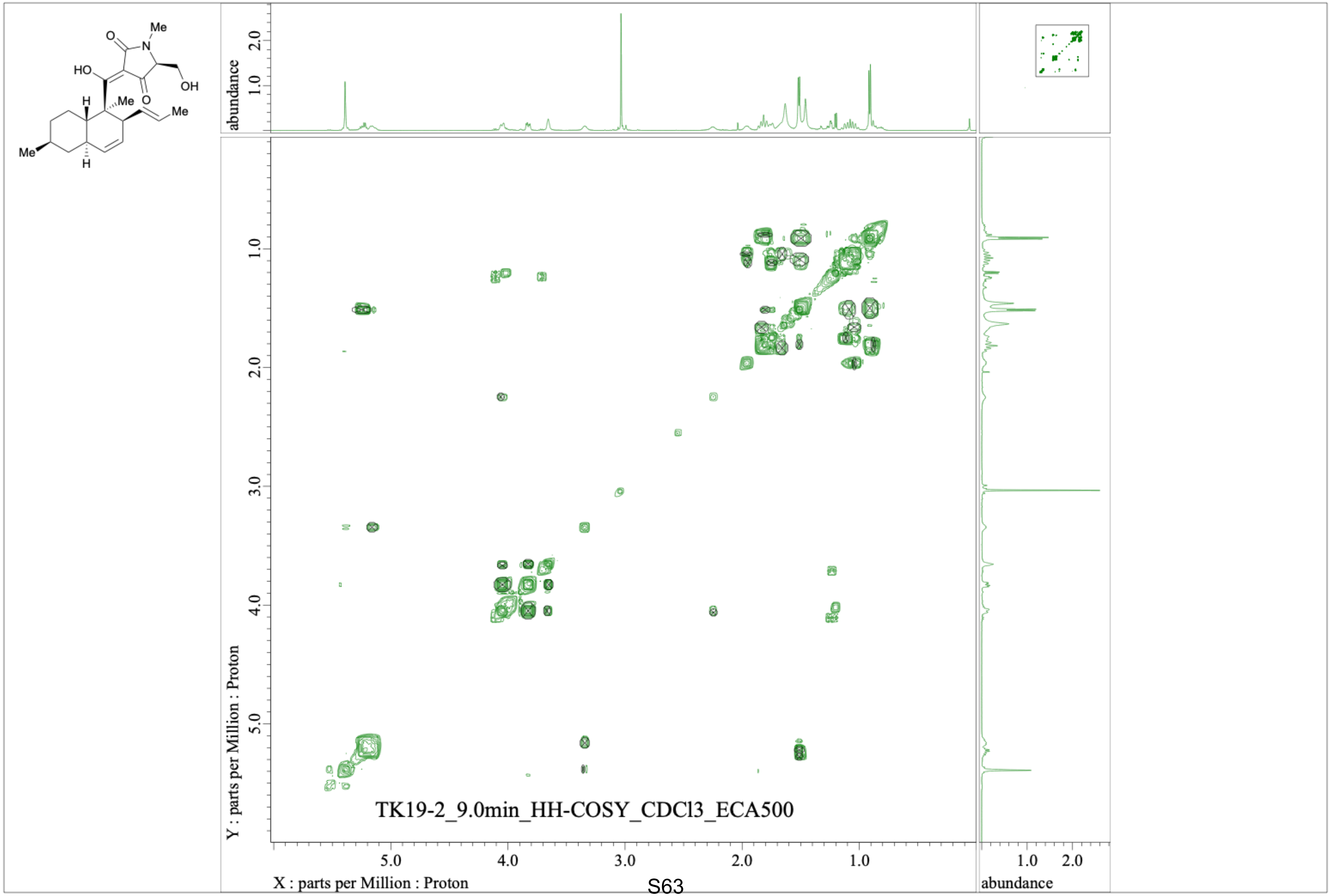

Figure S35. HSQC spectrum of Compound 2Aa in $CDCl_3$

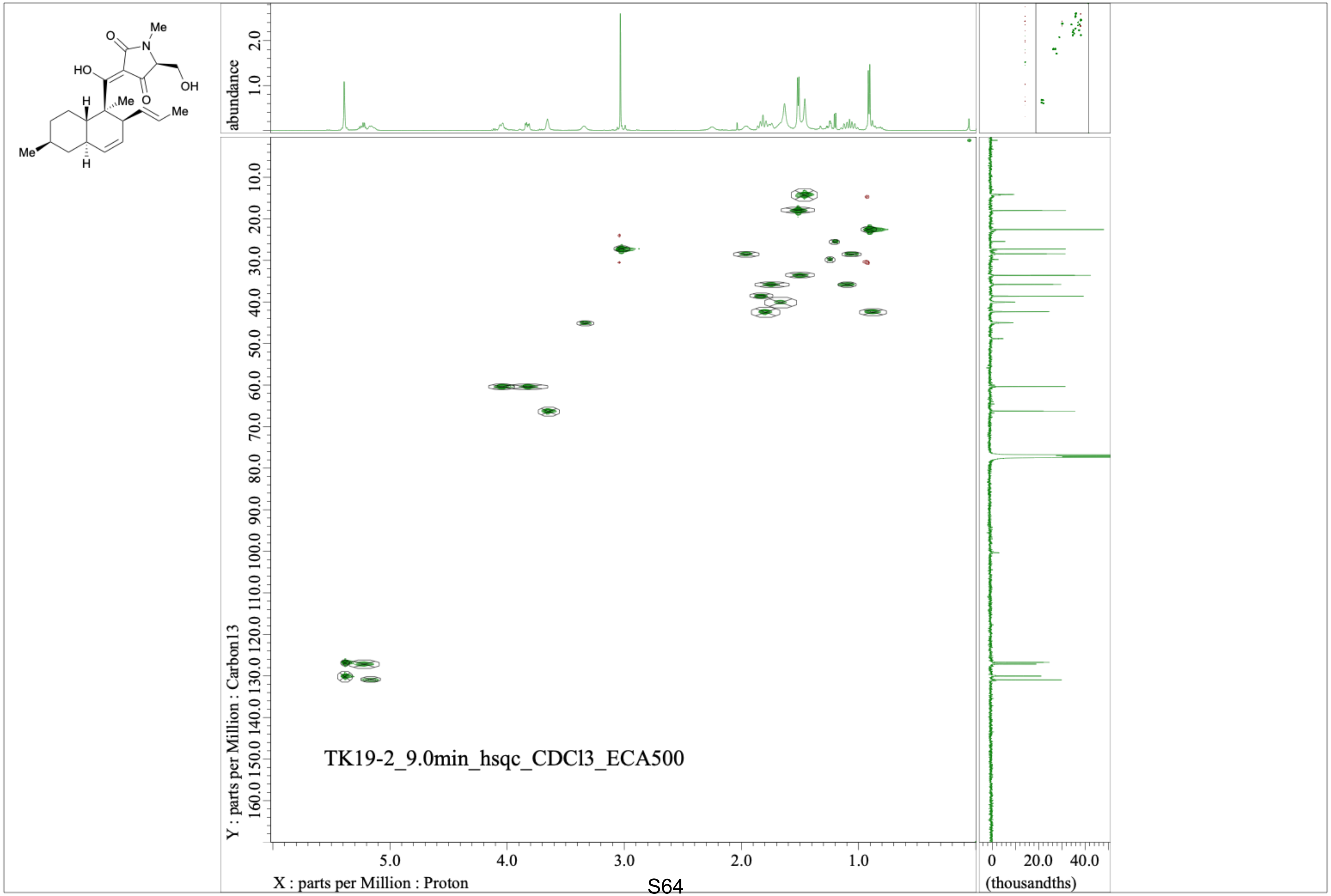

Figure 36. HMBC spectrum of Compound 2Aa in CDCl$_3$

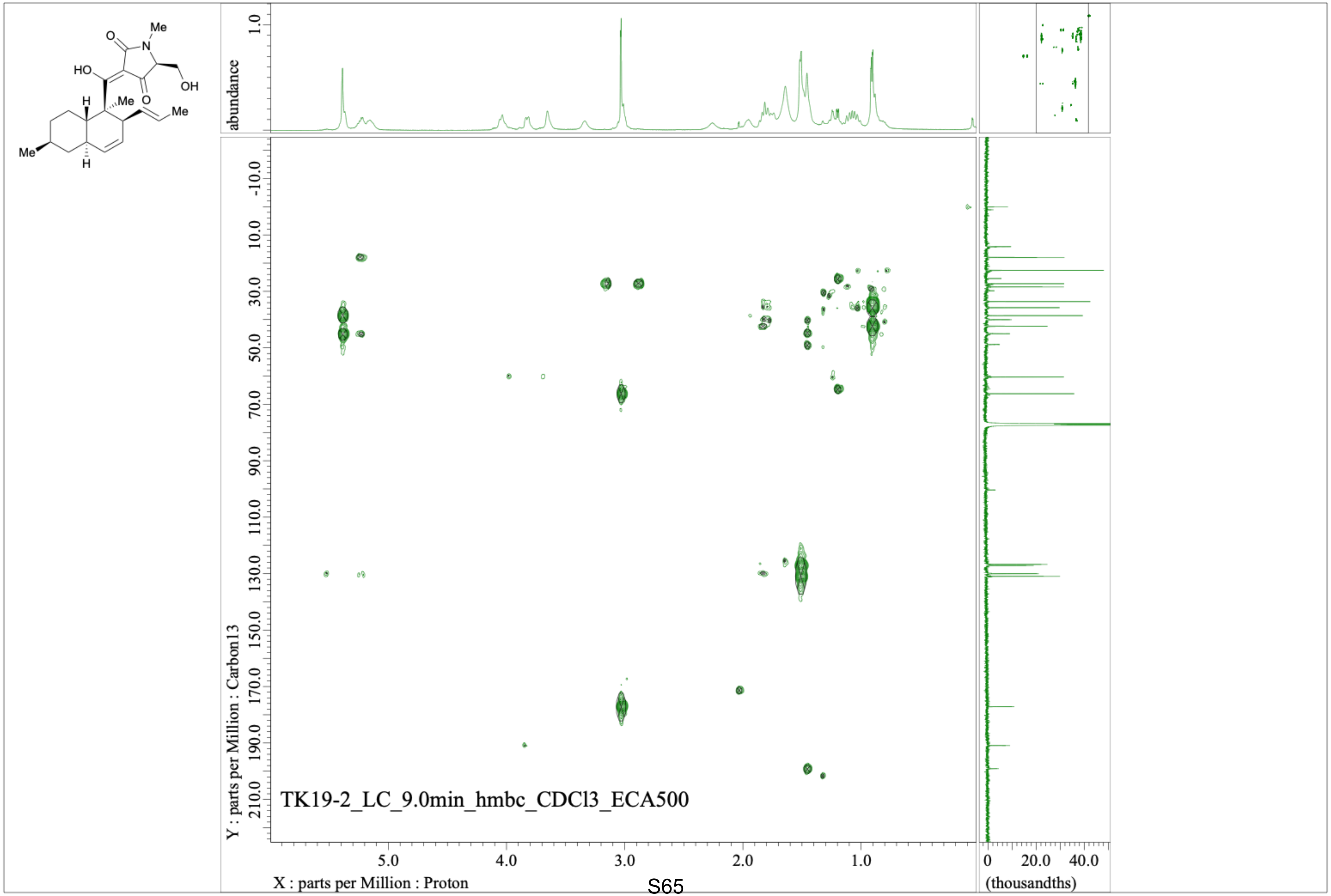

Figure S37. NOESY spectrum of Compound 2Aa in $CDCl_3$

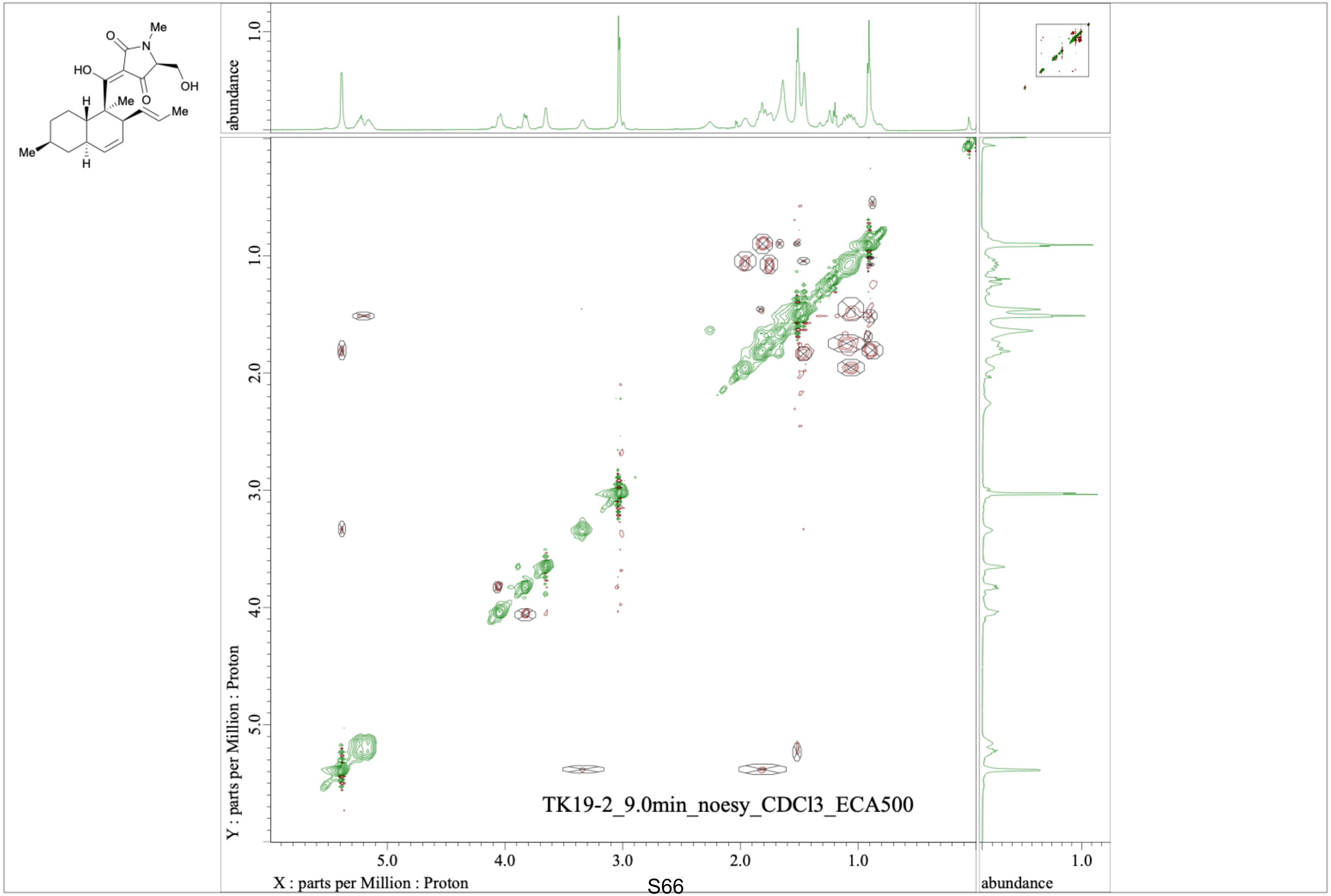

Figure S38. ^{1}H NMR spectrum of Compound 2Ab in $CDCl_3$ (600 MHz)

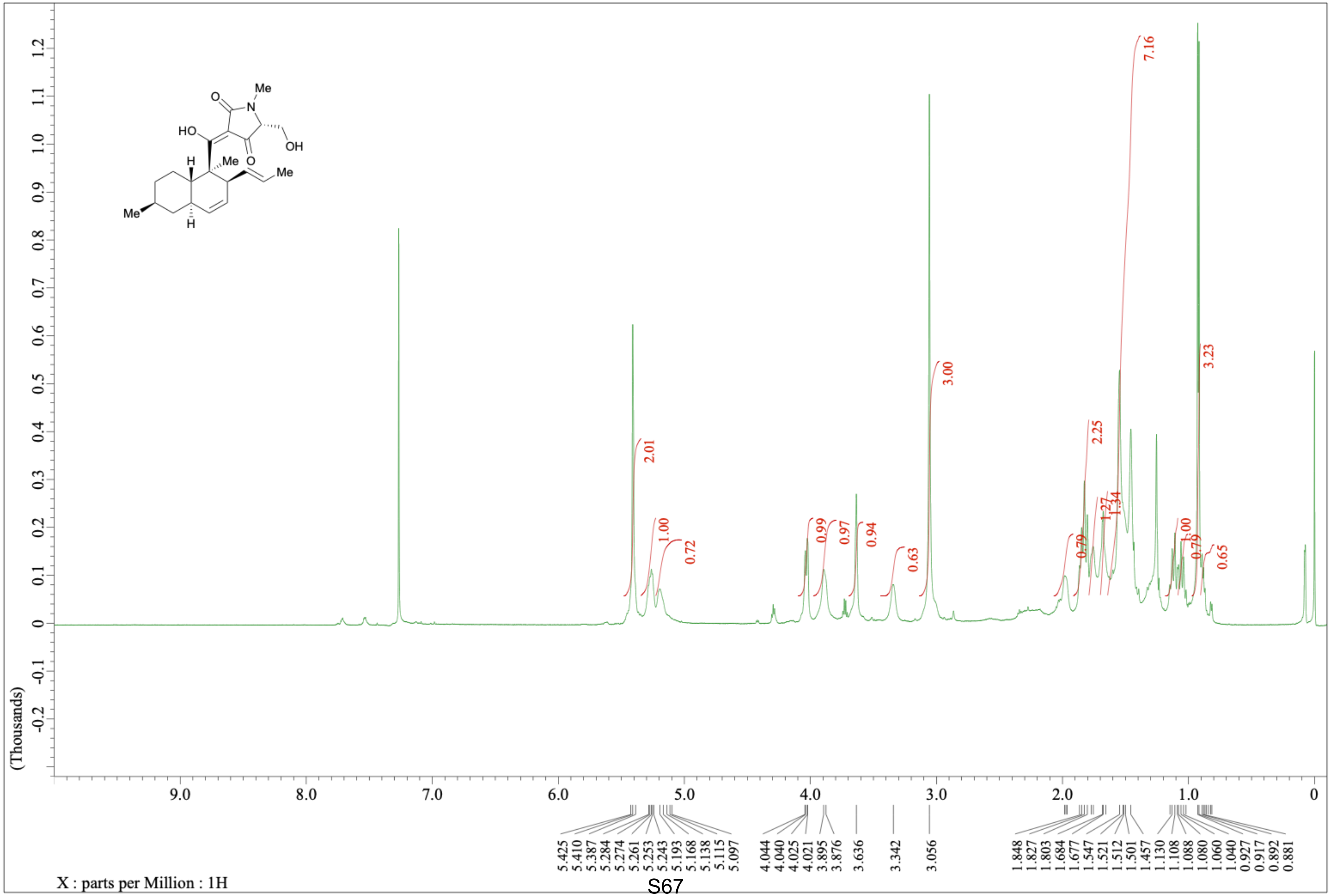

Figure S39. ***^{13}C NMR spectrum of Compound 2Ab in $CDCl_3$ (125 MHz)***

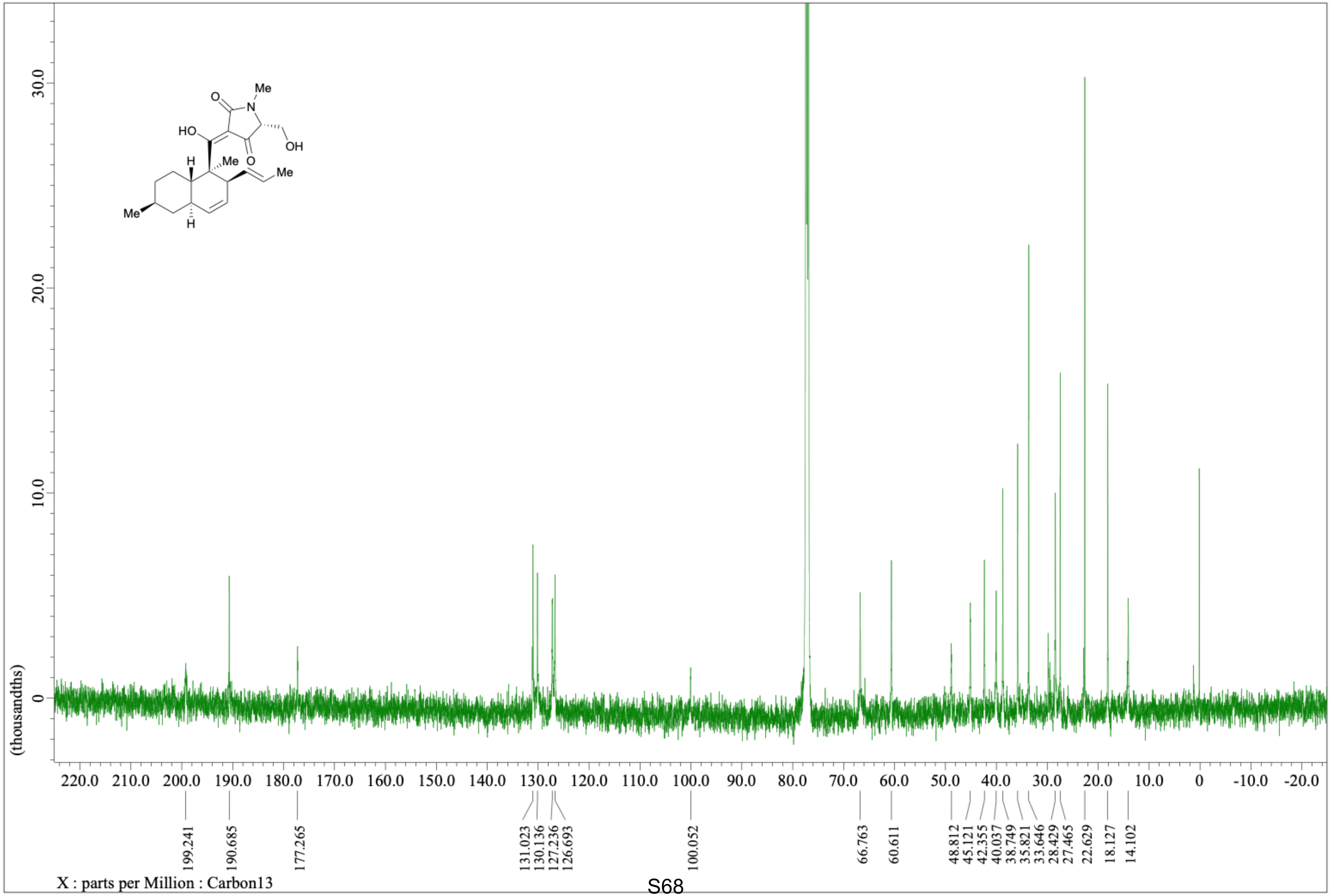

Figure S40. HH-COSY spectrum of Compound 2Ab in CDCl₃

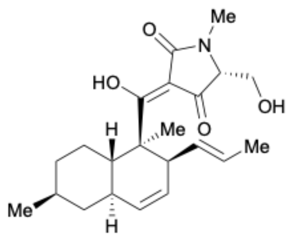

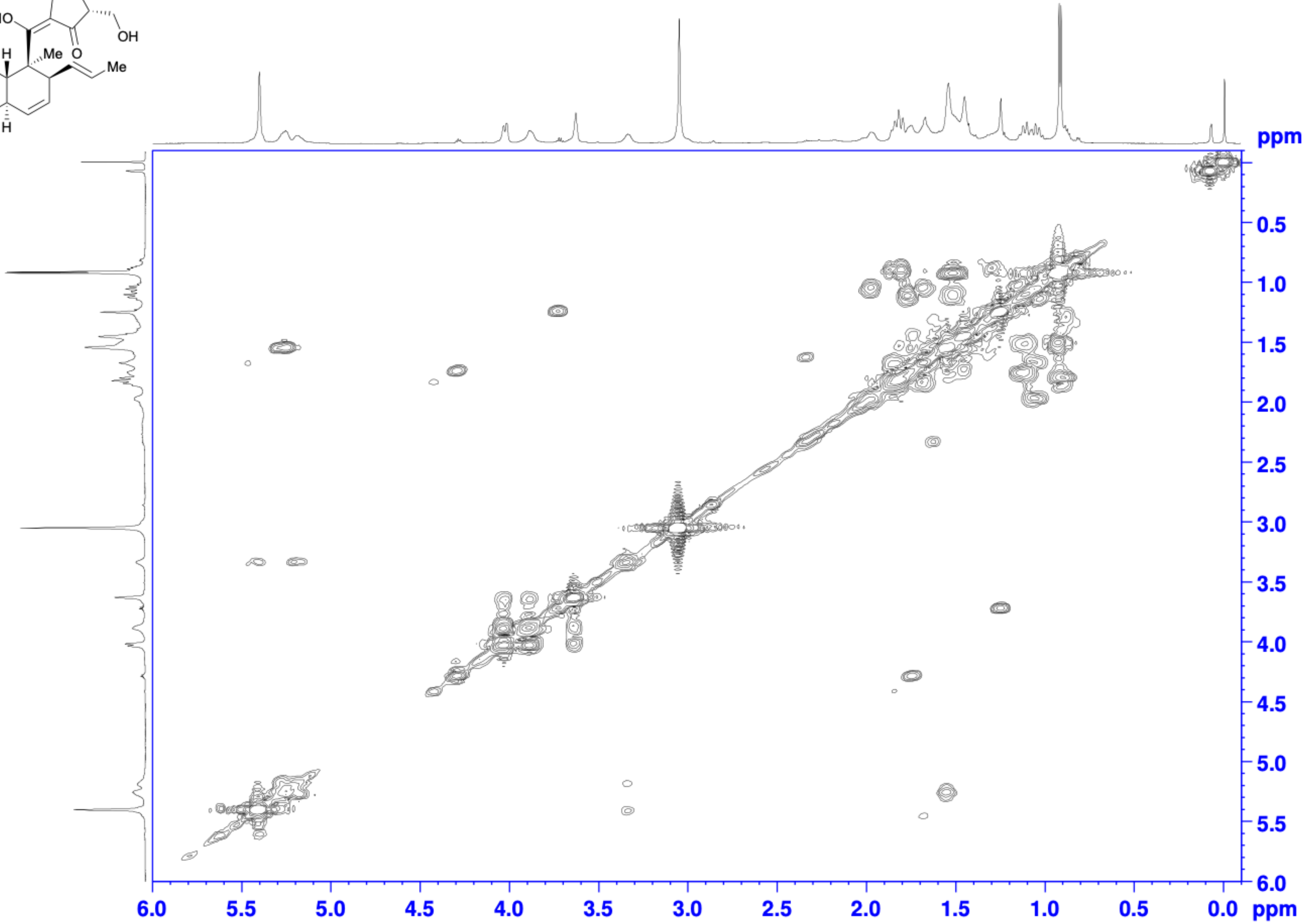

Figure S41. HMQC spectrum of Compound 2Ab in $CDCl_3$

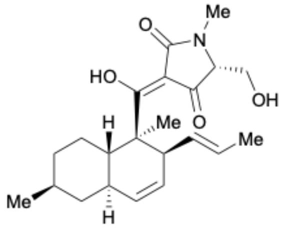

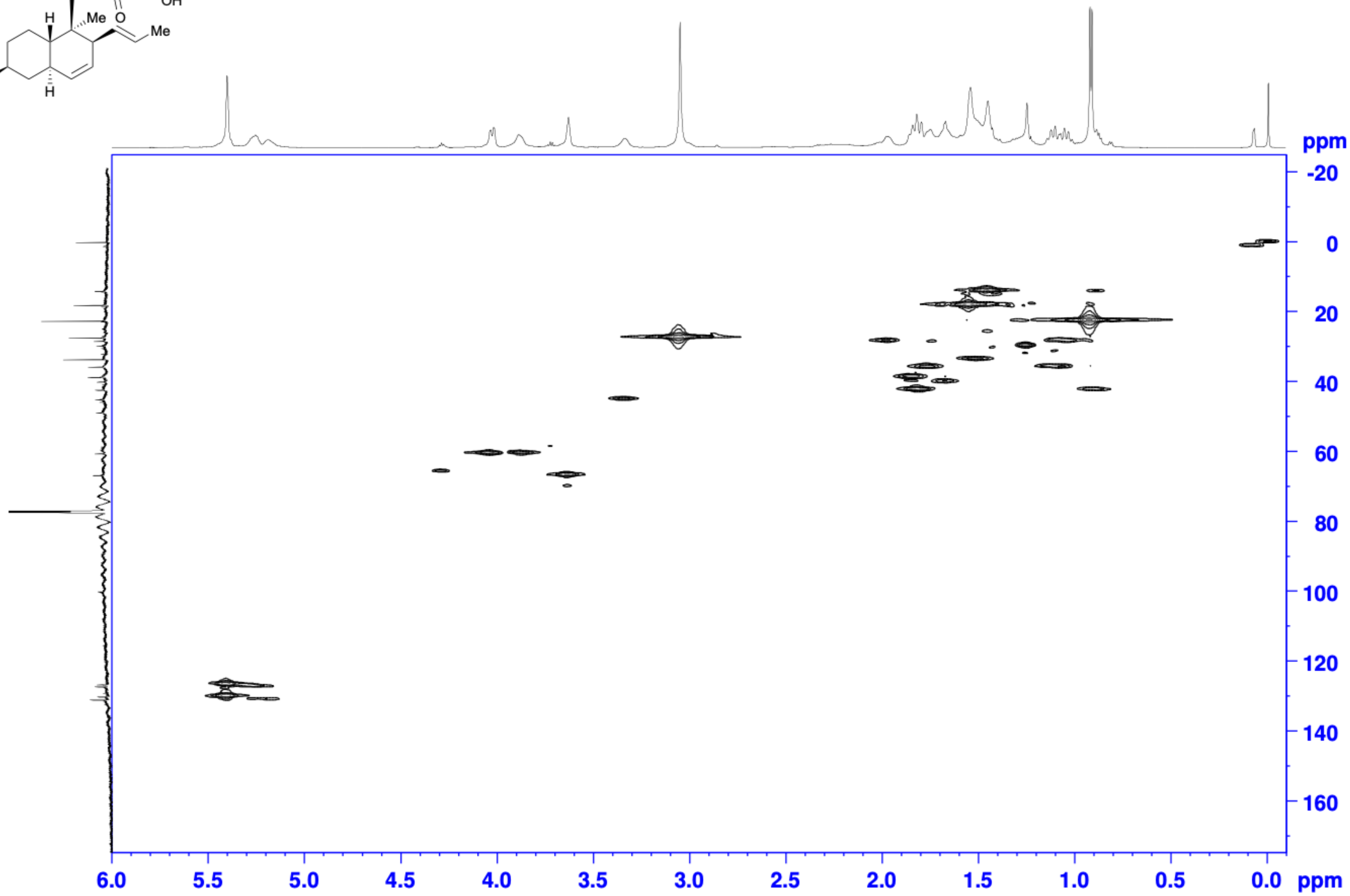

Figure S42. HMBC spectrum of Compound 2Ab in CDCl₃

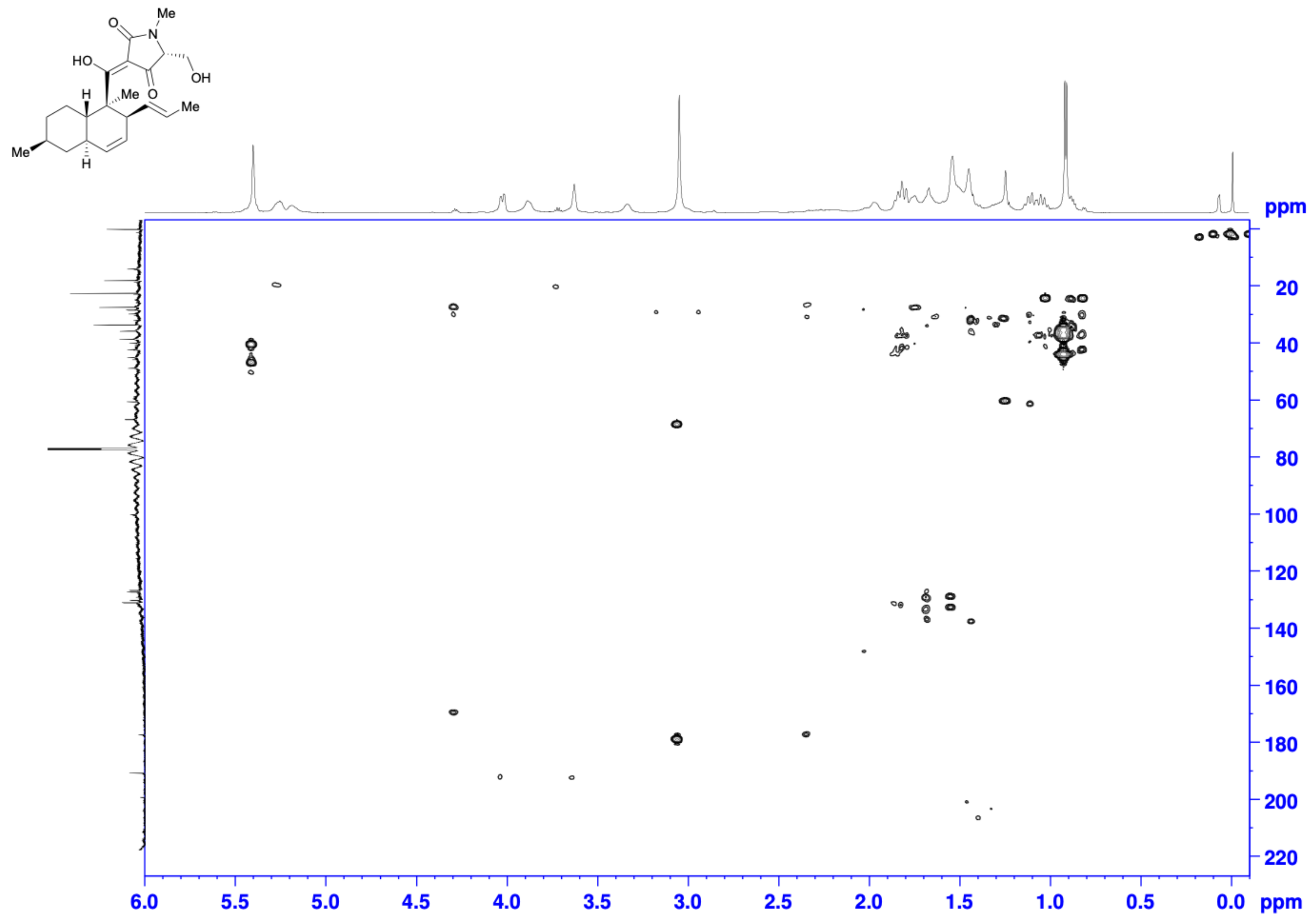

Figure S43. NOESY spectrum of Compound 2Ab in $CDCl_3$

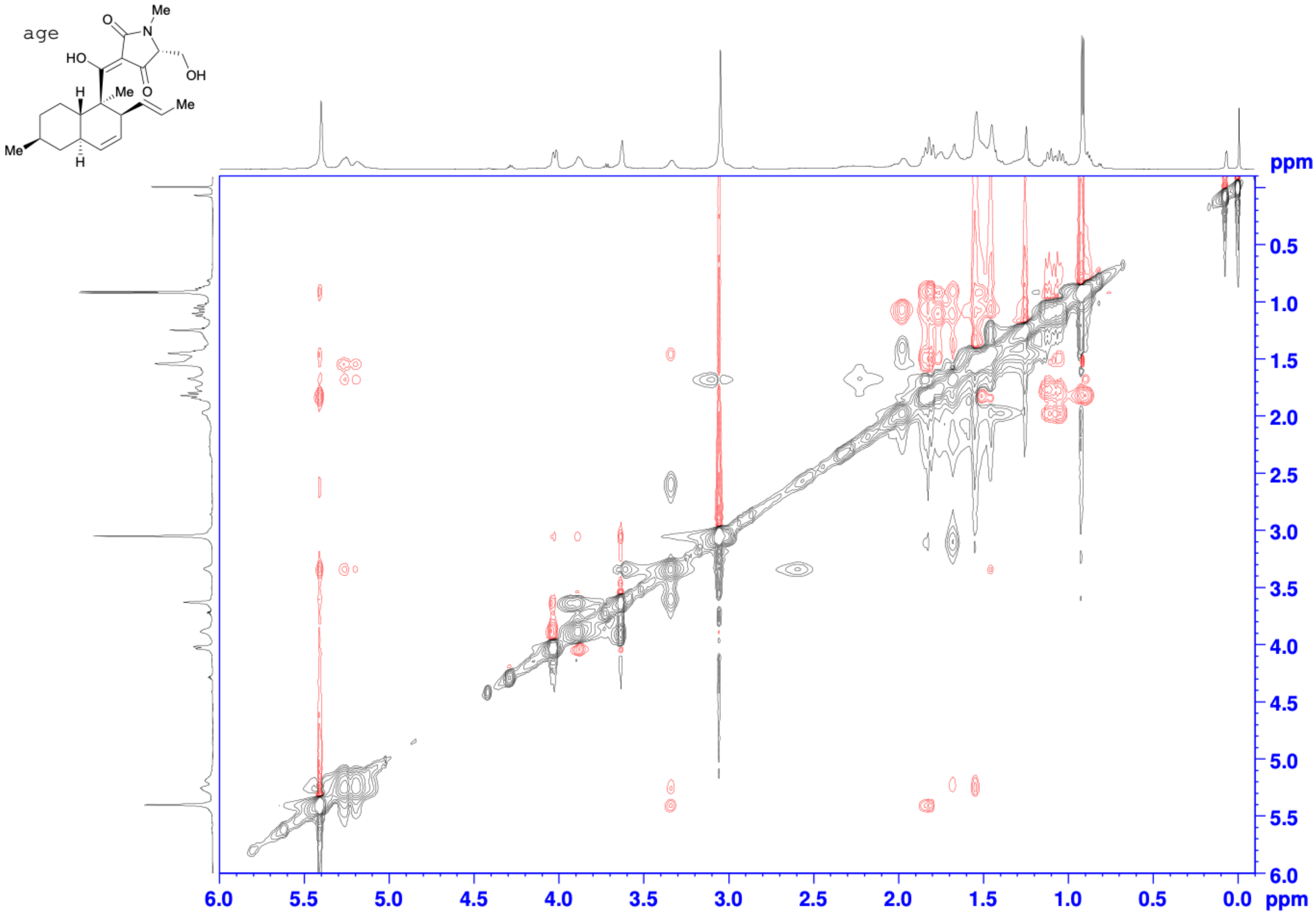

Figure S44. ^{1}H NMR spectrum of Compound 1Ba in CDCl₃ (600 MHz)

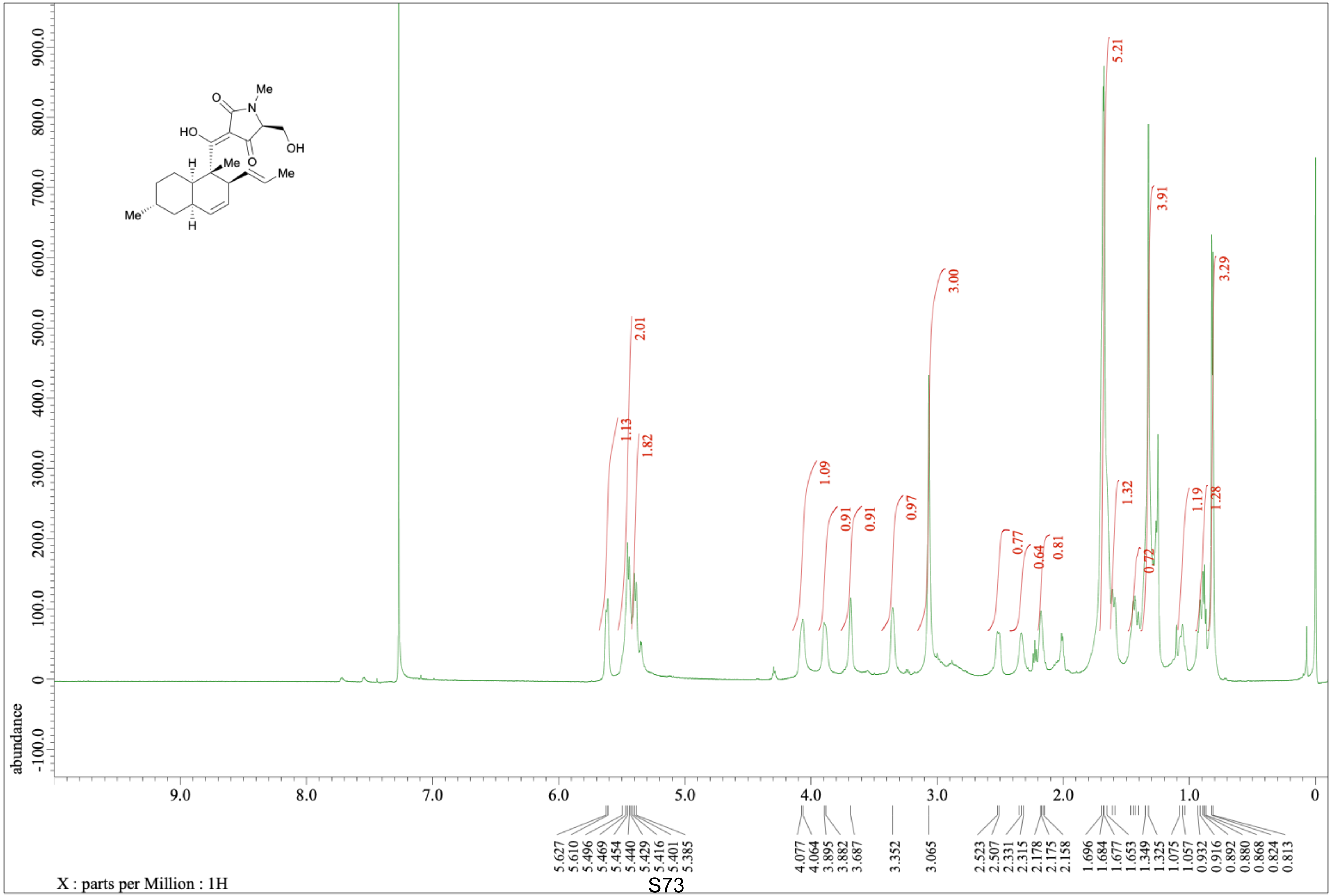

Figure S45. ^{13}C NMR spectrum of Compound 1Ba in $CDCl_3$ (150 MHz)

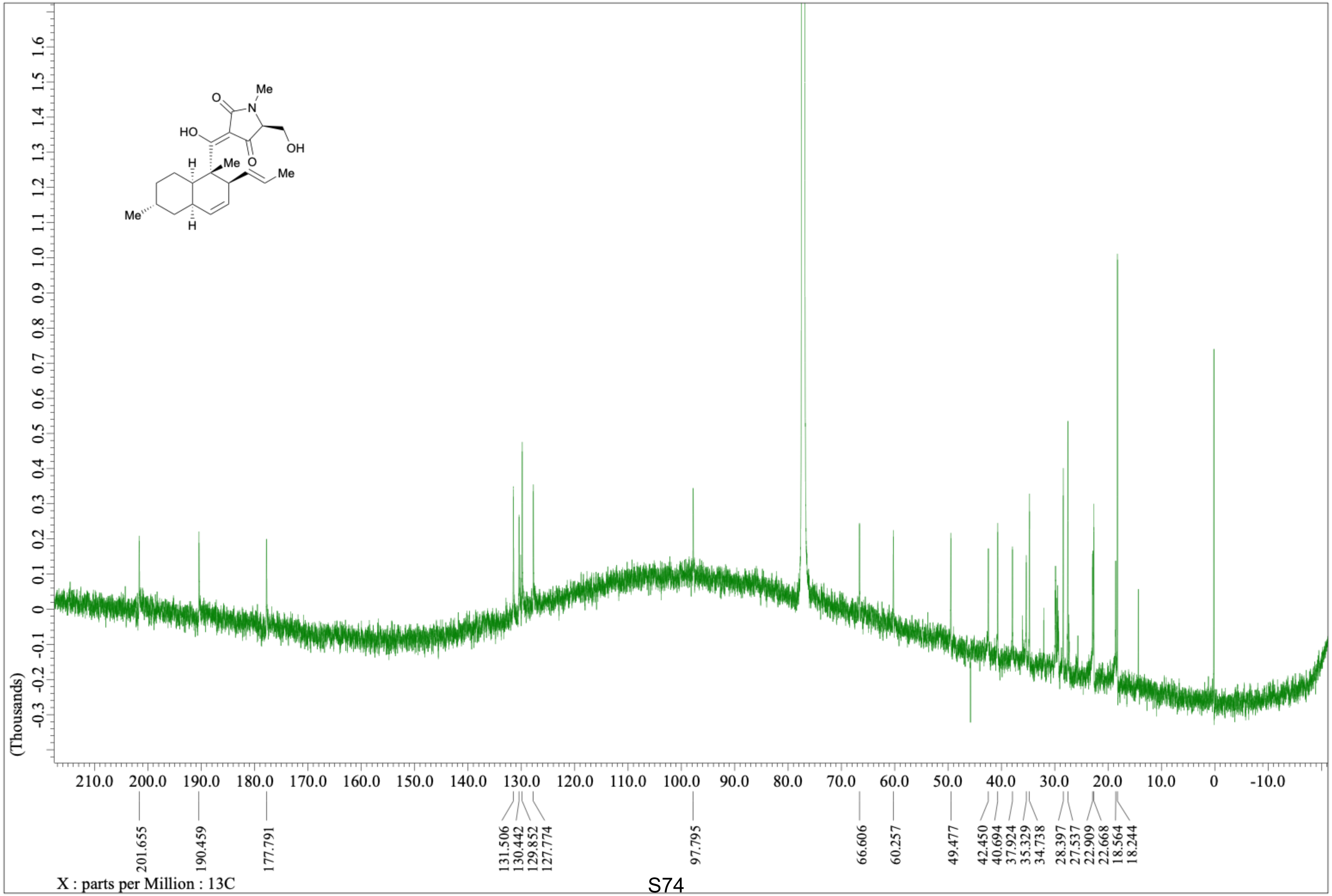

Figure S46. HH-COSY spectrum of Compound 1Ba in $CDCl_3$

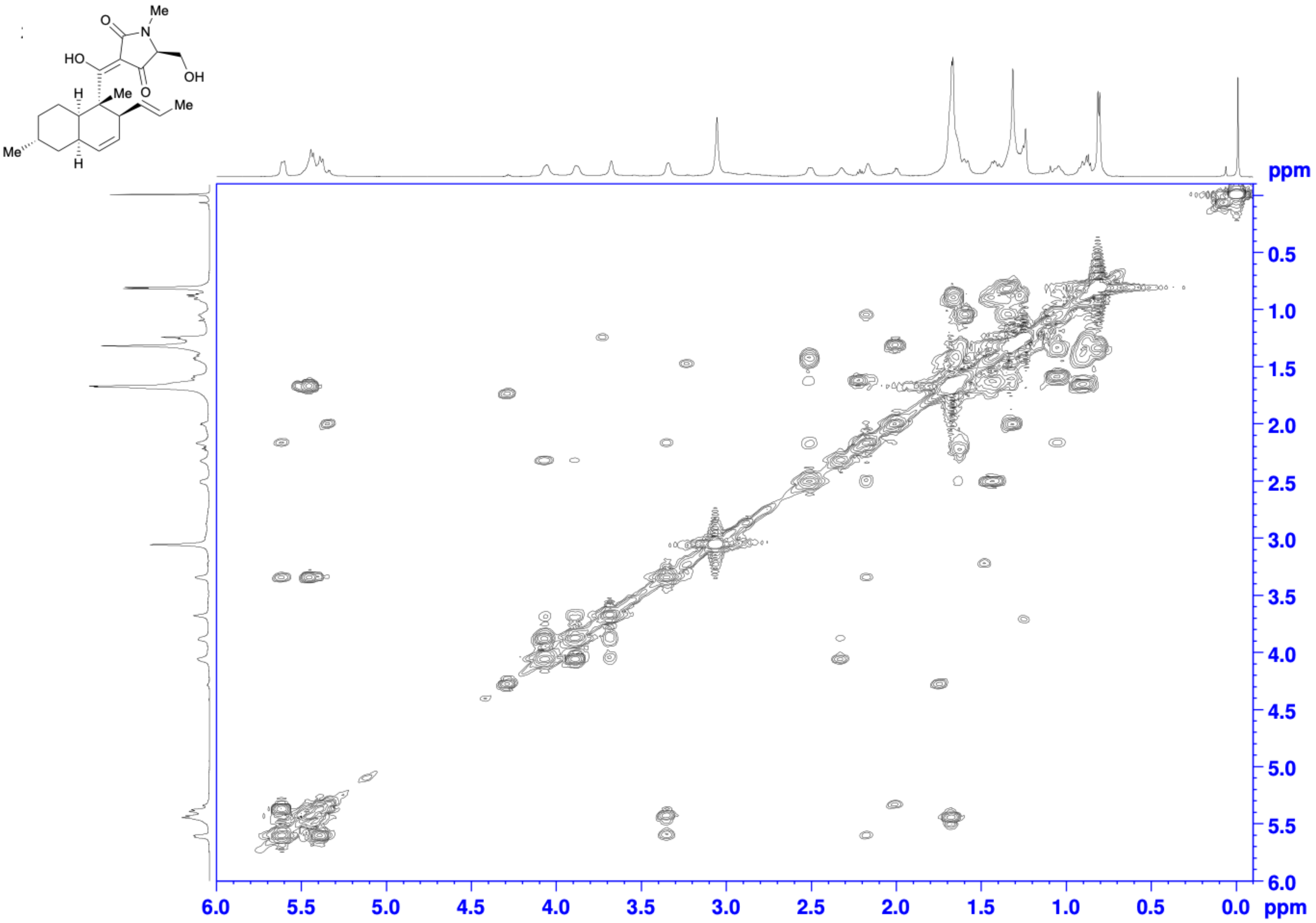

Figure S47. HMQC spectrum of Compound 1Ba in CDCl₃

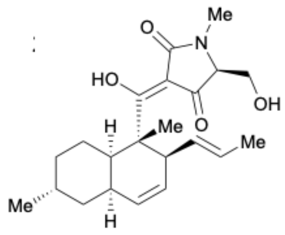

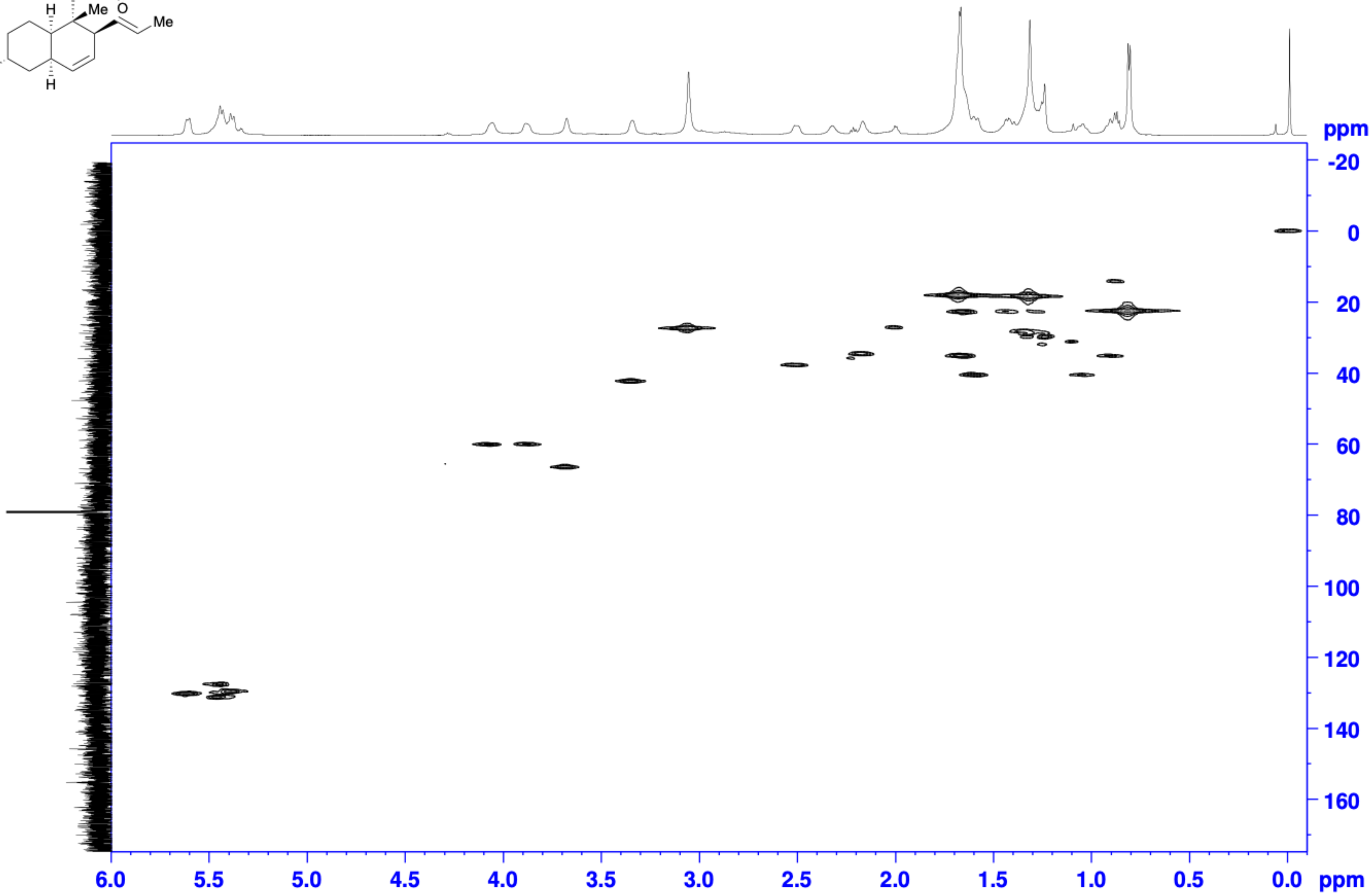

Figure S48. HMBC spectrum of Compound 1Ba in $CDCl_3$

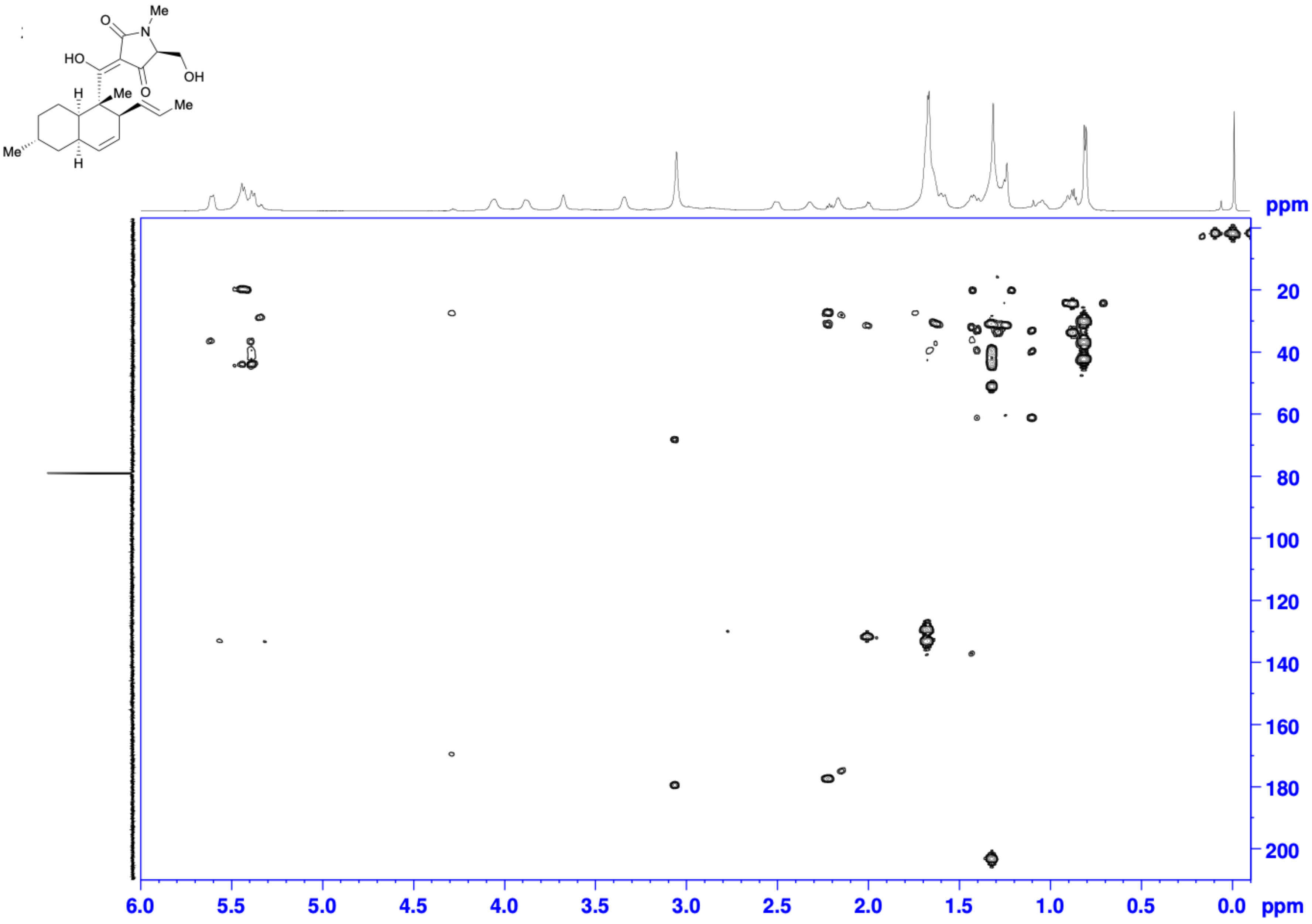

Figure S49. NOESY spectrum of Compound 1Ba in $CDCl_3$

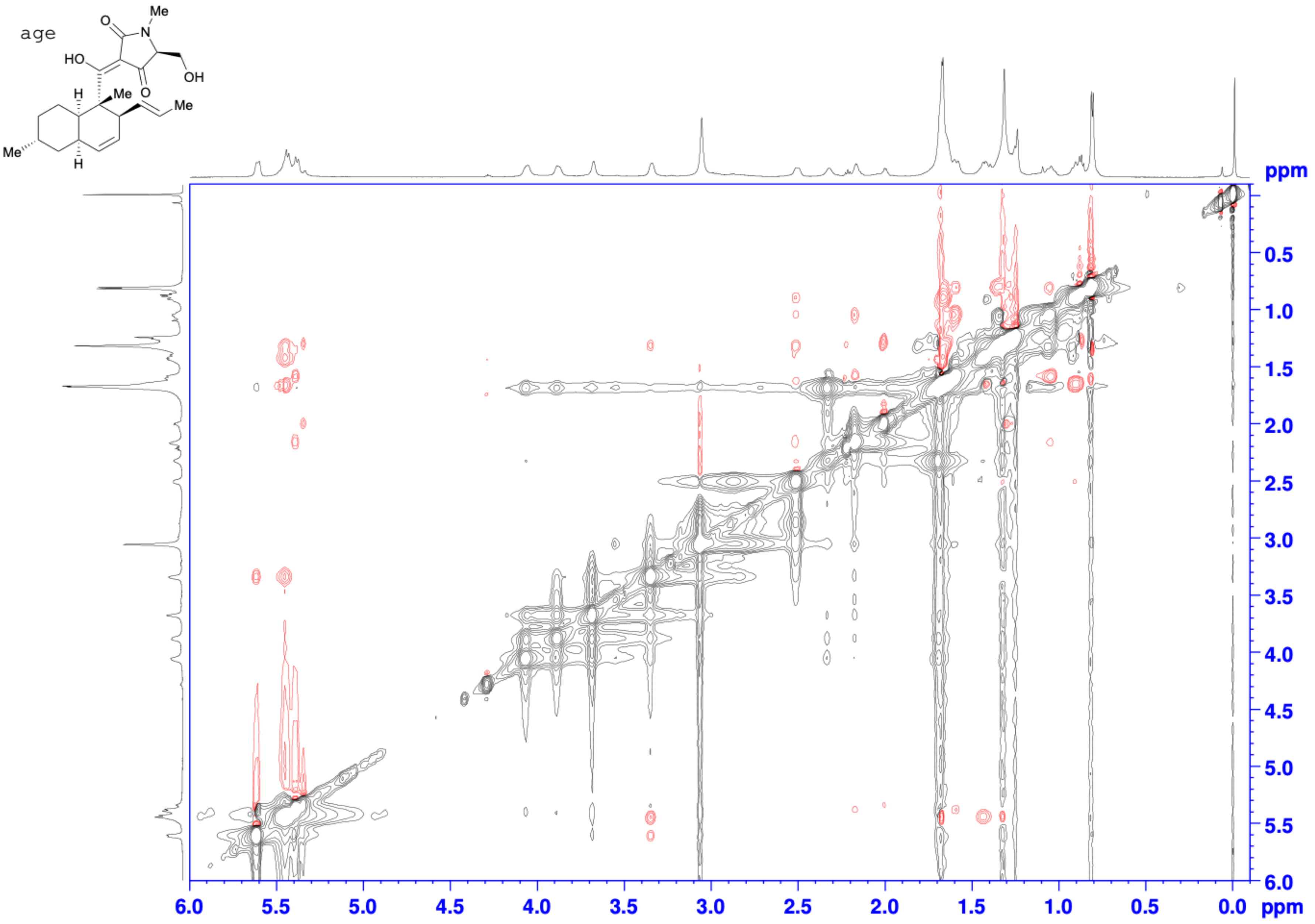

Figure S50. ¹H NMR spectrum of Compound 1Bb in CDCl₃ (600 MHz)

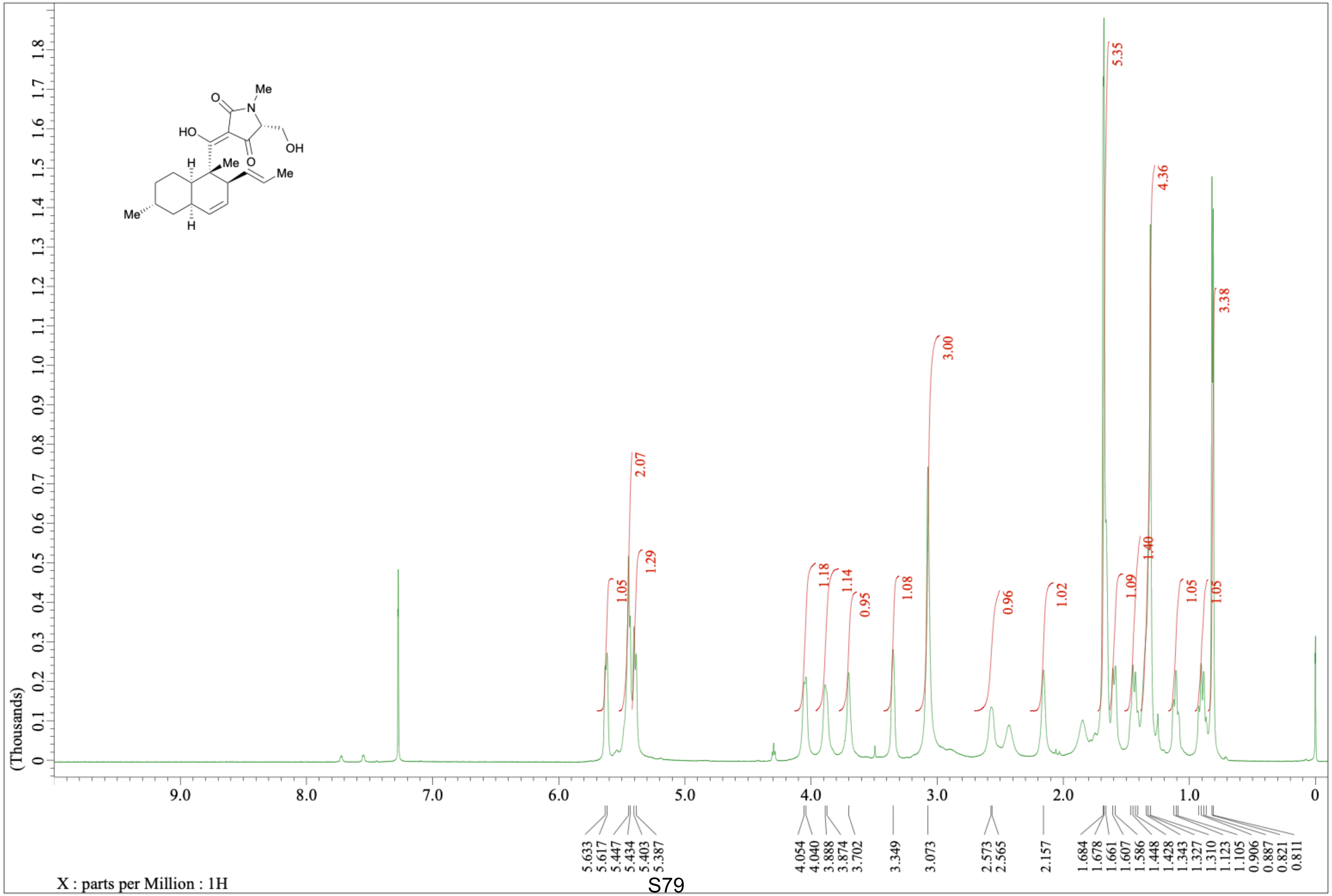

Figure S51. ^{13}*C NMR spectrum of Compound 1Bb in CDCl*$_3$ *(150 MHz)*

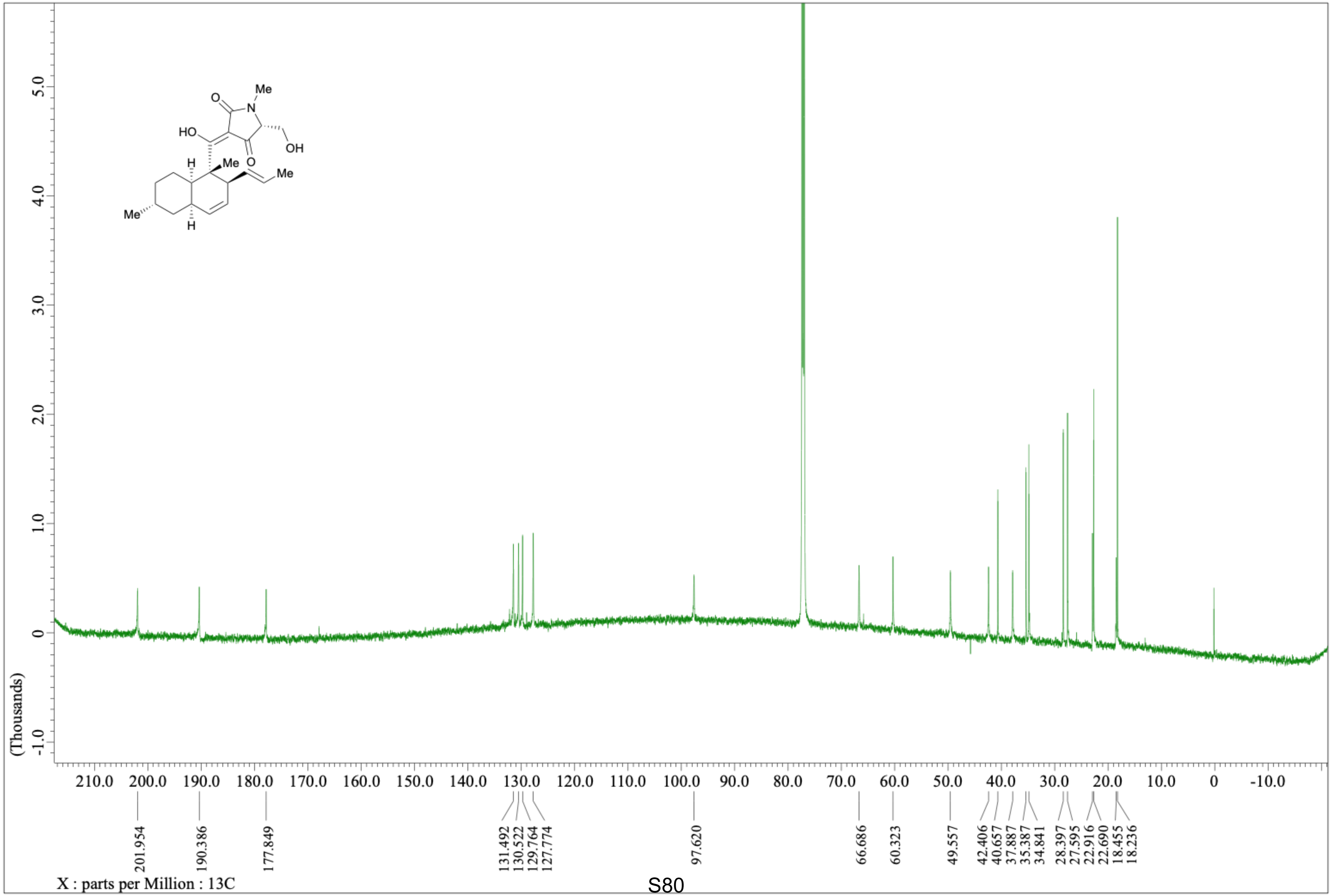

Figure S52. HH-COSY spectrum of Compound 1Bb in $CDCl_3$

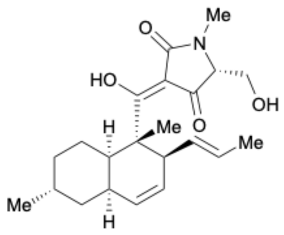

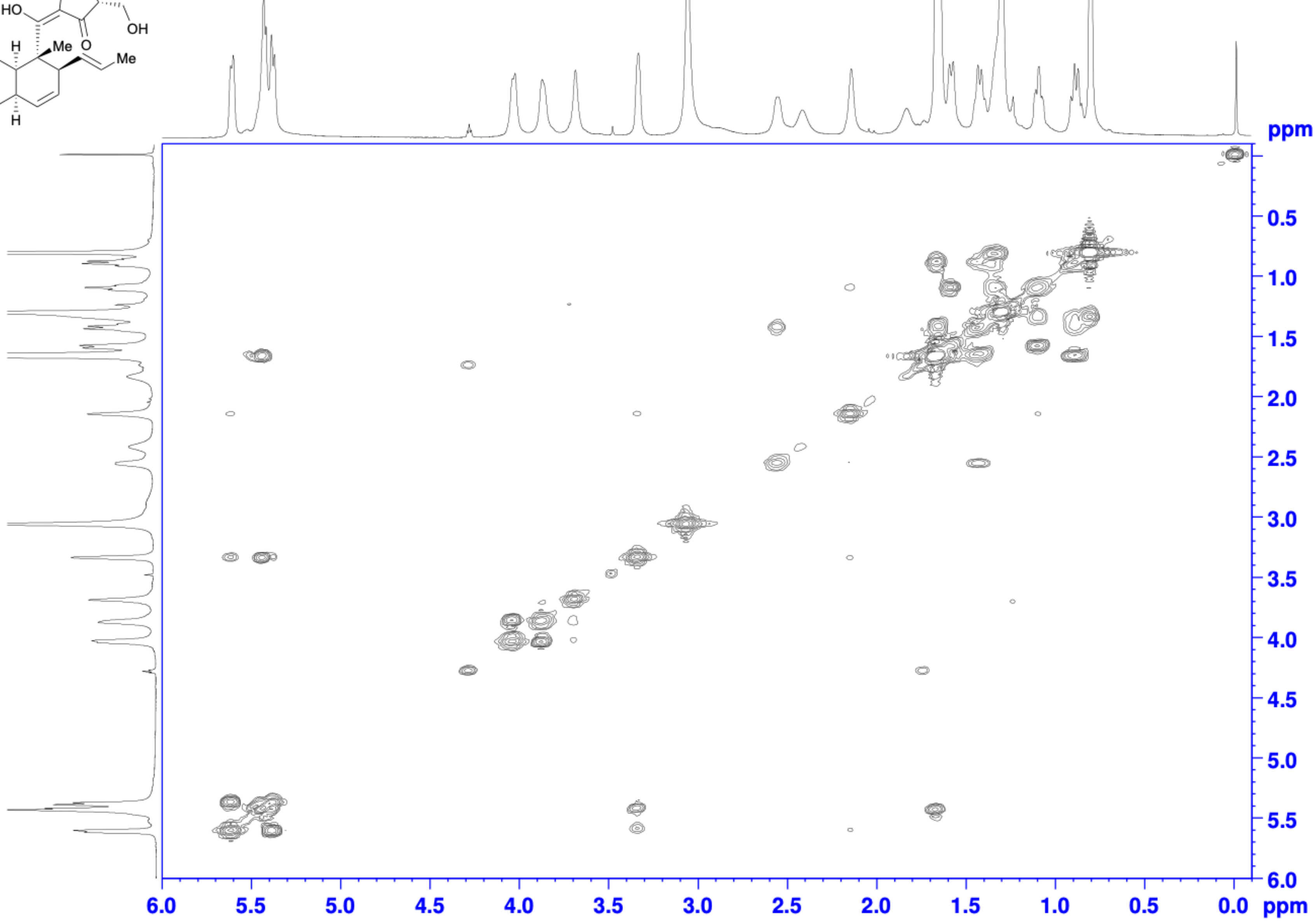

Figure S53. HMQC spectrum of Compound 1Bb in $CDCl_3$

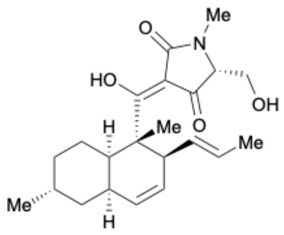

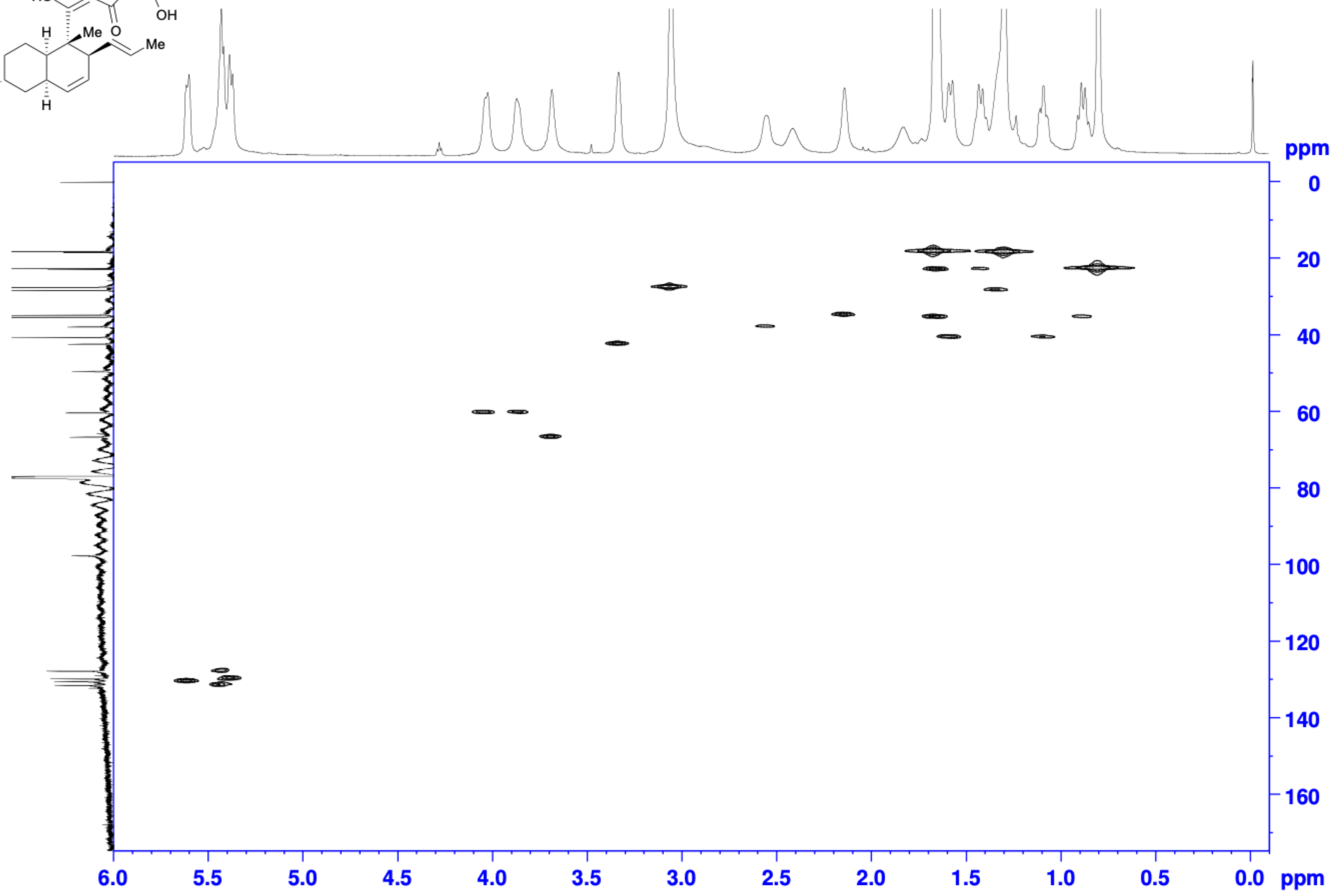

Figure S54. HMBC spectrum of Compound 1Bb in $CDCl_3$

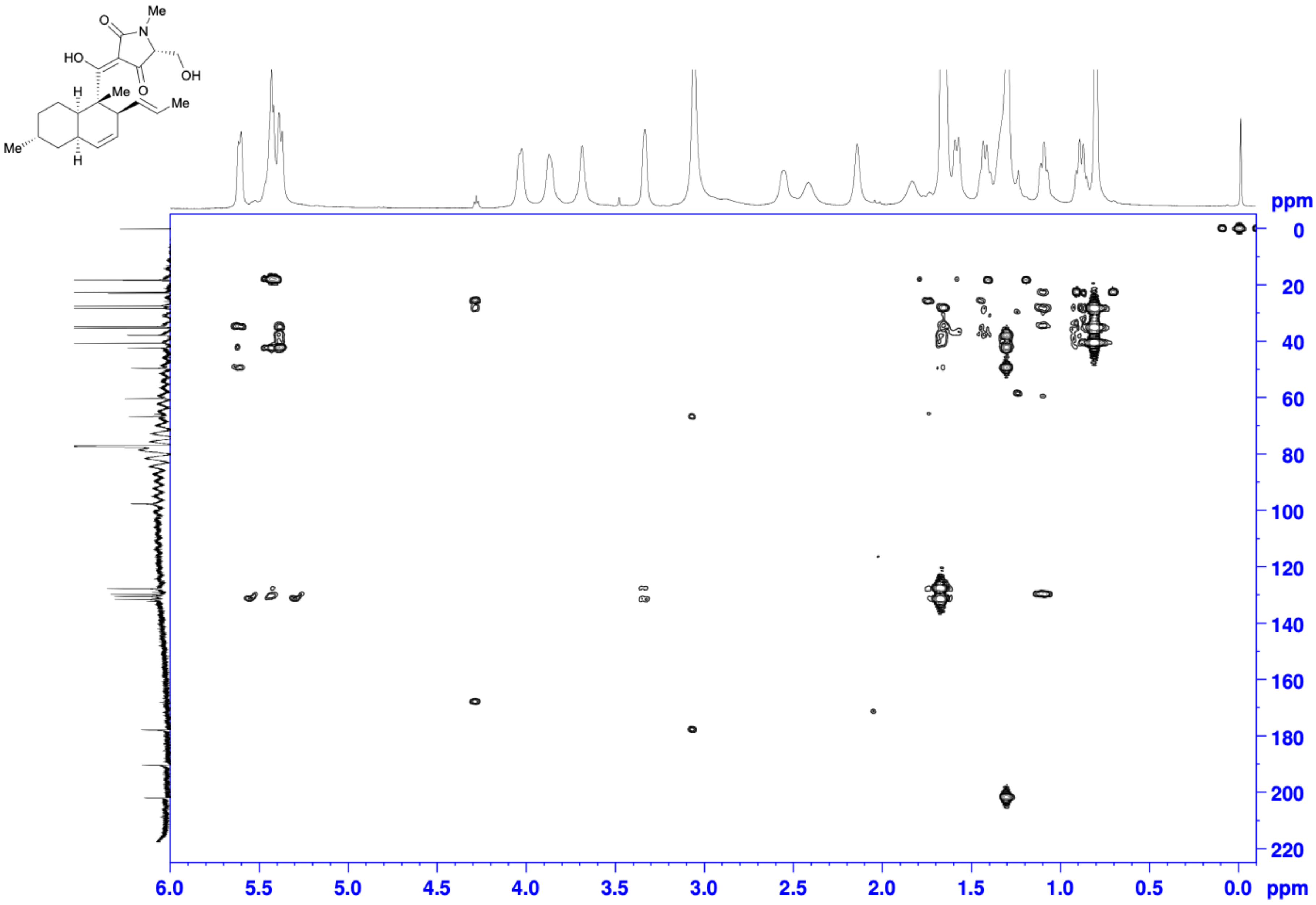

Figure S55. NOESY spectrum of Compound 1Bb in $CDCl_3$

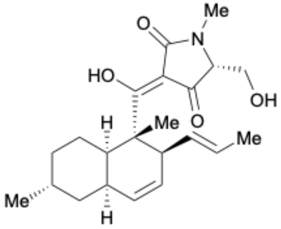

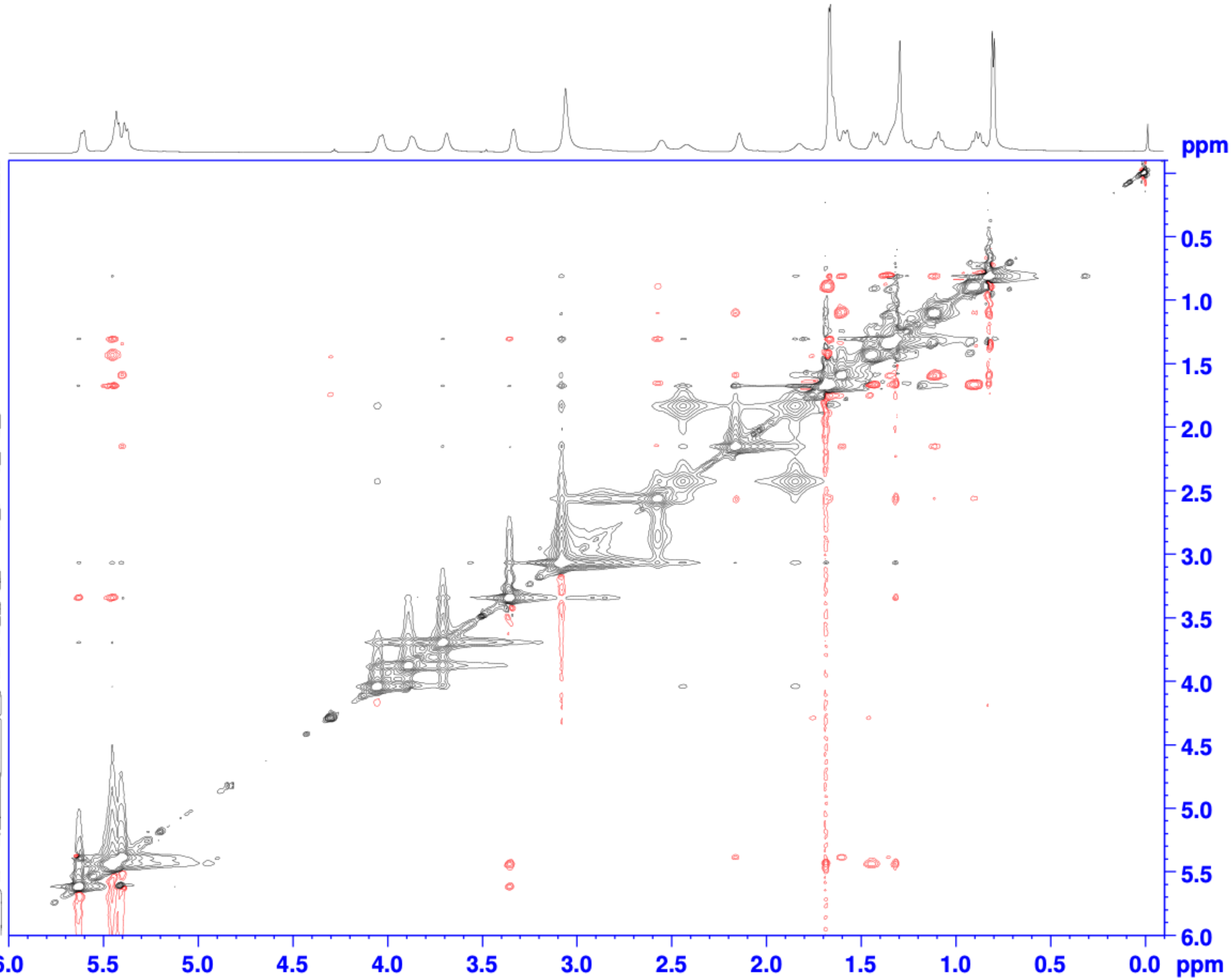

Figure S56. ^{1}H NMR spectrum of Compound 2Ba in CDCl$_3$ (500 MHz)

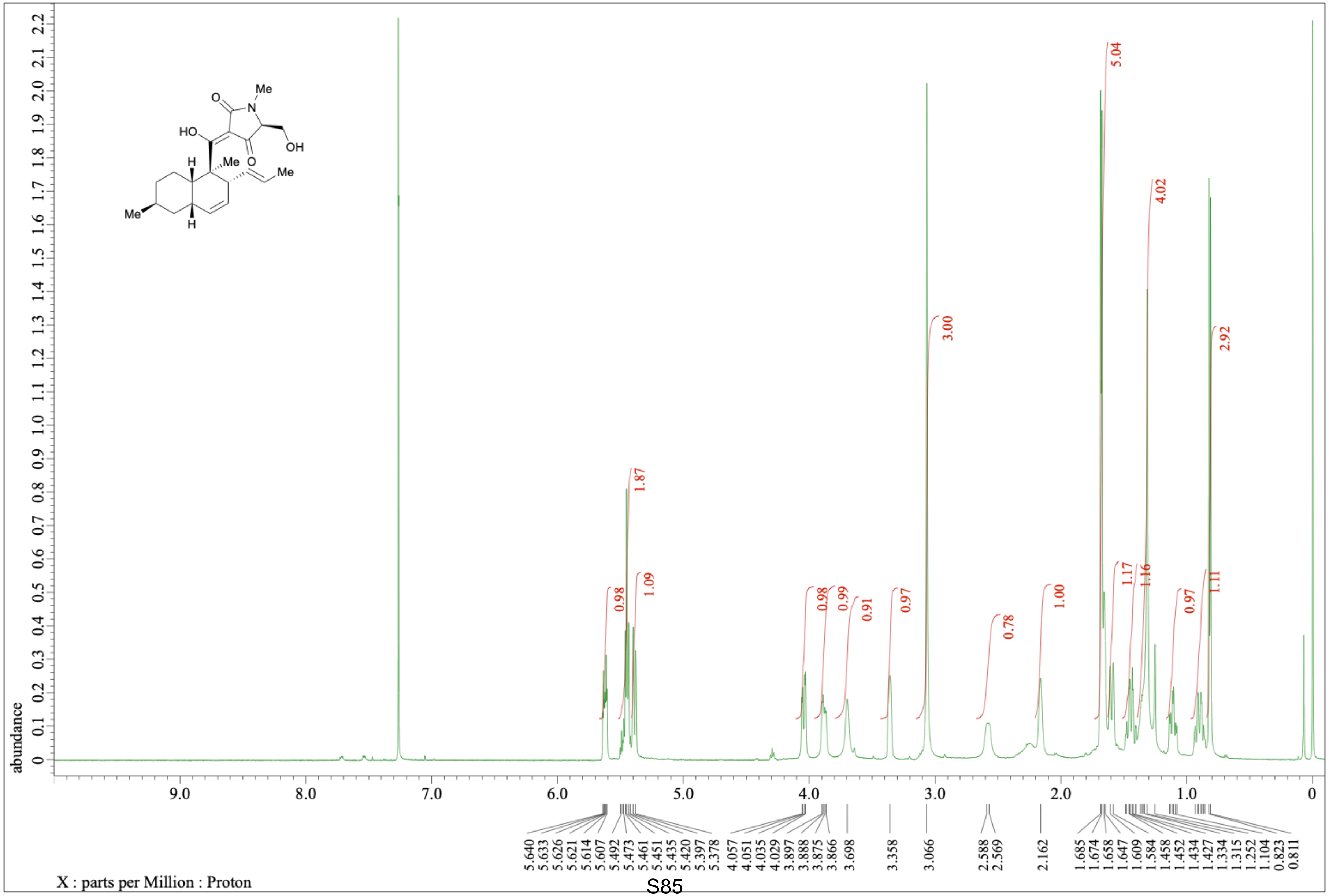

Figure S57. ^{13}C NMR spectrum of Compound 2Ba in $CDCl_3$ (125 MHz)

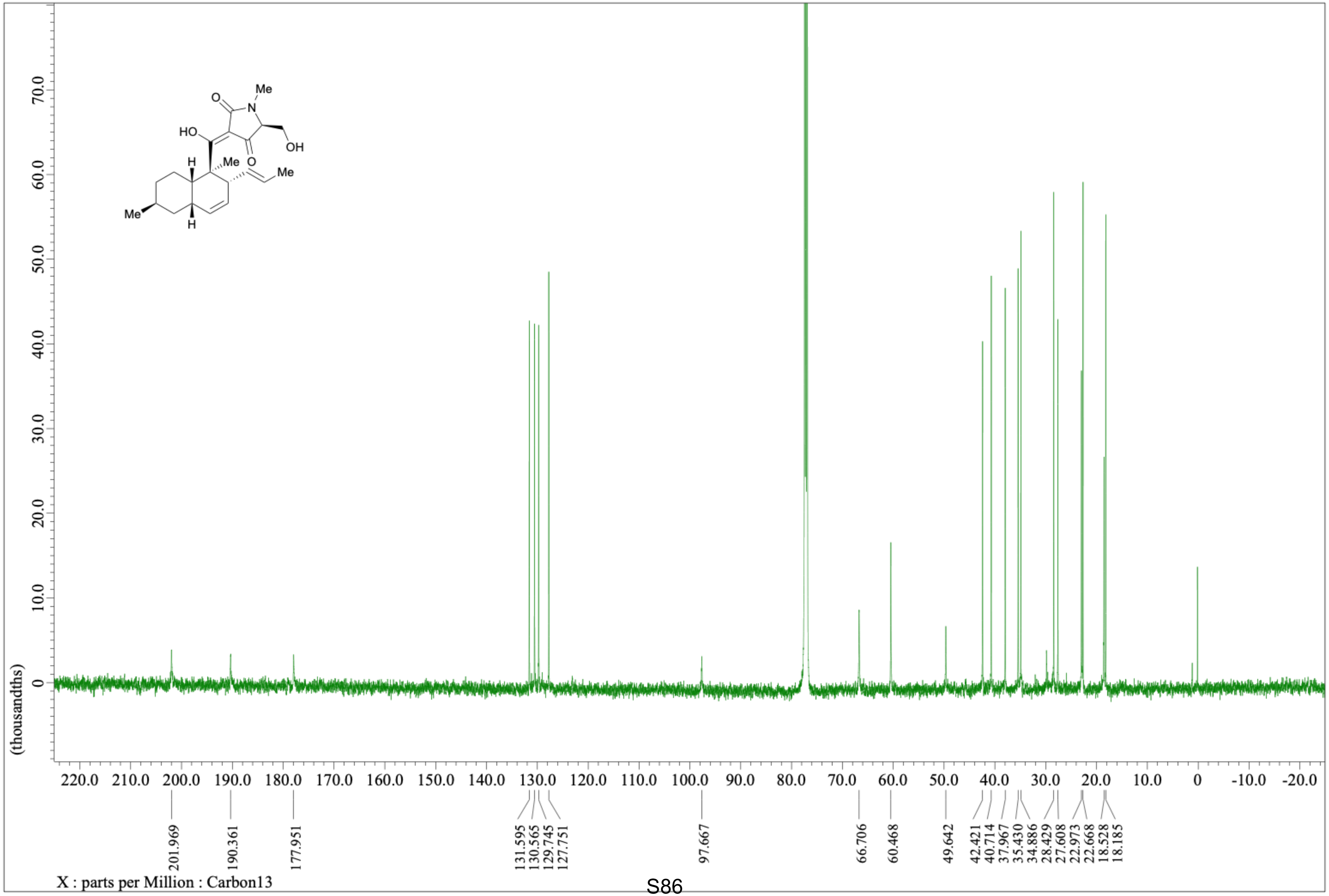

Figure S58. HH-COSY spectrum of Compound 2Ba in $CDCl_3$

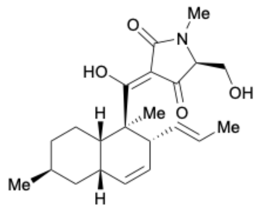

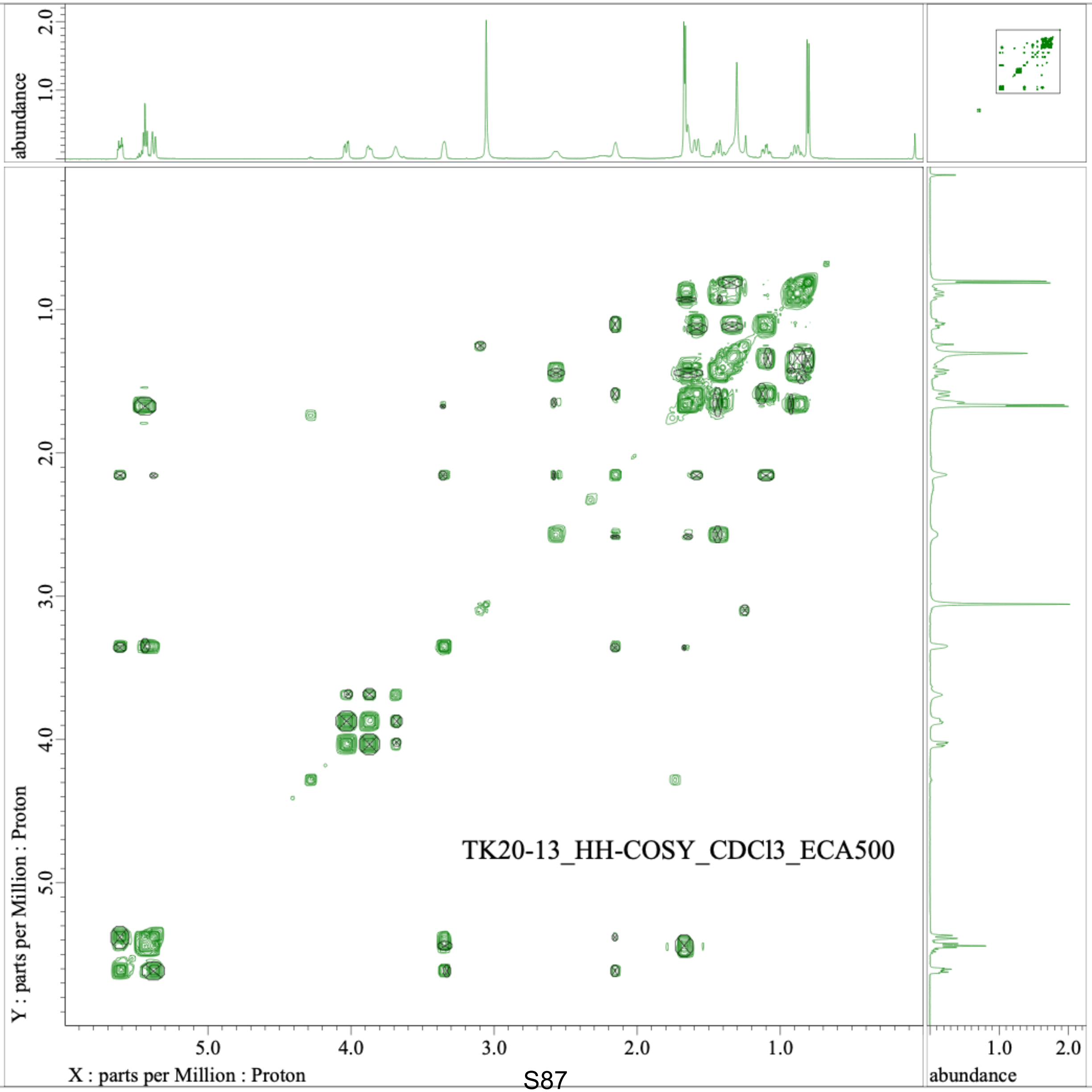

Figure S59. HSQC spectrum of Compound 2Ba in $CDCl_3$

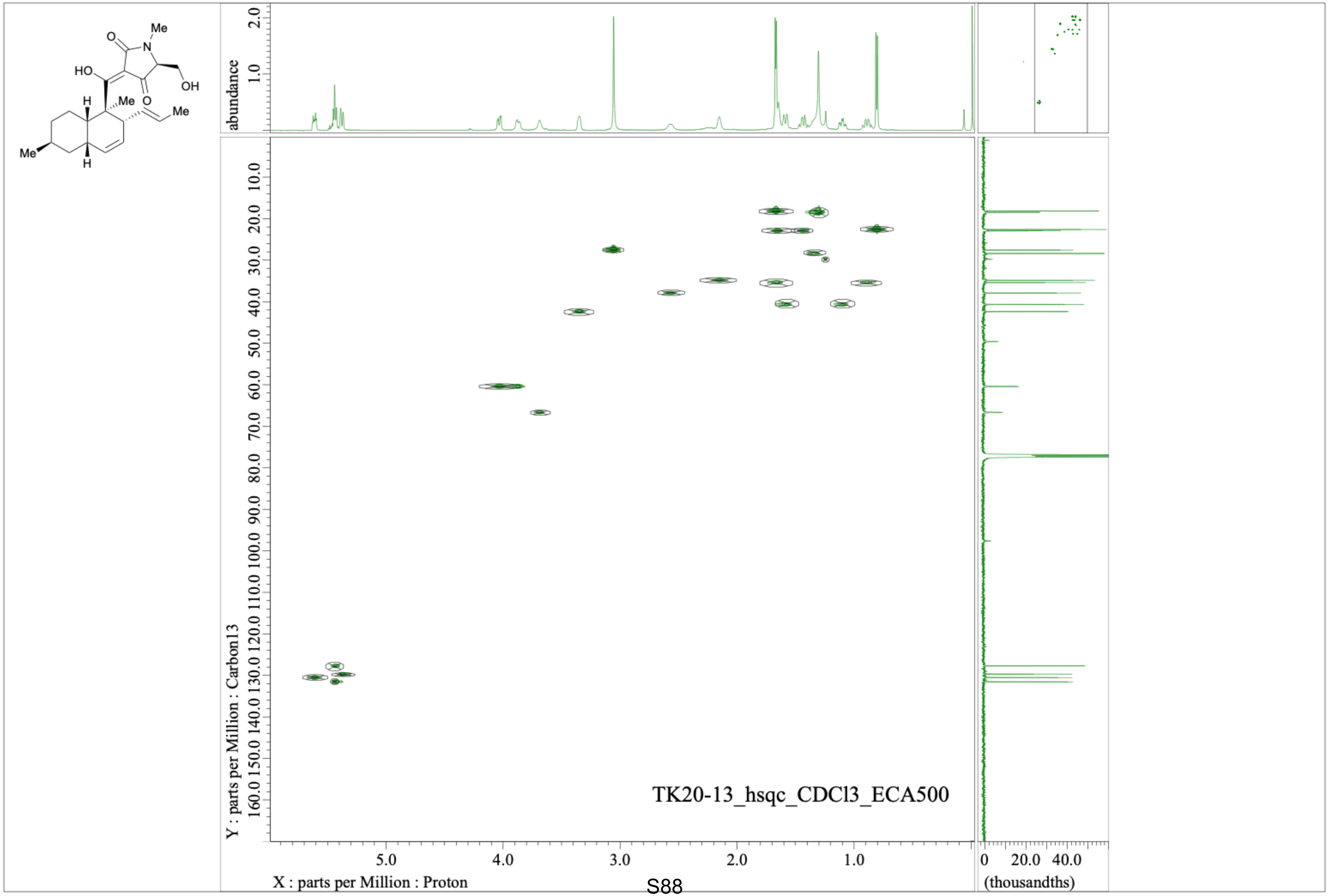

Figure S60. HMBC spectrum of Compound 2Ba in CDCl₃

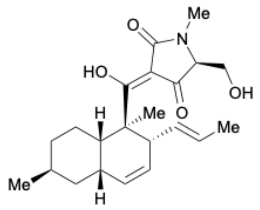

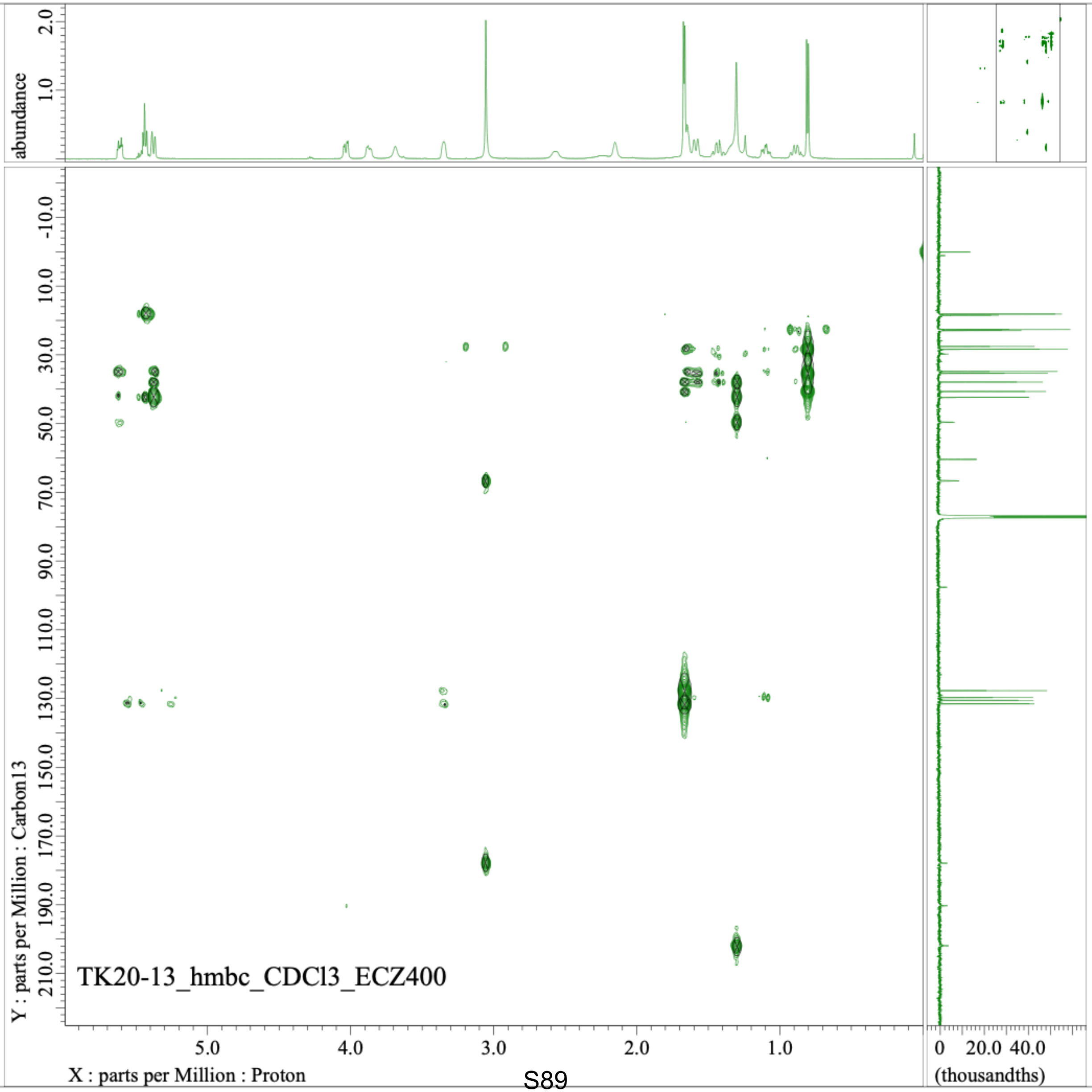

Figure S61. NOESY spectrum of Compound 2Ba in CDCl₃

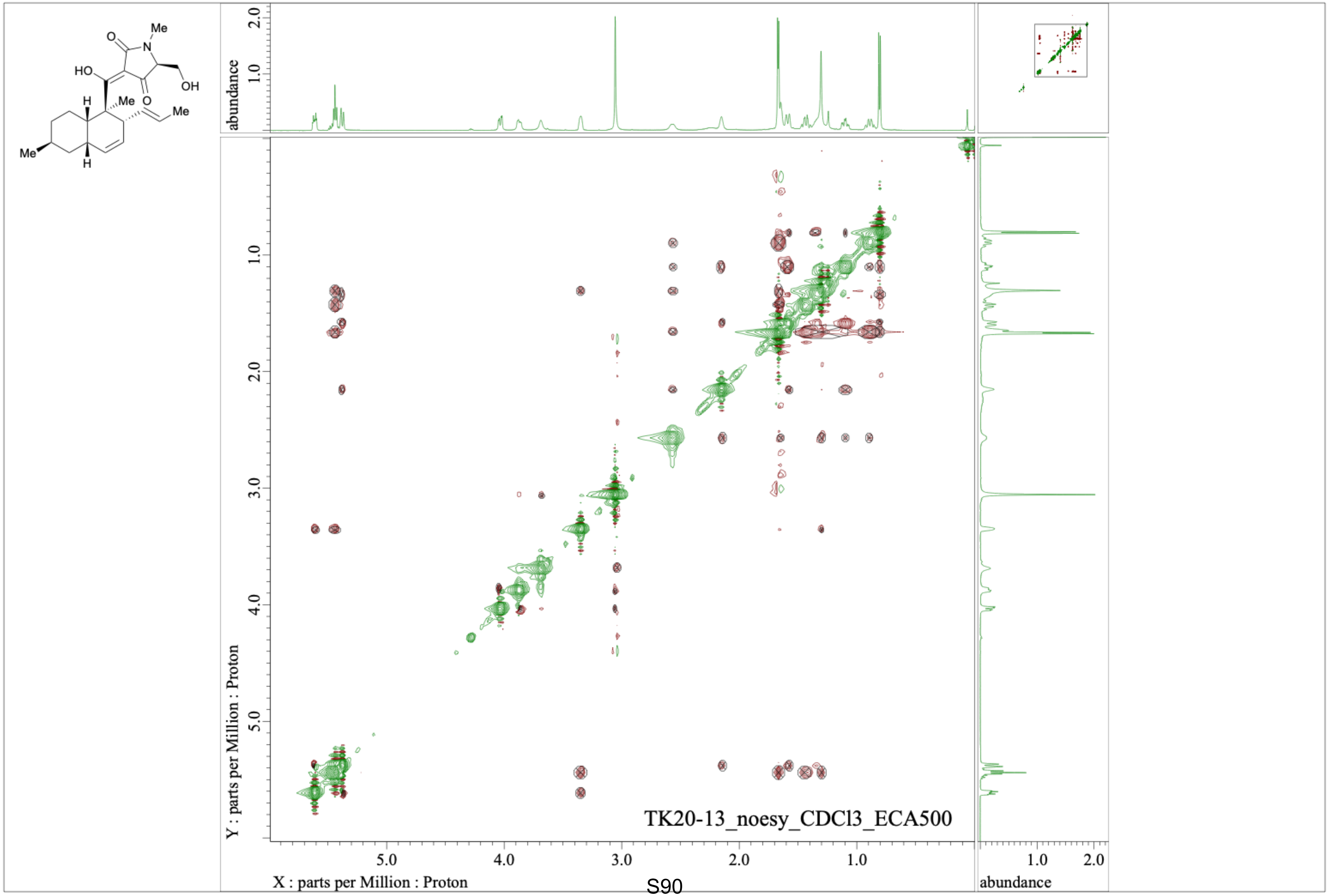

Figure S62. ^{1}H NMR spectrum of Compound 2Bb in CDCl$_3$ (600 MHz)

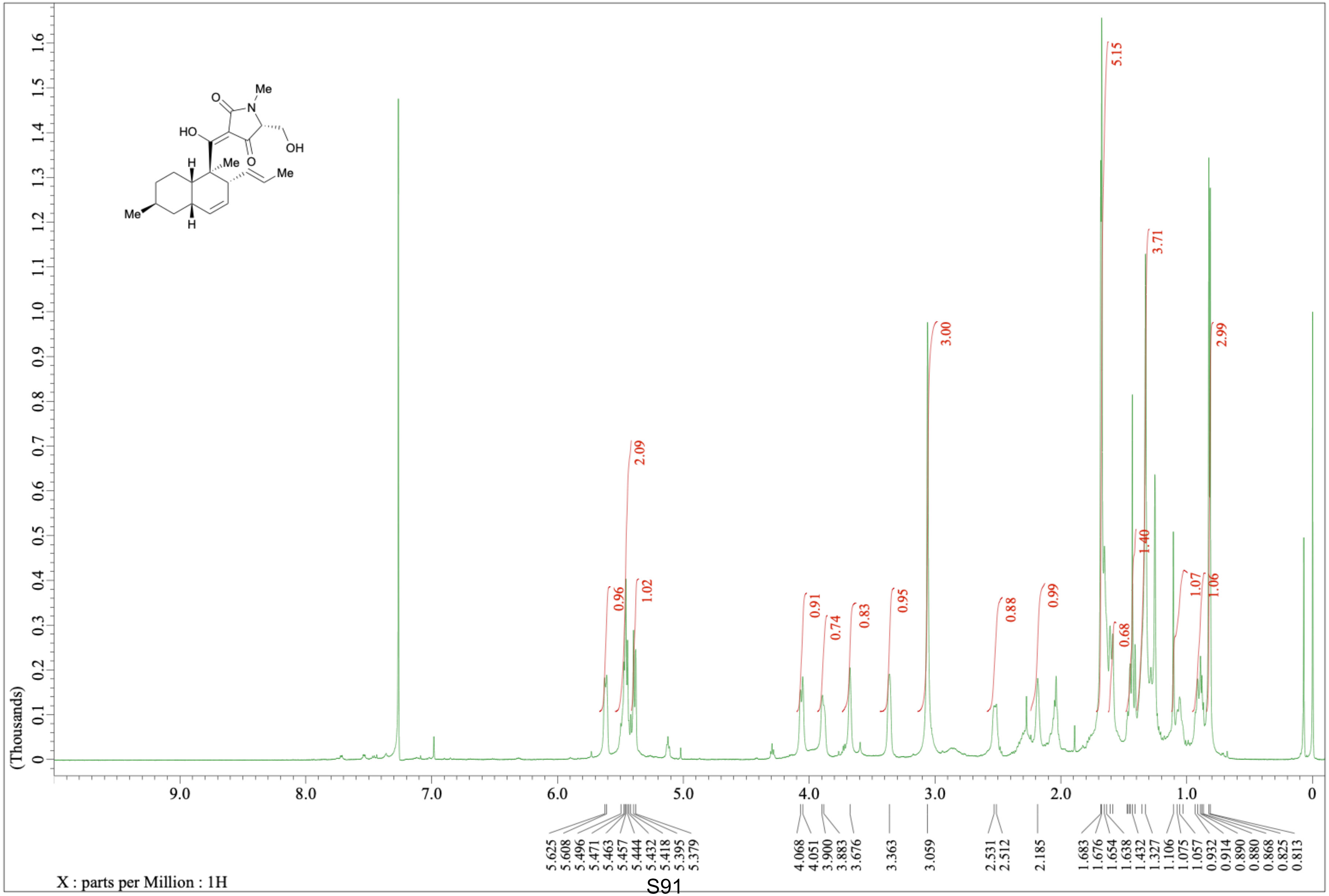

Figure S63. ***^{13}C NMR spectrum of Compound 2Bb in CDCl$_3$ (125 MHz)***

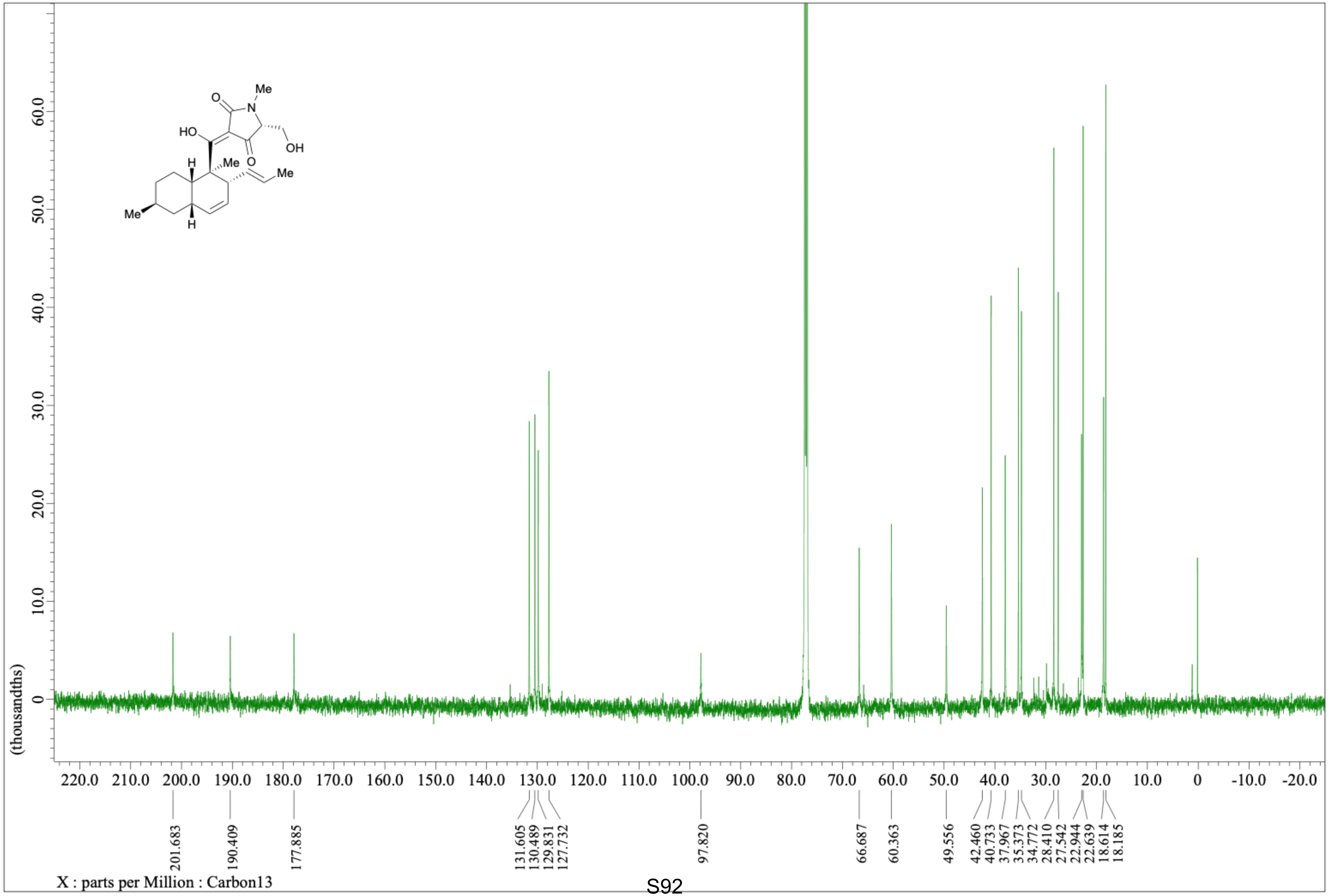

Figure S64. HH-COSY spectrum of Compound 2Bb in $CDCl_3$

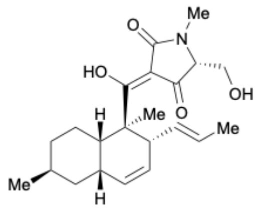

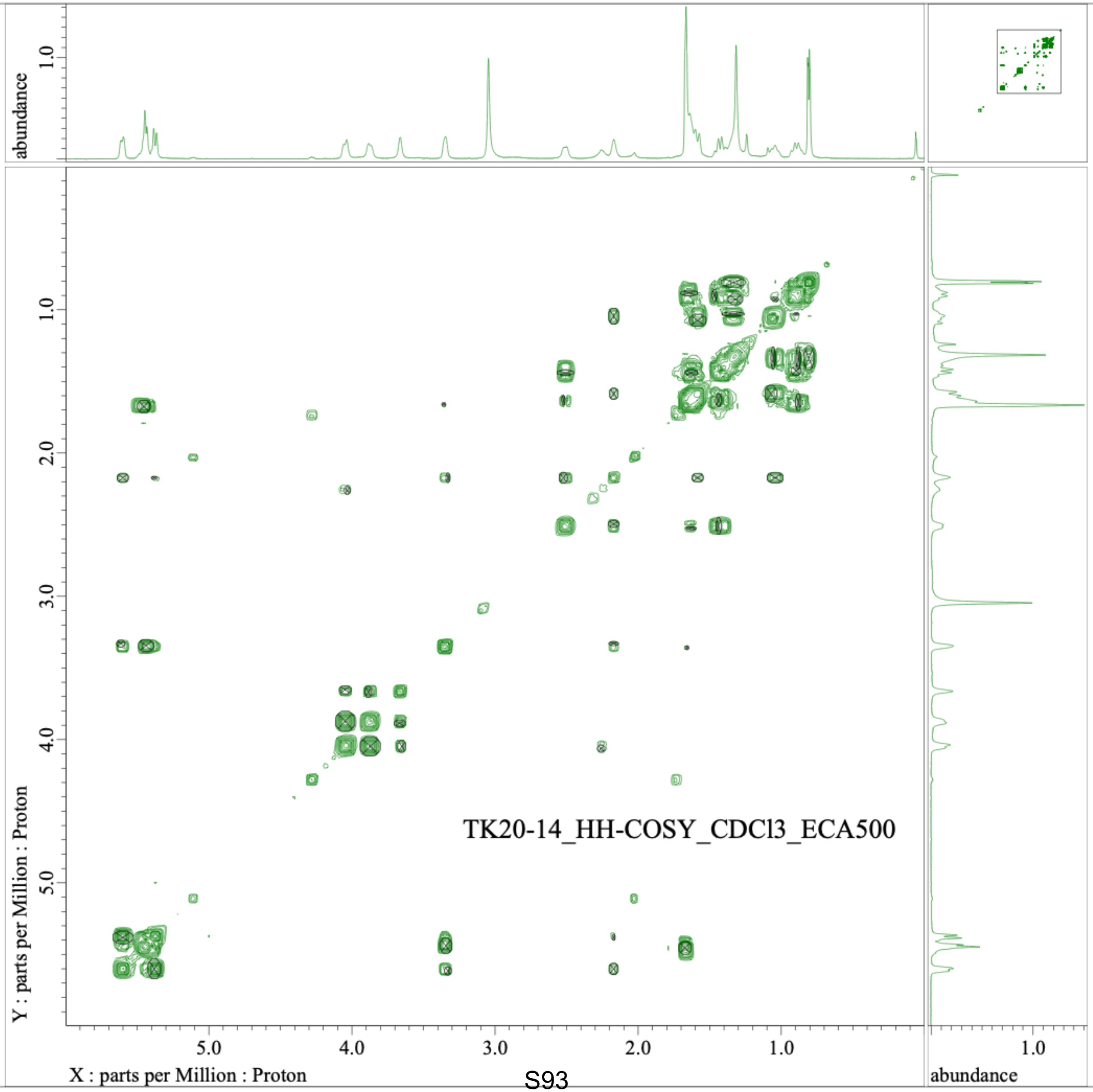

Figure S65. HSQC spectrum of Compound 2Bb in $CDCl_3$

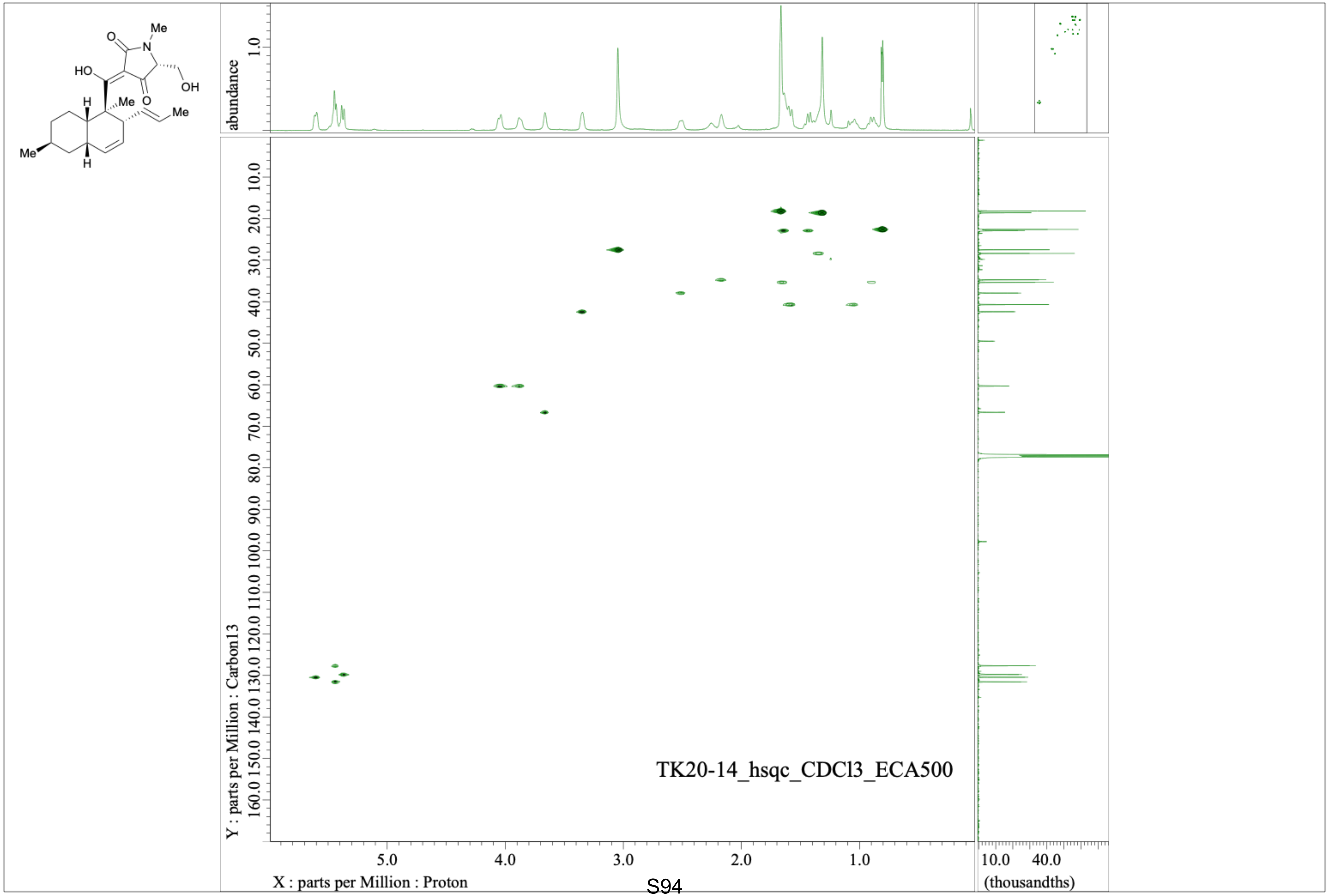

Figure S66. HMBC spectrum of Compound 2Bb in CDCl₃

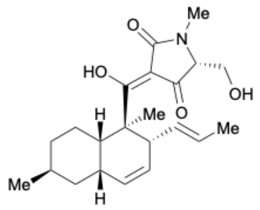

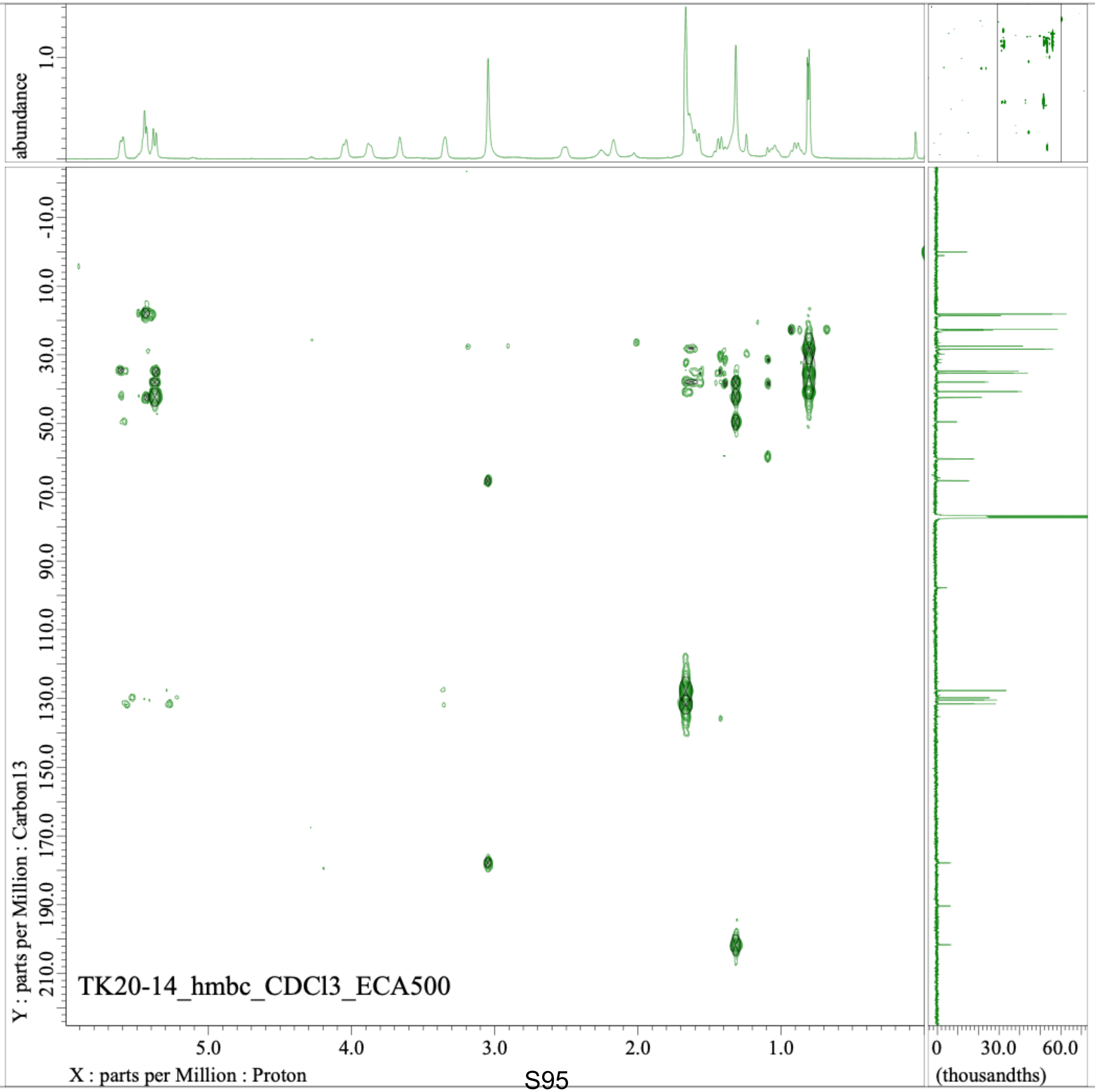

Figure S67. NOESY spectrum of Compound 2Bb in $CDCl_3$

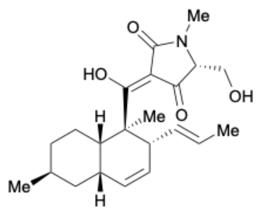

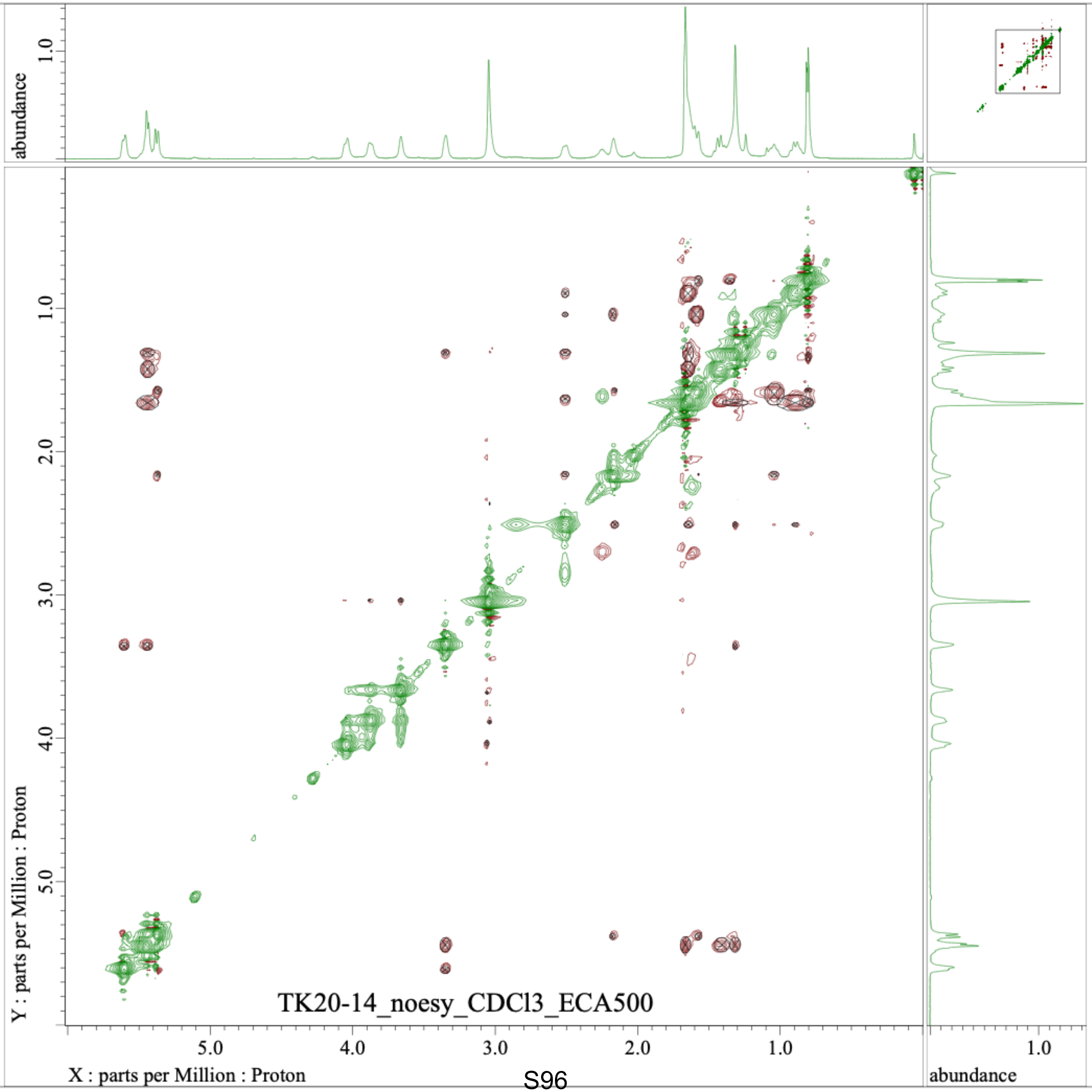

Figure S68. ^{1}H NMR spectrum of Compound 2Ca in CDCl$_3$ (600 MHz)

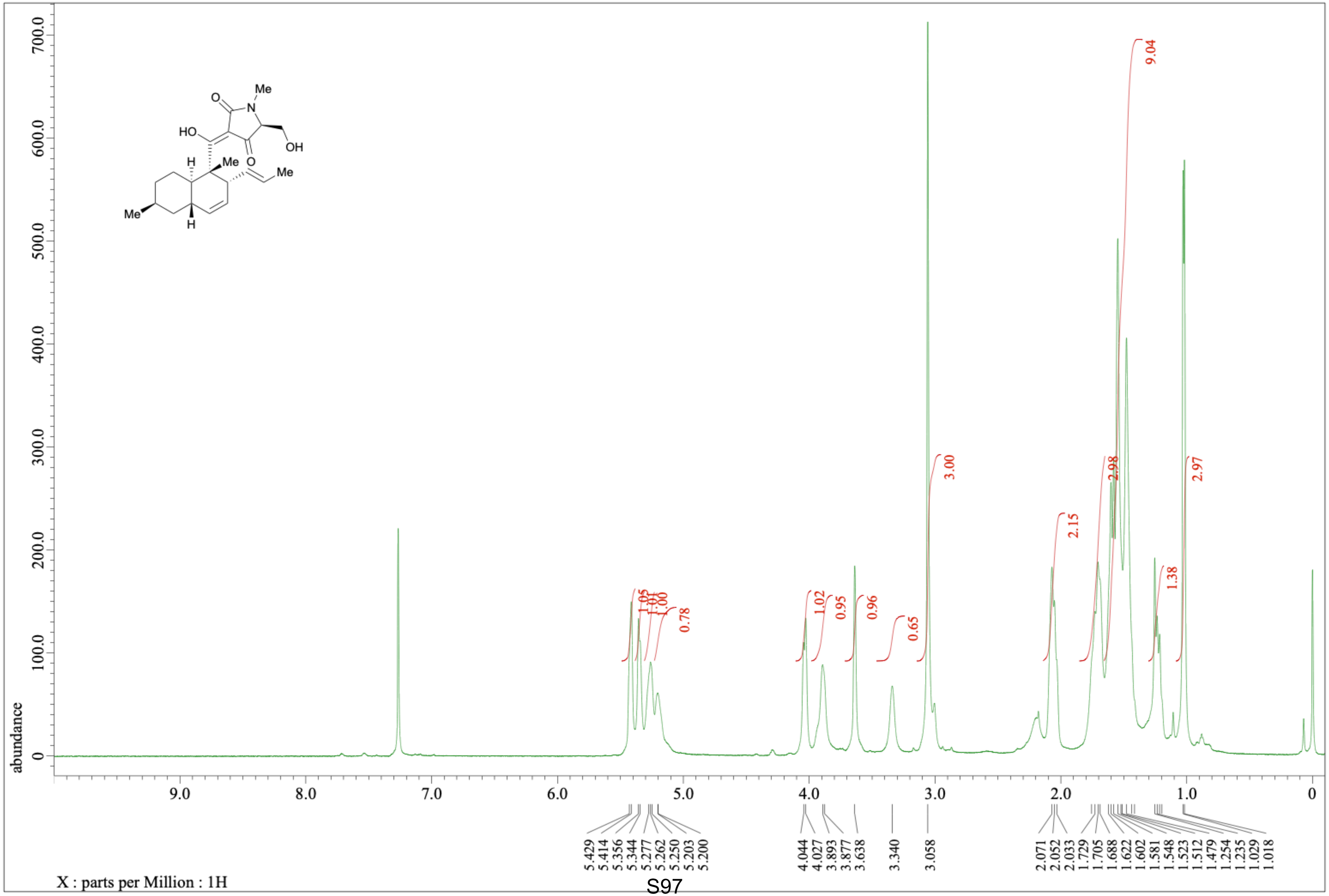

Figure S69. ^{13}C NMR spectrum of Compound 2Ca in CDCl$_3$ (150 MHz)

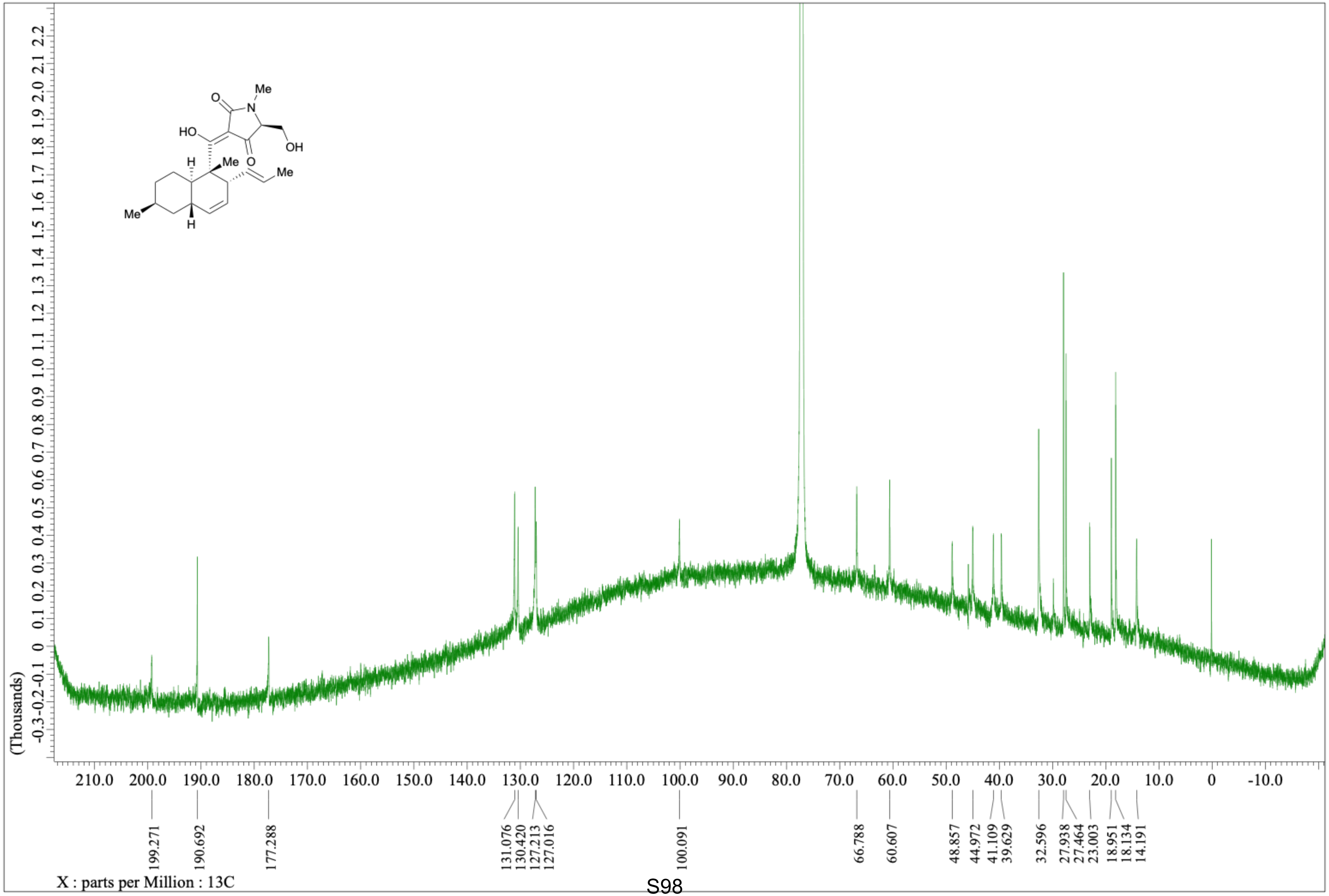

Figure S70. HH-COSY spectrum of Compound 2Ca in $CDCl_3$

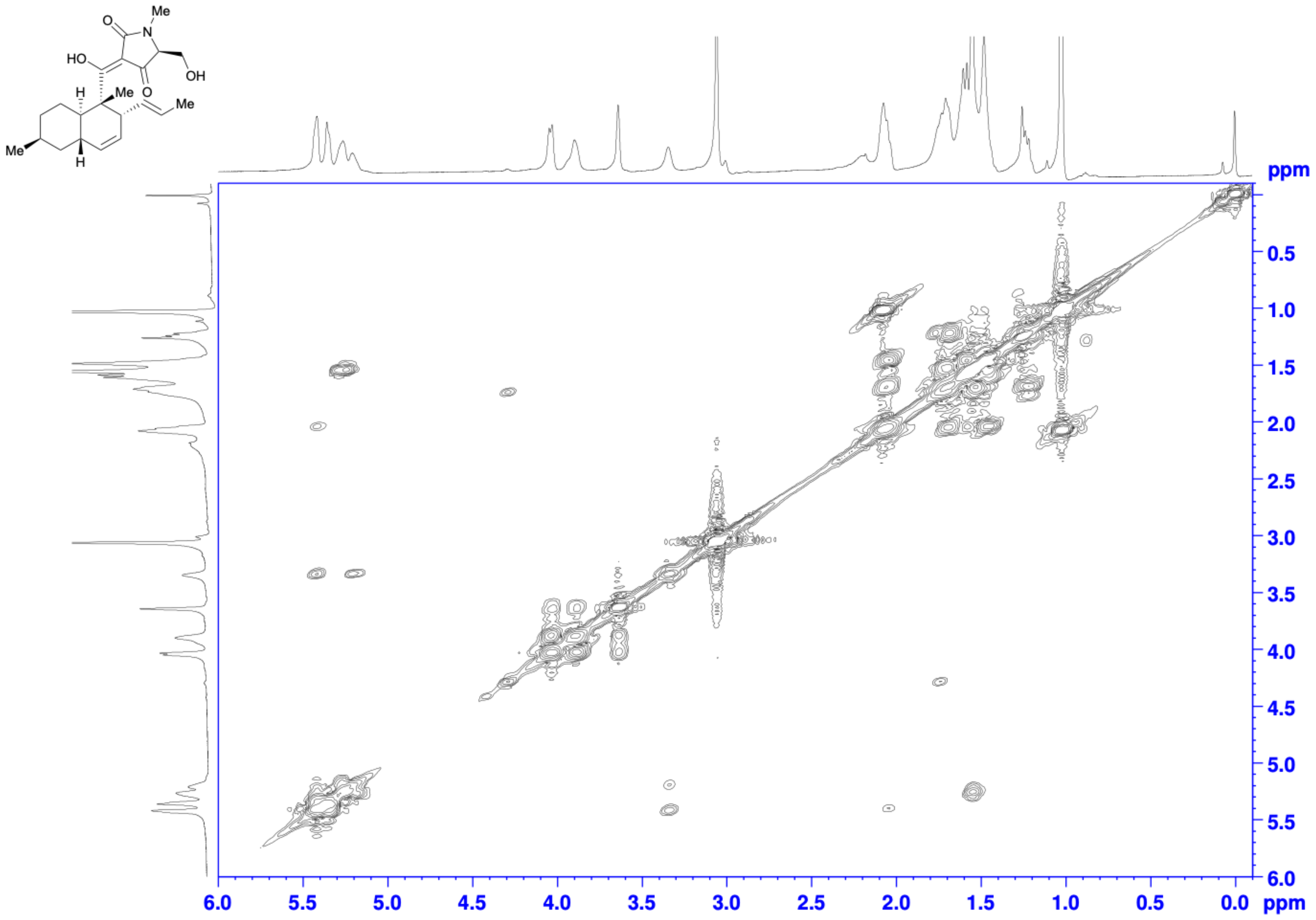

Figure S71. HMQC spectrum of Compound 2Ca in $CDCl_3$

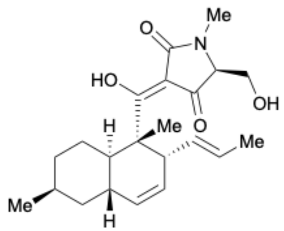

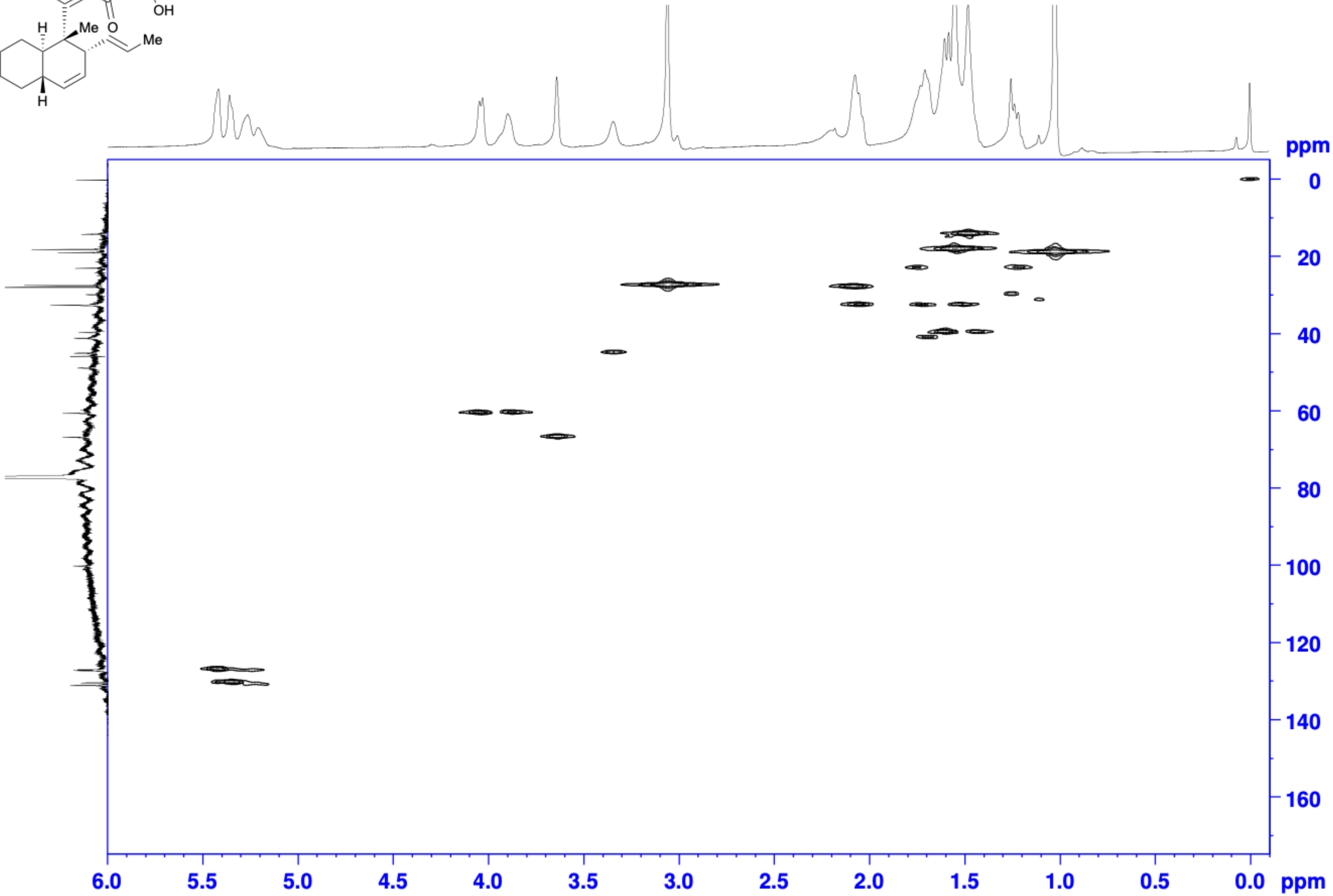

Figure S72. HMBC spectrum of Compound 2Ca in $CDCl_3$

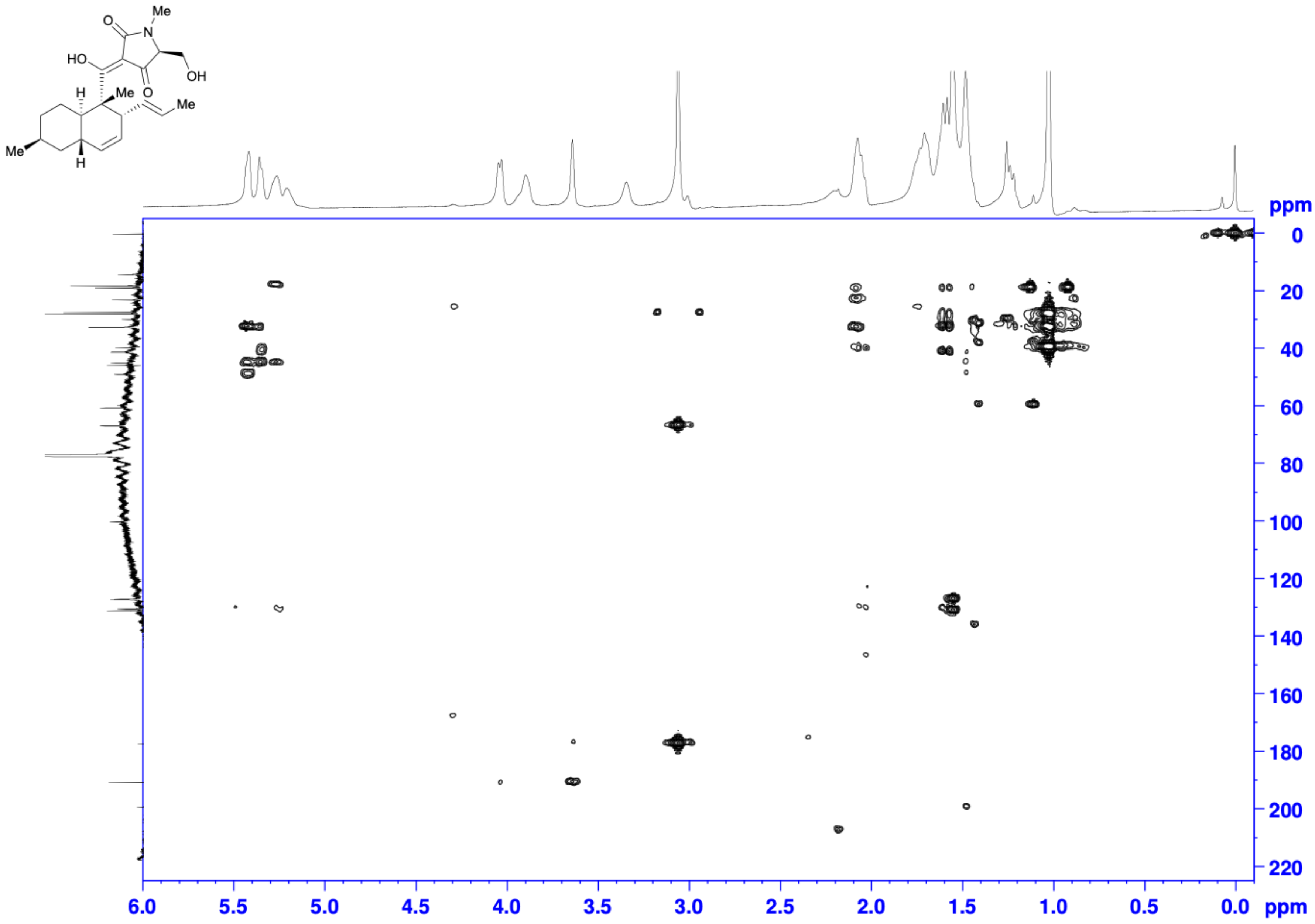

Figure S73. NOESY spectrum of Compound 2Ca in $CDCl_3$

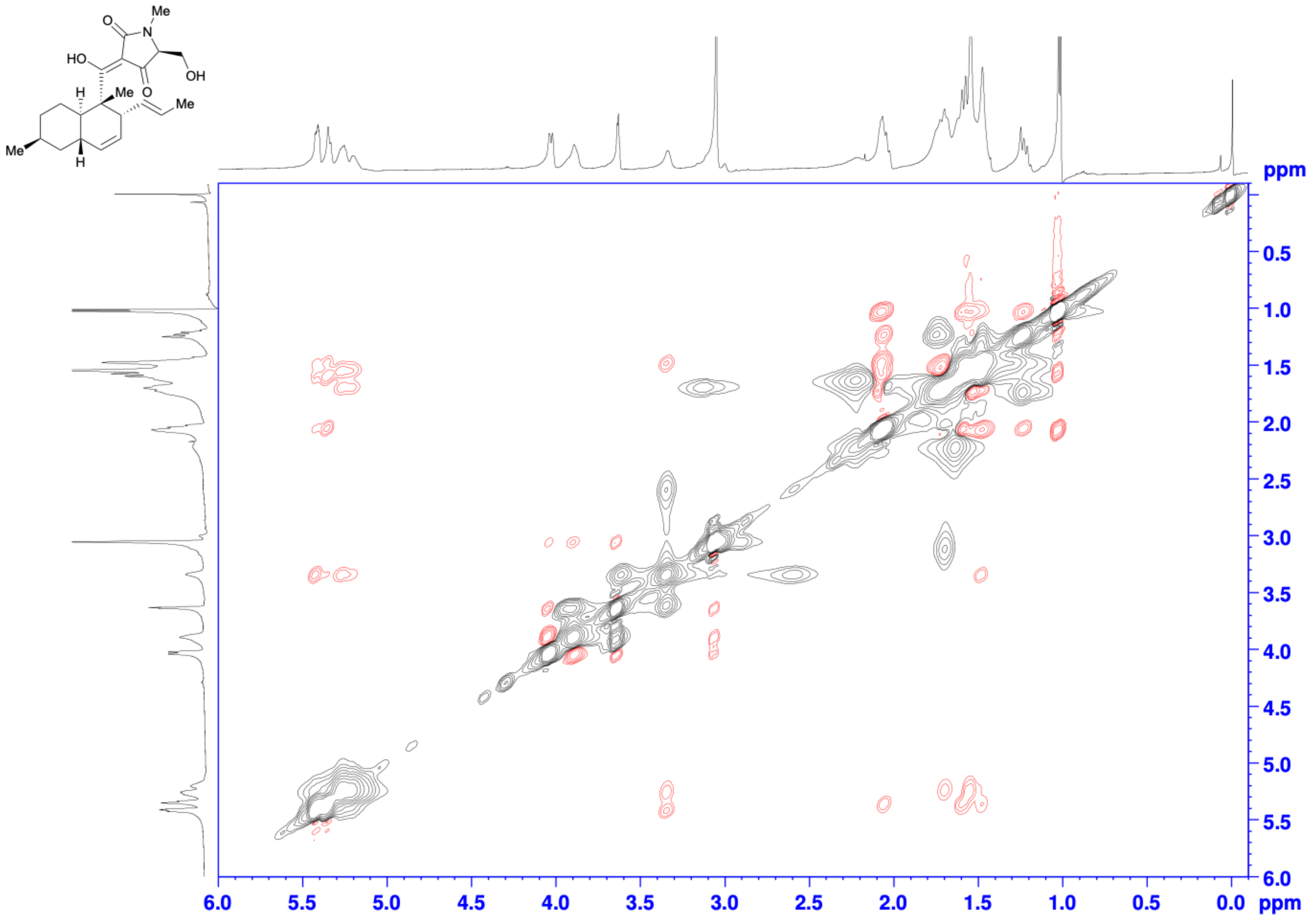

Figure S74. ^{1}H NMR spectrum of Compound 17a in CDCl₃ (600 MHz)

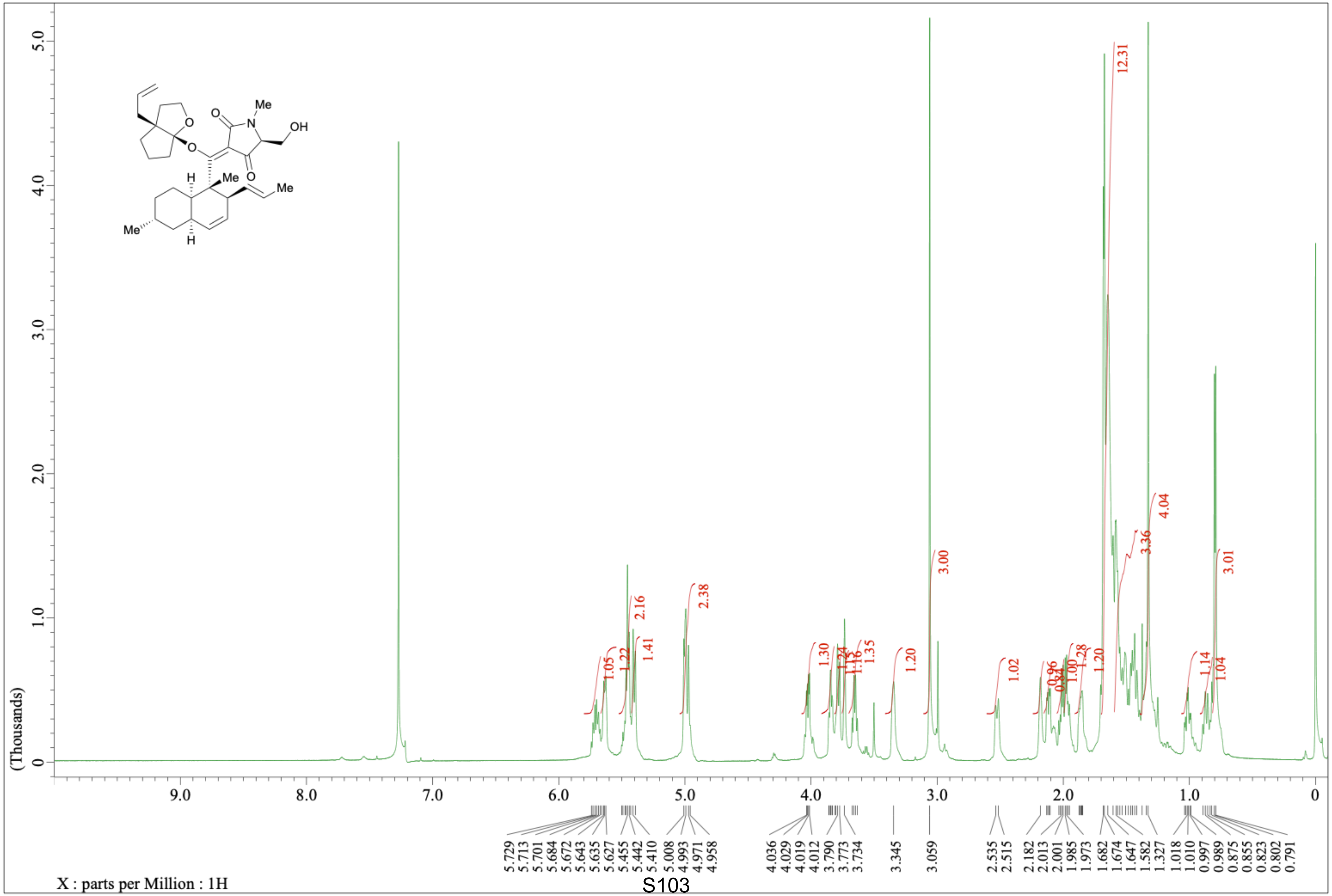

Figure S75. ***^{13}C NMR spectrum of Compound 17a in $CDCl_3$ (100 MHz)***

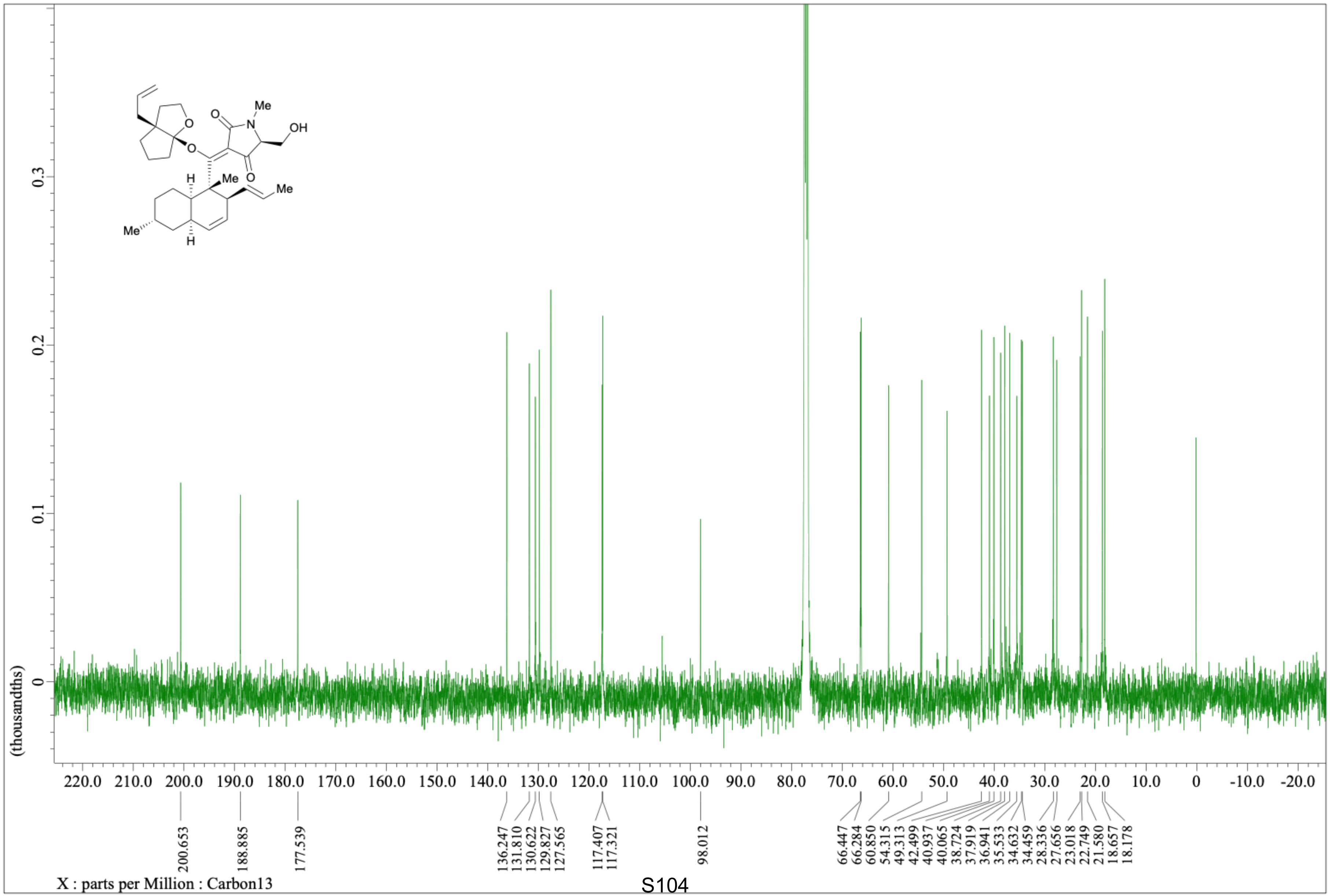

Figure S76. HSQC spectrum of Compound 17a in CDCl₃

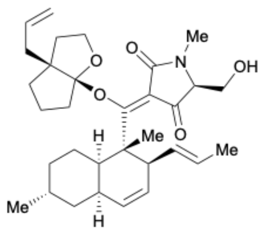

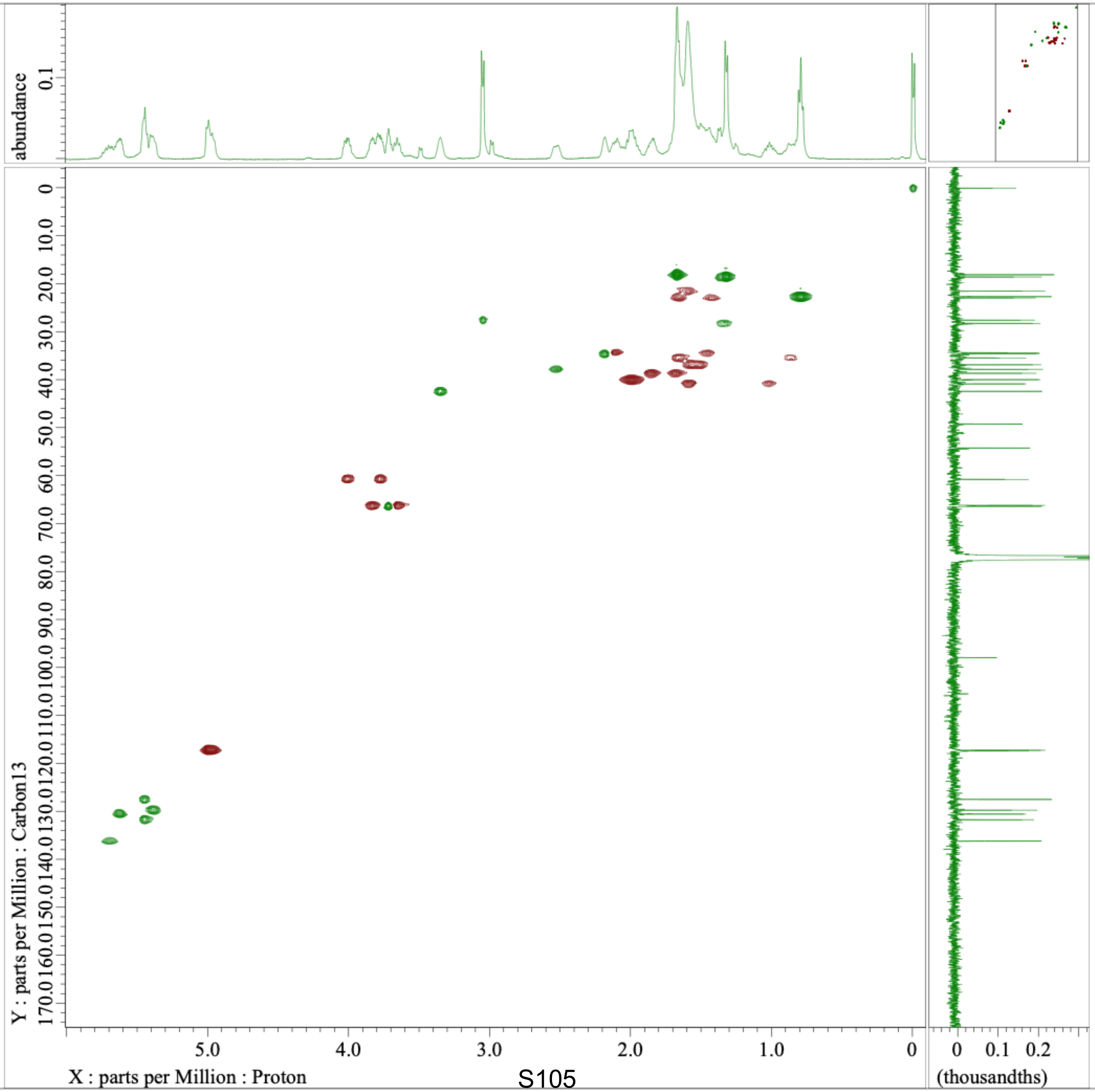

Figure S77. ^{1}H NMR spectrum of Compound 17b in CDCl₃ (600 MHz)

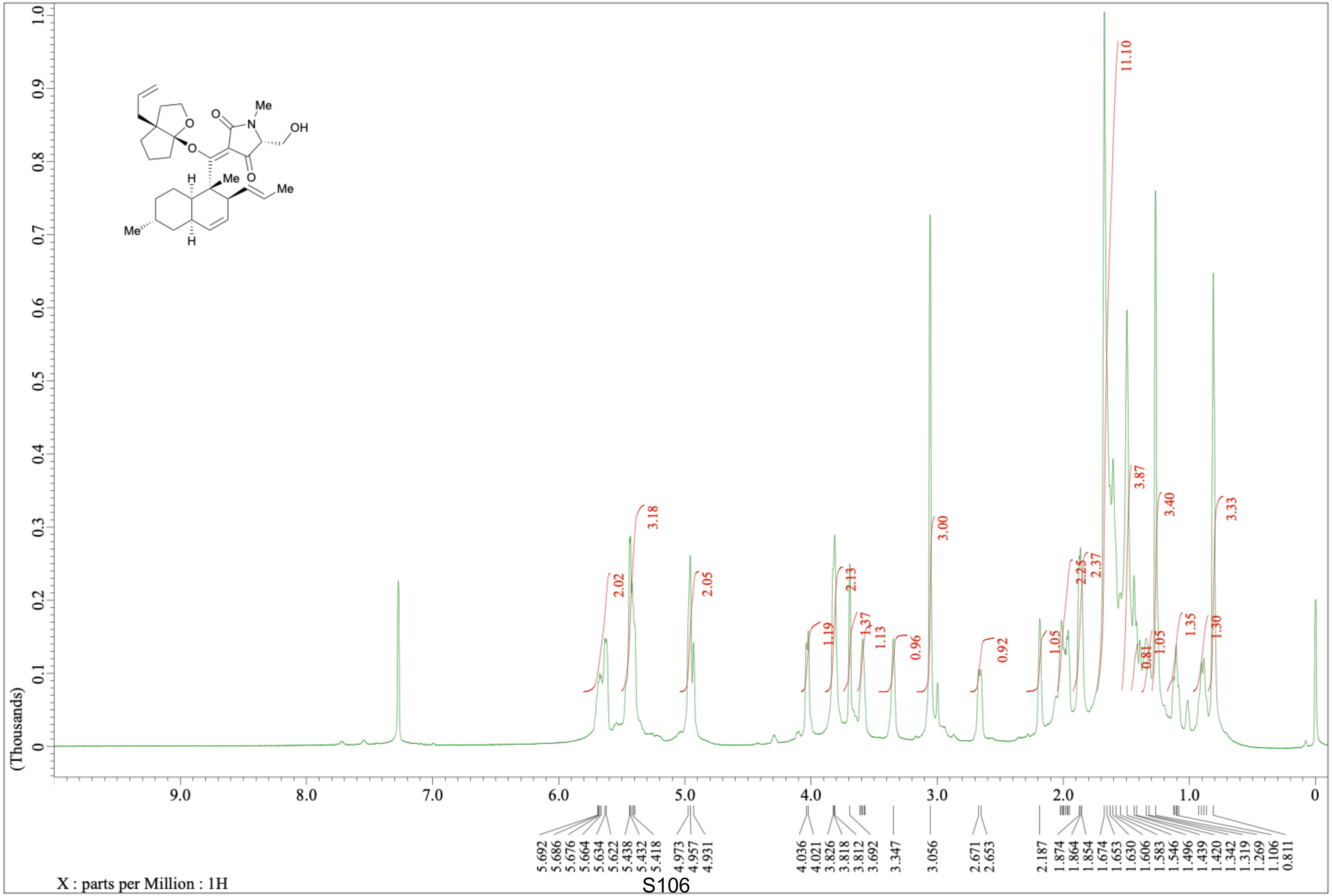

Figure S78. ^{13}C NMR spectrum of Compound 17b in $CDCl_3$ (100 MHz)

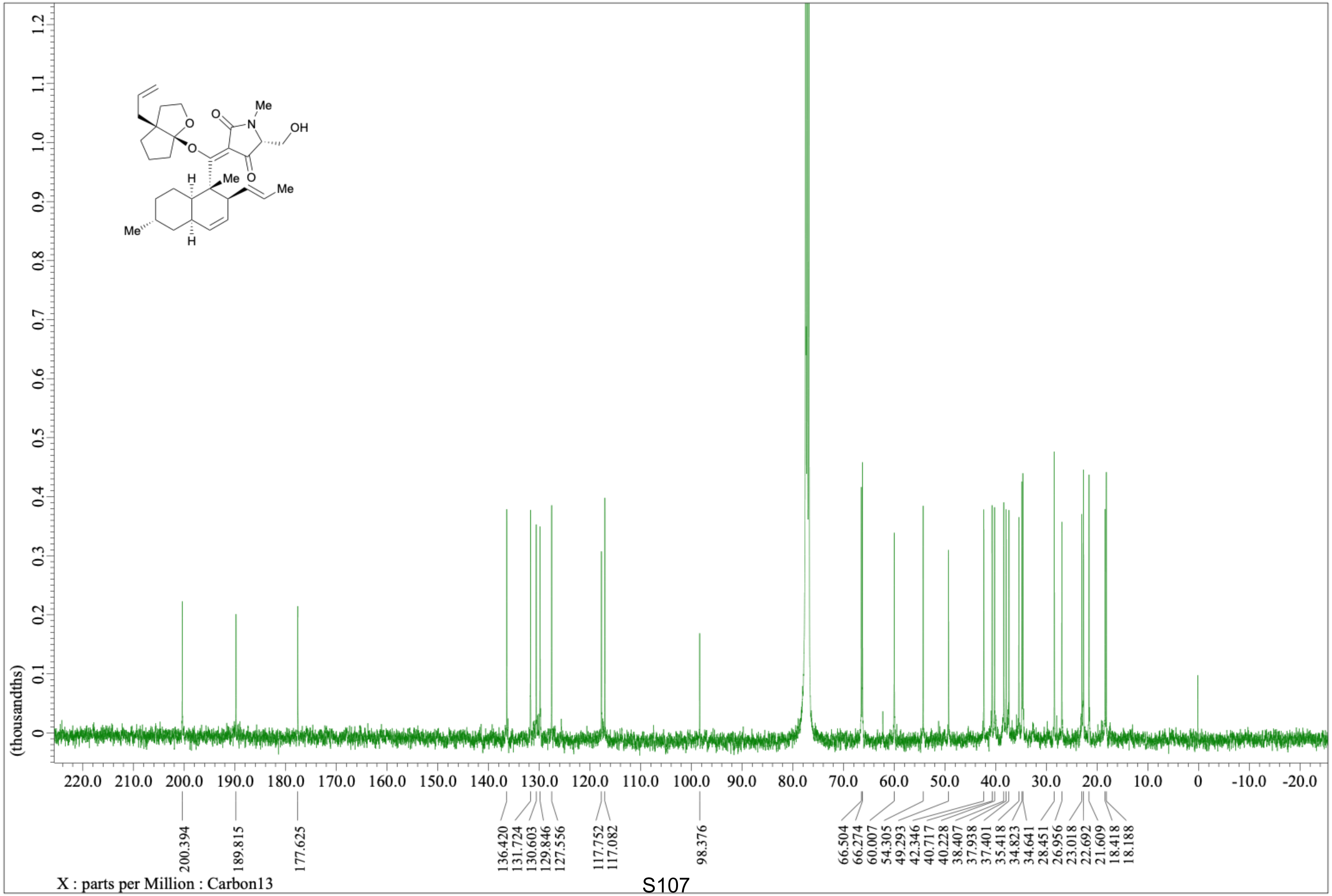

Figure S79. HSQC spectrum of Compound 17b in CDCl$_3$

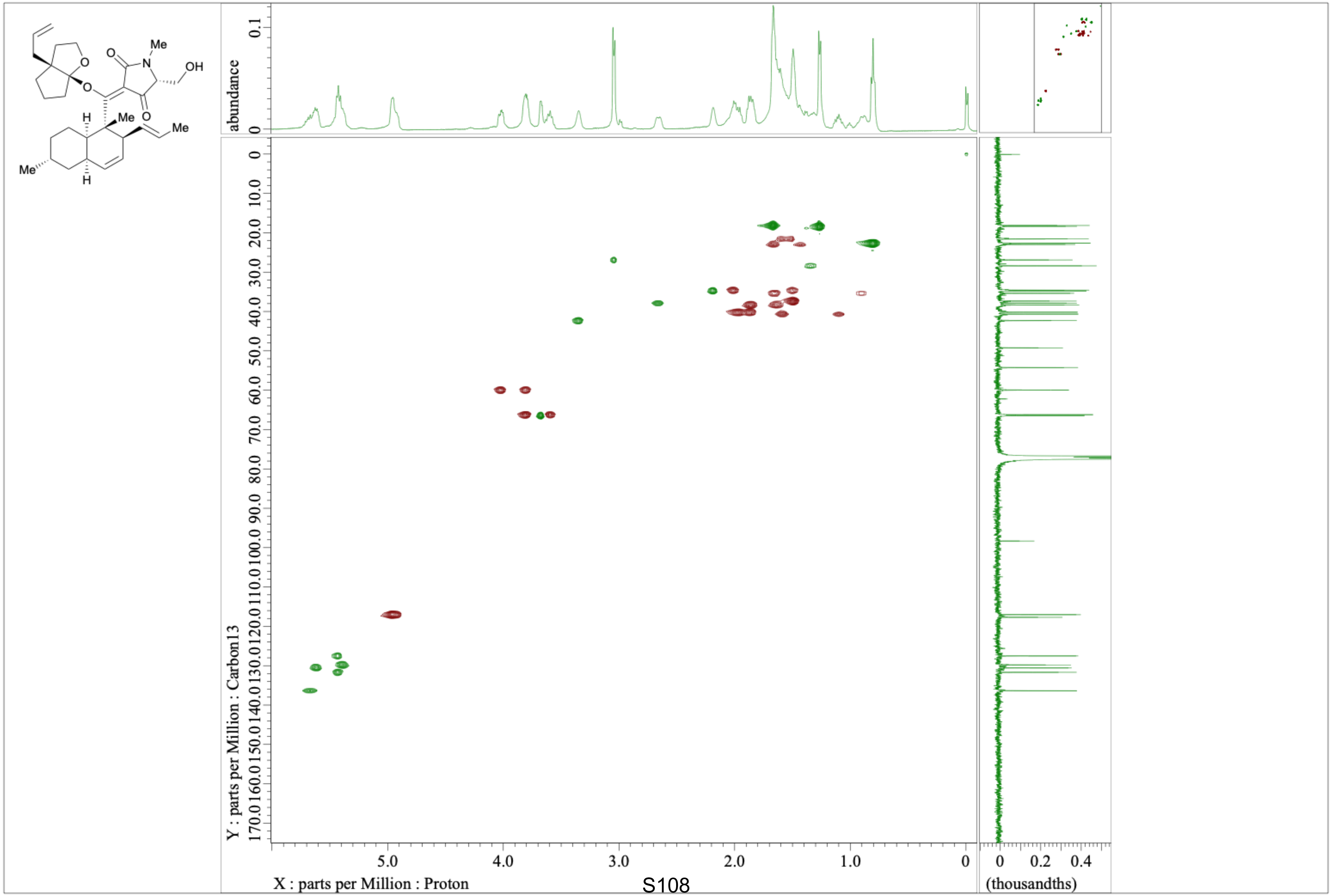

Figure S80. ¹H NMR spectrum of Compound 18a in CDCl₃ (600 MHz)

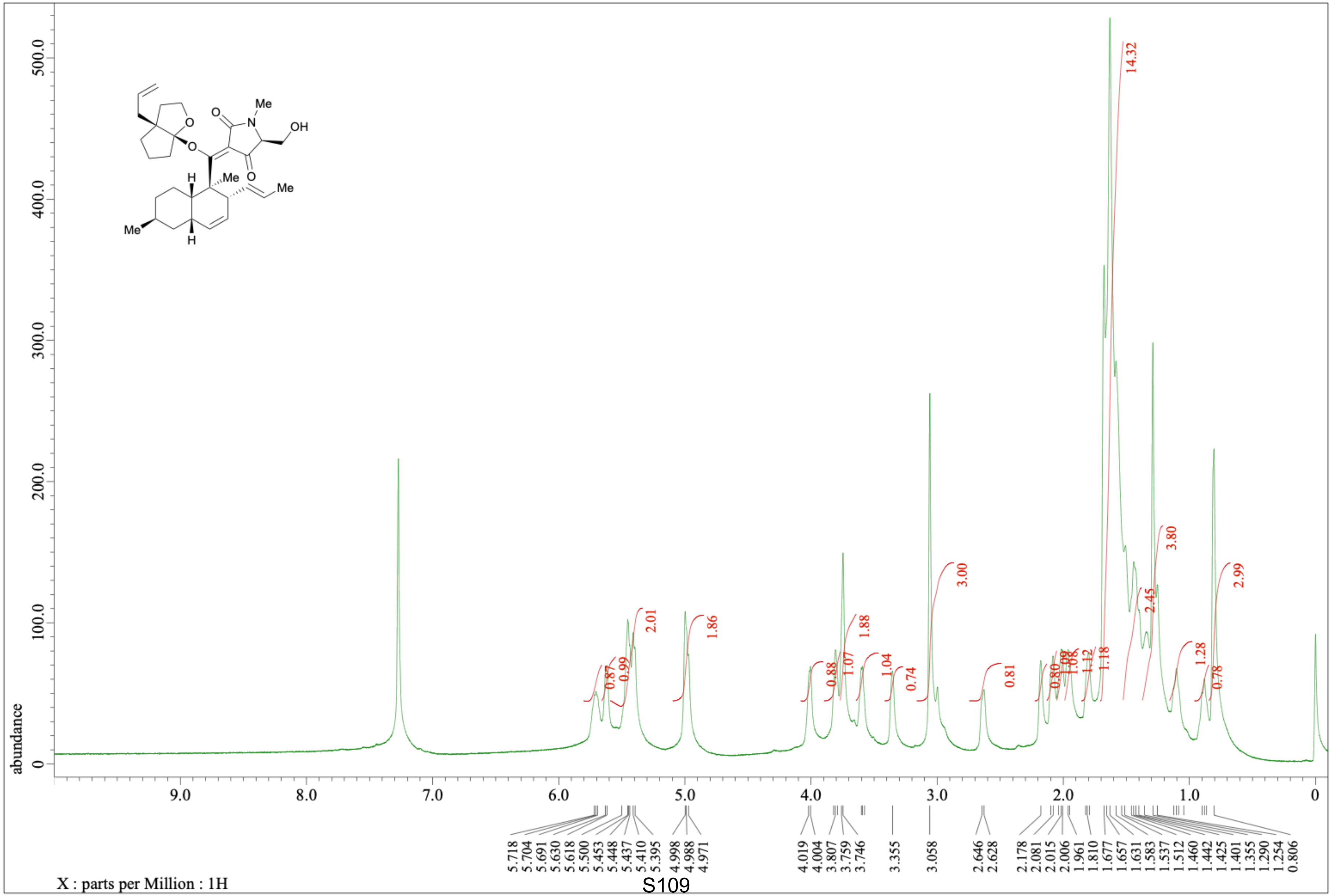

Figure S81. ***^{13}C NMR spectrum of Compound 18a in $CDCl_3$ (100 MHz)***

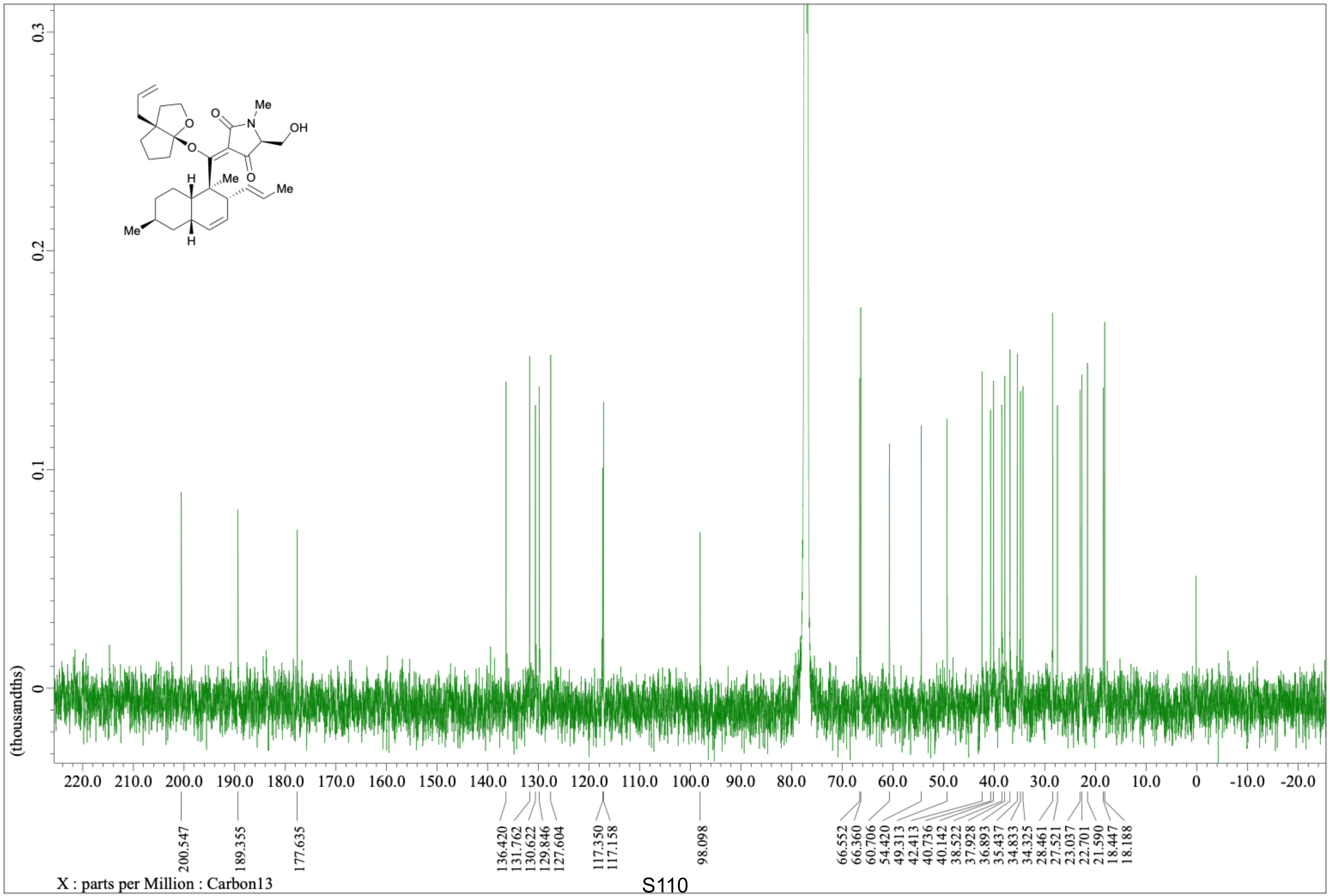

Figure S82. HSQC spectrum of Compound 18a in $CDCl_3$

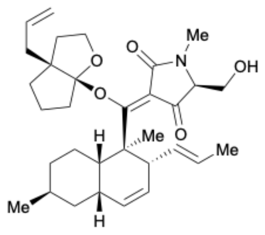

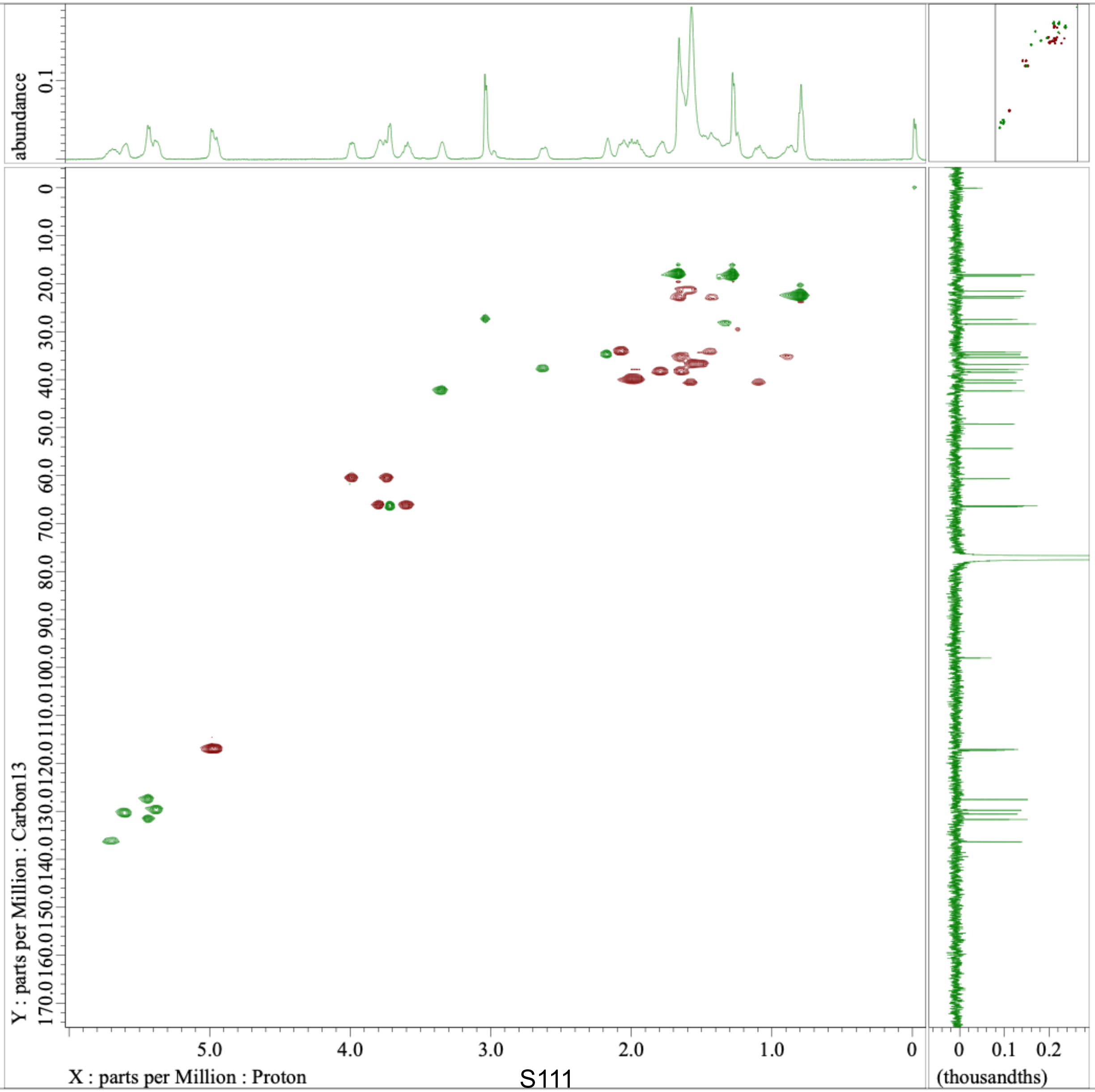

Figure S83. ***^{1}H NMR spectrum of Compound 18b in CDCl₃ (400 MHz)***

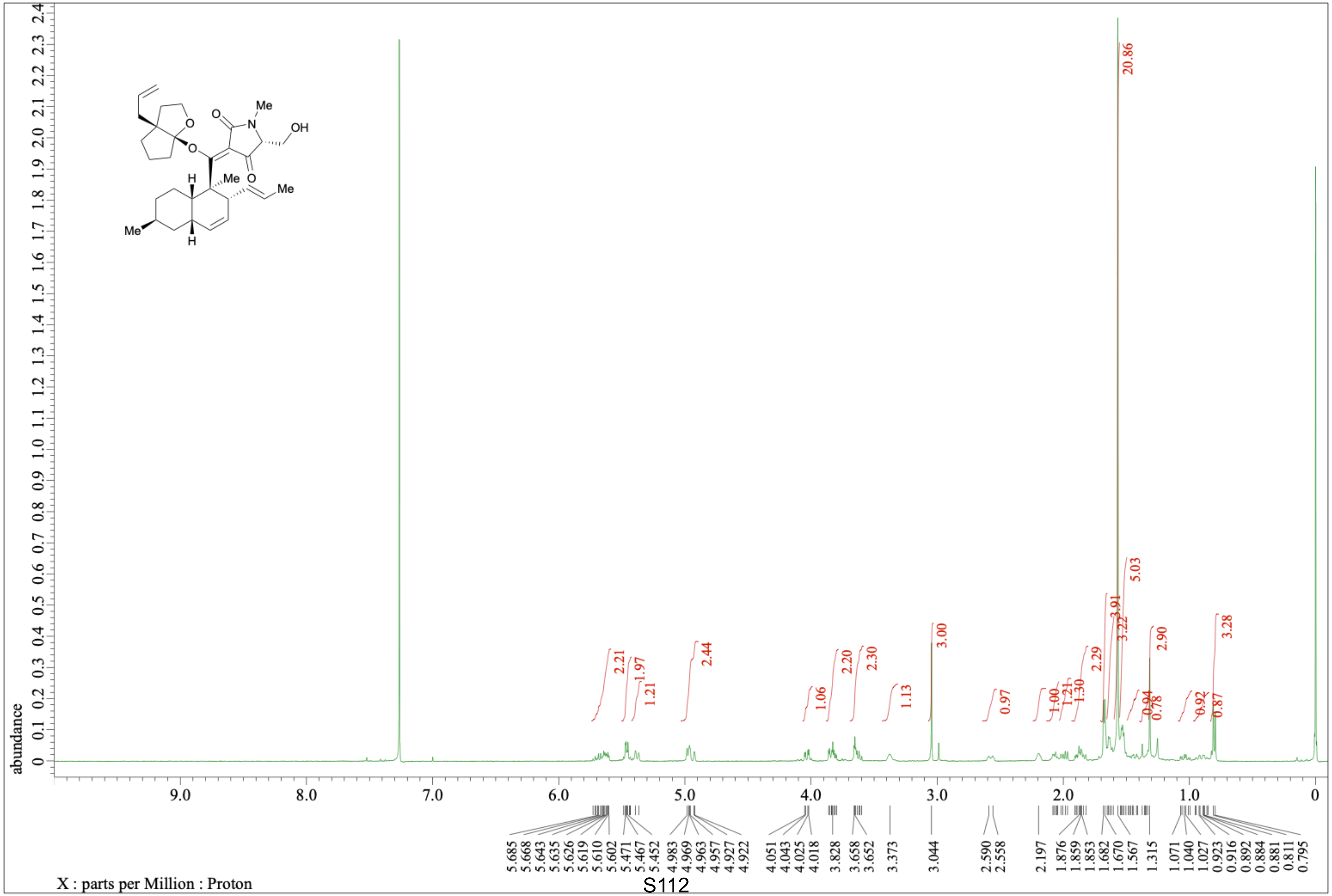

Figure S84. ^{13}C NMR spectrum of Compound 18b in CDCl$_3$ (100 MHz)

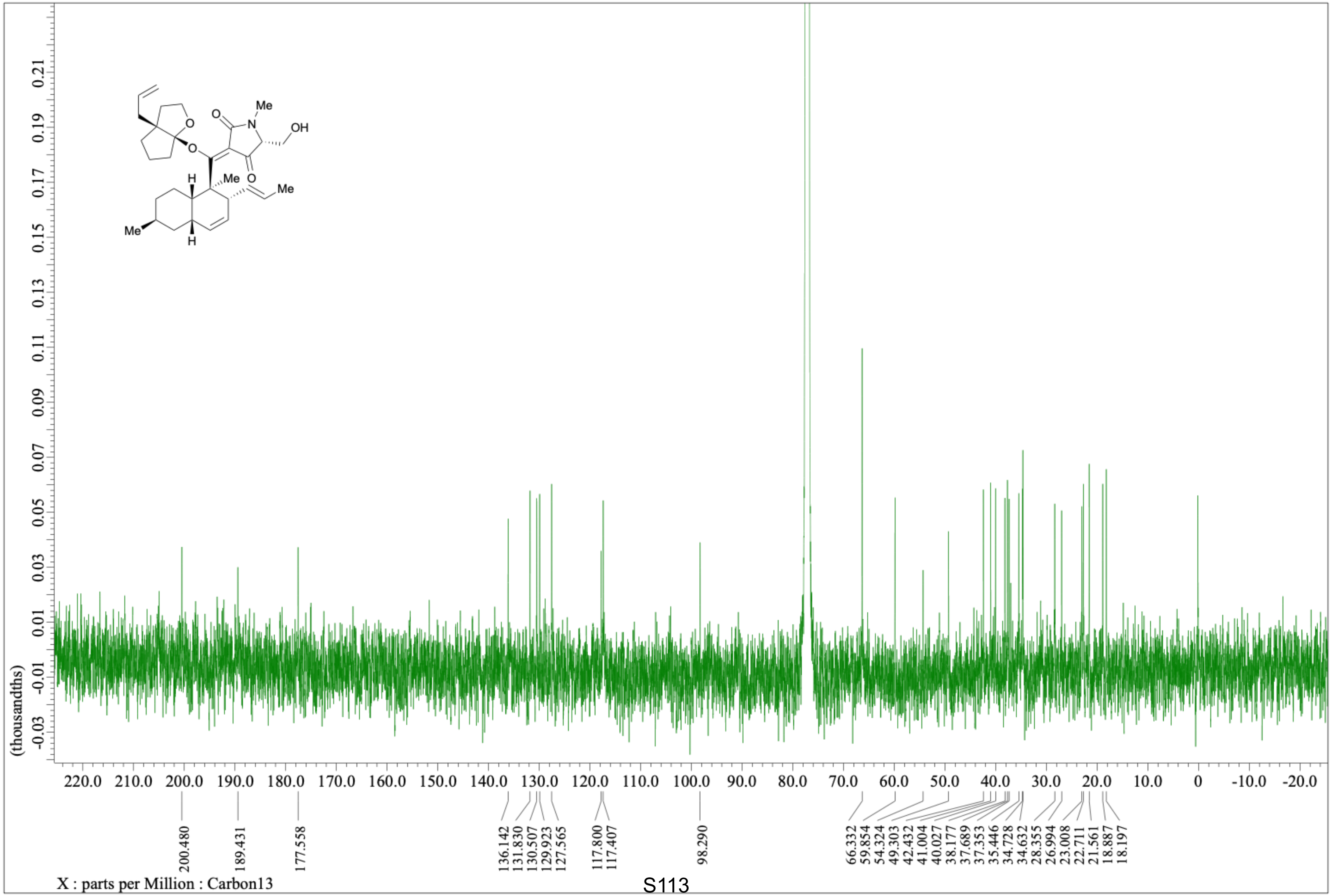

Figure S85. HSQC spectrum of Compound 18b in $CDCl_3$

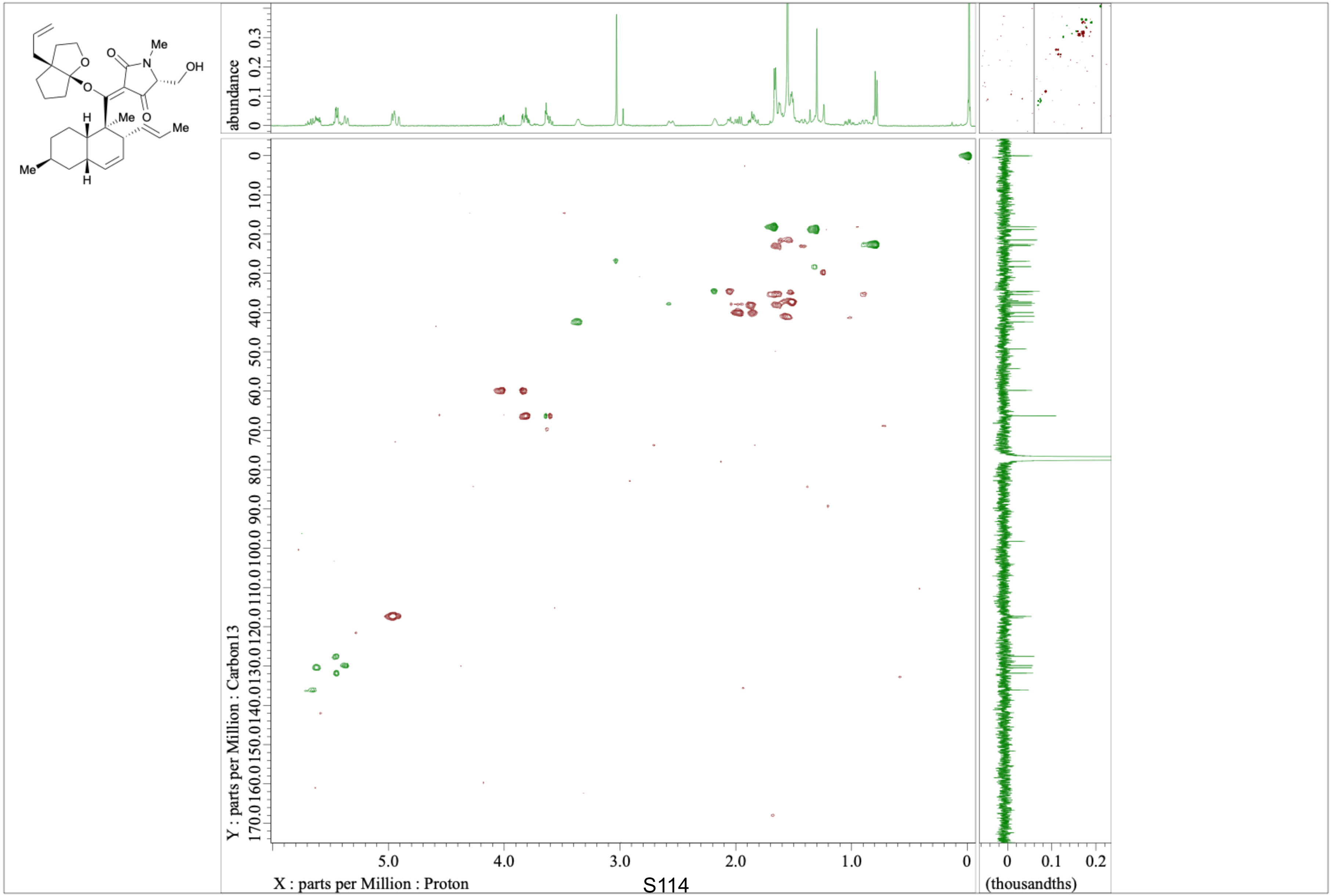

Cartesian Coordinates and Energies

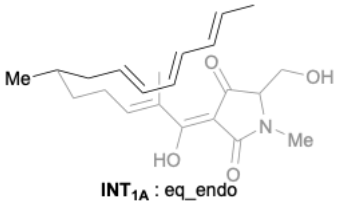

INT_{1A} : eq_endo

Electronic Energy (EE) [A.U.] : −1212.452895

EE + Zero-point Energy [A.U.] : −1211.955279

Gibbs Free energy [A.U.] : −1212.015254

C	-5.34364300	-0.23637000	-0.43465700
C	-4.77007100	-1.54472300	0.13547100
C	-4.74806800	1.04644800	0.16570200
H	-4.74090300	-1.48124200	1.23040300
H	-5.48353300	-2.33809600	-0.10332800
H	-5.14730000	-0.22469900	-1.51489300
H	-4.92632400	1.06402900	1.24743500
H	-5.31510300	1.88842200	-0.25343900
C	-6.86043500	-0.21852800	-0.22514900
H	-7.30443300	0.69464500	-0.62821000
H	-7.33561300	-1.07336300	-0.71109800
H	-7.09657700	-0.26445900	0.84300500
C	-3.39645700	-1.99183800	-0.39994300
H	-3.30759800	-1.74969700	-1.46177200
H	-3.35736100	-3.08781300	-0.34215600
C	-2.21795000	-1.50359400	0.37779200
H	-2.35250400	-1.47370700	1.45533100
C	-3.29384300	1.28632900	-0.11270500
H	-2.93788900	0.98424000	-1.09785600
C	-2.44831000	1.88020600	0.73934100
H	-2.81859300	2.14699500	1.72691100
C	-1.03594600	2.16734700	0.48897500
H	-0.44052400	2.42054600	1.36426800
C	-0.40799300	2.11471600	-0.69891200
H	-0.97016600	1.89146500	-1.60383200
C	1.02136600	2.32852200	-0.86103100
H	1.59083600	2.56162600	0.03944800
C	1.65567800	2.24611300	-2.03823900
H	1.07014600	2.00903900	-2.92556100
C	3.11908200	2.48422200	-2.24174400
H	3.62877900	2.66046700	-1.29201000
H	3.59134500	1.63517000	-2.74486800
H	3.28213200	3.35437300	-2.88495200
C	-1.01061200	-1.16999100	-0.11666400
C	-0.71438100	-1.11050600	-1.59441600
H	-0.44271800	-2.08506900	-2.00220200
H	-1.59545600	-0.75023800	-2.12889500
H	0.10514300	-0.42473900	-1.80288200
C	0.00083200	-0.74115600	0.85197000
C	1.36794400	-0.62250200	0.62546600
C	2.20472300	0.08928800	1.59855800
C	2.24260600	-1.09530300	-0.41875800
C	3.63528200	-0.51989200	-0.13200700
H	3.90930300	0.18451500	-0.92625000
O	-0.46001000	-0.39217300	2.03886400
H	0.29944100	-0.00591800	2.56453400
O	1.80847800	0.56626500	2.67668100
N	3.46858100	0.16299000	1.13589000
C	4.54290400	0.86936600	1.80564400
H	4.10800400	1.49957800	2.57886800
H	5.24841200	0.17482700	2.26716100
H	5.07486300	1.49389100	1.08542000
O	2.05650100	-1.88885400	-1.33119600
C	4.67658400	-1.64304300	-0.07759800
H	5.64127100	-1.24044300	0.23536600
H	4.35922300	-2.39051800	0.66061800
O	4.85650600	-2.21738700	-1.35492000
H	3.97608400	-2.44822200	-1.68380300

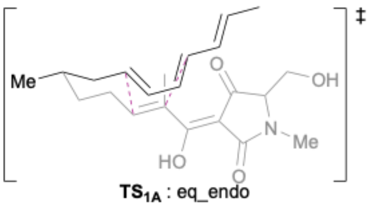

TS_{1A} : eq_endo

Electronic Energy (EE) [A.U.] : −1212.433277

EE + Zero-point Energy [A.U.] : −1211.935097

Gibbs Free energy [A.U.] : −1211.992916

C	-5.40202500	-0.43404600	-0.20015100
C	-4.71127700	-1.73922400	0.18423300
C	-4.63252300	0.76722100	0.34473200
H	-4.71505100	-1.84065900	1.27713900
H	-5.28018100	-2.58391300	-0.21514500
H	-5.40822900	-0.36442100	-1.29624600
H	-4.62290500	0.72354100	1.44122500

H	-5.17267000	1.68196100	0.07521900
C	-6.84547600	-0.40893200	0.29684900
H	-7.35057800	0.51310300	-0.00092900
H	-7.41172000	-1.25309700	-0.10344800
H	-6.87283900	-0.47167100	1.38925100
C	-3.27985400	-1.81334600	-0.33480000
H	-3.28079000	-1.68726700	-1.42113800
H	-2.87072500	-2.81251000	-0.14461200
C	-2.32524900	-0.82959800	0.31987100
H	-2.46783100	-0.72241900	1.39066500
C	-3.20481600	0.90937700	-0.15474900
H	-3.06813300	0.73873200	-1.22226600
C	-2.42280700	1.90245400	0.43988300
H	-2.77214200	2.30293100	1.38860700
C	-1.15772100	2.32435300	0.00193200
H	-0.60092500	2.99800000	0.64752600
C	-0.51830900	1.80760500	-1.09546500
H	-1.06884000	1.21349700	-1.81720400
C	0.86697700	2.06904300	-1.39171600
H	1.43383100	2.64699100	-0.66327700
C	1.48494000	1.58446700	-2.48189100
H	0.89874600	1.00963500	-3.19701000
C	2.92916700	1.78373500	-2.80007300
H	3.45007900	2.29668600	-1.98969700
H	3.41650200	0.82198500	-2.98549900
H	3.04284600	2.36950800	-3.71747500
C	-0.97695800	-0.82179500	-0.11754100
C	-0.62547800	-1.33053600	-1.48757300
H	-0.56581200	-2.42225700	-1.51828100
H	-1.39206200	-1.02602200	-2.20617400
H	0.33148200	-0.93988900	-1.82328100
C	-0.00216100	-0.32958800	0.77775800
C	1.41405200	-0.34625300	0.62784400
C	2.22664100	0.55234500	1.44129000
C	2.30246700	-1.12397500	-0.16452100
C	3.71997600	-0.58507300	0.08163200
H	4.10842900	-0.13944200	-0.84305800
O	-0.48058400	0.23557300	1.89304200
H	0.26454400	0.74051700	2.31755200
O	1.80208400	1.33515600	2.31444000
N	3.53188300	0.41845800	1.10499700
C	4.60081300	1.25446400	1.60814600
H	5.29730900	0.68319700	2.22631400
H	5.14865000	1.70029700	0.77430300
H	4.15971700	2.04476100	2.21261300
O	2.12797400	-2.12789400	-0.86604800
C	4.65176400	-1.71912900	0.51931400
H	5.63399700	-1.32044300	0.77955900
H	4.22835300	-2.20044400	1.41066100
O	4.83577900	-2.64597900	-0.53157500
H	3.94888500	-2.84634500	-0.86978000

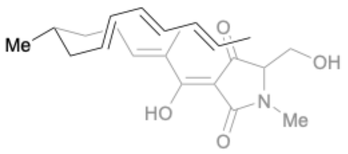

INT$_{1B}$: eq_exo

Electronic Energy (EE) [A.U.] : −1212.453295

EE + Zero-point Energy [A.U.] : −1211.955203

Gibbs Free energy [A.U.] : −1212.014489

C	-5.30822400	-0.30582400	0.00722200
C	-4.69808400	-1.69242700	0.26491800
C	-4.62317200	0.84112300	0.77788200
H	-4.64733400	-1.86571800	1.34749600
H	-5.40037500	-2.43272100	-0.12915700
H	-5.23006800	-0.09321100	-1.06592600
H	-4.57848500	0.56532500	1.83658700
H	-5.27745500	1.71840500	0.71783100
C	-6.79133300	-0.33687100	0.38624800
H	-7.27205400	0.62147100	0.17714200
H	-7.32358100	-1.11462500	-0.16593100
H	-6.90614700	-0.54448300	1.45521800
C	-3.33035900	-1.98358300	-0.37011600
H	-3.32159700	-1.64361400	-1.40913000
H	-3.20273300	-3.07383300	-0.40950400
C	-2.15360400	-1.44046500	0.37511200
H	-2.25927100	-1.37392500	1.45543500
C	-3.24798000	1.23610600	0.30093900
H	-2.44053800	1.17565600	1.02532000
C	-2.96992800	1.65929100	-0.94035700
H	-3.77401800	1.70323900	-1.67246700
C	-1.64066200	2.02322300	-1.42978300
H	-1.53777800	2.09807200	-2.51073300
C	-0.54045700	2.23946700	-0.68461600
H	-0.60117400	2.21022700	0.40203400

C	0.76668700	2.51871500	-1.25657500
H	0.83232400	2.54243400	-2.34398900
C	1.87097500	2.73065100	-0.52791000
H	1.78905100	2.70611900	0.55852000
C	3.22584400	3.01597300	-1.09582200
H	3.21031700	3.00290600	-2.18690000
H	3.58947400	3.99334500	-0.76491000
H	3.95781300	2.27962000	-0.75030900
C	-0.97453000	-1.09029700	-0.17378800
C	-0.73350700	-1.11627200	-1.66113900
H	-0.56577800	-2.12826600	-2.03338900
H	-1.60880100	-0.70093300	-2.16615100
H	0.12489600	-0.50540800	-1.93365900
C	0.05350200	-0.57445800	0.73075500
C	1.42090500	-0.55709200	0.50189800
C	2.31095600	0.16882700	1.41453700
C	2.25553500	-1.19983700	-0.48407200
C	3.68992700	-0.70963100	-0.24401500
H	4.01738100	-0.10714000	-1.10119500
O	-0.39701900	-0.04158800	1.85521100
H	0.38399900	0.33029900	2.35752500
O	1.95612600	0.76101600	2.44805900
O	2.00063400	-2.05579100	-1.31813100
N	3.57583600	0.10124900	0.95385600
C	4.70637000	0.74251300	1.59781500
H	4.33910300	1.57982600	2.18926500
H	5.24158100	0.04984300	2.25175500
H	5.39269900	1.11365900	0.83512100
C	4.63909100	-1.90122900	-0.07546700
H	5.63152800	-1.54796300	0.20938700
H	4.26144700	-2.54966500	0.72531700
O	4.77696700	-2.60373100	-1.29208900
H	3.88333700	-2.80435200	-1.60399100

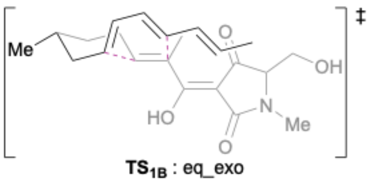

$\mathbf{TS_{1B}}$: eq_exo

Electronic Energy (EE) [A.U.] : −1212.436297

EE + Zero-point Energy [A.U.] : −1211.937325

Gibbs Free energy [A.U.] : −1211.993123

C	-5.32918700	-0.43674500	0.15986400
C	-4.62395300	-1.78970000	0.06070100
C	-4.48561400	0.55299900	0.96977500
H	-4.52837700	-2.21212100	1.06961600
H	-5.24837500	-2.48081100	-0.51291300
H	-5.46041500	-0.04464200	-0.85621200
H	-4.32400400	0.13193400	1.96875100
H	-5.04734400	1.48245200	1.11468300
C	-6.71006400	-0.59154900	0.79367300
H	-7.23543100	0.36558300	0.83438500
H	-7.32439700	-1.29510700	0.22700600
H	-6.61875500	-0.97067100	1.81643000
C	-3.24326600	-1.71177000	-0.58809600
H	-3.33476300	-1.33118100	-1.60879500
H	-2.82512200	-2.72194800	-0.67114900
C	-2.25275300	-0.87975800	0.20172800
H	-2.32746900	-1.01095700	1.27946300
C	-3.13856400	0.90038200	0.36284700
H	-2.40111300	1.23729900	1.08551300
C	-3.04977900	1.45196100	-0.91580200
H	-3.90310500	1.36309800	-1.58261200
C	-1.86467600	1.98741700	-1.45502800
H	-1.84894400	2.21422900	-2.51763100
C	-0.69593500	2.12468300	-0.74602300
H	-0.70741600	2.04188100	0.33600800
C	0.56440000	2.46390300	-1.34949500
H	0.60421700	2.54470600	-2.43402400
C	1.68457800	2.62890400	-0.62174300
H	1.60774000	2.55679700	0.46204900
C	3.03505000	2.93428000	-1.17724600
H	3.02808300	2.95245600	-2.26760100
H	3.39108900	3.90163300	-0.81001400
H	3.76254900	2.18936000	-0.83867600
C	-0.93127100	-0.72610800	-0.28161300
C	-0.65511500	-0.94014900	-1.74072600
H	-0.61722700	-2.00063900	-2.00490600
H	-1.46503000	-0.48538400	-2.31969500
H	0.28092200	-0.47822900	-2.04549400
C	0.07640900	-0.36102400	0.63790500
C	1.47688700	-0.40120600	0.43986700
C	2.37421900	0.25167300	1.39260300
C	2.29822200	-1.11404500	-0.48920500
O	2.03093300	0.86461000	2.42129300

O	2.03128100	-1.97501800	-1.32842000
C	4.60249200	-1.98955800	0.02618300
H	4.15149300	-2.58482100	0.83084700
H	5.61320500	-1.71204800	0.33084700
C	4.80248900	0.66498900	1.66751000
H	5.34163200	-0.08881700	2.24694900
H	5.48298500	1.10321900	0.93439000
H	4.45021500	1.44390300	2.34130200
N	3.65637700	0.09722100	0.98781300
C	3.75594900	-0.73168400	-0.19425700
H	4.16614600	-0.17024100	-1.04413200
O	4.71128400	-2.73256000	-1.17014700
H	3.80957900	-2.82962600	-1.51204100
O	-0.35399300	0.09883900	1.82517200
H	0.43364700	0.43380500	2.32925300

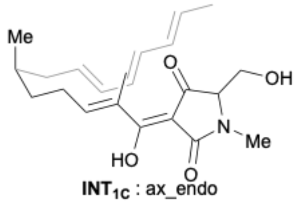

INT_{1C} : ax_endo

Electronic Energy (EE) [A.U.] : −1212.449931

EE + Zero-point Energy [A.U.] : −1211.952223

Gibbs Free energy [A.U.] : −1212.012568

C	-4.67110000	-0.29490600	-1.24596900
C	-5.40863000	-0.10327400	0.09422800
C	-4.80647800	-0.85508000	1.29459600
H	-5.52946700	-0.77221800	2.11162600
H	-6.39407700	-0.55799900	-0.05798000
H	-4.56653900	-1.36358800	-1.46052900
H	-5.32947200	0.11570000	-2.02333300
H	-4.74053500	-1.92149900	1.05181000
C	-5.65339200	1.37558800	0.40653500
H	-6.16418400	1.86475100	-0.42693400
H	-6.27995400	1.47946800	1.29595200
H	-4.72748600	1.92440000	0.59172600
C	-3.44384800	-0.38114500	1.82297400
H	-3.39012900	0.71036700	1.84279900
H	-3.35934600	-0.68839900	2.87385400
C	-2.26284900	-0.96283800	1.10910600
H	-2.44723700	-1.87691700	0.55310300
C	-3.33094900	0.37172800	-1.37435300
H	-3.23233600	1.36686000	-0.94414200
C	-2.28897700	-0.15570800	-2.02951500
H	-2.40893600	-1.13398400	-2.49002400
C	-0.96333000	0.44869900	-2.16031500
H	-0.30636700	0.01323200	-2.91087900
C	-0.47907700	1.44166200	-1.39382200
H	-1.10284000	1.86729500	-0.61046700
C	0.85765100	2.00019600	-1.52531100
H	1.49728300	1.57913900	-2.30088800
C	1.31936200	2.99608900	-0.75614300
H	0.66210600	3.40007400	0.01298600
C	2.67081800	3.62757300	-0.88103500
H	3.23972100	3.18983600	-1.70362000
H	2.57713900	4.70244300	-1.06056700
H	3.24756000	3.50942300	0.04201800
C	-1.00089300	-0.49117600	1.15073400
C	-0.63382200	0.78208800	1.86696800
H	-0.38291800	0.60647400	2.91430900
H	0.22446000	1.26205400	1.40113600
H	-1.47383500	1.47793100	1.83021300
C	0.00228100	-1.23404800	0.38367300
C	1.39229500	-1.09986900	0.44538200
C	2.30390700	-0.43656500	1.34760400
C	2.21647600	-1.79550800	-0.55289600
O	1.78506500	-2.55307800	-1.44274300
O	2.12967300	0.18396800	2.38694900
O	-0.48234600	-2.10179400	-0.47961400
H	0.28874100	-2.48353400	-1.00734300
N	3.51491300	-1.49453500	-0.36295700
C	4.60637400	-2.04320700	-1.14348800
H	4.20883600	-2.83257000	-1.77812700
H	5.06583900	-1.27787700	-1.77216000
H	5.36217100	-2.46021300	-0.47526300
C	3.71381300	-0.62672200	0.77857500
H	4.35784700	-1.09660300	1.52934300
C	4.28583400	0.73979000	0.37949800
H	5.25404900	0.60915200	-0.10674900
H	3.59665000	1.20411300	-0.33703700
O	4.49241100	1.56025900	1.50976100
H	3.69762600	1.50939100	2.05887600

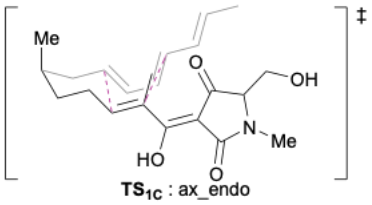

Electronic Energy (EE) [A.U.] : −1212.430298

EE + Zero-point Energy [A.U.] : −1211.931484

Gibbs Free energy [A.U.] : −1211.988317

C	4.55295000	-0.70113200	0.89963100
C	5.44112100	-0.19661000	-0.24207500
C	4.79638400	-0.47986700	-1.60178900
H	5.43600000	-0.08482000	-2.39638200
H	6.37116200	-0.77238200	-0.19415900
H	4.44846500	-1.78964000	0.82937600
H	5.05559900	-0.49643700	1.85136200
H	4.73485200	-1.56357900	-1.75199700
C	5.81299100	1.27895600	-0.07013500
H	6.31331500	1.43971300	0.88801100
H	6.49209200	1.59710500	-0.86487500
H	4.94102400	1.93659500	-0.10059000
C	3.39728000	0.11602700	-1.74270300
H	3.42887400	1.19977500	-1.60755600
H	3.03841400	-0.04647600	-2.76578600
C	2.37147600	-0.51694300	-0.81826800
H	2.49103500	-1.58974500	-0.70515500
C	3.16129800	-0.09049300	0.96133800
H	3.10869100	0.98181500	0.78391700
C	2.27944200	-0.60754700	1.91451000
H	2.53401100	-1.56451900	2.36326800
C	1.02480800	-0.06404400	2.23698100
H	0.38336600	-0.62893700	2.90774800
C	0.51015100	1.04960600	1.62571000
H	1.15600400	1.68168200	1.02653600
C	-0.83842000	1.52444700	1.82032300
H	-1.50686200	0.91915400	2.43020300
C	-1.27645100	2.67442800	1.27988400
H	-0.58166700	3.25186700	0.67197600
C	-2.64121100	3.25023600	1.46036700
H	-3.23940100	2.65712300	2.15340700
H	-2.57273900	4.27261900	1.84232700
H	-3.16622800	3.30745300	0.50059700
C	1.02854500	-0.06488400	-0.89414500
C	0.72163600	1.28084700	-1.48838100
H	0.73490500	1.25426900	-2.58248300
H	-0.25450600	1.64163700	-1.17987000
H	1.47677300	2.00824600	-1.17649300
C	0.03091400	-0.95478200	-0.43056400
C	-1.39361700	-0.85452400	-0.52710800
C	-2.28390700	-0.05730700	-1.30264400
C	-2.21412000	-1.79504100	0.23233200
C	-3.71212700	-0.47018100	-0.92393800
H	-4.23681200	-0.82868600	-1.81724500
O	-2.11096200	0.82424600	-2.15213100
O	-1.78961000	-2.71841500	0.95977400
N	-3.52648000	-1.53242800	0.03642100
C	-4.61219800	-2.34666000	0.54069300
H	-4.18476000	-3.16874200	1.11121400
H	-5.26877500	-1.76647700	1.19227700
H	-5.19888100	-2.74861400	-0.28921100
C	-4.45700300	0.72930700	-0.32927600
H	-5.45813100	0.43032600	-0.01257200
H	-3.90279500	1.06824900	0.55507200
O	-4.60374100	1.76631900	-1.27770000
H	-3.76327700	1.82981100	-1.75769100
O	0.49891900	-2.04646000	0.17592500
H	-0.28087800	-2.52992800	0.58840900

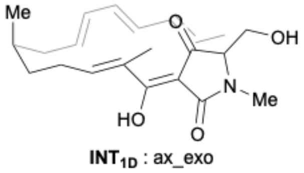

Electronic Energy (EE) [A.U.] : −1212.452933

EE + Zero-point Energy [A.U.] : −1211.954120

Gibbs Free energy [A.U.] : −1212.012727

C	-4.81053100	1.28834000	-0.11476700
C	-5.36657500	-0.15428300	-0.15850000
C	-4.75935500	-1.07253700	-1.24029900
H	-5.51250900	-1.83631400	-1.45318500
H	-6.41685700	-0.03380700	-0.44709800
H	-5.02030100	1.76431500	-1.07761600
H	-5.38217500	1.83199400	0.64455800
H	-4.62942400	-0.50596400	-2.16949400

C	-5.35607900	-0.79928400	1.22699000
H	-5.97591000	-0.22449900	1.91970100
H	-5.74684400	-1.81988100	1.18662900
H	-4.34694600	-0.84004700	1.64402700
C	-3.45181500	-1.82635400	-0.91281400
H	-3.45401000	-2.18278900	0.11790800
H	-3.44121000	-2.72628800	-1.54104600
C	-2.18576900	-1.10082400	-1.23442700
H	-2.13735300	-0.66184500	-2.22843300
C	-3.34458800	1.41106700	0.18365600
H	-2.66243200	1.38692800	-0.66260500
C	-2.82825500	1.52093000	1.41476300
H	-3.50249200	1.49850500	2.26978700
C	-1.39720400	1.62442800	1.70932000
H	-1.06344100	1.21477400	2.66102700
C	-0.48486800	2.18121000	0.89576000
H	-0.81307500	2.64574500	-0.03226000
C	0.94448600	2.21557000	1.16636800
H	1.29636900	1.69549100	2.05714900
C	1.82218500	2.83774800	0.37007400
H	1.44338300	3.34438300	-0.51616600
C	3.30068400	2.89104200	0.58729300
H	3.82777800	2.54129100	-0.30461400
H	3.63163200	3.91867400	0.76664500
H	3.60402500	2.27691700	1.43770900
C	-1.09743300	-1.01814600	-0.44990800
C	-1.06318000	-1.53330700	0.96730400
H	-0.21125200	-1.12898200	1.51100800
H	-1.97265500	-1.20700000	1.47772900
H	-1.00498200	-2.62128900	1.01175300
C	0.07515100	-0.34240700	-1.01922100
C	1.40329300	-0.62528200	-0.73576500
C	2.03981000	-1.67239100	0.02712300
C	2.47099200	0.18611700	-1.33417800
C	3.54234900	-1.37613700	0.01590600
H	4.09028600	-2.23395400	-0.38929300
O	2.30492300	1.09248400	-2.16641000
O	-0.18760500	0.61428200	-1.88808300
H	0.68474900	0.99063500	-2.20783100
N	3.66462600	-0.22076600	-0.85128900
C	4.94476800	0.25797900	-1.33471600
H	5.50374700	-0.56234900	-1.79101600
H	4.76084600	1.02886600	-2.08080100
H	5.53320200	0.68334900	-0.51956200
O	1.58471100	-2.63786500	0.62120000
C	4.00954300	-1.10244200	1.45144500
O	3.96048800	-2.28015900	2.22842900
H	3.38451300	-0.30847100	1.88128000
H	5.04502200	-0.75873800	1.44710700
H	3.09015500	-2.68043600	2.09650900

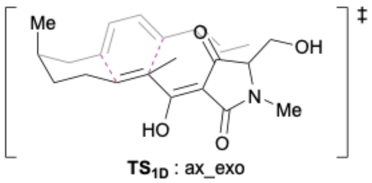

TS_{1D} : ax_exo

Electronic Energy (EE) [A.U.] : −1212.430416

EE + Zero-point Energy [A.U.] : −1211.930944

Gibbs Free energy [A.U.] : −1211.986602

C	4.50148900	0.85738300	0.98554500
C	5.40519000	-0.28443400	0.48921700
C	4.71992300	-1.64323200	0.66784300
H	5.37556500	-2.43038700	0.28397100
H	6.27499100	-0.27124800	1.15428800
H	4.35074500	0.71434000	2.06021800
H	5.03263700	1.80884000	0.87453800
H	4.58082000	-1.83966500	1.73699200
C	5.94099700	-0.10632400	-0.93584300
H	6.30170900	0.91239600	-1.09993700
H	6.77598200	-0.79106400	-1.10294000
H	5.19055500	-0.32599900	-1.69757300
C	3.36098100	-1.73954700	-0.02556200
H	3.45870300	-1.57129700	-1.10050600
H	2.97504500	-2.75935400	0.08821400
C	2.32669800	-0.80441900	0.56876300
H	2.39062500	-0.71698400	1.65181900
C	3.12556600	1.00450100	0.35214000
H	2.38940300	1.42447100	1.03249600
C	2.95790300	1.34575100	-0.99241300
H	3.79209200	1.22582200	-1.67453200
C	1.72502500	1.73304200	-1.55386900
H	1.66456500	1.80670600	-2.63632400
C	0.57722900	1.92617600	-0.82599000
H	0.63029800	1.99539900	0.25528700
C	-0.71310400	2.16774200	-1.41682200

H	-0.80047900	2.10312200	-2.49964900
C	-1.79464800	2.45095100	-0.66788900
H	-1.66630200	2.51583700	0.41149900
C	-3.16378900	2.72762200	-1.19176200
H	-3.90051600	2.09721900	-0.68380600
H	-3.44560000	3.76455800	-0.98310400
H	-3.22796200	2.55786800	-2.26754700
C	1.00698700	-0.81783300	0.05680500
C	0.78113700	-1.28714100	-1.34891600
H	-0.18280300	-0.96256100	-1.73379000
H	1.56635300	-0.86221100	-1.98413600
H	0.84063000	-2.37519000	-1.43959200
C	-0.03961500	-0.36968500	0.88989000
C	-1.42982600	-0.56469400	0.70014400
C	-2.16991000	-1.51467900	-0.06972400
C	-2.39610800	0.19306800	1.49125200
C	-3.65563700	-1.20164900	0.13583100
H	-4.15986800	-2.07533100	0.56604000
O	-2.12858900	0.98455700	2.41414100
O	-1.82241100	-2.45273000	-0.78807600
O	0.33050400	0.32428600	1.97943500
H	-0.49057400	0.67029100	2.41673200
N	-3.65504600	-0.09351500	1.06829900
C	-4.84848100	0.32339000	1.77682900
H	-5.24928600	-0.49582200	2.38129800
H	-4.58757800	1.15607200	2.42679800
H	-5.61279200	0.65075600	1.07057400
C	-4.28874400	-0.87183300	-1.22019700
H	-5.31019500	-0.51138100	-1.08565600
H	-3.70222300	-0.07887400	-1.70220100
O	-4.35029100	-2.02843500	-2.03086900
H	-3.47849900	-2.44807700	-1.98625700

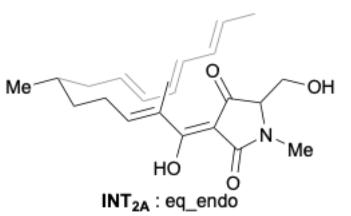

INT$_{2A}$: eq_endo

]Electronic Energy (EE) [A.U.] : −1212.451496

EE + Zero-point Energy [A.U.] : −1211.954252

Gibbs Free energy [A.U.] : −1212.014577

C	-4.65236200	0.04406500	-1.15883800
C	-5.35780700	0.28347000	0.18695400
C	-4.76690000	-0.49043900	1.37501500
H	-5.45481600	-0.36027300	2.21528300
H	-4.61689300	-1.03073300	-1.37319600
H	-5.28700400	0.49285200	-1.93446200
H	-4.76987100	-1.56269000	1.14195900
C	-3.36795500	-0.06829100	1.84568500
H	-3.26925200	1.02074400	1.78814000
H	-3.26763000	-0.31026700	2.91166600
C	-2.23409000	-0.74103500	1.13314700
H	-2.48813400	-1.62727200	0.56056600
C	-3.27976100	0.63957000	-1.28829900
H	-3.13534700	1.61497200	-0.82352900
C	-2.27258500	0.08180300	-1.97174100
H	-2.44737400	-0.87589100	-2.45720300
C	-0.92044200	0.62274000	-2.10482200
C	-0.37607700	1.56851300	-1.31907700
H	-0.96663600	2.00378600	-0.51535300
C	0.98591200	2.06039100	-1.45791600
H	1.59054900	1.62553100	-2.25375500
C	1.51156700	3.01193300	-0.67383400
H	0.88906900	3.43157100	0.11553200
C	-0.93916100	-0.37050900	1.18794500
C	-0.47891600	0.85146400	1.93769700
H	-0.19220600	0.61765300	2.96414600
H	0.38319200	1.30771200	1.45475100
H	-1.28246400	1.58868100	1.96839400
C	0.00557600	-1.16414400	0.39815300
C	-6.83938500	-0.07460300	0.04477600
H	-7.38368000	0.12153700	0.97122600
H	-7.30928000	0.50070700	-0.75612300
H	-6.95210700	-1.13729300	-0.19334000
H	-5.28450700	1.35500000	0.41626800
C	2.89263000	3.57384800	-0.80763700
H	2.85370200	4.65672400	-0.95658000
H	3.47893800	3.39931200	0.10034000
H	3.42223700	3.12947500	-1.65259300
H	-0.29649600	0.17613400	-2.87667000
C	1.40242000	-1.11169500	0.43385600
C	2.37151000	-0.51066400	1.32060600
C	2.16363700	-1.83735700	-0.59319400
C	3.75416700	-0.76944600	0.71380100

H	4.38970300	-1.28430200	1.44202000
O	1.67054300	-2.55850400	-1.48138500
O	2.25930700	0.10469400	2.37140300
N	3.48057800	-1.60861800	-0.43328800
C	4.52163100	-2.20602400	-1.24616000
H	5.26247300	-2.68330000	-0.60184500
H	4.06436500	-2.95503200	-1.88935600
H	5.01590700	-1.45638000	-1.86723000
C	4.39070200	0.56975500	0.31945400
H	5.33657100	0.39324700	-0.19545800
H	3.70977600	1.08434400	-0.37035000
O	-0.54445400	-1.99018700	-0.46686800
H	0.19354300	-2.40880600	-1.01453500
O	4.67351400	1.35788700	1.45612100
H	3.89247500	1.34137900	2.02671300

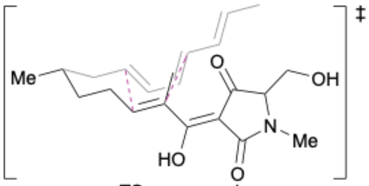

TS$_{2A}$: eq_endo

Electronic Energy (EE) [A.U.] : −1212.432483

EE + Zero-point Energy [A.U.] : −1211.934170

Gibbs Free energy [A.U.] : −1211.991267

C	4.53646000	-0.30514600	0.92164200
C	5.37887700	0.25811000	-0.22212300
C	4.77336600	-0.10150500	-1.57690700
H	5.38905900	0.32498900	-2.37423200
H	4.52447100	-1.40080300	0.85983600
H	5.02014600	-0.05239200	1.87186200
H	4.79929100	-1.19151100	-1.70356300
C	3.34000200	0.39685700	-1.73056500
H	3.31364100	1.48143300	-1.59057400
H	2.99414500	0.21347000	-2.75454800
C	2.35119900	-0.29187900	-0.80646000
H	2.53845400	-1.35311900	-0.67911800
C	3.10649200	0.20459200	0.97253900
H	2.99376000	1.26997000	0.77593800
C	2.25444100	-0.35498800	1.92798200
H	2.56638200	-1.28873700	2.38918400
C	0.96652300	0.11293900	2.23817500
C	0.38686100	1.18506500	1.61125700
H	0.99530100	1.84813600	1.00644500
C	-0.98891600	1.57881800	1.79668600
H	-1.62059100	0.94162400	2.41327700
C	-1.49536600	2.69329600	1.24192300
H	-0.83641400	3.30496200	0.62755200
C	0.98328000	0.07373900	-0.89204200
C	0.59479000	1.38967400	-1.50504200
H	0.61504000	1.34953800	-2.59876300
H	-0.40378500	1.69183800	-1.20579100
H	1.30082500	2.16746800	-1.20017600
C	0.04173100	-0.87249700	-0.42134900
C	6.81887100	-0.23852100	-0.11702100
H	7.43461800	0.17361600	-0.91987800
H	7.26714400	0.04647500	0.83782800
H	6.85130400	-1.32991700	-0.19330600
H	5.37941000	1.35282600	-0.13218900
C	-2.89325800	3.18621700	1.41460600
H	-2.88831900	4.21658900	1.78082700
H	-3.42128600	3.19583300	0.45480800
H	-3.45331300	2.56790100	2.11742900
H	0.35807000	-0.48260700	2.91311600
C	-1.38607200	-0.86326600	-0.52100500
C	-2.32452200	-0.12826700	-1.30144600
C	-2.14675900	-1.84751400	0.24566100
C	-3.72416400	-0.62691400	-0.91956600
H	-4.22552800	-1.02367600	-1.81010600
O	-1.66566200	-2.73707700	0.98004300
O	-2.20735400	0.75685700	-2.15661100
N	-3.47283900	-1.66871700	0.04818900
C	-4.50608000	-2.54569400	0.55768100
H	-5.06587500	-2.98909800	-0.26965900
H	-4.02893500	-3.33568500	1.13398700
H	-5.19804600	-2.00316400	1.20500000
C	-4.54216000	0.52822800	-0.33322500
H	-5.52202200	0.16967000	-0.01189800
H	-4.00878600	0.90848900	0.54710400
O	0.57588300	-1.92623500	0.19698800
H	-0.17315300	-2.45501200	0.61172000
O	-4.75509200	1.54592200	-1.28991300
H	-3.92090500	1.65811300	-1.77189600

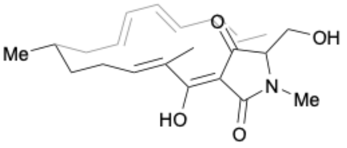

INT$_{2B}$: eq_exo

Electronic Energy (EE) [A.U.] : −1212.453198

EE + Zero-point Energy [A.U.] : −1211.955180

Gibbs Free energy [A.U.] : −1212.014515

C	4.83115700	1.13543100	-0.12795900
C	5.33222400	-0.32263700	-0.11543200
C	4.68891400	-1.21760800	0.96115800
H	5.40550600	-2.01046800	1.19074000
H	5.12444200	1.60228200	0.81967400
H	5.36732100	1.66193300	-0.92376500
H	4.56494900	-0.64547300	1.88898700
C	3.36018300	-1.90531400	0.57963600
H	3.34433500	-2.12268500	-0.49052600
H	3.33223000	-2.87850400	1.08659300
C	2.11640300	-1.19316700	1.00379800
H	2.12164700	-0.80134600	2.01832500
C	3.35383200	1.30508500	-0.33329200
H	2.71997200	1.29403100	0.55015700
C	2.78021600	1.44559700	-1.53535400
H	3.41279700	1.40808300	-2.42139800
C	1.34292800	1.60736600	-1.76399100
C	0.48274500	2.16146900	-0.89361600
H	0.86087100	2.57684400	0.03864100
C	-0.95376000	2.25201800	-1.10719900
H	-1.35414000	1.78537000	-2.00703300
C	-1.78309900	2.86076900	-0.25080500
H	-1.35826600	3.31365400	0.64364600
C	0.99005600	-1.06969800	0.28061600
C	0.87702700	-1.53006600	-1.15100500
H	0.00971300	-1.08737700	-1.63775300
H	1.76905300	-1.20693900	-1.69286700
H	0.79115400	-2.61426500	-1.23104000
C	-0.14346100	-0.40428000	0.93401600
C	6.85215100	-0.31109500	0.06860300
H	7.26714800	-1.31693200	-0.02684600
H	7.33574000	0.33106200	-0.67141000
H	7.11046400	0.06719000	1.06306300
H	5.10600000	-0.75660700	-1.09736000
C	-3.26585300	2.96788700	-0.41362600
H	-3.77475200	2.58317400	0.47455000
H	-3.57117400	4.01295900	-0.52454500
H	-3.61422400	2.41181900	-1.28633500
H	0.95706300	1.24590000	-2.71538800
C	-1.48841000	-0.65062800	0.69823900
C	-2.18058400	-1.65316300	-0.07575100
C	-2.51101400	0.16133800	1.36989500
C	-3.67458000	-1.32698800	0.01293800
H	-4.22092200	-2.18666500	0.41662200
O	-2.28937400	1.03247100	2.22669200
O	-1.77362600	-2.60500300	-0.72435500
N	-3.73262100	-0.19863400	0.92149700
C	-4.97969600	0.30197700	1.46546800
H	-5.55403400	-0.51845700	1.90189400
H	-4.74582800	1.03111600	2.23907700
H	-5.57648900	0.78455700	0.68910800
C	-4.20312900	-0.99991900	-1.38962900
H	-5.23073100	-0.63890200	-1.32523800
H	-3.58455600	-0.20417700	-1.82530400
O	0.17498200	0.50959300	1.82956700
H	-0.67555600	0.88975400	2.20050500
O	-4.21340000	-2.15375500	-2.20317100
H	-3.34554600	-2.57318800	-2.12536300

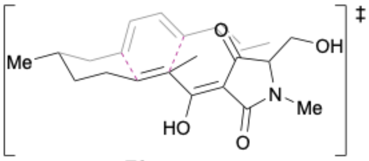

TS$_{2B}$: eq_exo

Electronic Energy (EE) [A.U.] : −1212.435466

EE + Zero-point Energy [A.U.] : −1211.936460

Gibbs Free energy [A.U.] : −1211.992309

C	5.34430400	-0.20819000	0.02394800
C	4.73469400	-1.59853500	0.20231400
H	5.37197300	-2.33889300	-0.28989400
H	4.72876700	-1.84638300	1.27185700
C	3.31716100	-1.71581200	-0.35365000
H	3.32369900	-1.51414000	-1.42800600
H	2.96630400	-2.74779500	-0.23500300
C	2.31365700	-0.82086400	0.34672000
H	2.44709300	-0.76849200	1.42576400
C	3.07673300	1.00527200	0.15979000

H	2.36463600	1.41543600	0.86993300
C	2.87073400	1.33619000	-1.18026500
H	3.68374200	1.18681900	-1.88565700
C	1.62287600	1.72454500	-1.70710900
C	0.50134700	1.92045800	-0.93993600
H	0.59314200	1.98903600	0.13888700
C	-0.81115300	2.15801400	-1.48078100
H	-0.94113900	2.09349400	-2.55927100
C	-1.86240500	2.43861400	-0.68869000
H	-1.69054600	2.50375500	0.38466900
C	0.96304900	-0.83293400	-0.08031400
C	0.64386200	-1.28864700	-1.47325600
H	-0.34477500	-0.96225200	-1.78747300
H	1.38099300	-0.85610400	-2.15794500
H	0.69860500	-2.37569600	-1.57830300
C	-0.02871600	-0.38662900	0.81695100
C	6.76853000	-0.16618700	0.57357100
H	7.39741400	-0.91652400	0.08881600
H	7.22360000	0.81464800	0.41673000
H	6.76730200	-0.37019700	1.64897200
H	5.38416800	0.01297200	-1.04986900
C	-3.25167500	2.71481300	-1.15645600
H	-3.96779400	2.09107800	-0.61242000
H	-3.52121200	3.75432100	-0.94407800
H	-3.36191800	2.53727000	-2.22721700
H	1.52621700	1.79060300	-2.78727800
C	-1.42966600	-0.56864700	0.70569600
C	-2.21955900	-1.51566000	-0.01595800
C	-2.34335300	0.20363300	1.54327500
C	-3.68874200	-1.18907700	0.27135000
H	-4.17223500	-2.05431600	0.74092400
O	-2.01788100	0.99692700	2.44561900
O	-1.92050900	-2.46089300	-0.74706800
N	-3.62672400	-0.07064100	1.18970500
C	-4.77382400	0.35834100	1.96468000
H	-4.47440300	1.20358700	2.58087700
H	-5.58148000	0.67145500	1.30173400
H	-5.13448200	-0.44933500	2.60879400
C	-4.39848700	-0.87235600	-1.04919600
H	-5.40732200	-0.50107500	-0.85985300
H	-3.83534700	-0.09195800	-1.57729500
O	-4.51692700	-2.04014900	-1.83737300
H	-3.64613800	-2.46430400	-1.83995800
O	0.40902900	0.29828300	1.88733700
H	-0.38210800	0.65331200	2.36911800
C	4.47969400	0.84830900	0.71812900
H	4.40773500	0.59036400	1.78098900
H	4.97852800	1.82254900	0.67052500

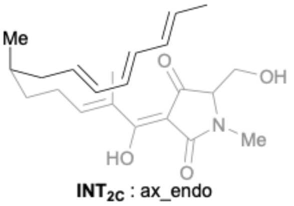

INT$_{2C}$: ax_endo

Electronic Energy (EE) [A.U.] : −1212.451783

EE + Zero-point Energy [A.U.] : −1211.953449

Gibbs Free energy [A.U.] : −1212.013260

C	5.37517800	0.20511900	-0.16689200
C	4.85200000	1.47245200	0.53715800
C	4.75499800	-1.12410100	0.30215800
H	4.80971600	1.29402200	1.61751700
H	5.60935000	2.24680800	0.38468700
H	4.90646100	-1.24186100	1.38000700
H	5.33199700	-1.92508500	-0.18003100
C	3.50779300	2.06460800	0.06950400
H	3.42876800	2.05322400	-1.01844700
H	3.51188300	3.12519400	0.35384800
C	2.29664300	1.48359000	0.72158700
H	2.36844700	1.35561300	1.79834000
C	3.30462500	-1.33818000	-0.01779500
H	2.94956100	-0.96061400	-0.97503200
C	2.44706700	-1.99406500	0.77424300
H	2.80123700	-2.34321600	1.74189800
C	1.03621100	-2.24727900	0.48035600
C	0.42967500	-2.11150200	-0.71209100
H	1.00851700	-1.83905000	-1.59281500
C	-0.99900200	-2.30153700	-0.90651500
H	-1.58199200	-2.57926900	-0.02743000
C	-1.61950300	-2.15237600	-2.08438700
H	-1.02421500	-1.87484900	-2.95315900
C	1.12804100	1.17946000	0.12687600
C	0.92229200	1.23903100	-1.36671800
H	0.66015400	2.23964400	-1.71195300
H	1.83917100	0.93125000	-1.87354900

H	0.12641100	0.56352800	-1.67813100
C	0.06184300	0.66601800	0.99063900
C	5.35577400	0.33670300	-1.69182100
H	5.83544900	1.26773200	-2.00553000
H	5.89369500	-0.49480000	-2.15353800
H	4.34175700	0.33755900	-2.09897700
H	6.42887500	0.13865200	0.12680400
C	-3.08198700	-2.37730700	-2.31130600
H	-3.54194500	-1.52465300	-2.81873800
H	-3.24239900	-3.24492000	-2.95889600
H	-3.60576500	-2.55339400	-1.36903300
H	0.42175800	-2.54992400	1.32637800
C	-1.28867700	0.58691100	0.67369200
C	-2.18683300	-0.21730600	1.50994100
C	-2.09270200	1.17904800	-0.36597200
C	-3.50594700	0.60071000	-0.22408100
H	-3.74636600	-0.00000700	-1.10879600
O	-1.86049000	-0.81356900	2.55089000
O	-1.84161600	2.06049000	-1.17641900
N	-3.41853700	-0.22858100	0.96178400
C	-4.53696900	-0.98882000	1.48387500
H	-4.15142100	-1.74043400	2.17009400
H	-5.24211800	-0.34705700	2.01711200
H	-5.05610300	-1.48228500	0.66025100
C	-4.53281600	1.73107400	-0.09122200
H	-5.51868800	1.31385100	0.12047000
H	-4.24568900	2.37916800	0.74663800
O	0.45172800	0.20050100	2.16297100
H	-0.33547200	-0.23254400	2.60071000
O	-4.63491900	2.45617900	-1.29843100
H	-3.73511900	2.70548200	-1.55266000

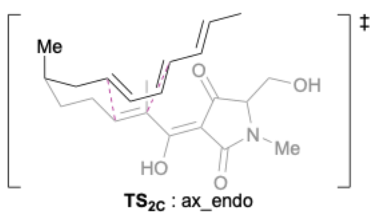

TS$_{2C}$: ax_endo

Electronic Energy (EE) [A.U.] : −1212.431120

EE + Zero-point Energy [A.U.] : −1211.932270

Gibbs Free energy [A.U.] : −1211.989428

C	-5.45937700	0.44174800	-0.25468900
C	-4.73328300	1.72530200	-0.66326300
C	-4.64737800	-0.79094000	-0.66057500
H	-4.65968000	1.76561000	-1.75583600
H	-5.32281400	2.59247700	-0.35192800
H	-4.55170200	-0.82153100	-1.75163600
H	-5.20461100	-1.68888700	-0.37097900
C	-3.33214800	1.83673700	-0.06826700
H	-3.37919100	1.80403000	1.02248400
H	-2.91261500	2.81811600	-0.31897800
C	-2.34743700	0.80753800	-0.59753400
H	-2.43017600	0.62858000	-1.66517400
C	-3.25418800	-0.89463200	-0.05842000
H	-3.17325400	-0.65790200	1.00095700
C	-2.44196400	-1.92614300	-0.53728200
H	-2.73687400	-2.39217400	-1.47449600
C	-1.20659900	-2.31695600	0.00216000
C	-0.62981100	-1.72525400	1.09651000
H	-1.21876800	-1.08191900	1.74173700
C	0.73457300	-1.96607700	1.49055100
H	1.34004800	-2.59476200	0.83932100
C	1.29048600	-1.40588600	2.57795500
H	0.66643400	-0.78131700	3.21510400
C	-1.02486900	0.83087700	-0.08649500
C	-0.74848100	1.43016200	1.26403900
H	-0.68617300	2.52148800	1.22631700
H	-1.55559100	1.17388100	1.95687700
H	0.18650100	1.06200900	1.67802000
C	-0.00403000	0.27798800	-0.88962700
C	-5.82621700	0.42402600	1.23182300
H	-6.43934300	1.29227300	1.48539300
H	-6.39594200	-0.47617100	1.47507300
H	-4.94695300	0.44152300	1.88017100
H	-6.39594900	0.40004300	-0.82026600
C	2.71247100	-1.58428400	2.99374000
H	3.19295000	-0.61274300	3.14223000
H	2.76881700	-2.10663800	3.95378200
H	3.27767300	-2.15169000	2.25223400
H	-0.61620100	-3.03411800	-0.56150500
C	1.40177900	0.30260700	-0.66102500
C	2.25314100	-0.65459200	-1.35934000
C	2.24802600	1.13208900	0.12440400
C	3.67338400	0.57184700	0.00135700
H	4.00143600	0.18864900	0.97614800

O	1.87344900	-1.49849700	-2.19538500
O	2.03983300	2.18429100	0.74103700
N	3.53868300	-0.49868600	-0.96085200
C	4.62501700	-1.38232100	-1.32674800
H	4.21563800	-2.19739200	-1.92050700
H	5.38138500	-0.85995900	-1.91688200
H	5.09427800	-1.79021800	-0.42784600
C	4.63951500	1.66909300	-0.45478400
H	5.63209600	1.24940500	-0.62815900
H	4.27439400	2.09112200	-1.40026300
O	-0.42167200	-0.36258000	-1.98868400
H	0.34265000	-0.89937600	-2.33195600
O	4.76845800	2.66399000	0.54072700
H	3.86453000	2.89320100	0.80801300

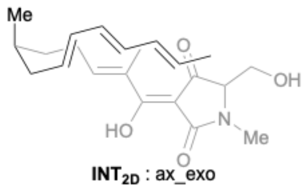

INT_{2D} : ax_exo

Electronic Energy (EE) [A.U.] : −1212.452465

EE + Zero-point Energy [A.U.] : −1211.953637

Gibbs Free energy [A.U.] : −1212.012237

C	-5.36397900	-0.52494500	0.33764400
C	-4.58181100	-1.49332800	1.24995500
C	-4.95054300	0.96258700	0.43088300
H	-4.43131800	-1.02963300	2.23163100
H	-5.23625300	-2.35327000	1.41760600
H	-5.12366700	1.30181400	1.45678400
H	-5.62970900	1.52873700	-0.21492700
C	-3.23830900	-2.05733400	0.73841800
H	-3.28725900	-2.27717900	-0.32867500
H	-3.09199300	-3.02463400	1.23624300
C	-2.02449000	-1.25180300	1.07155700
H	-1.94881700	-0.92392400	2.10594000
C	-3.53117100	1.26297000	0.04706000
H	-2.78271400	1.20938000	0.83373900
C	-3.12874400	1.55907100	-1.19585100
H	-3.86607200	1.56845500	-1.99728100
C	-1.74013300	1.82461000	-1.57661400
C	-0.80956900	2.35812100	-0.76797400
H	-1.09422000	2.68393500	0.23074800
C	0.58719900	2.53247000	-1.13472600
H	0.89952000	2.14182700	-2.10291400
C	1.48754200	3.11874700	-0.33688300
H	1.15450000	3.49707700	0.62820600
C	-1.00099300	-0.98092800	0.24322300
C	-1.01276000	-1.33381100	-1.22307200
H	-0.86631900	-2.40083700	-1.39344700
H	-1.97667600	-1.03779600	-1.64466500
H	-0.23357100	-0.79693400	-1.76169200
C	0.14772000	-0.27570600	0.82495600
C	-5.41098200	-1.00257600	-1.11347100
H	-5.70008600	-2.05581700	-1.16909800
H	-6.13834900	-0.41929600	-1.68367300
H	-4.44145500	-0.89239500	-1.60531800
H	-6.39231600	-0.54799400	0.71556400
C	2.93760900	3.29029300	-0.66067200
H	3.19512300	4.34957000	-0.75721200
H	3.55701900	2.88888200	0.14562100
H	3.20124500	2.78361200	-1.59138000
H	-1.45328900	1.55698400	-2.59202300
C	1.47247200	-0.41395300	0.43974400
C	2.51814500	0.38038200	1.09628300
C	2.13253600	-1.32098200	-0.46706900
C	3.61728900	-0.93144100	-0.48577300
H	3.86933200	-0.50920300	-1.46699200
N	3.71573800	0.07495300	0.55565800
O	2.33227900	1.19099500	2.01908600
O	1.71404300	-2.29170700	-1.08029200
C	4.96975100	0.68734700	0.94674400
H	4.75237800	1.49103300	1.64778600
H	5.62592100	-0.03681800	1.43382400
H	5.47427400	1.09620100	0.06825400
C	4.49658100	-2.15931400	-0.22647500
H	5.54203100	-1.85921600	-0.14272900
H	4.19411800	-2.62368900	0.72072600
O	-0.13310000	0.55122800	1.81326300
H	0.72572900	0.96161800	2.12683200
O	4.40977400	-3.06851300	-1.30320800
H	3.46860600	-3.23403800	-1.45519200

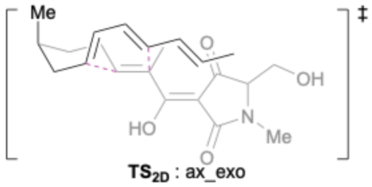

TS$_{2D}$: ax_exo

Electronic Energy (EE) [A.U.] : −1212.431135

EE + Zero-point Energy [A.U.] : −1211.931829

Gibbs Free energy [A.U.] : −1211.987671

C	-5.35600900	-0.60492900	0.63068400
C	-4.57339600	-1.92041500	0.55212900
C	-4.49412300	0.51910900	1.23258200
H	-4.34444600	-2.26567000	1.56675400
H	-5.20469800	-2.68657900	0.09241400
H	-4.25393000	0.23497400	2.26198100
H	-5.09491100	1.43262000	1.29962200
C	-3.26412000	-1.81175600	-0.23043100
H	-3.44835400	-1.48893000	-1.25780700
H	-2.80855900	-2.80640100	-0.30162700
C	-2.25408400	-0.90372500	0.44161200
H	-2.26085300	-0.97354100	1.52750600
C	-3.18394800	0.86049000	0.53921200
H	-2.42437000	1.23223800	1.22187600
C	-3.14118100	1.39092700	-0.75218300
H	-4.01627000	1.30497800	-1.38633600
C	-1.97843400	1.92481400	-1.33978800
C	-0.78581100	2.08972400	-0.67844200
H	-0.75535000	2.03232400	0.40475100
C	0.44422600	2.43429500	-1.33927900
H	0.43879500	2.49086700	-2.42612800
C	1.59043000	2.63291300	-0.66252100
H	1.55959900	2.58425300	0.42485600
C	-0.96919600	-0.74527400	-0.12816100
C	-0.78524000	-1.00759200	-1.59303600
H	-0.75186700	-2.07621600	-1.82273200
H	-1.63972800	-0.58250800	-2.13021300
H	0.12189900	-0.54621300	-1.97594900
C	0.08703800	-0.33833100	0.71757700
C	-6.01450800	-0.26175400	-0.71048800
H	-6.81790500	-0.97276400	-0.91789500
H	-6.44738100	0.74186500	-0.69545200
H	-5.31620900	-0.31627600	-1.54773300
H	-6.16943200	-0.75622800	1.34799000
C	2.91190700	2.94492600	-1.28086200
H	3.27013100	3.92379100	-0.94797900
H	3.66329700	2.21600900	-0.95988200
H	2.85813100	2.94138000	-2.37007800
H	-2.00381700	2.13349500	-2.40602900
C	1.47428200	-0.37791500	0.44328600
C	2.41924800	0.29770100	1.33233600
C	2.24782900	-1.10708400	-0.51445100
C	3.71775500	-0.71485000	-0.30537400
H	4.07997500	-0.16837700	-1.18626200
O	2.12949800	0.93234800	2.36454300
O	1.94031500	-1.98472700	-1.32154800
N	3.67839200	0.13667600	0.86398800
C	4.57955600	-1.96581000	-0.10534600
H	5.60371800	-1.67938300	0.14046500
H	4.17368800	-2.54630300	0.73344600
C	4.85738600	0.72151200	1.46889000
H	4.54019500	1.51883900	2.13854400
H	5.42592400	-0.01702500	2.03979800
H	5.49886400	1.13883800	0.68984900
O	-0.27941400	0.15794400	1.91131700
H	0.53418700	0.50882100	2.36184800
O	4.62864900	-2.73217800	-1.29077800
H	3.71090500	-2.83855500	-1.58344900
